# Triazolophostins: a library of novel and potent agonists of IP_3_ receptors†
†Electronic supplementary information (ESI) available: Synthetic procedures and spectral data for all new compounds, crystal data for disaccharide **4** and details of the docking study. CCDC 1022279. For ESI and crystallographic data in CIF or other electronic format see DOI: 10.1039/c5ob00440c



**DOI:** 10.1039/c5ob00440c

**Published:** 2015-04-14

**Authors:** Amol M. Vibhute, Vera Konieczny, Colin W. Taylor, Kana M. Sureshan

**Affiliations:** a School of Chemistry , Indian Institute of Science Education and Research , Thiruvananthapuram , Kerala-695016 , India . Email: kms@iisertvm.ac.in ; http://www.iisertvm.ac.in/scientists/kana-m-sureshan/personal-information.html ; Fax: (+) 914712597427; b Department of Pharmacology , Tennis Court Road , University of Cambridge , Cambridge CB2 1PD , UK

## Abstract

IP_3_ receptors are channels that mediate the release of Ca^2+^ from the intracellular stores of cells stimulated by hormones or neurotransmitters. Adenophostin A (AdA) is the most potent agonist of IP_3_ receptors, with the β-anomeric adenine contributing to the increased potency. The potency of AdA and its stability towards the enzymes that degrade IP_3_ have aroused interest in AdA analogs for biological studies. The complex structure of AdA poses problems that have necessitated optimization of synthetic conditions for each analog. Such lengthy one-at-a-time syntheses limit access to AdA analogs. We have addressed this problem by synthesizing a library of triazole-based AdA analogs, triazolophostins, by employing click chemistry. An advanced intermediate having all the necessary phosphates and a β-azide at the anomeric position was reacted with various alkynes under Cu(i) catalysis to yield triazoles, which upon deprotection gave triazolophostins. All eleven triazolophostins synthesized are more potent than IP_3_ and some are equipotent with AdA in functional analyses of IP_3_ receptors. We show that a triazole ring can replace adenine without compromising the potency of AdA and provide facile routes to novel AdA analogs.

## Abstract 2

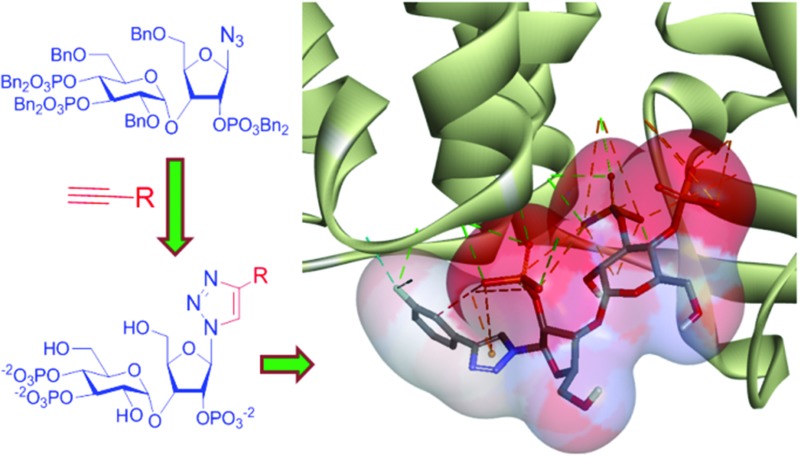
IP_3_R initiate most cellular Ca^2+^ signaling. AdA is the most potent agonist of IP_3_R. The structural complexity of AdA makes synthesis of its analogs cumbersome. We report an easy method for generating a library of potent triazole-based analogs of AdA, triazolophostins, which are the most potent AdA analogs devoid of a nucleobase.

## I. Introduction

**Fig. 1:**
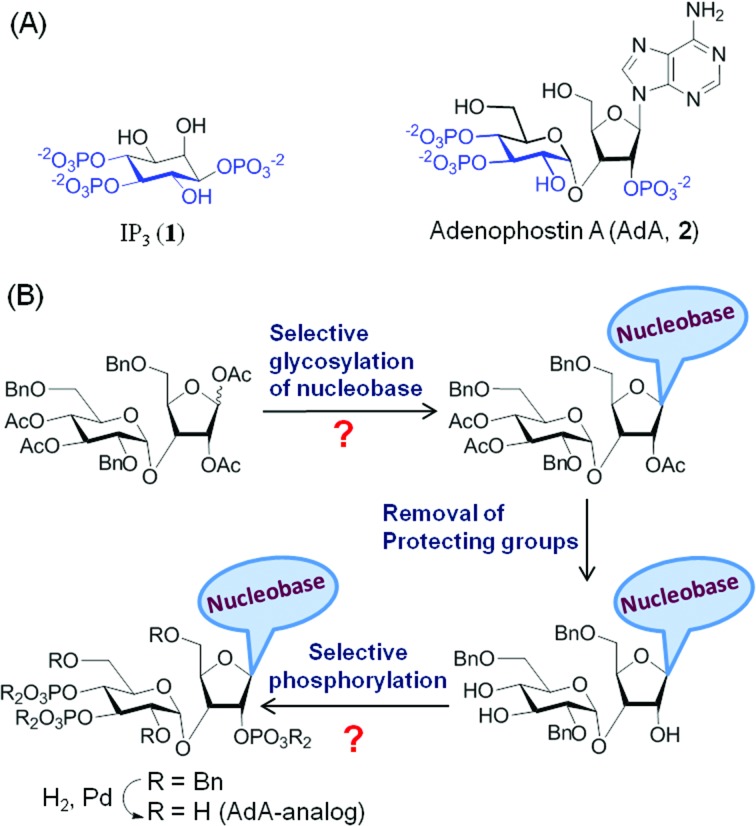
(A) Molecular structures of IP_3_ (**1**) and AdA (**2**); (B) conventional synthesis of AdA-analogs. The question marks represent the complexity of conventional syntheses.

**Fig. 2:**
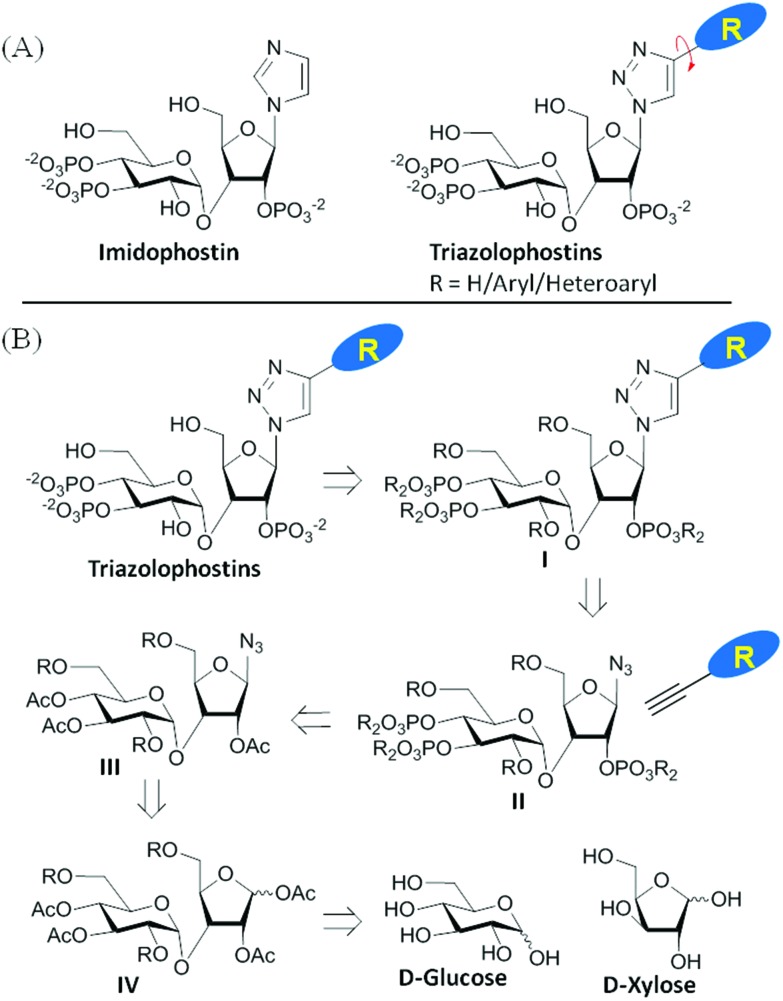
(A) Imidophostin and triazolophostins. (B) Retrosynthetic analysis of triazolophostins.

Many biological processes are regulated by changes in the intracellular concentration of Ca^2+^.^1^ A major pathway for these Ca^2+^ signals is the release of Ca^2+^ from intracellular stores within the endoplasmic reticulum (ER) *via* IP_3_ receptors (IP_3_R).^2^ IP_3_R are a family of intracellular Ca^2+^ channels that are expressed largely in ER membranes.^3^ IP_3_ (Fig. 1A, **1**) is produced when cell-surface receptors stimulate phospholipase C activity. IP_3_ then binds to IP_3_R causing its channel to open and release Ca^2+^ into the cytosol.^4^ The ability of IP_3_ to interact with IP_3_R is terminated by its dephosphorylation or phosphorylation by specific 5-phosphatase and 3-kinase enzymes.^5^ While terminating the Ca^2+^-mobilizing ability of IP_3_, these steps also initiate recycling of inositol to the lipid from which IP_3_ is generated and the synthesis of more complex bioactive phosphoinositols including the pyrophosphates,^6^ many of which have been recent synthetic targets.^7^ Adenophostin A (AdA), a fungal metabolite isolated from the culture broth of *Penicillium brevicompactum*, is a more potent agonist than IP_3_ of IP_3_R.^8^ Moreover, AdA is resistant to the enzymes that metabolize IP_3_. Many analogs of AdA have been synthesized to address structure–activity relationships and the determinants of the increased potency of AdA.^9^ These studies revealed that the three phosphates and 2′′-OH (color coded in Fig. 1A, **2**), which structurally mimic the essential pharmacophore of IP_3_,^10^ are essential for AdA activity. The nucleobase/surrogate with β-stereochemistry is indispensible for the enhanced potency.^9b,11^

Recent studies have suggested that a cation–π interaction between adenine and a cationic residue (Arg504) of the IP_3_R might be responsible for the increased potency of AdA.^9b,12^ Additional base-modified analogs of AdA would be useful in both testing this proposal and as potent and metabolically stable agonists of IP_3_R to probe Ca^2+^ signaling mechanisms.

The major hurdle in developing such AdA analogs lies in the synthesis of these structurally complex base-modified analogs (disaccharide nucleotides).^9e,13^ The traditional synthesis involves N-glycosylation of a nucleobase with an orthogonally protected disaccharide derivative followed by chemoselective demasking and phosphorylation of specific hydroxyl groups in the nucleoside (Fig. 1B). This early introduction of the nucleobase necessitates an almost individually tailored synthesis (branching at an early stage of synthesis) for each base-modified analog. Further complications arise from the lack of a generally applicable method for glycosylation and phosphorylation of such complex molecules. For instance glycosylation of nucleobases is challenging with respect to yield^14^ and regioselectivity,^13b,15^ and each glycosylation condition has to be optimized individually. Chemoselective O-phosphorylation in the presence of nucleobases having nucleophilic groups/sites is also challenging.^16^ Thus synthesis of analogs by a one-at-a-time method is cumbersome, time-consuming and impractical. We herein report a general strategy for making a combinatorial library of structurally diverse AdA analogs that are potent agonists of IP_3_R.

## II. Results and discussion

Imidophostin (Fig. 2A), though weaker than AdA, is more potent than IP_3_, suggesting that even a 5-membered aromatic heterocyclic ring can be a partial functional mimic of adenine. As triazole is isosteric with imidazole, we envisioned that triazole-based AdA analogs would also be potent agonists of IP_3_R. This and the earlier reports on the use of click reaction in biologically important molecules^17^ prompted us to adopt azide–alkyne click chemistry to make a library of triazolophostins, AdA analogs wherein the nucleobase is replaced by substituted triazoles. These molecules can easily be prepared by reacting appropriately protected tris-phosphate derivative **II** having an azide at the anomeric position with aryl or heteroaryl alkynes under Cu(i) catalysis (Fig. 2B). Such an approach would solve three major issues associated with conventional AdA analog synthesis: (i) ensure the necessary β-anomeric stereochemistry of the base-surrogate, (ii) as the triazole-based base-surrogate is introduced only at the penultimate step (after phosphorylation), the interference of nucleobases during phosphorylation can be avoided and (iii) diversification at the penultimate step provides access to many structurally diverse analogs from a common advanced intermediate.

**Scheme 1:**
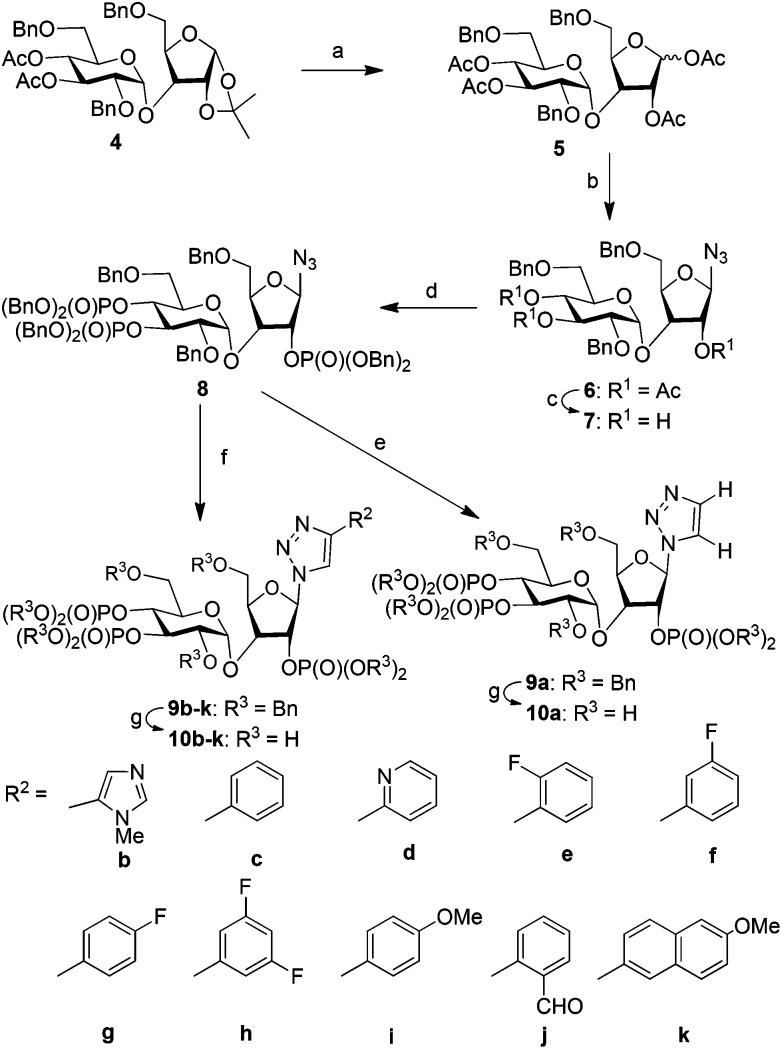
Synthesis of triazolophostins. (a) H_2_SO_4_-silica, Ac_2_O, DCM, rt, 12 h, 90%; (b) TMSN_3_, AlCl_3_, DCM, 0 °C, 10 min, 99%; (c) NaOMe, MeOH, rt, 30 min, 96%; (d) (i) (iPr)_2_NP(OBn)_2_, ImOTf, DCM, rt, 1 h, (ii) *m*-CPBA, –78 °C, 45 min, 90%; (e) (i) trimethylsilylacetylene, Cu, CuSO_4_, H_2_O : ^*t*^BuOH, rt, 12 h; (ii) TBAF, THF, rt, 2 h, (95% for two steps); (f) alkyne, Cu, CuSO_4_, H_2_O : ^*t*^BuOH, rt, 12 h, 48–97%; (g) cyclohexene, Pd(OH)_2_, MeOH : H_2_O, 80 °C, 4 h, Bn = benzyl, Ac = acetyl, Me = methyl.

We have synthesized orthogonally protected disaccharide **4** in thirteen steps from d-glucose and d-xylose in a convergent manner as reported previously.^18^ Acetolysis^19^ of the ketal **4** gave tetraacetate **5** as anomeric mixtures in very good yield. Lewis acid catalysed azidation of the acetate **5** gave anomerically pure β-azide **6**.^20^ Methanolysis of the acetate groups in **6** provided the triol **7** in excellent yield. Phosphitylation of the triol **7** using phosphoramidite followed by *in situ* oxidation using *m*-CPBA provided the fully protected trisphosphate **8**. Compound **8** with three phosphates in their appropriate relative positions and β-oriented azide is the common advanced intermediate for coupling with different alkynes. Copper(i) catalysed click reaction of azide **8** with various alkynes provided fully protected triazolophostins **9a–k**. As the reaction is catalyzed by copper(i), 1,4-substituted triazoles^21^ were obtained exclusively in all the cases. Global removal of the benzyl protecting groups by transfer hydrogenolysis provided triazolophostins **10a–k** in very good yields (Scheme 1). Though in a recent elegant communication, Potter *et al*. reported a functionally active triazole based cADPR analog,^22^ ours is the first report on the use of click chemistry to generate a library of IP_3_R agonists.

**Fig. 3:**
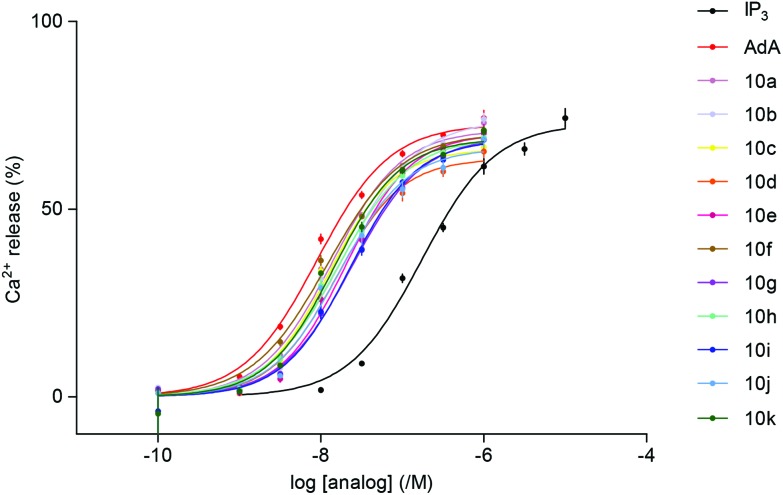
Concentration-dependent release of Ca^2+^ (%) by IP_3_, AdA and triazolophostins from the intracellular stores of cells expressing IP_3_R1. Results show means ± SEM from at least 3 experiments.

The abilities of the newly synthesized ligands to evoke Ca^2+^ release *via* IP_3_R were assessed using permeabilized cells that express only type 1 IP_3_R.^23^ A low-affinity Ca^2+^ indicator trapped within the ER allowed the effects of IP_3_ to be directly measured using a fluorescence plate-reader equipped to allow automated additions. All the triazolophostins fully release the IP_3_-sensitive Ca^2+^ stores and all are more potent than IP_3_ (Table 1, Fig. 3). It is interesting to note that the parent analog triazolophostin **10a** having only the five-membered ring is 13-fold more potent than IP_3_. This remarkable AdA-like potency of **10a** suggests that a single triazole ring can effectively replace the adenine base in AdA without compromising potency. In contrast, imidophostin was previously reported to be only slightly (1.3-fold) more potent than IP_3_, and it was therefore suggested that a fused bicyclic nucleobase/surrogate is essential for AdA-like potency.^18*a*^ Among all triazolophostins, the 3-fluoro derivative **10f** is the most potent, and equipotent to AdA: it is the most potent AdA analog without a purine nucleobase. Of the several AdA analogs reported in the literature, very few match the potency of AdA.^9c,12b,13b,14,18a,24,25^ In this context, our late-stage diversification method is important because it allows many potent analogs to be made in an easy and combinatorial way from the same advanced intermediate.

**Table 1:** Ca^2+^ release *via* IP_3_R evoked by triazolophostins^*a*^

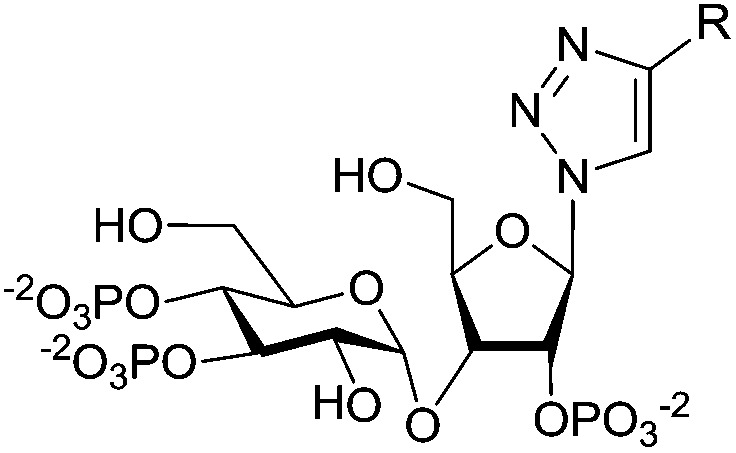					
Ligand	R =	pEC_50_	EC_50_ (nM)	EC_50_ w.r.t. **1**	Max. response (%)
IP_3_ (**1**)		6.75 ± 0.08	178	1	73 ± 3
AdA (**2**)		8.05 ± 0.04	9	20	73 ± 2
**10a**	H	7.87 ± 0.00	13	13	71 ± 1
**10b**	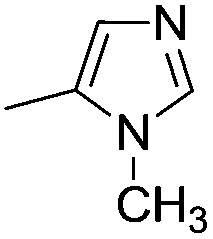	7.75 ± 0.03	18	10	72 ± 2
**10c**	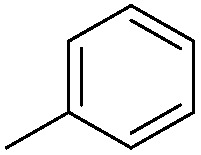	7.81 ± 0.05	15	11	65 ± 3
**10d**	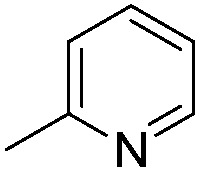	7.77 ± 0.03	17	10	64 ± 1
**10e**	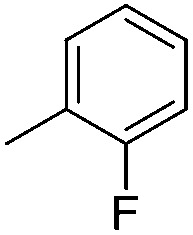	7.70 ± 0.07	20	9	75 ± 4
**10f**	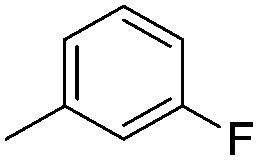	8.00 ± 0.03	10	18	69 ± 1
**10g**	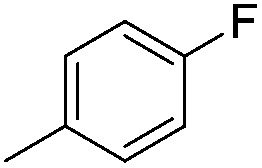	7.86 ± 0.04	14	13	71 ± 2
**10h**	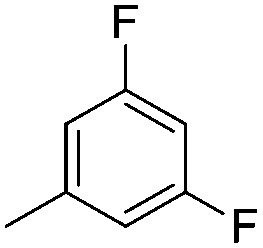	7.64 ± 0.01	23	8	67 ± 2
**10i**	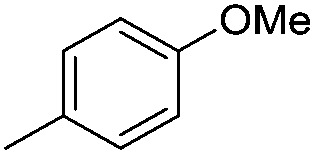	7.45 ± 0.03	35	5	65 ± 1
**10j**	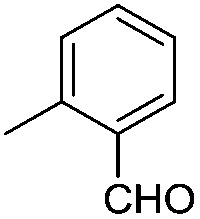	7.78 ± 0.03	17	11	68 ± 2
**10k**	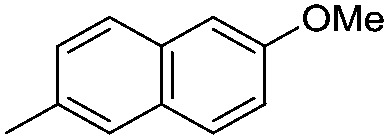	7.89 ± 0.02	13	14	70 ± 1

^*a*^Results show the maximal Ca^2+^ release (% of Ca^2+^ content of intracellular stores), EC_50_ and pEC_50_ values (means ± SEM from at least 3 experiments), and EC_50_ relative to **1** (ECIP350/ECanalog50). Hill coefficients were not significantly different from **1**.

The docking study suggests that triazolophostin **10a** and AdA exhibit similar interactions with the IP_3_-binding core of IP_3_R1 (IBC, residues 224–604).^24^ The triazolyl ring of **10a** forms a cation–π interaction with Arg504 similar to the proposed interaction between the adenine of AdA and Arg504. This supports previous reports that a cation–π interaction between the base and IP_3_R contributes to the increased potency of AdA and analogs.^9c,24^ All other triazolophostin analogs showed a similar interaction. The small differences in activity among these analogs seem to arise from steric, rather than electronic factors. For instance, attaching aromatic rings with different electrostatic surface potentials to the triazole ring did not cause any systematic change in biological activity. The fact that all the analogs are both more potent than IP_3_, and show a conserved cation–π interaction between the triazole ring and Arg504 suggests that this critical interaction is unaffected by aromatic substitution, irrespective of the electronic nature of the aromatic substituent. The single bond connection between the triazole ring and the substituent (aromatic ring) allow torsional freedom (freely rotatable) and hence the sterically favorable non-coplanar conformation of the two aromatic rings. As a result of such non-coplanarity (lack of conjugation), the electron density on the triazole ring cannot be influenced by the aromatic unit. This could be the reason why there is no clear trend in activity when the aromatic ring is modified with electron-deficient or electron-rich substituents.

**Fig. 4:**
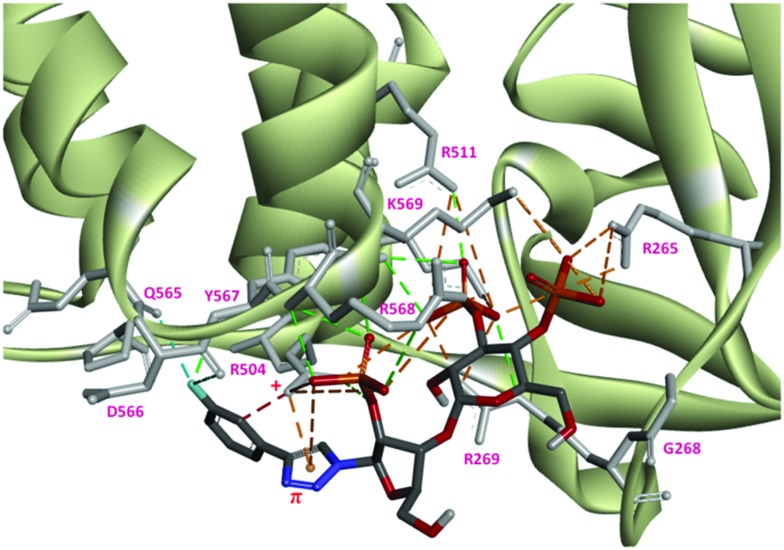
Docking of 3-fluorophenyl triazolophostin with IBC showing preferred binding mode and the important cation–π and fluorine–oxygen interactions with residues Arg504 and Asp566, respectively. The cation–π distance *r* = 2.97 Å. The fluorophenyl moiety orients perpendicular to the triazole ring. Color code for ligands: carbon, gray; oxygen, red; nitrogen, blue; phosphorus, yellow; fluorine, cyan.

One reason for the increased potency of AdA is believed to be its conformational flexibility allowing it to adopt an optimal conformation for binding to IP_3_R.^26^ The additional conformational freedom due to the single bond linkage of this hetero-biaryl unit (rather than fused rings in AdA) allows the second ring to orient to an electronically favorable position, such that it can have additional electrostatic interactions. Docking results suggest that the most potent analog, **10f**, has additional interactions between F and IP_3_R side-chains (D566, Q565 and Q507) that may enhance its activity (Fig. 4).

## III. Conclusions

In conclusion, addressing the problems associated with conventional one-at-a-time synthesis of AdA analogs, we have synthesized a library of new analogs using azide–alkyne click chemistry. The simple reaction conditions, high yield and atom-economy of the click reaction to install base-surrogates are advantageous over the conventional Vorbruggen method of installation of a nucleobase. By this easy and rapid method, we have generated a variety of ligands with diverse structures from a common advanced intermediate. All the analogs synthesized by this method are more potent than IP_3_ and some (**10a**, **10f**, **10g**, **10k**) are as potent as AdA. Though replacement of purines with pyrimidines or other aromatic groups is reported to give agonists that are more potent than IP_3_, none of the previously reported non-purinated AdA analogs was as potent as AdA. We have shown that AdA-like potency can be achieved without a nucleobase. Our study reveals that a five-membered aromatic ring, triazole, is sufficient as a base surrogate to exhibit AdA-like potency. Easy access to potent, non-metabolizable ligands of IP_3_R and the ease of conjugation with reporter groups such as fluorescent probes, photoaffinity probes, *etc.* offers avenues for advanced biological exploration. This report that triazoles can be functional mimics of nucleobases has broader implication and might trigger such attempts in other nucleobase-derived signaling molecules.

## IV. Experimental section

### 1. General methods

Chromatograms were visualized under UV light and by dipping plates into either phosphomolybdic acid in MeOH or anisaldehyde in ethanol, followed by heating. ^1^H NMR, COSY, NOESY and HMQC spectra were recorded on a 500 MHz NMR spectrometer. Proton chemical shifts are reported in ppm (*δ*) relative to the internal standard tetramethylsilane (TMS, *δ* 0.0 ppm) or with the solvent reference relative to TMS employed as the internal standard (CDCl_3_, *δ* 7.26 ppm; D_2_O, *δ* 4.79 ppm). Data are reported as follows: chemical shift (multiplicity [singlet (s), doublet (d), doublet of doublet (dd), triplet (t), quartet (q), and multiplet (m)], coupling constants [Hz], integration and peak identification). All NMR signals were assigned on the basis of ^1^H NMR, ^13^C NMR, COSY and HMQC experiments. ^13^C spectra were recorded with complete proton decoupling. Carbon chemical shifts are reported in ppm (*δ*) relative to TMS with the respective solvent resonance as the internal standard. All NMR data were collected at 25 °C. The concentration of the compounds for ^1^H NMR was 5 mg per 0.5 mL and for ^13^C NMR it was 20 mg per 0.5 mL for protected compounds and 5–7 mg per 0.5 mL for final compounds in the case of ^1^H and ^13^C NMR. Each triazolophostin (**10a–k**) was quantified using the Briggs's/Ames's phosphate assay. Melting point was determined using melting point apparatus and is uncorrected. Flash column chromatography was performed using a silica gel 230–400 mesh. Wherever needed, the reactions were carried out under an argon or nitrogen atmosphere employing oven dried glassware.

X-ray intensity data measurements of freshly grown crystals of **4** were carried out at 293–296 K on a Bruker-KAPPA APEX II CCD diffractometer with graphite-monochromatized (MoKα = 0.71073 Å) radiation. The X-ray generator was operated at 50 kV and 30 mA. Data were collected with a scan width of 0.3° at different settings of *φ* (0°, 90° and 180°) keeping the sample to detector distance fixed at 40 mm and the detector position (2*θ*) fixed at 24°. The X-ray data collection was monitored by SMART program. All the data were corrected for Lorentzian, polarization and absorption effects using SAINT and SADABS programs. SHELX-97 was used for structure solution and full matrix least-squares refinement on *F* ^2^. Molecular and packing diagrams were generated using Mercury-3.1. Geometrical calculations were performed using SHELXTL and PLATON.

### 2. Synthesis of 1,2,3′,4′-tetra-*O*-acetyl-2′,5,6′-tri-*O*-benzyl-3-*O*-α-d-glucopyranosyl-d-ribofuranose (**5**)

To a solution of disaccharide **4** (1.0 g, 1.41 mmol) and Ac_2_O (0.4 mL, 4.24 mmol) in dry DCM (20 mL), 0.05 g of freshly prepared H_2_SO_4_-silica^19^ was added. The reaction mixture was stirred at room temperature for 12 h under a nitrogen atmosphere and the reaction was monitored using TLC. When the starting material disappeared, the reaction mixture was quenched by the addition of solid NaHCO_3_. The solid material was filtered off and the organic layer was washed with saturated aqueous NaHCO_3_ solution. The organic layer was dried over Na_2_SO_4_ and evaporated under reduced pressure. The crude product thus obtained was purified by flash column chromatography using 40% ethyl acetate in petroleum ether as the eluent to obtain the known^18*a*^ tetraacetate **5** (0.96 g, 90%) as a sticky mass.

### 3. Synthesis of 2,3′,4′-tri-*O*-acetyl-2′,5,6′-tri-*O*-benzyl-3-*O*-α-d-glucopyranosyl-β-d-ribofuranosyl azide (**6**)

To a solution of tetraacetate **5** (0.51 g, 0.68 mmol) and TMSN_3_ (0.44 mL, 3.4 mmol) in anhydrous dichloromethane (10 mL) was added AlCl_3_ (0.09 g, 0.68 mmol) at 0 °C. The reaction mixture was stirred at 0 °C under a nitrogen atmosphere for 10 min. When the starting material disappeared (TLC), the reaction mixture was quenched by the addition of aqueous NaHCO_3_. The reaction mixture was partitioned between dichloromethane and aqueous layers in a separating funnel. The dichloromethane layer was washed with brine, dried over Na_2_SO_4_ and evaporated under reduced pressure. The crude product thus obtained was purified by flash column chromatography using 25% ethyl acetate in petroleum ether as the eluent to obtain triacetate azide **6** (0.495 g, 99%) as a colourless gum. ^1^H NMR (500 MHz, CDCl_3_) *δ*: 1.79 (s, 6H, 2 × COC*H*_3_), 1.86 (s, 3H, COCH_3_), 3.22 (dd, *J* = 10.0, 4.0 Hz, 1H, H-6A′), 3.26 (dd, *J* = 10.0, 2.0 Hz, 1H, H-6B′), 3.46 (dd, *J* = 10.0, 3.4 Hz, 1H, H-2′), 3.52 (dd, *J* = 11.0, 4.0 Hz, 1H, H-5A), 3.61 (dd, *J* = 11.0, 3.0 Hz, 1H, H-5B), 3.77 (d, *J* = 9.8, Hz, 1H, H-5′), 4.22 (d, *J* = 12.0, Hz, 1H, 0.5 × PhC*H*_2_), 4.29–4.30 (m, 1H, H-4), 4.38–4.43 (m, 3H, 1.5 × PhC*H*_2_), 4.46–4.54 (m, 3H, H-3 and 1 × PhC*H*_2_), 4.88 (d, *J* = 3.3 Hz, 1H, H-1′), 4.95 (t, *J* = 9.8 Hz, 1H, H-4′), 5.02 (d, *J* = 2.6 Hz, 1H, H-2), 5.27–5.29 (m, 2H, H-1 and H-3′), 7.16–7.22 (m, 15H, 3 × Ph); ^13^C NMR (125 MHz, CDCl_3_) *δ*: 20.5, 20.7, 20.9 (CO*C*H_3_), 67.6 (C-6′), 69.0 (C-4′ and C-5′), 69.5 (C-5), 71.9 (C-3′), 73.3, 73.5, 73.5 (3 × *C*H_2_Ph), 73.9 (C-2), 74.5 (C-3), 76.6 (C-2′), 81.4 (C-4), 92.77 (C-1), 96.7 (C-1′), 127.5, 127.7, 127.7, 127.8, 128.0, 128.4, 128.4, 128.5 (Ar-*C*), 137.5, 137.7, 137.9 (*ipso* Ar–*C*), 169.8, 170.5, 170.5 (3 × *C*OCH_3_); Anal. calcd for C_38_H_43_N_3_O_12_: C, 62.20; H, 5.91; N, 5.73. found: C, 62.12; H, 6.09; N, 5.71.

### 4. Synthesis of 2′,5,6′-tri-*O*-benzyl-3-*O*-α-d-glucopyranosyl-β-d-ribofuranosyl azide (**7**)

To the suspension of triacetate **6** (0.42 g, 0.57 mmol) in MeOH (10 mL) was added sodium methoxide (0.015 g, 0.28 mmol) and stirred at room temperature. The reaction mixture became clear after 30 min. When TLC showed complete disappearance of the starting material, the reaction was quenched by adding Dowex 50-ion exchange resin. The reaction mixture was then filtered through a filter paper and washed successively with methanol (2 times). The filtrate was concentrated under reduced pressure to obtain a thick mass. The thick mass was then purified by flash column chromatography using 50% ethyl acetate in petroleum ether as the eluent to obtain triol azide **7** (0.34 g, 96%) as a white solid. mp: 69–71 °C; ^1^H NMR (500 MHz, CDCl_3_) *δ*: 3.33 (dd, *J* = 9.5, 3.5 Hz, 1H, H-2′), 3.43–3.54 (m, 5H, H-4′, H-5_A_, H-5_B_, H-6_A_′, H-6_B_′), 3.66 (ddd, *J* = 9.8, 8.7, 3.9, 1H, H-5′), 3.82–3.85 (m, 2H, H-3′ and H-2), 4.16–4.18 (m, 2H, 3-H and H-4), 4.38–4.48 (m, 4H, 2 × C*H*_2_Ph), 4.61–4.70 (m, 3H, H-1′ and 1 × C*H*_2_Ph), 5.20 (d, *J* = 1.4 Hz, 1H, H-1), 7.18–7.29 (m, 15H, 3 × Ar-*H*); ^13^C NMR (125 MHz, CDCl_3_) *δ*: 69.2 (C-5 or C-6′), 70.2 (C-5 or C-6′), 70.6 (C-5′), 71.1 (C-4′), 73.3 (C-3′), 73.5 and 73.6 (2 × *C*H_2_Ph), 74.0 (C-2), 74.3 (1 × *C*H_2_Ph), 78.3 (C-3 or C-4), 78.4 (C-2′), 81.4 (C-3 or C-4), 95.1 (C-1), 98.0 (C-1′), 127.6, 127.7, 127.8, 127.9, 128.4, 128.5, 128.6, 128.8 (Ar-*C*), 137.1, 137.6, 137.9 (*ipso* Ar-*C*); Anal. calcd for C_32_H_37_N_3_O_9_: C, 63.25; H, 6.14; N, 6.92; found: C, 63.23; H, 6.16; N, 6.90.

### 5. Synthesis of 2′,5,6′-tri-*O*-benzyl-2,3′,4′-tris(dibenzyloxyphosphoryl)-3-*O*-α-d-glucopyranosyl-β-d-ribofuranosyl azide (**8**)

A solution of the triol **7** (0.1 g, 0.16 mmol), bis(benzyloxy) *N*,*N*-diisopropylaminophosphine (0.27 mL, 0.82 mmol), and imidazolium triflate (0.20 g, 0.90 mmol) in anhydrous DCM (10 mL) was stirred at room temperature under a N_2_ atmosphere for 30 min. The reaction mixture was then cooled to –78 °C, and then *m*-CPBA (0.093 g, 0.54 mmol) was added. The reaction mixture was then stirred for 45 min at –78 °C and then at room temperature for an additional 30 min. The reaction was quenched by the addition of aqueous NaHCO_3_. The reaction mixture was partitioned between DCM and the aqueous layer in a separating funnel. The DCM layer was washed with brine, dried over Na_2_SO_4_ and evaporated under reduced pressure. The crude product thus obtained was purified by flash column chromatography using 25% acetone in petroleum ether as the eluent to obtain pure **8** (0.206 g, 90%) as a colourless gum: ^1^H NMR (500 MHz, CDCl_3_) *δ*: 3.43–3.58 (m, 5H, H-6_A_′, H-6_B_′, H-5_B_, H-5_A_, H-2′), 3.70–3.72 (m, 1H, H-5′), 4.17–4.22 (m, 2H, H-4, and 0.5 × PhC*H*_2_), 4.27–4.34 (m, 3H, H-3 and 1 × PhC*H*_2_), 4.37–4.42 (m, 2H, 1 × PhC*H*_2_), 4.47 (dd, 19.05, 9.75 Hz, 1H, H-4′), 4.55–4.59 (m, 2H, H-2, 0.5 × PhC*H*_2_), 4.65 (dd, 11.95, 8.5 Hz, 1H, 0.5 × PhC*H*_2_), 4.78–4.94 (m, 12H, H-3′ and 5.5 × PhC*H*_2_), 5.01 (d, 3.55 Hz, 1H, H-1′), 5.30 (d, 2.6 Hz, 1H, H-1), 6.96–7.25 (m, 45H, 9 × Ph), ^13^C NMR (125 MHz, CDCl_3_) *δ*: 68.1 (C-6′), 69.1 (C-5), 69.3, 69.3, 69.4, 69.5, 69.8, 69.83, 69.9, (^31^P coupled, Ph*C*H_2_OP), 70.0 (C-5′), 72.0 (1 × Ph*C*H_2_OC), 73.2 (C-3), 73.3, 73.5 (2 × Ph*C*H_2_OC), 74.4 (^31^P coupled, C-4′), 76.9 (C-2′), 77.6 (C-3′, ^31^P coupled), 78.1 (C-2, ^31^P coupled), 81.3 (C-4), 92.4 (C-1), 95.2 (C-1′), 127.6, 127.65, 127.70, 127.8, 128.06, 128.08, 128.26, 128.28, 128.31, 128.34, 128.39, 128.44, 128.49, 128.52, 128.62, 128.64, 128.68, 128.69 (aromatic carbons), 135.40, 135.45, 135.70, 135.77, 135.85, 135.90, 136.14, 136.16, 136.20, 136.23, 137.6, 137.9, 138.1, (^31^P coupled, *ipso* carbons of POCH_2_Ph); ^31^P NMR (202.4 MHz, CDCl_3_) *δ*: –1.20, –1.90, –2.15; Anal. calcd for C_74_H_76_N_3_O_18_P_3_: C, 64.02; H, 5.52; N, 3.03. Found: C, 64.33; H, 5.45; N, 3.19.

### 6. Synthesis of fully protected triazolophostins

#### (a) Synthesis of 1-{5-*O*-benzyl-2-*O*-bisbenzyloxyphosphoryl-3-*O*-[2,6-di-*O*-benzyl-3,4-di-*O*-(bisbenzyloxyphosphoryl)-α-d-glucopyranosyl]-β-d-ribofuranosyl}-1,2,3-triazole (**9a**)

A mixture of tris-phosphate **8** (0.1 g, 0.07 mmol), trimethylsilylacetylene (0.011 mL, 0.071 mmol), Cu (0.018 g, 0.28 mmol) and CuSO_4_ (0.004 g, 0.014 mmol) in H_2_O–^*t*^BuOH (1/1, v/v, 1 mL) was stirred at room temperature for 12 h. After complete consumption of the starting material (TLC, 12 h), TBAF (1.0 M in THF, 0.5 mL) was added to the reaction and the mixture was stirred for additional 2 h at room temperature. The reaction mixture was then filtered and washed with ethyl acetate. The filtrate was extracted with ethyl acetate (2 × 10 mL) and washed with water and brine. The organic layer was dried over MgSO_4_ and concentrated under reduced pressure. The crude product thus obtained was purified by flash column chromatography using a mixture of acetone and petroleum ether (3 : 7 v/v) as the eluent to obtain pure **9a** (0.098 g, 97%) as a colourless gum. ^1^H NMR (500 MHz, CDCl_3_) *δ*: 3.43–3.45 (m, 1H, H-5_A_′), 3.47–3.57 (m, 3H, H-2′′, H-5_B_′, H-6_A_′′), 3.59–3.61 (m, 1H, H-6_B_′′), 3.75 (ddd, *J* = 9.2, 4.65, 4.2 Hz, 1H, H-5′′), 4.21–4.23 (m, 1H, 0.5 × PhC*H*_2_), 4.29–4.32 (m, 4H, H-4′, 1.5 × PhC*H*_2_), 4.39–4.46 (m, 3H, H-3′, H-4′′ and 0.5 × PhC*H*_2_), 4.58–4.66 (m, 3H, 1.5 × PhC*H*_2_), 4.68–4.72 (m, 3H, 1.5 × PhC*H*_2_), 4.80–4.96 (m, 8H, H-3′′, 3.5 × PhC*H*_2_), 5.13 (d, *J* = 3.0 Hz, 1H, H-1′′), 5.27 (ddd, *J* = 8.75, 8.2, 4.6 Hz, 1H, H-2′), 6.31 (d, 4.9 Hz, 1H, H-1′), 6.99–7.27 (m, 45H, 9 × C_6_ *H*_5_), 7.48 (s, 1H, H-5), 7.61 (s, 1H, H-4); ^13^C NMR (125 MHz, CDCl_3_) *δ*: 68.5 (C-6′′), 69.1 (C-5′), 69.2, 69.36, 69.40, 69.5, 69.6, 69.8, 69.83, 69.9, 70.0, 70.02 (^31^P coupled, Ph*C*H_2_OP), 70.3 (C-5′′), 71.9, 73.4, 73.6 (3 × Ph*C*H_2_OC), 73.9 (C-3′), 74.5 (^31^P coupled, C-4′′), 76.9 (C-2′), 78.0 (C-3′′, ^31^P coupled), 78.6 (C-2′, ^31^P coupled), 82.81 (C-4′), 90.0 (C-1′), 95.6 (C-1′′), 122.4 (C-5), 127.6, 127.7, 127.74, 127.77, 127.8, 127.9, 128.0, 128.07, 128.1, 128.2, 128.3, 128.34, 128.4, 128.5, 128.52, 128.55, 128.59, 128.7, (aromatic carbons), 135.2, 135.28, 135.7, 135.74, 135.8, 135.87, 136.1, 136.2, 137.4, 137.6, 138.0 (^31^P coupled, *ipso* carbons of POCH_2_Ph); ^31^P NMR (202.4 MHz, CDCl_3_) *δ*: –1.427, –1.908, –2.128; Anal. calcd for C_76_H_78_N_3_O_18_P_3_: C, 64.54; H, 5.56; N, 2.97. Found: C, 64.31; H, 5.42; N, 3.01.

#### (b) Synthesis of 1-{5-*O*-benzyl-2-*O*-bisbenzyloxyphosphoryl-3-*O*-[2,6-di-*O*-benzyl-3,4-di-*O*-(bisbenzyloxyphosphoryl)-α-d-glucopyranosyl]-β-d-ribofuranosyl}-4-(1-methylimidazol-5-yl)-1,2,3-triazole (**9b**)

A mixture of tris-phosphate **8** (0.2 g, 0.14 mmol), 5-ethynyl-1-methyl-1*H*-imidazole (0.016 mL, 0.15 mmol), Cu (0.036 g, 0.57 mmol) and CuSO_4_ (0.008 g, 0.028 mmol) in H_2_O–^*t*^BuOH (1/1, v/v, 2 mL) was stirred at room temperature for 12 h. After completion of the reaction, the mixture was filtered and washed successively with ethyl acetate. The filtrate was extracted with ethyl acetate and washed with water and brine. The organic layer was dried over MgSO_4_ and concentrated under reduced pressure. The resulting crude product was purified by flash column chromatography using a mixture of acetone, diethyl ether and petroleum ether (4 : 1 : 15 v/v/v) as the eluent to obtain pure **9b** (0.205 g, 95%) as a colourless gum. ^1^H NMR (500 MHz, CDCl_3_) *δ*: 3.55–3.76 (m, 8H, 1 × N-C*H*_3_, 6_A_′′, H-6_B_′′, H-5_B_′, H-2′′ and H-5_A_′), 3.88 (bs, 1H, H-5′′), 4.31–4.45 (m, 5H, H-4′ and 2 × PhC*H*_2_), 4.51–4.54 (m, 3H, H-4′′ and 1 × PhC*H*_2_), 4.70–4.82 (m, 6H, H-3′, and 2.5 × PhC*H*_2_), 4.94–5.03 (m, 8H, H-3′′ and 3.5 × PhC*H*_2_), 5.26 (bs, 1H, H-1′′), 5.41 (bs, 1H, H-2′), 6.42 (s, 1H, H-1′), 6.97–7.34 (m, 47H, Ar-*H*), 7.75 (s, 1H, H-5); ^13^C NMR (125 MHz, CDCl_3_) *δ*: 33.4 (N-*C*H_3_), 68.6 (C-6′′), 69.2 (C-5′), 69.4, 69.42, 69.5, 69.59, 69.8, 69.9, 70.03 (^31^P coupled Ph*C*H_2_OP), 70.3 (C-5′′), 71.92, 73.4, 73.7 (3 × Ph*C*H_2_OC), 74.3 (C-3′), 74.5 (C-4′′, ^31^P coupled), 77.0 (C-2′, ^31^P coupled), 78.0 (C-2′′), 78.8 (C-3′′, ^31^P coupled), 83.1 (C-4′), 90.4 (C-1′, ^31^P coupled), 95.77 (C-1′′), 119.41 (C-5), 127.64, 127.67, 127.71, 127.80, 127.8, 127.96, 128.0, 128.1, 128.14, 128.3, 128.5, 128.52, 128.6, 128.67 (aromatic carbons), 135.1, 135.2, 135.6, 135.76, 135.80, 135.9, 136.1, 136.18, 137.2, 137.6 (^31^P coupled, *ipso* carbons of POCH_2_Ph), 138.0, 138.8 (*ipso* carbons of COCH_2_Ph); ^31^P NMR (202.4 MHz, CDCl_3_) *δ*: –1.453, –1.894, –2.080; Anal. calcd for C_80_H_82_N_5_O_18_P_3_: C, 64.29; H, 5.53; N, 4.69. Found: C, 63.99; H, 5.23; N, 4.81.

#### (c) Synthesis of 1-{5-*O*-benzyl-2-*O*-bisbenzyloxyphosphoryl-3-*O*-[2,6-di-*O*-benzyl-3,4-di-*O*-(bisbenzyloxyphosphoryl)-α-d-glucopyranosyl]-β-d-ribofuranosyl}-4-phenyl-1,2,3-triazole (**9c**)

A mixture of tris-phosphate **8** (0.1 g, 0.07 mmol), phenylacetylene (0.016 mL, 0.158 mmol), Cu (0.018 g, 0.28 mmol) and CuSO_4_ (0.004 g, 0.014 mmol) in H_2_O–^*t*^BuOH (1/1, v/v, 1 mL) was stirred at rt for 12 h. After completion of the reaction (TLC) the mixture was filtered. The filtrate was extracted with ethyl acetate and washed successively with water and brine. The organic layer was dried over MgSO_4_ and concentrated under reduced pressure. The crude product thus obtained was purified by flash column chromatography using a mixture of acetone and petroleum ether (3 : 7 v/v) as the eluent to obtain pure **9c** (0.101 g, 94%) as a colourless gum. ^1^H NMR (500 MHz, CDCl_3_) *δ*: 3.46–3.52 (m, 2H, H-2′′ and H-5_A_′), 3.55–3.58 (m, 2H, H-6_B_′′ and H-5_B_′), 3.64–3.66 (m, 1H, H-6_A_′′), 3.77–3.80 (m, 1H, H-5′′), 4.21–4.35 (m, 5H, H-4′ and 2 × PhC*H*_2_), 4.41–4.46 (m, 3H, H-3′, H-4′′ and 0.5 × PhC*H*_2_), 4.58–4.64 (m, 3H, 1.5 × PhC*H*_2_), 4.68–4.72 (m, 3H, 1.5 × PhC*H*_2_), 4.81–4.94 (m, 8H, H-3′′, and 3.5 × PhC*H*_2_), 5.16 (d, *J* = 3.5 Hz, 1H, H-1′′), 5.33 (ddd, *J* = 9.4, 8.55, 5.0, Hz, 1H, H-2′), 6.33 (d, 5.4 Hz, 1H, H-1′), 6.99–7.47 (m, 50H, 10 × C_6_ *H*_5_), 7.83 (s, 1H, H-5); ^13^C NMR (125 MHz, CDCl_3_) *δ*: 68.7 (C-6′′), 69.2 (C-5′), 69.2, 69.4, 69.45, 69.5, 69.55, 69.6, 69.85, 69.88, 70.0, 70.02, 70.1 (^31^P coupled, Ph*C*H_2_OP), 70.4 (C-5′′), 72.0, 73.5, 73.7 (3 × Ph*C*H_2_OC), 74.5 (C-3′), 74.6 (^31^P coupled, C-4′′), 77.0 (^31^P coupled, C-2′′), 78.1 (C-3′′, ^31^P coupled), 78.8 (C-2′), 83.0 (C-4′), 90.1 (C-1′), 95.9 (C-1′′), 118.2 (C-5), 125.8 (Ar-*C*), 127.6, 127.7, 127.8, 127.86, 128.0, 128.1, 128.2, 128.3, 128.4, 128.45, 128.50, 128.53, 128.6, 128.7 (aromatic carbons), 130.3, 135.2, 135.23, 135.3, 135.7, 135.8, 135.87, 135.93, 136.2, 136.25, 137.4, 137.6, 138.1, 148.1; (^31^P coupled, *ipso* carbons of POCH_2_Ph); ^31^P NMR (202.4 MHz, CDCl_3_) *δ*: –1.551, –1.932, –2.105; Anal. calcd for C_82_H_82_N_3_O_18_P_3_: C, 66.08; H, 5.55; N, 2.82. Found: C, 65.95; H, 5.72; N, 2.97.

#### (d) Synthesis of 1-{5-*O*-benzyl-2-*O*-bisbenzyloxyphosphoryl-3-*O*-[2,6-di-*O*-benzyl-3,4-di-*O*-(bisbenzyloxyphosphoryl)-α-d-glucopyranosyl]-β-d-ribofuranosyl}-4-(2-pyridyl)-1,2,3-triazole (**9d**)

A mixture of tris-phosphate **8** (0.2 g, 0.14 mmol), 2-ethynylpyridine (0.016 mL, 0.15 mmol), Cu (0.036 g, 0.57 mmol) and CuSO_4_ (0.008 g, 0.028 mmol) in H_2_O–^*t*^BuOH (1/1, v/v, 2 mL) was stirred at rt for 12 h. After completion of the reaction (TLC) the mixture was filtered. The filtrate was extracted with ethyl acetate and washed successively with water and brine. The organic layer was dried over MgSO_4_ and concentrated under reduced pressure. The crude product thus obtained was purified by flash column chromatography using a mixture of acetone and petroleum ether (3 : 7 v/v) as the eluent to obtain pure **9d** (0.104 g, 48%) as a colourless gum. ^1^H NMR (500 MHz, CDCl_3_) *δ*: 3.43–3.45 (m, 1H, H-5_A_′), 3.48–3.59 (m, 4H, H-2′′, H-5_B_′, H-6_B_′′and H-6_A_′′), 3.64–3.71–3.76 (m, 1H, H-5′′), 4.20–4.23 (m, 1H, 0.5 × PhC*H*_2_), 4.27–4.37 (m, 4H, H-4′ and 1.5 × PhC*H*_2_), 4.41–4.45 (m, 3H, H-3′, H-4′′ and 0.5 × PhC*H*_2_), 4.60–4.62 (m, 1H, 0.5 × PhC*H*_2_), 4.66–4.72 (m, 5H, 2.5 × PhC*H*_2_), 4.80–4.93 (m, 8H, H-3′′, and 3.5 × PhC*H*_2_), 5.15 (d, *J* = 2.9 Hz, 1H, H-1′′), 5.34–5.35 (m, 1H, H-2′), 6.30 (d, *J* = 5.0 Hz, 1H, H-1′), 6.99–7.19 (m, 45H, 9 × C_6_ *H*_5_), 7.28 (d, *J* = 7.0 Hz, 1H, pyr-H), 7.72 (t, *J* = 7.0 Hz, 1H, pyr-H), 8.03 (d, *J* = 7.0 Hz, 1H, pyr-H), 8.44 (s, 1H, H-5), 8.52 (d, *J* = 4.0 Hz, 1H, pyr-H); ^13^C NMR (125 MHz, CDCl_3_) *δ*: 68.4 (C-6′′), 69.1 (C-5′), 69.13, 69.18, 69.4, 69.42, 69.5, 69.55, 69.8, 69.85, 69.87, 69.9, 70.0, 70.04 (^31^P coupled, Ph*C*H_2_OP), 70.3 (C-5′′), 72.0, 73.38, 73.6 (3 × Ph*C*H_2_OC), 74.2 (C-3′), 74.5 (^31^P coupled, C-4′′), 77.1 (^31^P coupled, C-2′′), 78.1 (C-3′′, ^31^P coupled), 78.7 (C-2′), 83.1 (C-4′), 90.4 (C-1′), 95.7 (C-1′′), 120.6 (C-5), 123.0 (Ar-*C*), 127.6, 127.7, 127.8, 127.9, 128.0, 128.1, 128.2, 128.3, 128.4, 128.43, 128.49, 128.5, 128.55 (aromatic carbons), 135.2, 135.25, 135.28, 135.30, 136.19, 136.25, 137.37, 137.60, 138.11; (^31^P coupled, *ipso* carbons of POCH_2_Ph); ^31^P NMR (202.4 MHz, CDCl_3_) *δ*: –1.60, –1.95, –2.13; Anal. calcd for C_81_H_81_N_4_O_18_P_3_: C, 65.23; H, 5.47; N, 3.76. Found: C, 65.01; H, 5.21; N, 3.92.

#### (e) Synthesis of 1-{5-*O*-benzyl-2-*O*-bisbenzyloxyphosphoryl-3-*O*-[2,6-di-*O*-benzyl-3,4-di-*O*-(bisbenzyloxyphosphoryl)-α-d-glucopyranosyl]-β-d-ribofuranosyl}-4-(2-fluorophenyl)-1,2,3-triazole (**9e**)

A mixture of tris-phosphate **8** (0.2 g, 0.14 mmol), 2-fluorophenyl acetylene (0.021 mL, 0.15 mmol), Cu (0.036 g, 0.57 mmol) and CuSO_4_ (0.008 g, 0.028 mmol) in H_2_O–^*t*^BuOH (1/1, v/v, 2 mL) was stirred at rt for 12 h. After completion of the reaction (TLC) the mixture was filtered. The filtrate was extracted with ethyl acetate and washed successively with water and brine. The organic layer was dried over MgSO_4_ and concentrated under reduced pressure. The crude product thus obtained was purified by flash column chromatography using a mixture of acetone and petroleum ether (1 : 3 v/v) as the eluent to obtain **9e** (0.203 g, 93%) as a colourless gum. ^1^H NMR (500 MHz, CDCl_3_) *δ*: 3.53–3.55 (m, 1H, H-5_A_′), 3.61–3.73 (m, 4H, H-2′′, H-5_B_′, H-6_A_′′ and H-6_B_′′), 3.88 (bs, 1H, H-5′′), 4.33–4.35 (m, 1H, 0.5 × PhC*H*_2_), 4.42–4.48 (m, 4H, H-4′ and 1.5 × PhC*H*_2_), 4.53–4.58 (m, 3H, H-3′, H-4′′ and 0.5 × PhC*H*_2_), 4.72–4.81 (m, 6H, 3 × PhC*H*_2_), 4.96–5.06 (m, 8H, H-3′′, 3.5 × PhC*H*_2_), 5.27 (bs, 1H, H-1′′), 5.46 (bs, 1H, H-2′), 6.42 (bs, 1H, H-1′), 7.13–7.40 (m, 48H, Ar-*H*), 8.21 (s, 1H, H-5), 8.30 (bs, 1H, Ar-*H*); ^13^C NMR (125 MHz, CDCl_3_) *δ*: 68.4 (C-6′′), 69.0 (C-5′), 69.1, 69.2, 69.4, 69.42, 69.5, 69.6, 69.8, 69.85, 69.89, 70.0, 70.05 (^31^P coupled, Ph*C*H_2_OP), 70.2 (C-5′′), 71.9, 73.4, 73.6 (3 × Ph*C*H_2_OC), 74.2 (C-3′), 74.4 (^31^P coupled, C-4′′), 77.1 (^31^P coupled, C-2′′), 78.0 (C-3′′, ^31^P coupled), 78.6 (C-2′), 83.0 (C-4′), 90.2 (C-1′), 95.7 (C-1′′), 115.7 (C-5), 121.6, 121.7, 124.5, 124.6, 127.6, 127.9, 128.0, 128.1, 128.5, 128.6 (aromatic carbons), 129.4, 129.5, 135.2, 135.22, 135.3, 135.7, 135.8, 135.84, 135.89, 136.2, 136.22, 137.3, 137.6, 138.1, 141.48, 141.5, 158.3, 160.3 (^31^P coupled, *ipso* carbons of POCH_2_Ph); ^31^P NMR (202.4 MHz, CDCl_3_) *δ*: –1.632, –1.930, –2.134; ^19^F NMR (470.68 MHz, CDCl_3_) *δ*: –114.22; Anal. calcd for C_82_H_81_FN_3_O_18_P_3_: C, 65.29; H, 5.41; N, 2.79. Found: C, 65.43; H, 5.59; N, 2.67.

#### (f) Synthesis of 1-{5-*O*-benzyl-2-*O*-bisbenzyloxyphosphoryl-3-*O*-[2,6-di-*O*-benzyl-3,4-di-*O*-(bisbenzyloxyphosphoryl)-α-d-glucopyranosyl]-β-d-ribofuranosyl}-4-(3-fluorophenyl)-1,2,3-triazole (**9f**)

A mixture of azide **8** (0.2 g, 0.14 mmol), 3-fluorophenyl acetylene (0.021 mL, 0.15 mmol), Cu (0.036 g, 0.57 mmol) and CuSO_4_ (0.008 g, 0.028 mmol) in H_2_O–^*t*^BuOH (1/1, v/v, 2 mL) was stirred at rt for 12 h. After completion of the reaction (TLC), the mixture was filtered, the filtrate was extracted with ethyl acetate and washed successively with water and brine. The organic layer was dried over MgSO_4_ and concentrated under reduced pressure. The crude product thus obtained was purified by flash column chromatography using a mixture of acetone and petroleum ether (1 : 3 v/v) as the eluent to obtain **9f** (0.188 g, 86%) as a colourless gum; ^1^H NMR (500 MHz, CDCl_3_) *δ*: 3.58 (dd, *J* = 10.85, 2.45 Hz, 1H, H-5_A_′), 3.62 (dd, *J* = 9.65, 3.65 Hz, 1H, H-2′′), 3.66–3.69 (m, 2H, H-6_A_′′, H-5_B_′), 3.78 (dd, *J* = 10.8, 1.5 Hz, 1H, H-6_B_′′) 3.90 (ddd, *J* = 10.8, 9.2, 4.2 Hz, 1H, H-5′′), 4.33–4.38 (m, 2H, 1 × PhC*H*_2_), 4.41–4.44 (m, 2H, 1 × PhC*H*_2_), 4.46–4.47 (m, 1H, H-4′), 4.52–4.56 (m, 3H, H-3′, H-4′′ and 0.5 × PhC*H*_2_), 4.70–4.75 (m, 3H, 1.5 × PhC*H*_2_), 4.80–4.84 (m, 3H, 1.5 × PhC*H*_2_), 4.92–5.06 (m, 8H, H-3′′, 3.5 × PhC*H*_2_), 5.28 (d, *J* = 3.6 Hz, 1H, H-1′′), 5.43 (ddd, *J* = 8.85, 5.2, 4.65 Hz, 1H, H-2′), 6.44 (d, 5.6 Hz, 1H, H-1′), 7.09–7.39 (m, 49H, Ar-*H*), 7.90 (s, 1H, H-5); ^13^C NMR (125 MHz, CDCl_3_) *δ*: 68.7 (C-6′′), 69.1 (C-5′), 69.4, 69.5, 69.8, 69.6, 69.7, 69.87, 69.9, 69.94, 70.07, 70.1 (^31^P coupled, Ph*C*H_2_OP), 72.0 (C-5′′), 72.1, 73.5, 73.7 (3 × Ph*C*H_2_OC), 74.5 (C-3′), 74.53 (^31^P coupled, C-4′′), 77.1 (^31^P coupled, C-2′′), 78.1 (C-3′′, ^31^P coupled), 78.9 (C-2′), 83.1 (C-4′), 90.3 (C-1′), 95.9 (C-1′′), 112.7, 114.8 (Ar-*C*), 118.5 (C-5), 121.4 (Ar-*C*), 127.6, 127.7, 127.8, 127.83, 127.9, 128.0, 128.07, 128.14, 128.31, 128.34, 128.43, 128.48, 128.5, 128.51, 128.69 (aromatic carbons), 130.2, 130.27, 136.2, 136.23, 137.31, 137.61, 138.1, 148.1, 162.3, 164.2 (^31^P coupled, *ipso* carbons of POCH_2_Ph); ^31^P NMR (202.4 MHz, CDCl_3_) *δ*: –1.515, –1.920, –2.064; ^19^F NMR (470.68 MHz, CDCl_3_) *δ*: –112.766; Anal. calcd for C_82_H_81_FN_3_O_18_P_3_: C, 65.29; H, 5.41; N, 2.79. Found: C, 65.50; H, 5.21; N, 2.66.

#### (g) Synthesis of 1-{5-*O*-benzyl-2-*O*-bisbenzyloxyphosphoryl-3-*O*-[2,6-di-*O*-benzyl-3,4-di-*O*-(bisbenzyloxyphosphoryl)-α-d-glucopyranosyl]-β-d-ribofuranosyl}-4-(4-fluorophenyl)-1,2,3-triazole (**9g**)

A mixture of azide **8** (0.2 g, 0.14 mmol), 4-fluorophenyl acetylene (0.018 mL, 0.15 mmol), Cu (0.036 g, 0.57 mmol) and CuSO_4_ (0.008 g, 0.028 mmol) in H_2_O–^*t*^BuOH (1/1, v/v, 2 mL) was stirred at rt for 12 h. After completion of the reaction (TLC), the mixture was filtered, the filtrate was extracted with ethyl acetate and washed successively with water and brine. The organic layer was dried over MgSO_4_ and concentrated under reduced pressure. The resulting crude product thus obtained was purified by flash column chromatography using a mixture of 25% acetone in petroleum ether as the eluent to obtain pure **9g** (0.207 g, 95%) as a colourless gum; ^1^H NMR (500 MHz, CDCl_3_) *δ*: 3.47–3.52 (m, 2H, H-2′′ and H-5_A_′), 3.55–3.59 (m, 2H, H-6_A_′′, H-5_B_′), 3.65–3.67 (m, 1H, H-6_B_′′), 3.77–3.80 (m, 1H, H-5′′), 4.21–4.28 (m, 2H, 1 × PhC*H*_2_), 4.30–4.35 (m, 3H, H-4′), 4.39–4.45 (m, 3H, H-4′′ and 1 × PhC*H*_2_), 4.61–4.65 (m, 3H, H-3′, and 1 × PhC*H*_2_), 4.67–4.73 (m, 3H, 1.5 × PhC*H*_2_), 4.81–4.97 (m, 8H, H-3′′, 3.5 × PhC*H*_2_), 5.16 (d, *J* = 3.6 Hz, 1H, H-1′′), 5.32 (ddd, *J* = 9.2, 8.5, 5.0 Hz, 1H, H-2′), 6.34 (d, 5.6 Hz, 1H, H-1′), 6.94–7.37 (m, 49H, Ar-*H*), 7.79 (s, 1H, H-5); ^13^C NMR (125 MHz, CDCl_3_) *δ*: 68.6 (C-6′′), 69.2 (C-5′), 69.22, 69.4, 69.45, 69.5, 69.56, 69.6, 69.8, 69.9, 69.91, 70.03, 70.07 (^31^P coupled, Ph*C*H_2_OP), 70.3 (C-5′′), 72.0, 73.5, 73.7 (3 × Ph*C*H_2_OC), 74.4 (C-3′), 74.5 (^31^P coupled, C-4′′), 77.3 (^31^P coupled, C-2′′), 78.0 (C-3′′, ^31^P coupled), 78.9 (C-2′), 83.0 (C-4′), 90.2 (C-1′), 95.9 (C-1′′), 115.6 (C-5), 117.9, 127.5, 127.51, 127.62, 127.67, 127.7, 127.8, 127.87, 128.0, 128.01, 128.07, 128.1, 128.16, 128.20, 128.35, 128.39, 128.47, 128.5, 128.55, 128.65, 128.67 (aromatic carbons), 136.14, 136.20, 137.4, 137.6, 138.0, 147.5, 162.1, 164.4 (^31^P coupled, *ipso* carbons of POCH_2_Ph); ^31^P NMR (202.4 MHz, CDCl_3_) *δ*: –1.49, –1.920, –2.092; ^19^F NMR (470.68 MHz, CDCl_3_) *δ*: –113.585; Anal. calcd for C_82_H_81_FN_3_O_18_P_3_: C, 65.29; H, 5.41; N, 2.79. Found: C, 64.98; H, 5.11; N, 2.93.

#### (h) Synthesis of 1-{5-*O*-benzyl-2-*O*-bisbenzyloxyphosphoryl-3-*O*-[2,6-di-*O*-benzyl-3,4-di-*O*-(bisbenzyloxyphosphoryl)-α-d-glucopyranosyl]-β-d-ribofuranosyl}-4-(3,5-difluorophenyl)-1,2,3-triazole (**9h**)

A mixture of azide **8** (0.2 g, 0.14 mmol), 3,5-difluorophenyl acetylene (0.018 mL, 0.15 mmol), Cu (0.036 g, 0.57 mmol) and CuSO_4_ (0.008 g, 0.028 mmol) in H_2_O–^*t*^BuOH (1/1, v/v, 2 mL) was stirred at rt for 12 h. After completion of the reaction (TLC), the mixture was filtered. The filtrate was extracted with ethyl acetate and washed successively with water and brine. The organic layer was dried over MgSO_4_ and concentrated under reduced pressure. The crude product thus obtained was purified by flash column chromatography using a mixture of 25% acetone in petroleum ether as the eluent to obtain pure **9h** (0.2 g, 91%) as a colourless gum. ^1^H NMR (500 MHz, CDCl_3_) *δ*: 3.46 (dd, *J* = 10.8, 2.1 Hz, 1H, H-5_A_′), 3.51 (dd, *J* = 9.65, 3.6 Hz, 1H, H-2′′), 3.54–3.58 (m, 2H, H-6_A_′′ and H-5_B_′), 3.68 (dd, *J* = 10.7, 1.3 Hz, 1H, H-6_B_′′), 3.79 (ddd, *J* = 5.95, 4.85, 3.95 Hz, 1H, H-5′′), 4.24 (dd, *J* = 11.5, 7.5 Hz, 2H, 1 × PhC*H*_2_), 4.29–4.34 (m, 2H, 1 × PhC*H*_2_), 4.36 (d, *J* = 2.3 Hz, 1H, H-4′), 4.38–4.45 (m, 3H, H-3′, H-4′′ and 0.5 × PhC*H*_2_), 4.59–4.62 (m, 3H, 1.5 × PhC*H*_2_), 4.68–4.72 (m, 3H, 1.5 × PhC*H*_2_), 4.81–4.94 (m, 8H, H-3′′, 3.5 × PhC*H*_2_), 5.18 (d, *J* = 3.6 Hz, 1H, H-1′′), 5.31 (ddd, *J* = 8.85, 5.2, 4.65 Hz, 1H, H-2′), 6.33 (d, *J* = 5.75 Hz, 1H, H-1′), 6.97–7.29 (m, 48H, Ar-*H*), 7.77 (s, 1H, H-5); ^13^C NMR (125 MHz, CDCl_3_) *δ*: 68.7 (C-6′′), 69.2 (C-5′), 69.23, 69.4, 69.45, 69.5, 69.57, 69.6, 69.8, 69.85, 69.9, 70.02, 70.07 (^31^P coupled, Ph*C*H_2_OP), 70.4 (C-5′′), 72.0, 73.48, 73.7 (3 × Ph*C*H_2_OC), 74.5 (C-3′), 74.54 (^31^P coupled, C-4′′), 77.1 (^31^P coupled, C-2′′), 78.0 (C-3′′, ^31^P coupled), 78.9 (C-2′), 83.3 (C-4′), 90.4 (C-1′), 95.8 (C-1′′), 108.7 (C-5), 118.9,127.6, 127.7, 127.73, 127.85, 127.92, 128.0, 128.1, 128.14, 128.3, 128.35, 128.39, 128.5, 128.51, 128.54, 128.58, 128.66, 128.79 (aromatic carbons), 135.2, 135.23, 135.25, 135.7, 135.71, 135.8, 135.89, 136.1, 136.2, 137.2, 137.23, 137.6, 146.45, 162.5, 163.3 (^31^P coupled, *ipso* carbons of POCH_2_Ph); ^31^P NMR (202.4 MHz, CDCl_3_) *δ*: –1.502, –1.902, –2.068; ^19^F NMR (470.68 MHz, CDCl_3_) *δ*: –109.331; Anal. calcd for C_82_H_80_F_2_N_3_O_18_P_3_: C, 64.52; H, 5.28; N, 2.75. Found: C, 64.37; H, 5.58; N, 2.91.

#### (i) Synthesis of 1-{5-*O*-benzyl-2-*O*-bisbenzyloxyphosphoryl-3-*O*-[2,6-di-*O*-benzyl-3,4-di-*O*-(bisbenzyloxyphosphoryl)-α-d-glucopyranosyl]-β-d-ribofuranosyl}-4-(4-methoxyphenyl)-1,2,3-triazole (**9i**)

A mixture of azide **8** (0.2 g, 0.14 mmol), 4-methoxyphenyl acetylene (0.02 mL, 0.15 mmol), Cu (0.036 g, 0.57 mmol) and CuSO_4_ (0.008 g, 0.028 mmol) in H_2_O–^*t*^BuOH (1/1, v/v, 2 mL) was stirred at rt for 12 h. After completion of the reaction (TLC) the mixture was filtered. The filtrate was extracted with ethyl acetate and washed successively with water and brine. The organic layer was dried over MgSO_4_ and concentrated under reduced pressure. The crude product thus obtained was purified by flash column chromatography using a mixture of acetone and petroleum ether (1 : 3 v/v) as the eluent to obtain **9i** (0.193 g, 88%) as a colourless gum; ^1^H NMR (500 MHz, CDCl_3_) *δ*: 3.49–3.56 (m, 5H, 6_A_′′, H-6_B_′′, H-5_B_′, H-2′′ and H-5_A_′), 3.77 (bs, 4H, 1 × OC*H*_3_ and H-5′′), 4.21–4.35 (m, 5H, H-4′ and 2 × PhC*H*_2_), 4.40–4.45 (m, 3H, H-3′, H-4′′ and 0.5 × PhC*H*_2_), 4.58–4.72 (m, 6H, and 3 × PhC*H*_2_), 4.84–4.93 (m, 8H, H-3′′ and 3.5 × PhC*H*_2_), 5.16 (bs, 1H, H-1′′), 5.32 (bs, 1H, H-2′), 6.33 (d, *J* = 3.2 Hz, 1H, H-1′), 6.80 (d, *J* = 7.5 Hz, 2H, Ar-*H*), 7.00–7.28 (m, 45H, 9 × C_6_ *H*_5_), 6.38 (d, *J* = 7.5 Hz, 2H, Ar-*H*), 7.75 (s, 1H, H-5); ^13^C NMR (125 MHz, CDCl_3_) *δ*: 55.4 (O*C*H_3_), 68.6 (C-6′′), 69.16 (C-5′), 69.2, 69.4, 69.44, 69.5, 69.58, 69.85, 69.9, 70.07, (^31^P coupled Ph*C*H_2_OP), 70.3 (C-5′′), 72.0, 73.4, 73.6 (3 × Ph*C*H_2_OC), 74.4 (C-3′ and C-4′′, ^31^P coupled), 77.0 (C-2′, ^31^P coupled), 78.0 (C-2′′), 78.6 (C-3′′, ^31^P coupled), 83.0 (C-4′), 90.3 (C-1′, ^31^P coupled), 95.9 (C-1′′), 114.2, 117.5 (C-5), 127.1, 127.2, 127.6, 127.7, 127.8, 127.82, 127.85, 128.0, 128.03, 128.07, 128.1, 128.2, 128.3, 128.4, 128.45, 128.5, 128.52, 128.6, 128.64 (aromatic carbons), 135.1, 135.15, 135.2, 135.65, 135.70, 135.8, 135.84, 136.1, 136.16, 137.4, 137.6, (^31^P coupled, *ipso* carbons of POCH_2_ *Ph*), 138.0 (*ipso* carbons of COCH_2_ *Ph*); ^31^P NMR (202.4 MHz, CDCl_3_) *δ*: –1.576, –1.941, –2.118; Anal. calcd for C_83_H_84_N_3_O_19_P_3_: C, 65.56; H, 5.57; N, 2.76. Found: C, 65.66; H, 5.77; N, 2.95.

#### (j) Synthesis of 1-{5-*O*-benzyl-2-*O*-bisbenzyloxyphosphoryl-3-*O*-[2,6-di-*O*-benzyl-3,4-di-*O*-(bisbenzyloxyphosphoryl)-α-d-glucopyranosyl]-β-d-ribofuranosyl}-4-(2-formylphenyl)-1,2,3-triazole (**9j**)

A mixture of azide **8** (0.2 g, 0.14 mmol), 2-ethynylbenzaldehyde (0.02 g, 0.15 mmol), Cu (0.036 g, 0.57 mmol) and CuSO_4_ (0.008 g, 0.028 mmol) in H_2_O–^*t*^BuOH (1/1, v/v, 2 mL) was stirred at rt for 12 h. After completion of the reaction (TLC) the mixture was filtered. The filtrate was extracted with ethyl acetate and washed successively with water and brine. The organic layer was dried over MgSO_4_ and concentrated under reduced pressure. The crude product thus obtained was purified by flash column chromatography using a mixture of acetone and petroleum ether (1 : 3 v/v) as the eluent to obtain pure **9j** (0.19 g, 87%) as a colourless gum;. ^1^H NMR (500 MHz, CDCl_3_) *δ*: 3.57–3.64 (m, 2H, H-2′′ and H-5_A_′), 3.68–3.70 (m, 2H, H-5_B_′ and H-6_A_′′), 3.76–3.78 (m, 1H, H-6_B_′′), 3.90 (bs, 1H, H-5′′), 4.33–4.44 (m, 4H, 2 × PhC*H*_2_), 4.49–4.58 (m, 4H, H-4′, H-4′′ and 1 × PhC*H*_2_), 4.72–4.86 (m, 6H, H-3′ and 2.5 × PhC*H*_2_), 4.93–5.07 (m, 8H, H-3′′, 3.5 × PhC*H*_2_), 5.29 (bs, 1H, H-1′′), 5.47 (bs, 1H, H-2′), 6.48 (d, 5.0 Hz, 1H, H-1′), 7.13–7.41 (m, 46H, Ar-*H*), 7.51–7.59 (m, 2H, Ar-*H*), 8.00 (s, 1H, H-5), 8.05 (d, *J* = 7.4 Hz, 1H, Ar-*H*), 10.35 (s, 1H, C*H*O); ^13^C NMR (125 MHz, CDCl_3_) *δ*: 68.6 (C-6′′), 68.66 (C-5′), 69.2, 69.22, 69.3, 69.4, 69.45, 69.6, 69.61, 69.87, 69.9, 69.94, 70.07, 70.1 (^31^P coupled, Ph*C*H_2_OP), 70.4 (C-5′′), 72.0, 73.5, 73.7 (3 × Ph*C*H_2_OC), 74.3 (C-3′), 74.5 (^31^P coupled, C-4′′), 77.3 (^31^P coupled, C-2′′), 78.0 (C-3′′, ^31^P coupled), 78.9 (C-2′), 83.2 (C-4′), 90.5 (C-1′), 95.8 (C-1′′), 121.7 (C-5), 127.7, 127.71, 127.73, 127.8, 127.83, 127.86, 128.0, 128.1, 128.14, 128.19, 128.35, 128.38, 128.5, 128.54, 128.59, 128.69 (aromatic carbons), 129.9, 132.8, 134.1, 136.2, 137.2, 137.6, 138.1, 145.6 (^31^P coupled, *ipso* carbons of POCH_2_Ph), 192.21 (*C*HO); ^31^P NMR (202.4 MHz, CDCl_3_) *δ*: –1.32, –1.89, –2.07; Anal. calcd for C_83_H_82_N_3_O_19_P_3_: C, 65.65; H, 5.44; N, 2.77. Found: C, 65.41; H, 5.73; N, 2.90.

#### (k) Synthesis of 1-{5-*O*-benzyl-2-*O*-bisbenzyloxyphosphoryl-3-*O*-[2,6-di-*O*-benzyl-3,4-di-*O*-(bisbenzyloxyphosphoryl)-α-d-glucopyranosyl]-β-d-ribofuranosyl}-4-(6-methoxynaphth-2-yl)-1,2,3-triazole (**9k**)

A mixture of azide **8** (0.1 g, 0.07 mmol), 2-ethynyl-6-methoxynaphthalene (0.014 g, 0.077 mmol), Cu (0.018 g, 0.28 mmol) and CuSO_4_ (0.004 g, 0.014 mmol) in H_2_O–^*t*^BuOH (1/1, v/v, 1 mL) was stirred at rt for 12 h. After completion of the reaction (TLC) the mixture was filtered. The filtrate was extracted with ethyl acetate and washed successively with water and brine. The organic layer was dried over MgSO_4_ and concentrated under reduced pressure. The crude product thus obtained was purified by flash column chromatography using 25% acetone in petroleum ether as the eluent to obtain pure, protected triazolophostin **9k** (93 mg, 82%) as a colourless gum; ^1^H NMR (500 MHz, CDCl_3_) *δ*: 3.47–3.52 (m, 2H, H-2′′ and H-5_A_′), 3.55–3.59 (m, 2H, H-5_B_′ and H-6_A_′′), 3.64–3.66 (m, 1H, H-6_B_′′), 3.77–3.80 (m, 1H, H-5′′), 3.85 (s, 3H, OC*H*_3_), 4.21–4.24 (m, 1H, 0.5 × PhC*H*_2_), 4.28–4.36 (m, 4H, H-4′ and 1.5 × PhC*H*_2_), 4.41–4.48 (m, 3H, H-3′, H-4′′ and 1 × PhC*H*_2_), 4.59–4.65 (m, 3H, 1.5 × PhC*H*_2_), 4.69–4.72 (m, 3H, 1.5 × PhC*H*_2_), 4.80–4.97 (m, 8H, H-3′′, 3.5 × PhC*H*_2_), 5.18 (d, *J* = 3.3 Hz, 1H, H-1′′), 5.37 (ddd, *J* = 9.1, 8.5, 4.9 Hz, 1H, H-2′), 6.35 (d, 5.3 Hz, 1H, H-1′), 7.00–7.19 (m, 45H, 9 × C_6_ *H*_5_), 7.29 (d, *J* = 7.5 Hz, 2H, Ar-*H*), 7.48 (d, *J* = 7.5 Hz, 1H, Ar-*H*), 7.63 (t, *J* = 7.5 Hz, 2H, Ar-*H*), 7.89 (s, 1H, Ar-*H*), 8.05 (s, 1H, H-5); ^13^C NMR (125 MHz, CDCl_3_) *δ*: 55.4 (O*C*H_3_), 68.7 (C-6′′), 69.2 (C-5′), 69.4, 69.6, 69.8, 69.9, 70.1 (^31^P coupled, Ph*C*H_2_OP), 70.3 (C-5′′), 72.0, 73.5, 73.6 (3 × Ph*C*H_2_OC), 74.5 (C-3′), 74.54 (^31^P coupled, C-4′′), 77.0 (^31^P coupled, C-2′′), 78.1 (C-3′′, ^31^P coupled), 78.8 (C-2′), 83.1 (C-4′), 90.1 (C-1′), 95.9 (C-1′′), 105.9, 119.20 (C-5), 124.5, 127.2, 127.6, 127.63, 127.7, 127.8, 127.86, 127.89, 127.97, 128.0, 128.1, 128.13, 128.2, 128.3, 128.36, 128.38, 128.45, 128.5, 128.52, 128.57, 128.6, 128.7, 129.0 (aromatic carbons), 129.8, 134.4, 136.2, 137.2, 137.5, 137.6, 138.1 (^31^P coupled, *ipso* carbons of POCH_2_Ph); ^31^P NMR (202.4 MHz, CDCl_3_) *δ*: –1.524, –1.920, –2.082; Anal. calcd for C_87_H_86_N_3_O_19_P_3_: C, 66.53; H, 5.52; N, 2.68. Found: C, 66.74; H, 5.39; N, 2.88.

### 7. General procedure for the synthesis of triazolophostins

To a solution of protected triazolophostins **9a–k** (80–200 mg, 0.06–0.13 mmol) and cyclohexene (2 mL) in a mixture of methanol and water (9 : 1 v/v, 5 mL), Pd(OH)_2_ (20% on carbon, 50 mg) was added and the mixture was stirred at 80 °C for 4 h. The reaction mixture was then cooled, filtered through a membrane filter by washing successively with methanol and water. The combined filtrate was evaporated under reduced pressure. The crude product thus obtained was purified by ion-exchange column chromatography on a Q-Sepharose matrix using 0→1.0 M triethylammonium bicarbonate (TEAB) as the eluent to obtain pure triazolophostins **10a–k**.

#### (a) 3′-*O*-α-d-glucopyranosyl-1-β-d-ribofuranosyl-1,2,3-triazole 2′,3′′,4′′-trisphosphate [triazolophostin] (**10a**)

By using 80 mg (0.056 mmol) of **9a**, 33 mg (97%) of triazolophostin **10a** was obtained as a white hygroscopic solid: ^1^H NMR (500 MHz, D_2_O) *δ*: 3.74–3.80 (m, 7H, H-4′′, H-5′′, H-5_A_′, H-5_B_′, H-6_A_′′, H-6_B_′′ and H-2′′), 4.03 (bs, 1H, H-4′), 4.41 (bs, 2H, H-3′ and H-3′′), 5.14 (bs, 1H, H-2′), 5.23 (bs, 1H, H-1′′), 6.38 (bs, 1H, H-1′), 7.80 (s, 1H, H-5), 8.18 (s, 1H, H-4); ^13^C NMR (125 MHz, D_2_O) *δ*: 60.3 (C-5′), 60.8 (C-6′′), 70.9 (C-2′′, ^31^P coupled), 71.8 (C-5′′, ^31^P coupled), 72.6 (C-4′′, ^31^P coupled), 73.6 (C-3′, ^31^P coupled), 76.3 (C-2′, ^31^P coupled), 77.3 (C-3′′, ^31^P coupled), 83.8 (C-4′), 90.8 (C-1′, ^31^P coupled), 98.0 (C-1′′), 125.1 (C-4, C-5); ^31^P NMR (202.4 MHz, CDCl_3_) *δ*: 3.496, 3.570, 4.382; HRMS (ESI) mass calcd for C_13_H_24_N_3_O_18_P_3_ [M]^+^, 603.0268, found: 603.0271.

#### (b) 4-(1-Methyl-imidazol-5-yl)-triazolophostin (**10b**)

By using 105 mg (0.07 mmol) of **9b**, 45 mg (94%) of triazolophostin **10b** was obtained as a white hygroscopic solid: ^1^H NMR (500 MHz, D_2_O) *δ*: 3.74–3.81 (m, 5H, H-2′′, H-5_A_′, H-5_B_′, H-6_A_′′ and H-6_B_′′), 3.87 (dd, 1H, *J* = 12.8, 3.05 Hz, H-4′′), 3.94 (s, 3H, N-C*H*_3_), 4.08–4.10 (m, 1H, H-5′′), 4.47–4.50 (m, 2H, H-4′ and H-3′′), 4.66–4.70 (m, 1H, obscured by HDO peaks, H-3′), 5.24 (dd, 1H, *J* = 8.8, 4.45 Hz, H-2′), 5.27 (d, *J* = 3. 3 Hz, 1H, H-1′′), 6.46 (d, *J* = 3.9 Hz, 1H, H-1′), 7.76 (s, 1H, Im*H*-4), 8.63 (s, 1H, H-5), 8.80 (s, 1H, Im*H*-2); ^13^C NMR (125 MHz, D_2_O) *δ*: 34.8 (N-*C*H_3_), 60.3 (C-5′), 60.7 (C-6′′), 70.6 (C-2′′, ^31^P coupled), 71.7 (C-5′′, ^31^P coupled), 72.8 (C-4′′, ^31^P coupled), 73.6 (C-3′, ^31^P coupled), 76.5 (C-2′, ^31^P coupled), 77.7 (C-3′′, ^31^P coupled), 84.2 (C-4′), 91.2 (C-1′, ^31^P coupled), 98.0 (C-1′′), 118.8 (C-5), 124.8 (Im*C*-4), 125.0 (*C*-4), 134.1 (Im*C*-5), 136.4 (Im*C*-2); ^31^P NMR (202.4 MHz, CDCl_3_) *δ*: 3.62, 3.69, 4.41; HRMS (ESI) mass calcd for C_17_H_28_N_5_O_18_P_3_ [M + H]^+^, 684.072, found 684.0699.

#### (c) 4-Phenyl triazolophostin (**10c**)

By using 100 mg (0.067 mmol) of **9c**, 41 mg (91%) of triazolophostin **10c** was obtained as a white hygroscopic solid: ^1^H NMR (500 MHz, D_2_O) *δ*: 3.73–3.85 (m, 6H, H-2′′, H-5_A_′, H-4′′, H-5_B_′, H-6_A_′′, H-6_B_′′), 4.07–4.09 (m, 1H, H-5′′), 4.43 (bs, 1H, H-4′), 4.47–4.49 (m, 1H, H-3′′), 4.64–4.75 (m, 1H, H-3′), 5.19–5.20 (m, 1H, H-2′), 5.24 (s, 1H, H-1′′), 6.39 (d, 1H, 3.65 Hz, H-1′), 7.38–7.41 (m, 1H, Ar-*H*), 7.46 (t, *J* = 7.5 Hz, 2H, Ar-*H*), 7.78 (d, *J* = 7.5 Hz, 2H, Ar-*H*), 8.48 (s, 1H, H-5); ^13^C NMR (125 MHz, D_2_O) *δ*: 60.2 (C-5′), 60.8 (C-6′′), 70.4 (C-2′′, ^31^P coupled), 71.6 (C-5′′, ^31^P coupled), 73.0 (C-4′′, ^31^P coupled), 73.8 (C-3′, ^31^P coupled), 76.6 (C-2′, ^31^P coupled), 78.1 (C-3′′, ^31^P coupled), 83.9 (C-4′), 91.0 (C-1′, ^31^P coupled), 98.1 (C-1′′), 121.3 (C-5), 125.9, 126.0, 129.1, 129.2, 129.3 (Ar-*H*); ^31^P NMR (202.4 MHz, D_2_O) *δ*: 3.551, 3.597, 4.343; HRMS (ESI) mass calcd for C_19_H_28_N_3_O_18_P_3_ [M – H]^+^, 678.0503, found 678.0482.

#### (d) 4-(2-Pyridyl)-triazolophostin (**10d**)

By using 100 mg (0.067 mmol) of **9d**, 21.2 mg (47%) of triazolophostin **10d** was obtained as a sticky mass: ^1^H NMR (500 MHz, D_2_O) *δ*: 3.79–3.90 (m, 5H, H-6_A_′′, H-6_B_′′, H-5_A_′, H-5_B_′, H-2′′), 4.11–4.12 (m, 1H, H-5′′), 4.49–4.51 (m, 2H, H-4′ and H-4′′), 4.72 (m, 2H, obscured by HDO peaks, H-3′ and H-3′′), 5.26–5.29 (m, 2H, H-2′ and H-1′′), 6.51 (s, 1H, H-1′), 7.98 (s, 1H, Pyr-*H*), 8.40 (d, *J* = 6.5 Hz, 1H, Pyr-*H*), 8.62 (s, 1H, Pyr-*H*), 8.74 (s, 1H, Pyr-*H*), 9.04 (s, 1H, H-5); ^13^C NMR (125 MHz, D_2_O) *δ*: 60.3 (C-5′), 60.7 (C-6′′), 70.6 (C-2′′, ^31^P coupled), 71.8 (C-5′′, ^31^P coupled), 73.0 (C-4′′, ^31^P coupled), 73.8 (C-3′, ^31^P coupled), 76.7 (C-2′, ^31^P coupled), 78.0 (C-3′′, ^31^P coupled), 84.5 (C-4′), 91.4 (C-1′, ^31^P coupled), 98.1 (C-1′′), 124.9 (C-5), 126.2, 139.4, 141.3, 142.4, 147.6 (Ar-*C*); ^31^P NMR (202.4 MHz, D_2_O) *δ*: 3.637, 3.720, 4.448; HRMS (ESI) mass calcd for C_18_H_27_N_4_O_18_P_3_ [M – H]^+^, 679.0455, found 679.0431.

#### (e) 4-(2-Fluorophenyl)-triazolophostin (**10e**)

By using 190 mg (0.12 mmol) of **9e**, 79 mg (91%) of triazolophostin **10e** was obtained as a white hygroscopic solid: ^1^H NMR (500 MHz, MeOD) 3.58–3.81 (m, 6H, H-5′′, H-6_A_′′, H-6_B_′′, H-5_A_′, H-5_B_′, H-2′′), 4.09–4.11 (m, 1H, H-4′′), 4.30 (bs, 1H, H-4′), 4.51–4.53 (m, 1H, H-3′′), 4.68 (t, *J* = 5.0 Hz, 1H, H-3′), 5.15 (bs, 1H, H-1′′), 5.22 (bs, 1H, H-2′), 6.31 (bs, 1H, H-1′), 7.10–7.14 (m, 1H, Ar-*H*), 7.18 (t, *J* = 7.5 Hz, 1H, Ar-*H*), 7.28 (dd, *J* = 13.0, 6.5 Hz, 1H, Ar-*H*), 8.02 (t, *J* = 7.5 Hz, 1H, Ar-*H*), 8.51 (s, 1H, H-5); ^13^C NMR (125 MHz, MeOD) *δ*: 61.9 (C-5′), 62.1 (C-6′′), 72.7 (C-2′′, ^31^P coupled), 73.6 (C-5′′, ^31^P coupled), 74.6 (C-4′′, ^31^P coupled), 76.8 (C-3′, ^31^P coupled), 79.1 (C-2′, ^31^P coupled), 79.5 (C-3′′, ^31^P coupled), 85.3 (C-4′), 93.0 (C-1′, ^31^P coupled), 100.6 (C-1′′), 116.9 (C-5), 124.0, 125.8, 128.8, 131.1, 142.4, 159.8, 161.7 (Ar-*C*); ^31^P NMR (202.4 MHz, MeOD) *δ*: 0.601 (2P), –0.550; ^19^F NMR (470.68 MHz, MeOD) *δ*: –116.02; HRMS (ESI) mass calcd for C_19_H_27_FN_3_O_18_P_3_ [M]^+^, 697.0486, found 697.0490.

#### (f) 4-(3-Fluorophenyl)-triazolophostin (**10f**)

By using 180 mg (0.11 mmol) of **9f**, 78 mg (93%) of triazolophostin **10f** was obtained as a white hygroscopic solid: ^1^H NMR (500 MHz, MeOD) 3.61–3.62 (m, 1H, H-2′′), 3.69–3.84 (m, 5H, H-5′′, H-6_A_′′, H-6_B_′′, H-5_A_′, H-5_B_′), 4.13–4.18 (m, 1H, H-4′′), 4.32–4.33 (m, 1H, H-4′), 4.53–4.55 (m, 1H, H-3′′), 4.69 (t, *J* = 5.0 Hz, 1H, H-3′), 5.18 (d, *J* = 3.0 Hz, 1H, H-1′′), 5.27 (t, *J* = 3.8 Hz, 1H, H-2′), 6.32 (d, *J* = 3.0 Hz, 1H, H-1′), 7.01 (dt, *J* = 8.5, 2.0 Hz, 1H, Ar-*H*), 7.38 (dd, *J* = 8.0, 14.0 Hz, 1H, Ar-*H*), 7.51 (d, *J* = 9.7 Hz, 1H, Ar-*H*), 7.57 (d, *J* = 7.5 Hz, 1H, Ar-*H*), 8.58 (s, 1H, H-5); ^13^C NMR (125 MHz, MeOD) *δ*: 61.9 (C-5′), 62.0 (C-6′′), 72.5 (C-2′′, ^31^P coupled), 73.4 (C-5′′, ^31^P coupled), 75.2 (C-4′′, ^31^P coupled), 76.6 (C-3′, ^31^P coupled), 79.3 (C-2′, ^31^P coupled), 80.0 (C-3′′, ^31^P coupled), 85.4 (C-4′), 92.7 (C-1′, ^31^P coupled), 100.5 (C-1′′), 113.4 (C-5), 116.2, 121.8, 122.6, 132.0, 133.9, 148.0 (Ar-*C*); ^31^P NMR (202.4 MHz, MeOD) *δ*: –0.755, –0.131, 0.279; ^19^F NMR (470.68 MHz, MeOD) *δ*: –114.722; HRMS (ESI) mass calcd for C_19_H_27_FN_3_O_18_P_3_ [M]^+^, 697.0486, found 697.0472.

#### (g) 4-(4-Fluorophenyl)-triazolophostin (**10g**)

By using 200 mg (0.13 mmol) of **9g**, 88 mg (95%) of triazolophostin **10g** was obtained as a white hygroscopic solid: ^1^H NMR (500 MHz, MeOD) 3.60–3.83 (m, 6H, H-2′′, H-5′′, H-6_A_′′, H-6_B_′′, H-5_A_′, H-5_B_′), 4.13 (bs, 1H, H-4′′), 4.31 (bs, 1H, H-4′), 4.54 (bs, 1H, H-3′′), 4.68 (bs, 1H, H-3′), 5.17 (bs, 1H, H-1′′), 5.25 (bs, 1H, H-2′), 6.30 (bs, 1H, H-1′), 7.09 (t, *J* = 8.5 Hz, 2H, Ar-*H*), 7.76 (d, *J* = 5.5 Hz, 2H, Ar-*H*), 8.50 (s, 1H, H-5); ^13^C NMR (125 MHz, MeOD) *δ*: 61.9 (C-5′), 62.1 (C-6′′), 72.6 (C-2′′, ^31^P coupled), 73.5 (C-5′′, ^31^P coupled), 74.9 (C-4′′, ^31^P coupled), 76.7 (C-3′, ^31^P coupled), 79.1 (C-2′, ^31^P coupled), 79.7 (C-3′′, ^31^P coupled), 85.4 (C-4′), 92.8 (C-1′, ^31^P coupled), 100.5 (C-1′′), 116.8 (C-5), 116.9, 121.2, 128.0, 128.8, 128.9, 148.2, 163.3, 165.2 (Ar-*C*); ^31^P NMR (202.4 MHz, MeOD) *δ*: –0.602, –0.269, 0.473; ^19^F NMR (470.68 MHz, MeOD) *δ*: –115.406; HRMS (ESI) mass calcd for C_19_H_27_FN_3_O_18_P_3_ [M]^+^, 697.0486, found 697.0481.

#### (h) 4-(3,5-Difluorophenyl)-triazolophostin (**10h**)

By using 200 mg (0.13 mmol) of **9h**, 79 mg (84%) of triazolophostin **10h** was obtained as a white hygroscopic solid: ^1^H NMR (500 MHz, MeOD) 3.59–3.79 (m, 6H, H-2′′, H-5′′, H-6_A_′′, H-6_B_′′, H-5_A_′, H-5_B_′), 4.10 (bs, 1H, H-4′′), 4.30 (bs, 1H, H-4′), 4.52 (bs, 1H, H-3′′), 4.66 (bs, 1H, H-3′), 5.15 (bs, 1H, H-1′′), 5.22 (bs, 1H, H-2′), 6.28 (s, 1H, H-1′), 6.83 (t, *J* = 8.9 Hz, 1H, Ar-*H*), 7.36 (d, *J* = 6.2 Hz, 2H, Ar-*H*), 8.60 (s, 1H, H-5); ^13^C NMR (125 MHz, MeOD) *δ*: 61.9 (C-5′), 62.1 (C-6′′), 72.7 (C-2′′, ^31^P coupled), 73.6 (C-5′′, ^31^P coupled), 74.7 (C-4′′, ^31^P coupled), 76.7 (C-3′, ^31^P coupled), 79.0 (C-2′, ^31^P coupled), 79.5 (C-3′′, ^31^P coupled), 85.4 (C-4′), 92.9 (C-1′, ^31^P coupled), 100.6 (C-1′′), 104.2 (C-5), 109.3, 122.4, 135.2, 146.9, 163.9, 165.9 (Ar-*C*); ^31^P NMR (202.4 MHz, MeOD) *δ*: –0.650, 0.47, 0.81; ^19^F NMR (470.68 MHz, MeOD) *δ*: –111.10; HRMS (ESI) mass calcd for C_19_H_26_F_2_N_3_O_18_P_3_ [M]^+^, 715.0392, found 715.0402.

#### (i) 4-(4-Methoxyphenyl)-triazolophostin (**10i**)

By using 195 mg (0.12 mmol) of **9i**, 87 mg (96%) of triazolophostin **10i** was obtained as a white hygroscopic solid: ^1^H NMR (500 MHz, D_2_O) *δ*: 3.65–3.76 (m, 9H, H-2′′, H-5′′, H-6_A_′′, H-6_B_′′, H-5_A_′, H-5_B_′ and 1 × OC*H*_3_), 4.00–4.02 (m, 1H, H-4′′), 4.33 (bs, 1H, H-4′), 4.40–4.41 (m, 1H, H-3′′), 4.54 (t, *J* = 5.1 Hz, 1H, H-3′), 5.08–5.15 (m, 1H, H-2′), 5.16 (bs, 1H, H-1′′), 6.27 (d, *J* = 3.2 Hz, 1H, H-1′), 6.89 (d, *J* = 8.6 Hz, 1H, Ar-*H*), 7.54 (d, *J* = 8.6 Hz, 1H, Ar-*H*), 8.25 (s, 1H, H-5); ^13^C NMR (125 MHz, D_2_O) *δ*: 55.6 (O*C*H_3_), 60.3 (C-5′), 61.0 (C-6′′), 70.7 (C-2′′, ^31^P coupled), 71.7 (C-5′′, ^31^P coupled), 73.0 (C-4′′, ^31^P coupled), 73.9 (C-3′, ^31^P coupled), 76.5 (C-2′, ^31^P coupled), 78.0 (C-3′′, ^31^P coupled), 83.8 (C-4′), 91.2 (C-1′, ^31^P coupled), 98.2 (C-1′′), 114.8 (C-5), 120.6, 122.1, 127.5, 147.4, 159.5 (Ar-*C*); ^31^P NMR (202.4 MHz, MeOD) *δ*: –0.55, 0.66 (2P); HRMS (ESI) mass calcd for C_20_H_30_N_3_O_19_P_3_ [M]^+^, 709.0686, found 709.0691.

#### (j) 4-(2-Formylphenyl)-triazolophostin (**10j**)

By using 190 mg (0.12 mmol) of **9j**, 78 mg (88%) of triazolophostin **10j** was obtained as a white hygroscopic solid: ^1^H NMR (500 MHz, D_2_O) *δ*: 3.75–3.87 (m, 6H, H-2′′, H-5′′, H-6_A_′′, H-6_B_′′, H-5_A_′ and H-5_B_′), 4.09–4.11 (m, 1H, H-4′′), 4.44 (bs, 1H, H-4′), 4.50 (bs, 1H, H-3′′), 4.65–4.68 (m, 1H, H-3′), 5.21 (bs, 1H, H-2′), 5.25 (bs, 1H, H-1′′), 6.40–6.42 (m, 1H, H-1′), 7.34 (bs, 1H, Ar-*H*), 7.46–7.47 (m, 1H, Ar-*H*), 7.60–7.61 (m, 1H, Ar-*H*), 7.77 (s, 1H, H-5 (triazole)), 8.36–8.48 (m, 1H, C*H*(OH)_2_); ^13^C NMR (125 MHz, D_2_O) *δ*: 60.3 (C-5′), 60.9 (C-6′′), 71.5 (C-2′′, ^31^P coupled), 72.0 (C-5′′, ^31^P coupled), 72.2 (C-4′′, ^31^P coupled), 73.4 (C-3′, ^31^P coupled), 76.1 (C-2′, ^31^P coupled), 77.1 (C-3′′, ^31^P coupled), 83.8 (C-4′), 91.2 (C-1′, ^31^P coupled), 97.8 (C-1′′), 121.2 (C-5), 125.8, 126.3, 129.0, 129.3, 130.9, 136.5, 146.7, 147.4, (Ar-*C*); ^31^P NMR (202.4 MHz, D_2_O) *δ*: 3.51, 3.63, 4.32; HRMS (ESI) mass calcd for C_20_H_28_N_3_O_19_P_3_ [M]^+^, 707.0530, found 707.0549.

#### (k) 4-(6-Methoxynaphth-2-yl)-triazolophostin (**10k**)

By using 90 mg (0.057 mmol) of **9k**, 42 mg (97%) of triazolophostin **10k** was obtained as a white hygroscopic solid: ^1^H NMR (500 MHz, MeOD) *δ*: 3.06 (d, *J* = 7.7 Hz, 1H, H-2′′), 3.69–3.85 (m, 8H, H-5′′, H-5_A_′, H-5_B_′, H-6_A_′′, H-6_B_′′ and OC*H*_3_), 4.13–4.15 (m, 1H, H-4′′), 4.32–4.33 (m, 1H, H-4′), 4.53–4.55 (m, 1H, H-3′′), 4.70 (t, *J* = 5.0 Hz, 1H, H-3′), 5.17 (bs, 1H, H-1′′), 5.28 (bs, 1H, H-2′), 6.32 (d, *J* = 2.7 Hz, 1H, H-1′), 7.05 (dd, *J* = 9.0, 2.4 Hz, 1H, Ar-*H*), 7.15 (d, *J* = 2.2 Hz, 1H, Ar-*H*), 7.69 (d, *J* = 9.0 Hz, 1H, Ar-*H*), 7.73 (d, *J* = 9.0 Hz, 1H, Ar-*H*), 7.79 (s, 1H, H-5), 8.13 (s, 1H, Ar-*H*), 8.57 (s, 1H, Ar-*H*); ^13^C NMR (125 MHz, MeOD) *δ*: 55.9 (O*C*H_3_), 61.9 (C-5′), 62.1 (C-6′′), 72.6 (C-2′′, ^31^P coupled), 73.5 (C-5′′, ^31^P coupled), 75.0 (C-4′′, ^31^P coupled), 77.0 (C-3′, ^31^P coupled), 79.3 (C-2′, ^31^P coupled), 79.5 (C-3′′, ^31^P coupled), 85.4 (C-4′), 92.9 (C-1′, ^31^P coupled), 100.7 (C-1′′), 106.9 (C-5), 120.4, 121.2, 125.2, 125.5, 126.6, 128.7, 130.4, 130.7, 136.1, 149.4, 159.7 (Ar-*C*); ^31^P NMR (202.4 MHz, MeOD) *δ*: 3.605 (2P), 4.393; HRMS (ESI) mass calcd for C_24_H_32_N_3_O_19_P_3_ [M – H]^+^, 758.0765, found 758.0785.

### 8. Biological assay

Ca^2+^ release from the intracellular stores of saponin-permeabilized DT40 cells expressing only type 1 IP_3_R was measured using a low-affinity Ca^2+^ indicator (Mag-fluo-4) as described previously.^23^ Briefly, the ER was loaded with Ca^2+^ to the steady state for ∼120 s by addition of 1.5 mM MgATP in medium containing *p*-trifluoromethoxyphenylhydrazone (FCCP) to inhibit mitochondria. IP_3_, AdA or triazolophostins were added with cyclopiazonic acid (10 μM) to inhibit further Ca^2+^ uptake. Ca^2+^ release was assessed 10–20 s after addition of the analog, and expressed as a fraction of the ATP-dependent Ca^2+^ uptake.
