# Supplementary material for: Triazolophostins: a library of novel and potent agonists of IP3 receptors
Source: Org Biomol Chem. 2015 Apr 14;13(24):6698–710. doi: 10.1039/c5ob00440c (PMC4533600; doi:10.1039/c5ob00440c)
Supplement: Supplementary file 1 [file OB-013-C5OB00440C-s001.pdf]

Electronic Supplementary Material (ESI) for Organic & Biomolecular Chemistry.
This journal is © The Royal Society of Chemistry 2015

Supplementary Information for

Triazolophostins: A Library of Novel and Potent Agonists of IP_3 Receptors

Amol M. Vibhute,[†] Vera Konieczny,[‡] Colin W. Taylor,[‡] and Kana M. Sureshan*[,†]

[†]*School of Chemistry, Indian Institute of Science Education and Research, Thiruvananthapuram, KERALA-695016, India, E-mail: kms@iisertvm.ac.in.* [‡]*Department of Pharmacology, Tennis Court Road, University of Cambridge, Cambridge CB2 1PD, U.K.*

INDEX

1. Crystal structure of **4**	S2	15. NMR data of **10e**	S73-S78
2. Docking study	S2-S5	16. NMR data of **9f**	S79-S86
3. NMR data of **6**	S6-S10	17. NMR data of **10f**	S87-S93
4. NMR data of **7**	S11-S16	18. NMR data of **9g**	S94-S101
5. NMR data of **8**	S17-S22	19. NMR data of **10g**	S102-S107
6. NMR data of **9a**	S23-S28	20. NMR data of **9h**	S108-S114
7. NMR data of **10a**	S29-S32	21. NMR data of **10h**	S115-S120
8. NMR data of **9b**	S33-S37	22. NMR data of **9i**	S121-S126
9. NMR data of **10b**	S38-S42	23. NMR data of **10i**	S127-S132
10. NMR data of **9c**	S43-S48	24. NMR data of **9j**	S133-S138
11. NMR data of **10c**	S49-S53	25. NMR data of **10j**	S139-S143
12. NMR data of **9d**	S54-S59	26. NMR data of **9k**	S144-S149
13. NMR data of **10d**	S60-S65	27. NMR data of **10k**	S150-S154
14. NMR data of **9e**	S66-S72	28. References	S155

1. Crystal structure of disaccharide 4

The disaccharide **4** was crystallized from the mixture of ethyl acetate: petroleum ether (1:3, v/v) by slow evaporation.

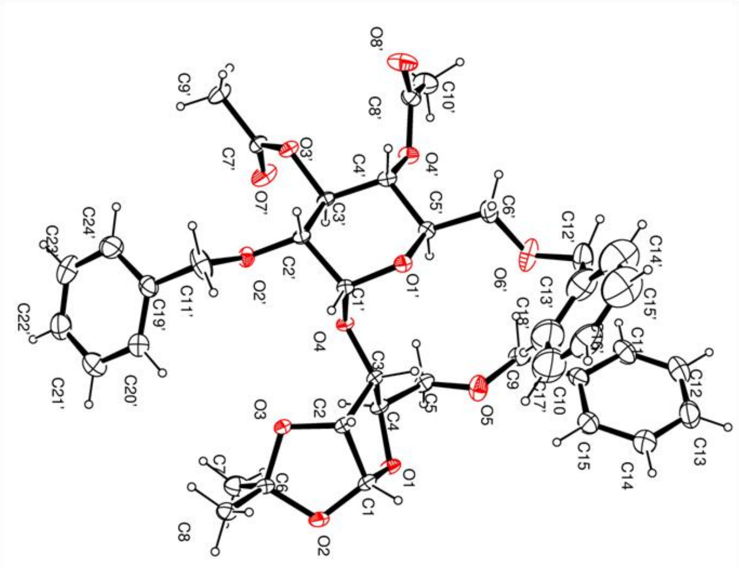

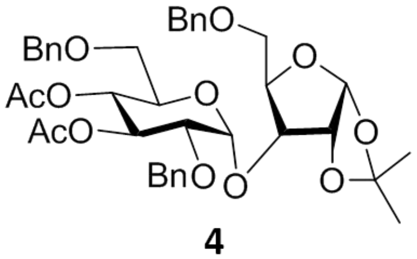

4

Figure S1. ORTEP diagram and molecular structure of disaccharide **4**.

Crystal data for 4:

CCDC 1022279: Refined formula: $C_{39}H_{46}O_{12}$, Formula weight: M = 706.79, colorless block, 0.25 x 0.15 x 0.15 mm^3, Monoclinic, space group: P2(1), Unit cell dimensions and volume: a = 13.088(5), b = 9.287(5), c = 16.183(5) Å, V = 2975.2(2) $Å^3$, No of formula units in the unit cell Z = 2, T = 296(2) K, $2\theta_{max}$ = 49.40°, Calculated density $\rho_{calcd:}$ (g cm^{-3}) = 1.229, F(000) = 752, Linear absorption coefficient μ: 0.091 mm^{-1}, 14958 reflections collected, 6326 unique reflections (R_{int} = 0.0788), multi-scan absorption correction, T_{min} = 0.9777, T_{max} = 0.9865, number of parameters = 431, number of restraints = 0, GoF = 1.074, R_1 = 0.0650, wR_2 = 0.1711, R indices based on 5392 reflections with I >2\s(I) (refinement on F2). $\Delta\rho_{max}$ = 0.028, $\Delta\rho_{min}$ = 0.002 ($eÅ^{-3}$).

2. Docking Study:

The structures of all the triazolophostins were energy-minimized using MM2 program in ChemBio3D Ultra 12.0 platform. The reported crystal structure of the binding domain of

IP_3R (pdb code: 1N4K) was used as receptor after deleting IP_3 and water molecules from its structure. The docking was done by using Auto Dock Vina 1 [1]. By docking with IP_3, we could reproduce almost all the interactions (shown by IP_3 with IP_3R1) in the reported crystal structure[2] showing the fidelity of the docking results (fig. S4). The docking of AdA also showed similar mode of binding and interactions as reported earlier.[3] Interestingly, the parent compound triazolophostin **10a** show similar interactions as AdA suggesting that the replacement of the nucleobase with an easily accessible triazole group did not compromise on interactions (Fig. S2). This allows lot of flexibility for the design of various ligands for different levels of probing. 3-fluorotriazolophostin **10f** was equipotent as AdA itself suggesting that the additional fluorophenyl group contribute towards its excess affinity. Docking results suggest that the triazole ring forms cation- π interaction with Arg504 and fluorine forms fluorine-oxygen interactions[4] with D566, Q565 and Q507 (Fig. S3).

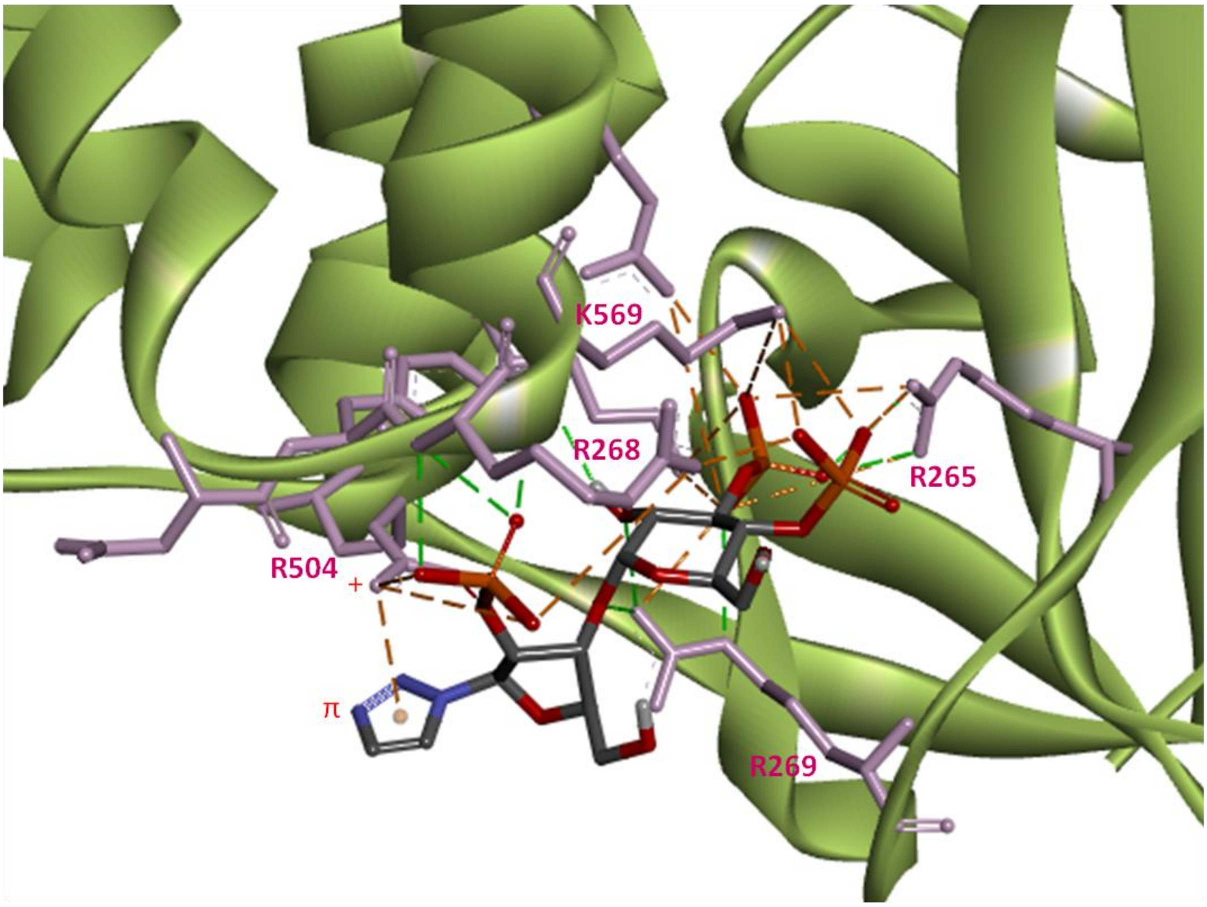

Fig. S2. Docking of triazolophostin **10a** with IBC showing the preferred binding mode and important cation-π interaction with Arg504. The cation-π distace R = 3.4 Å. Color code for ligand: carbon, gray; oxygen, red; nitrogen, blue; phosphorous, yellow. Docking conditions: Exhaustiveness = 8, grid parameters, center_x = 22.5, y = -1.8, z = 11.5; size_x = 24, y = 36, z = 30.

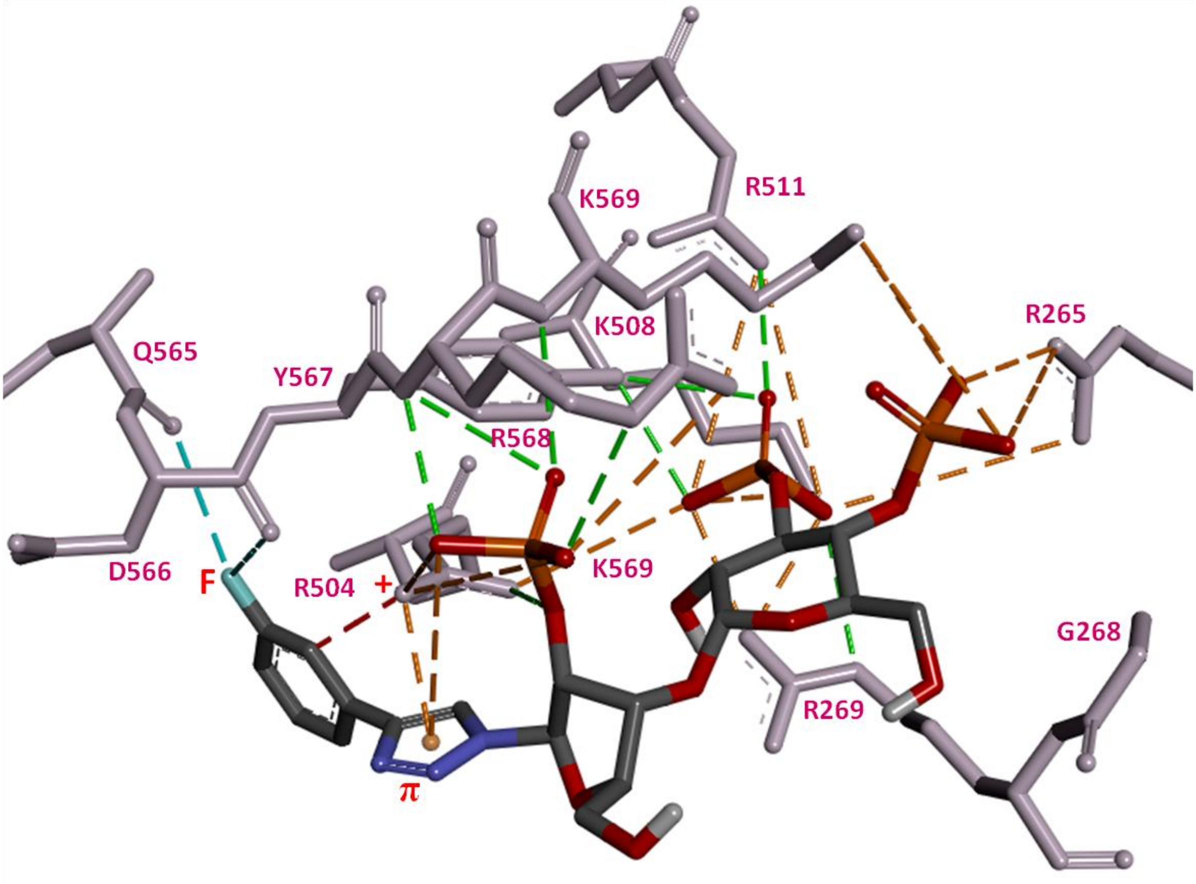

Fig. S3. Docking of 3-fluorophenyl triazolophostin **10f** with IBC showing the preferred binding mode and important cation-π interaction with Arg504. Color code for ligand: carbon, gray; oxygen, red; nitrogen, blue; phosphorous, yellow; fluorine, cyan. The protein ribbons and water molecules were omitted for clarity. Docking conditions: Exhaustiveness = 8, grid parameters, center_x = 22.5, y = -1.8, z = 11.5; size_x = 24, y = 36, z = 30.

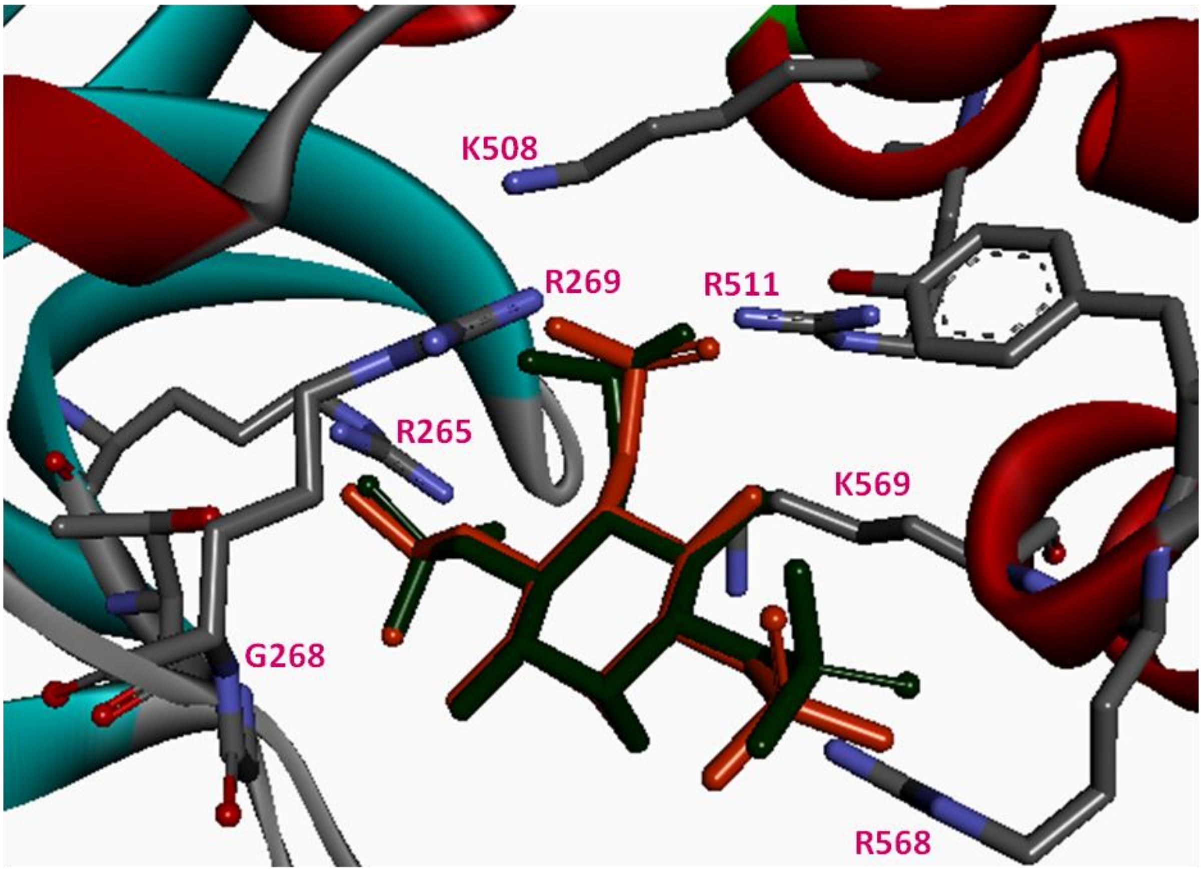

Fig. S4. The overlay of binding mode of IP_3 (brown) obtained from the crystal structure of IP_3R1 with IP_3 and the docked IP_3 (green) with IP_3R1. The water molecules and hydrogen atoms were removed for clarity.

^{1}H NMR of 6 in $CDCl_3$

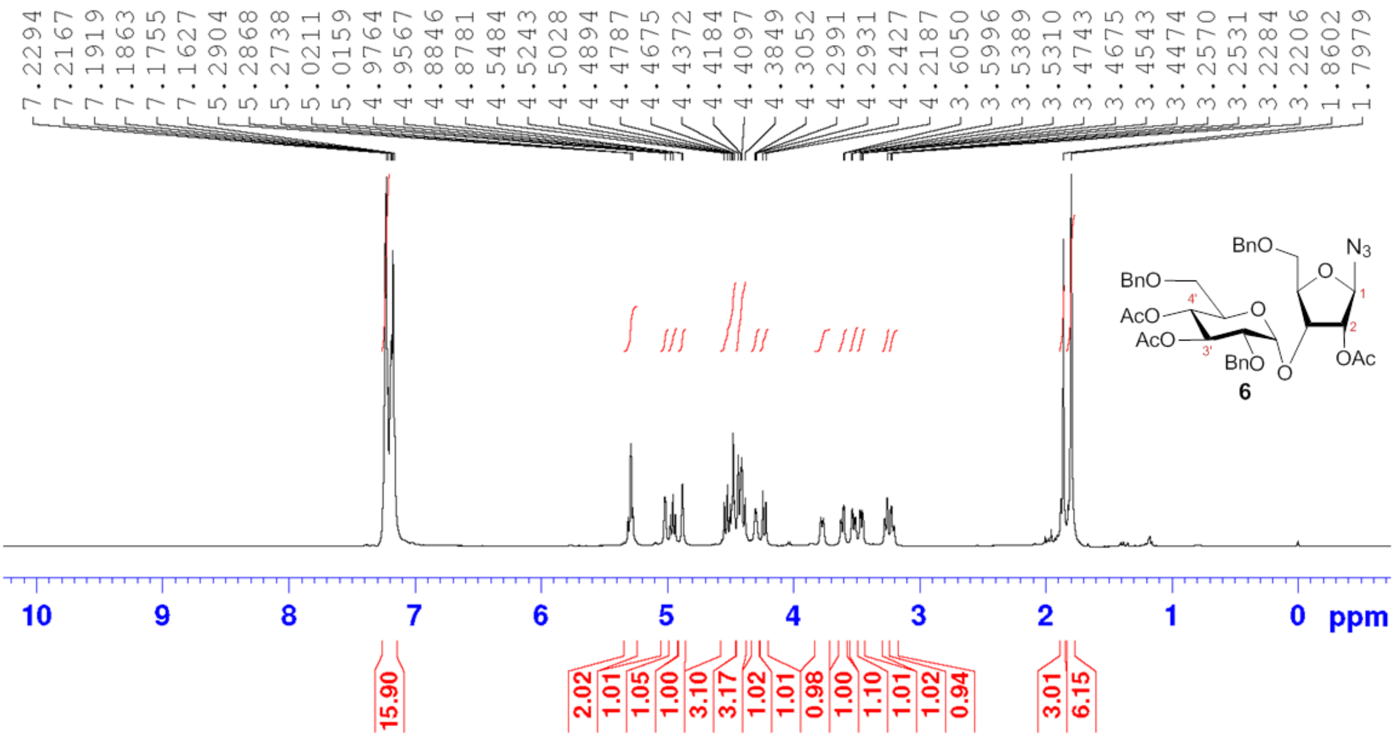

zoom

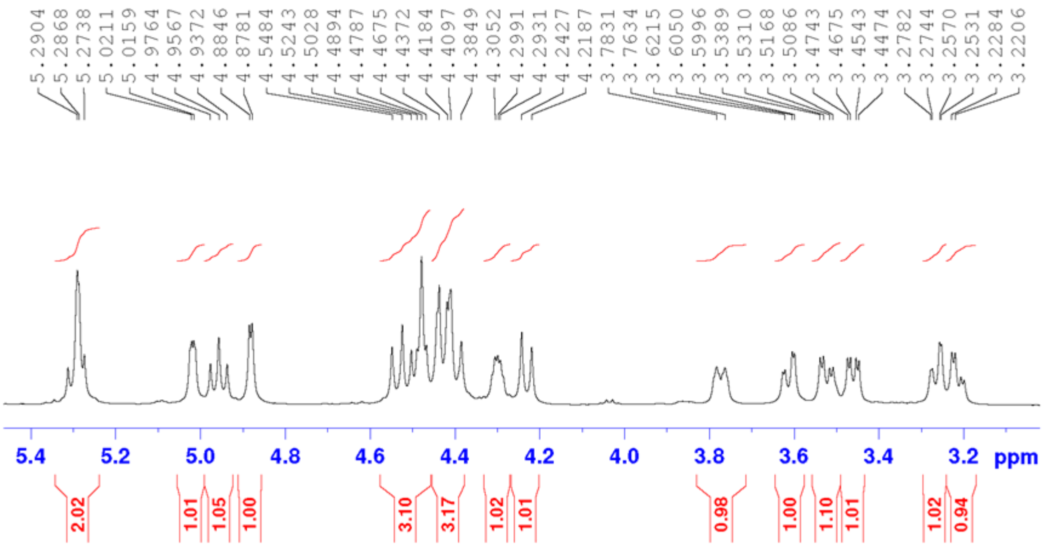

COSY of 6 in $CDCl_3$

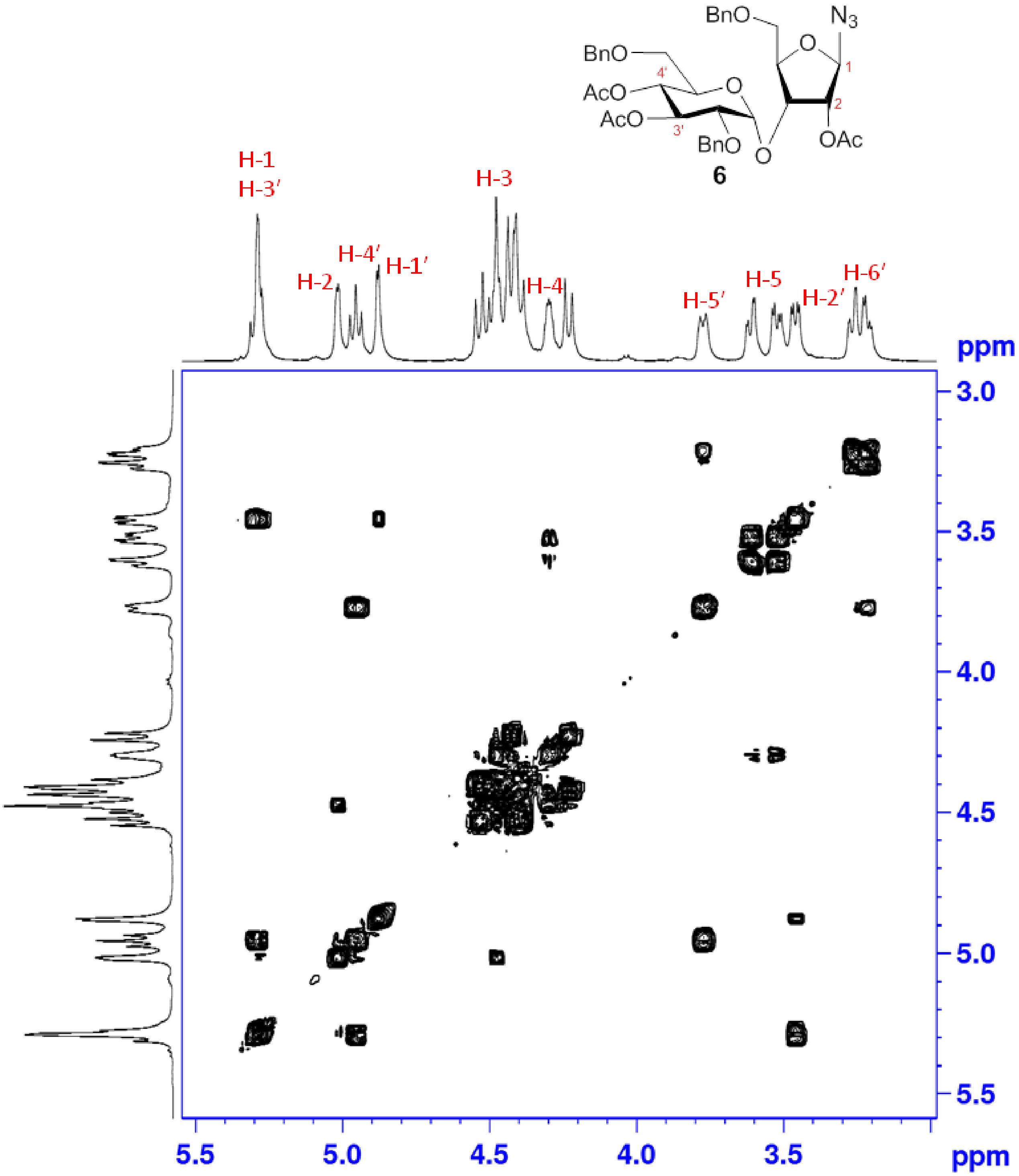

^{13}C NMR of 6 in $CDCl_3$

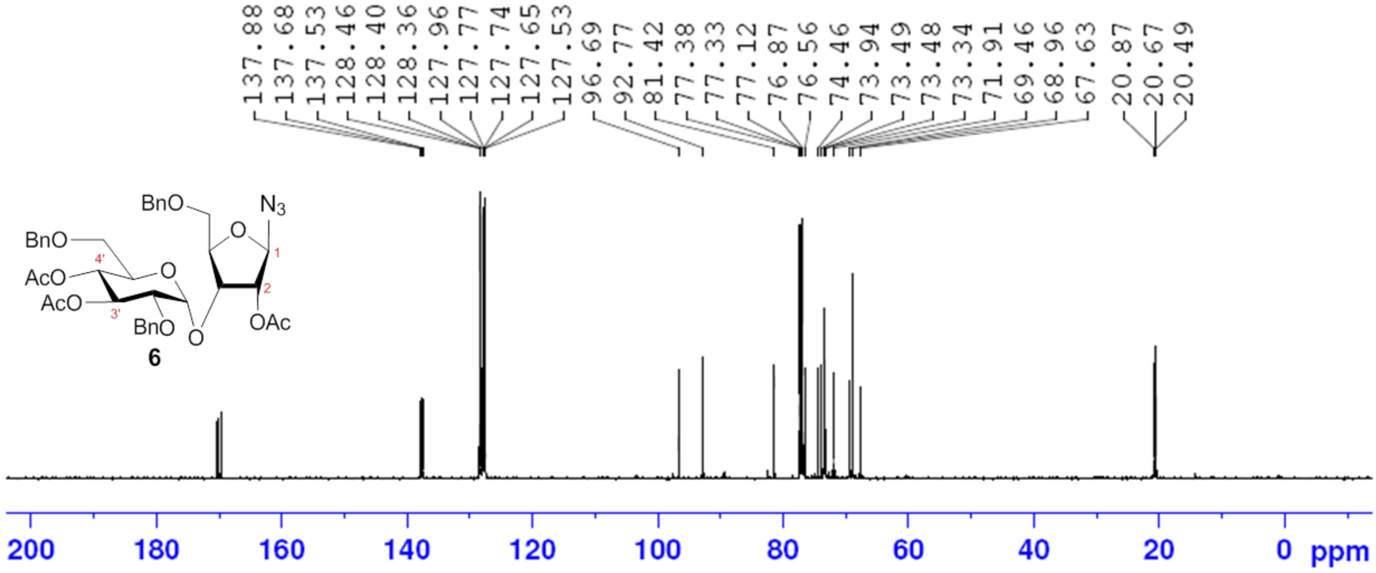

DEPT of 6 in $CDCl_3$

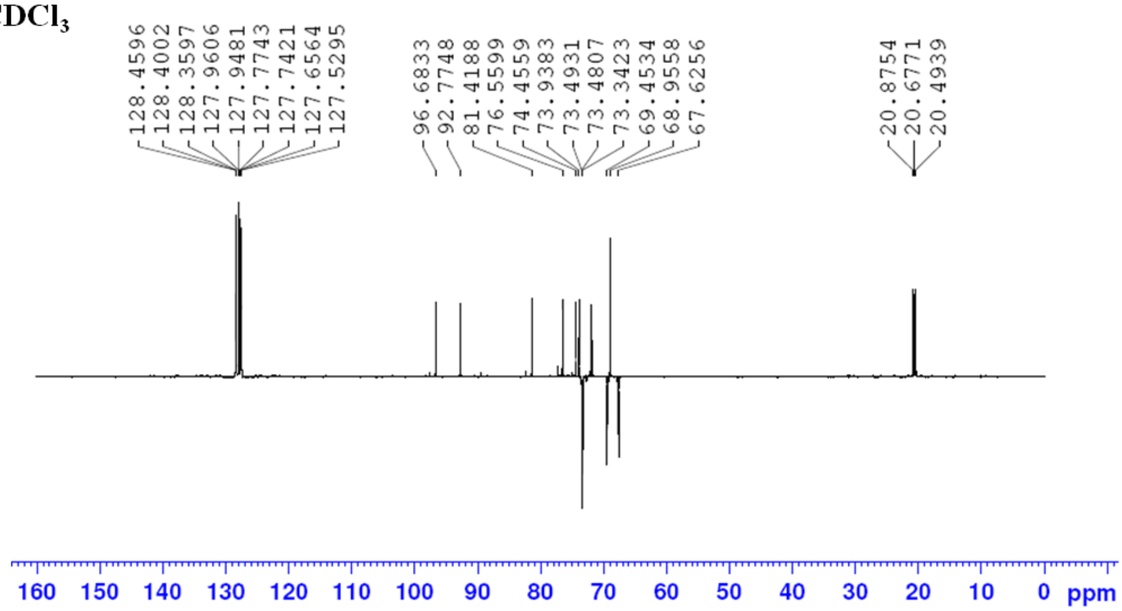

HMBC of 6 in $CDCl_3$

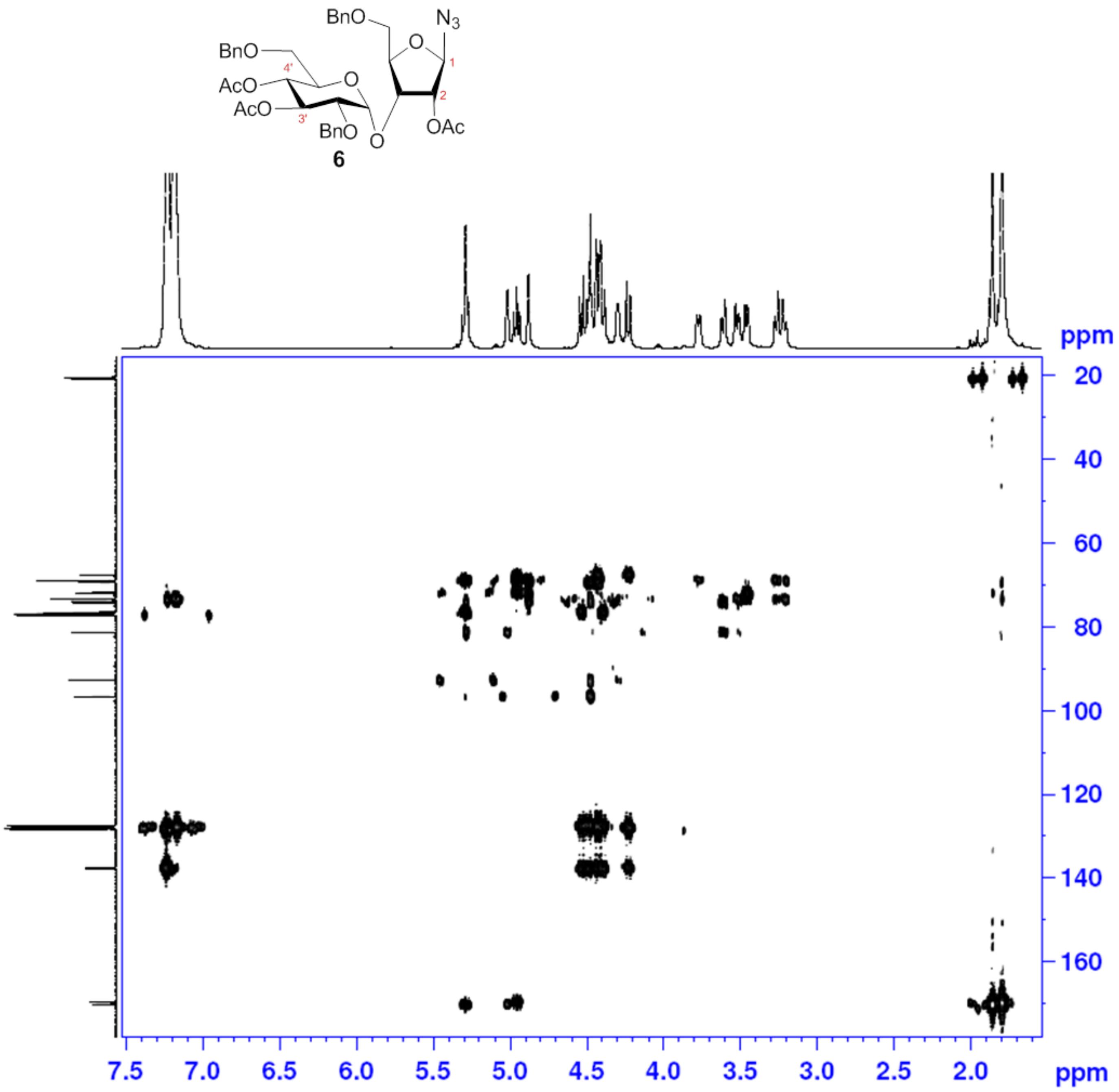

HMQC of 6 in $CDCl_3$

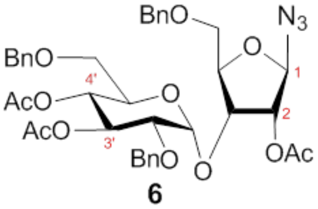

6

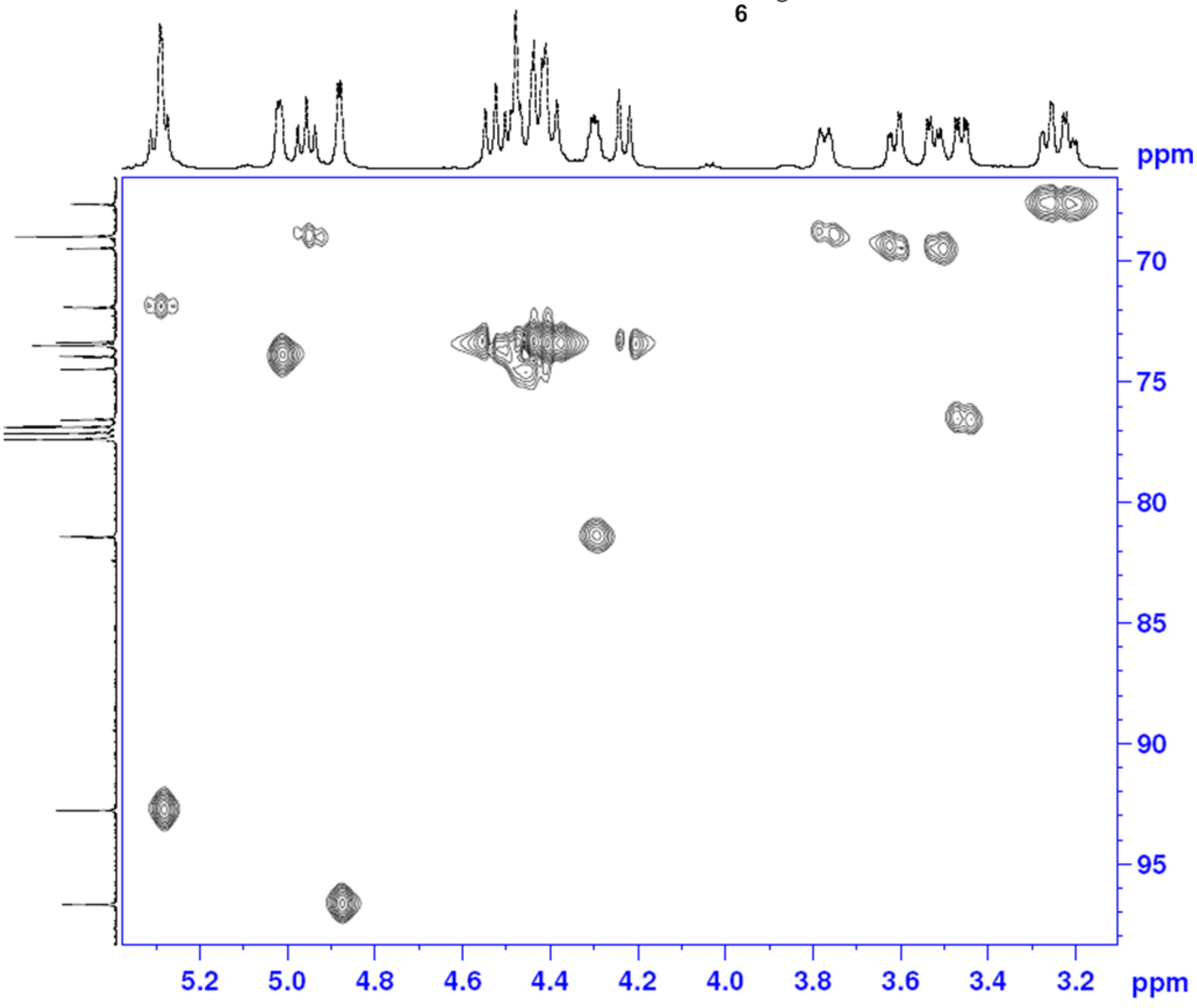

^{1}H NMR of 7 in CDCl$_3$

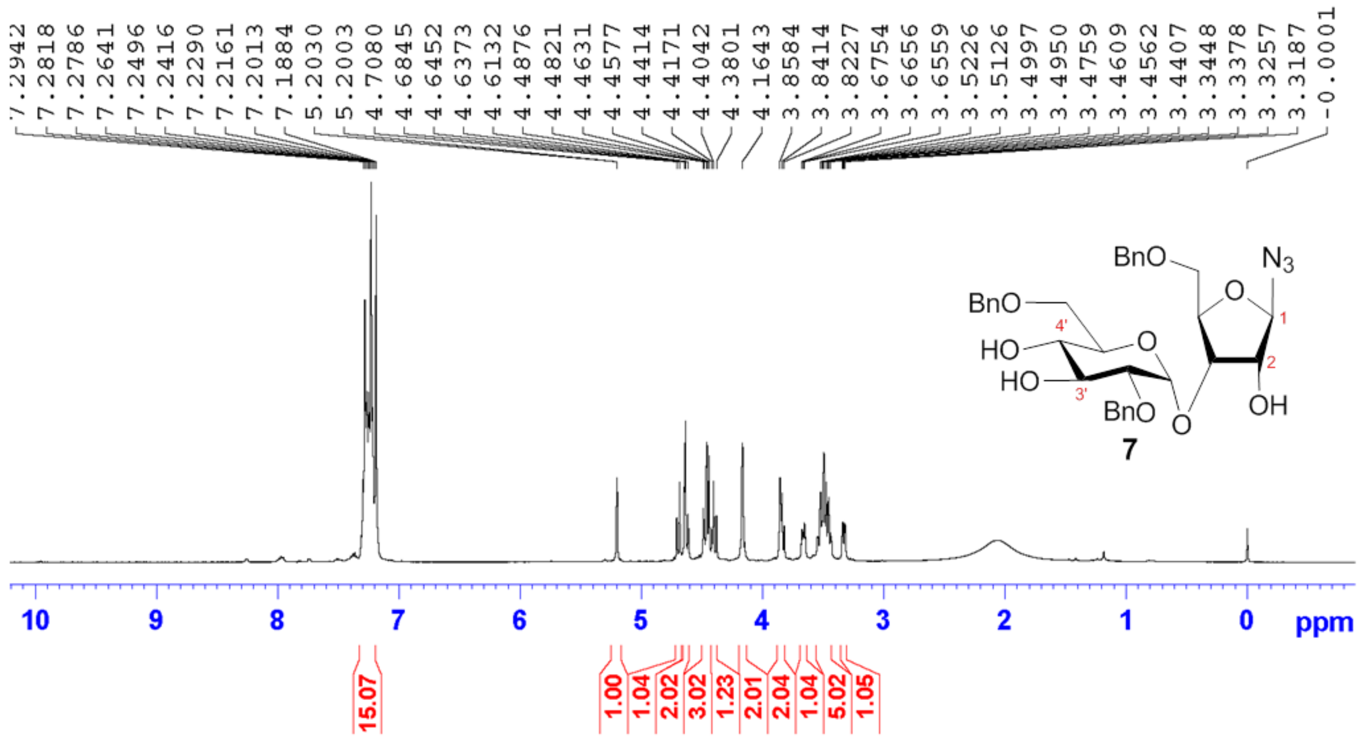

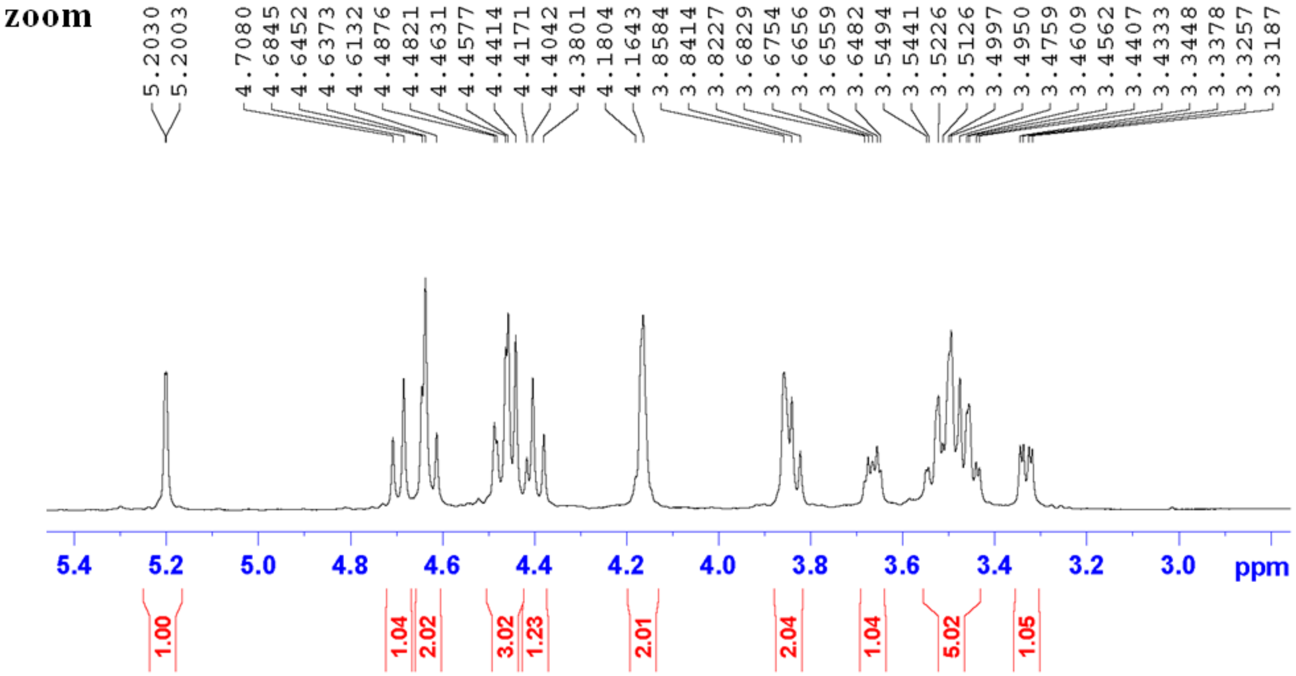

COSY of 7 in $CDCl_3$

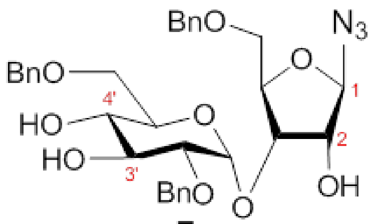

7

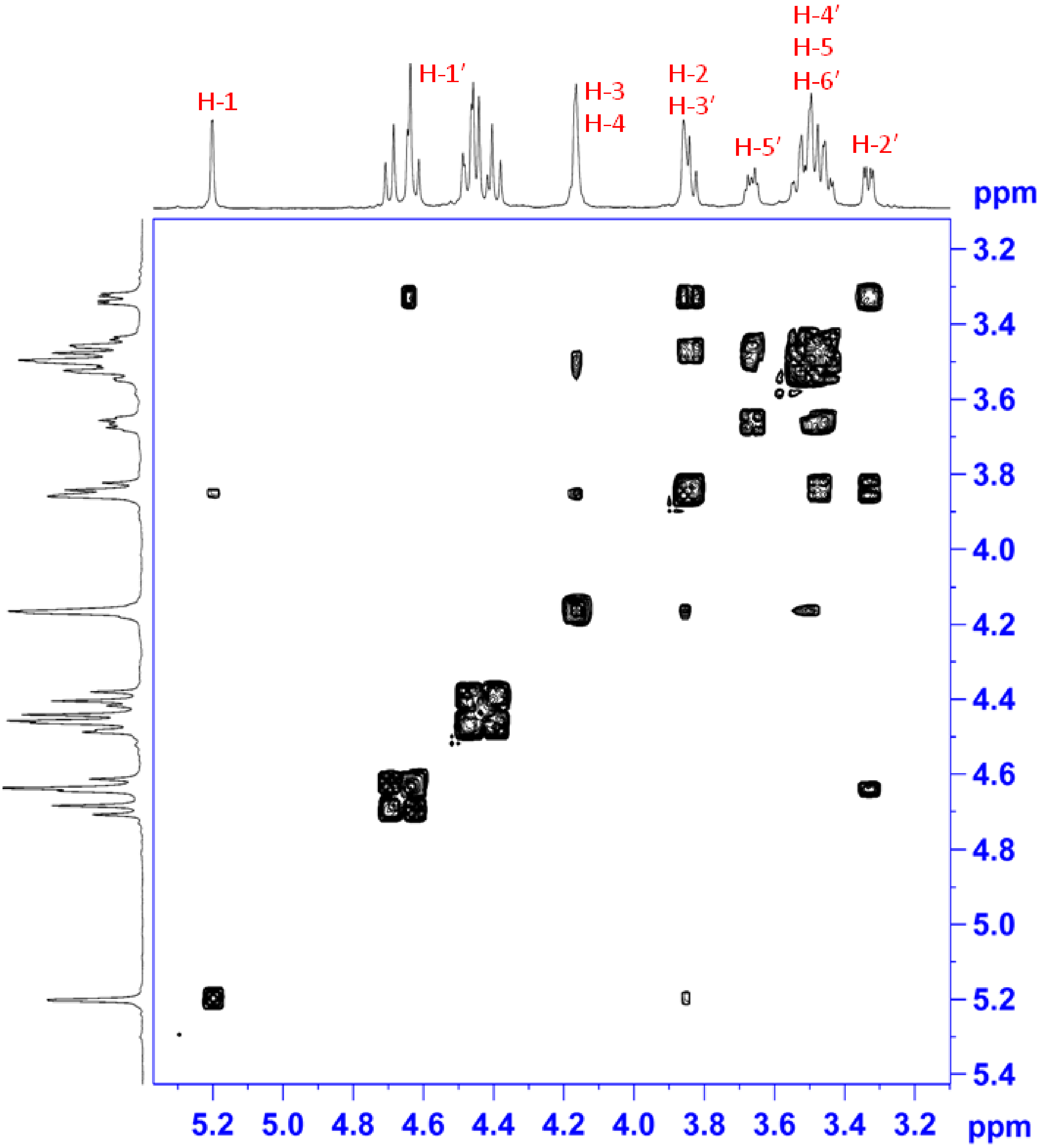

^{13}C NMR of 7 in $CDCl_3$

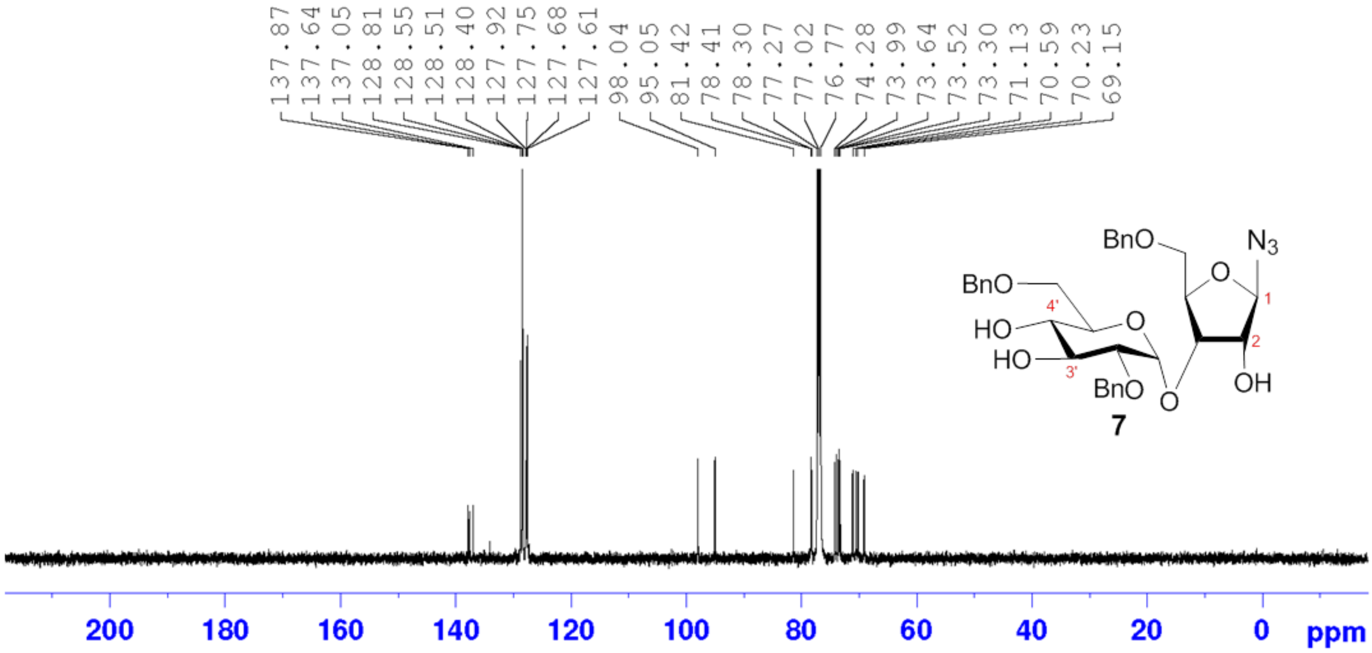

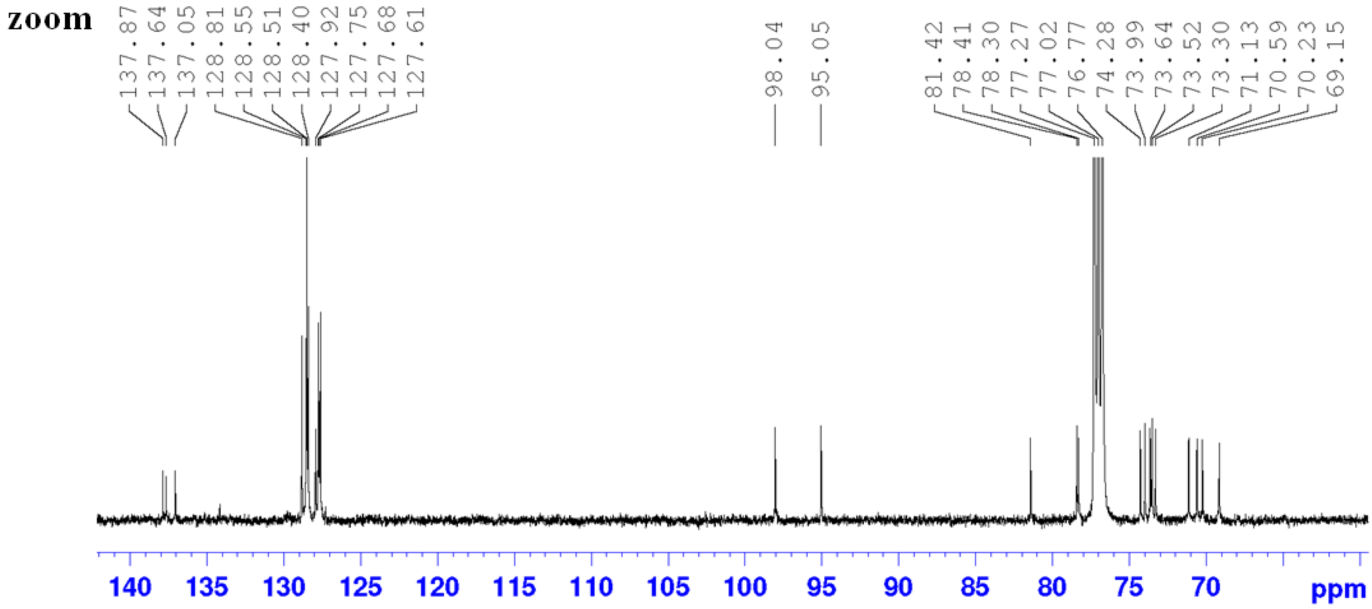

DEPT of 7 in $CDCl_3$

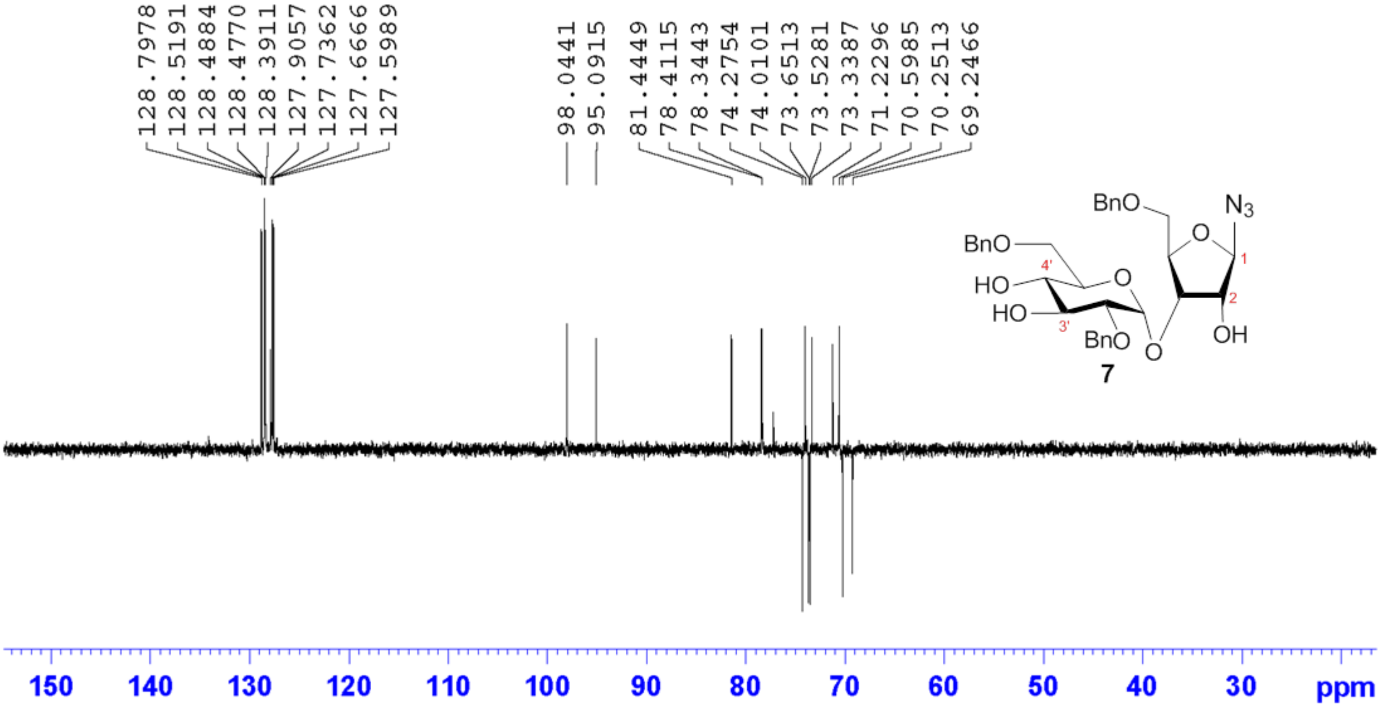

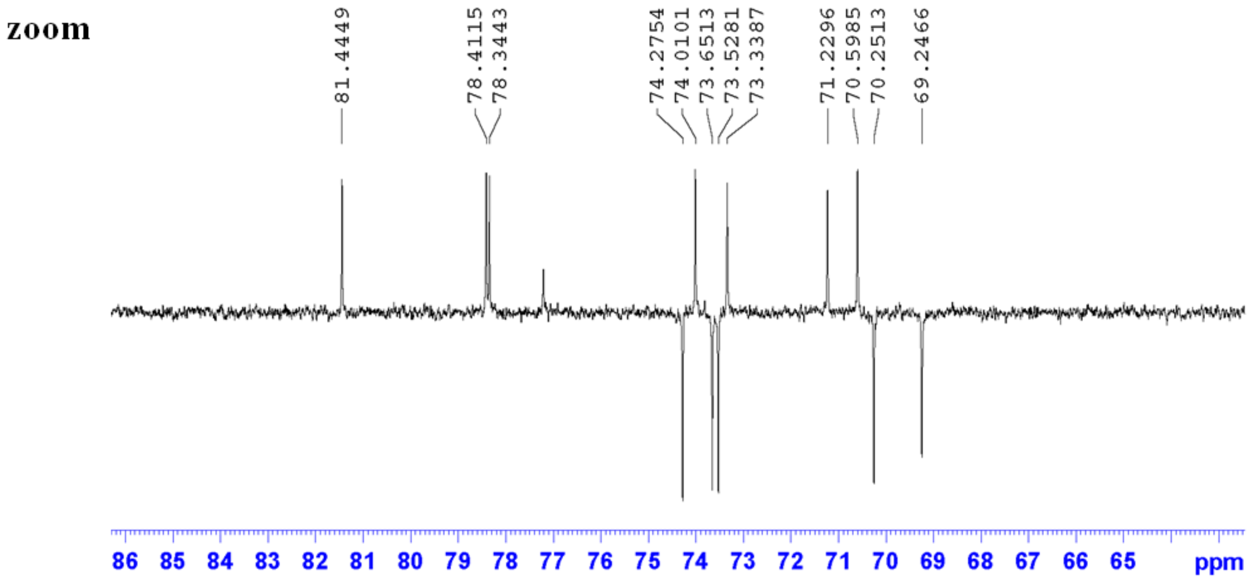

HMBC of 7 in $CDCl_3$

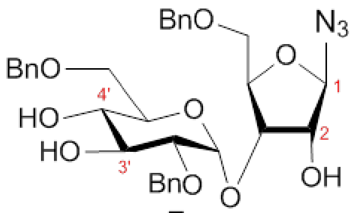

7

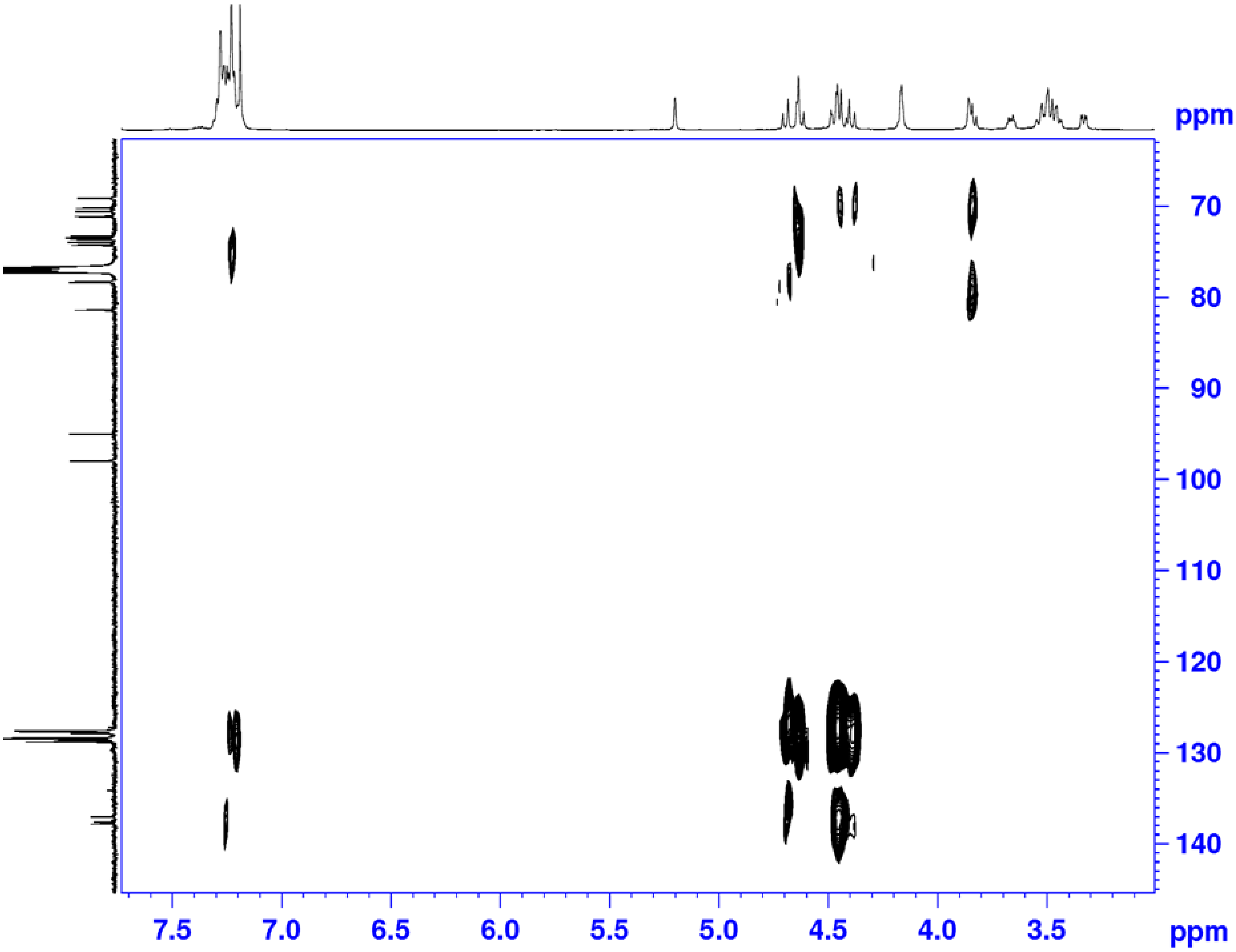

HMQC of 7 in $CDCl_3$

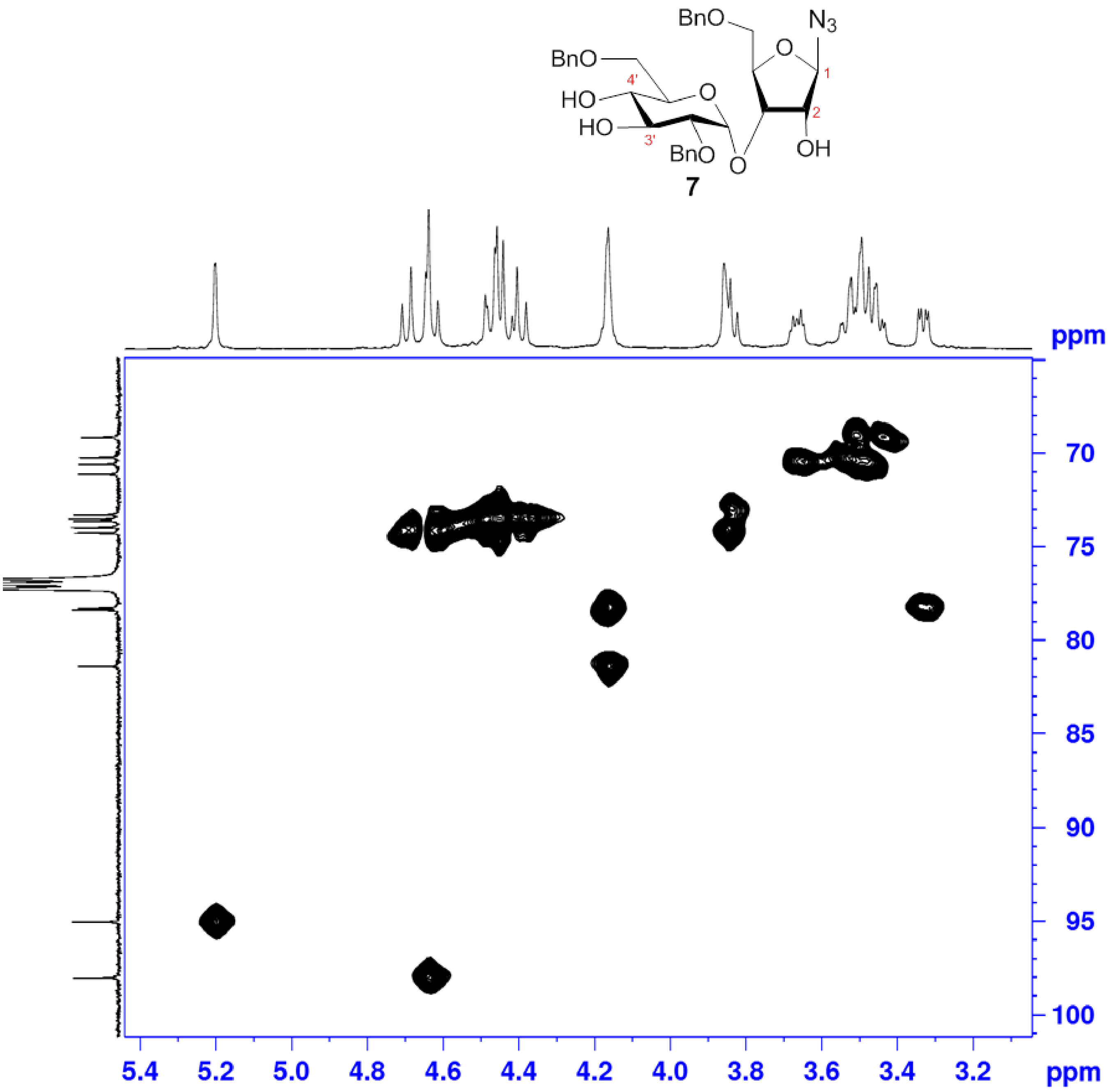

^{1}H NMR of 8 in $CDCl_3$

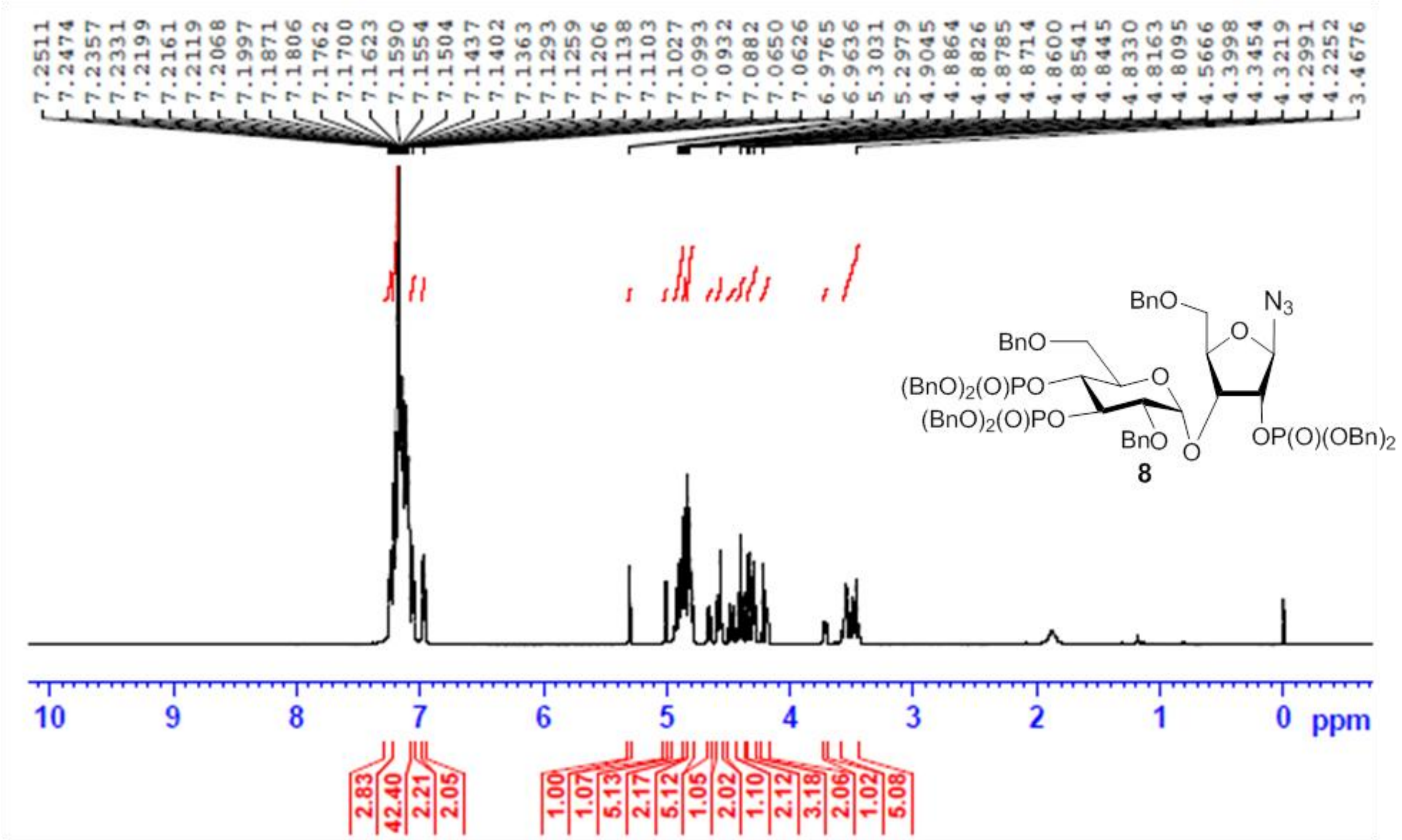

zoom

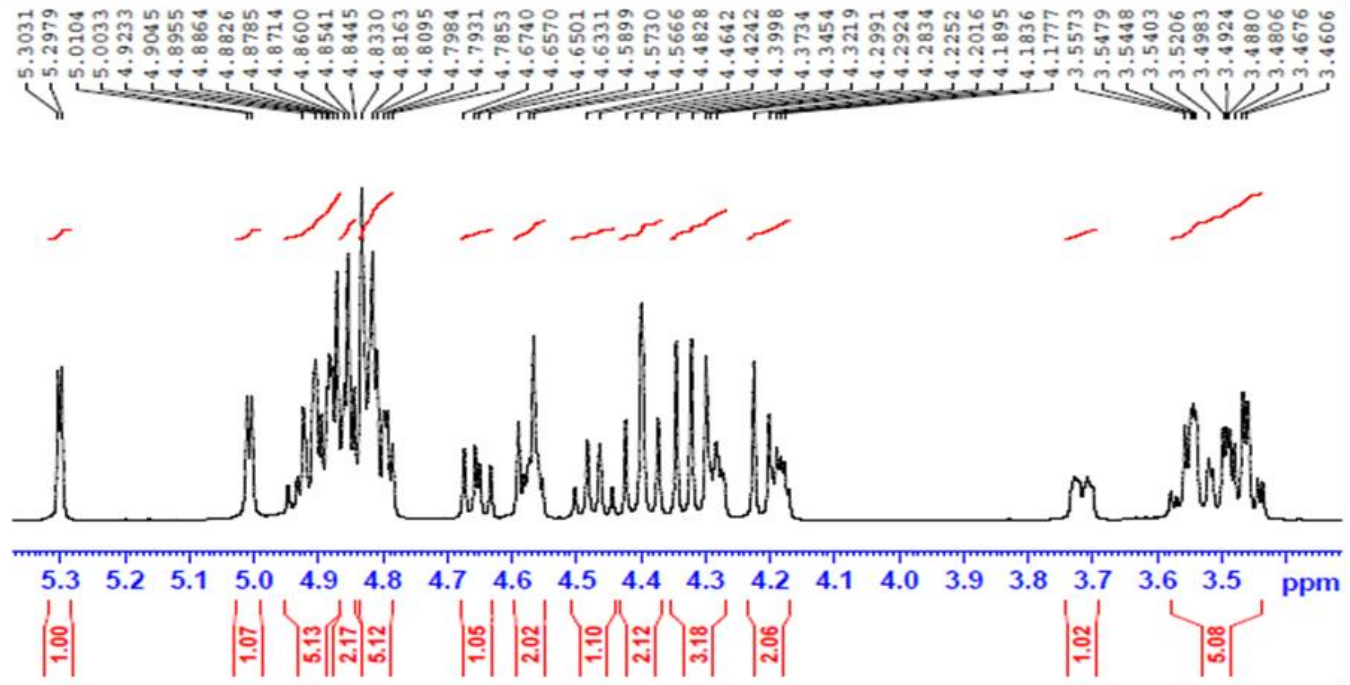

COSY of 8 in $CDCl_3$

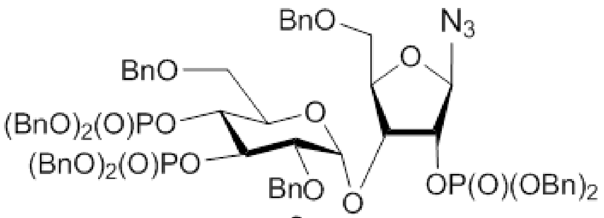

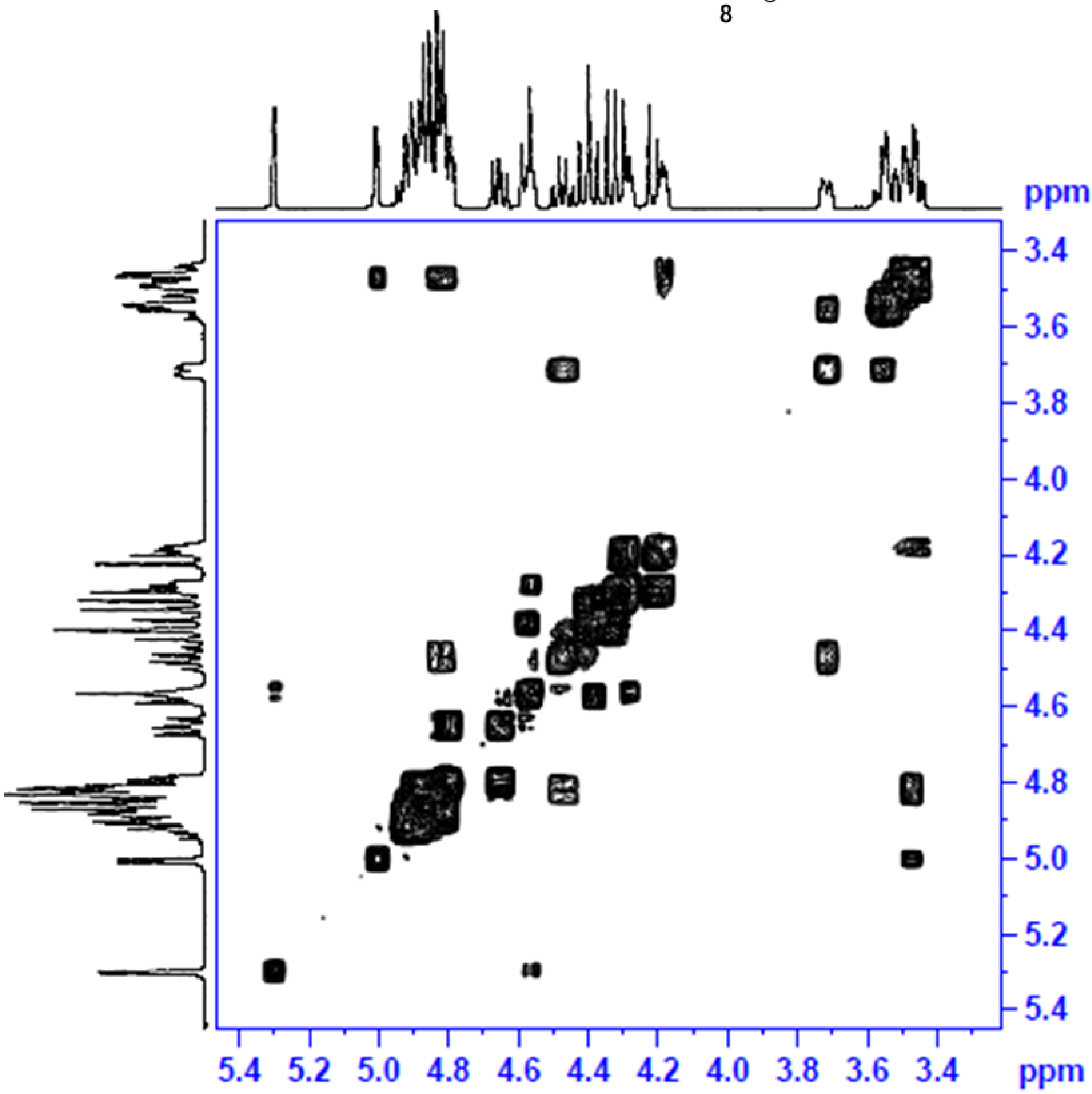

^{13}C NMR of 8 in $CDCl_3$

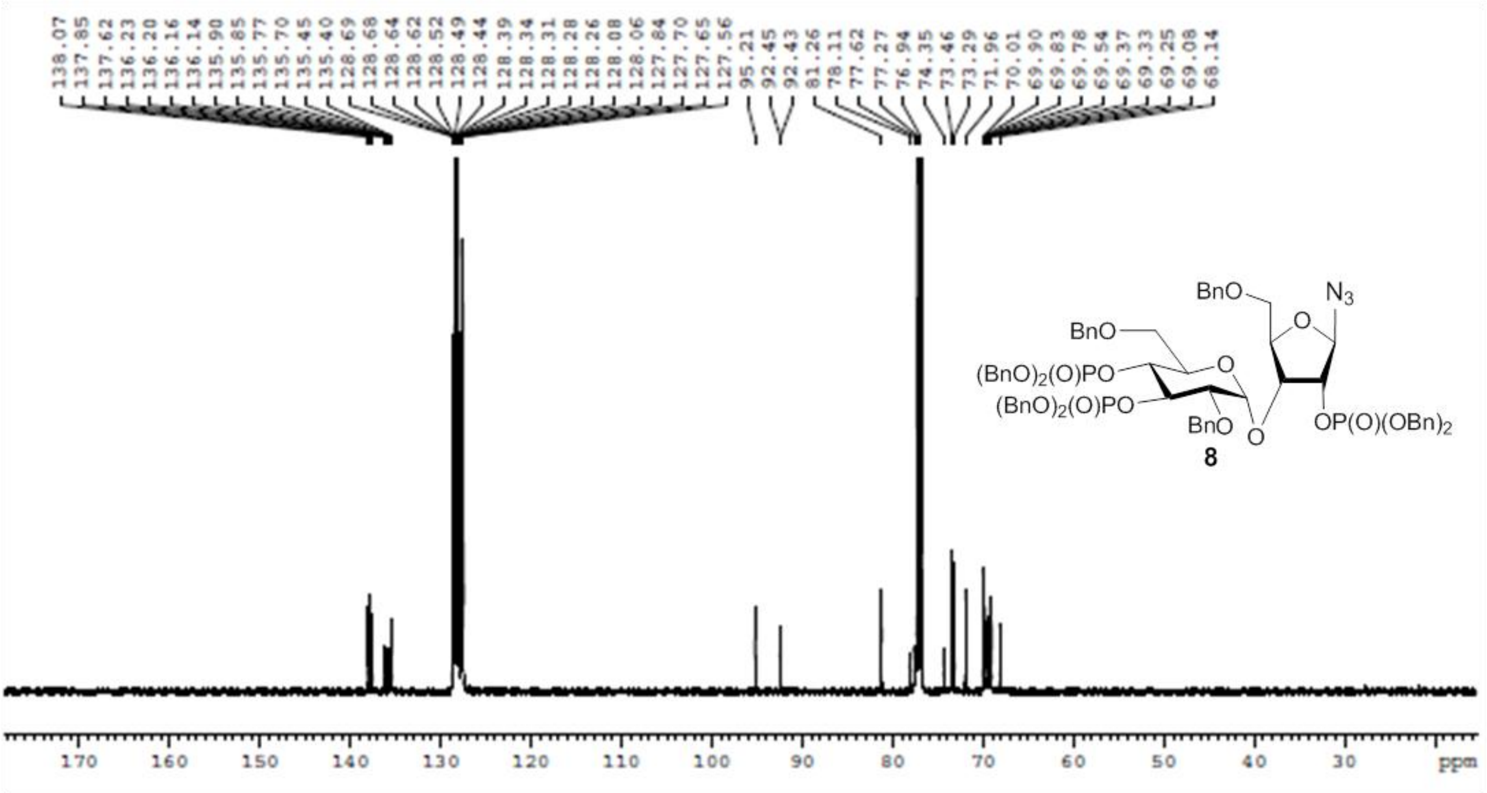

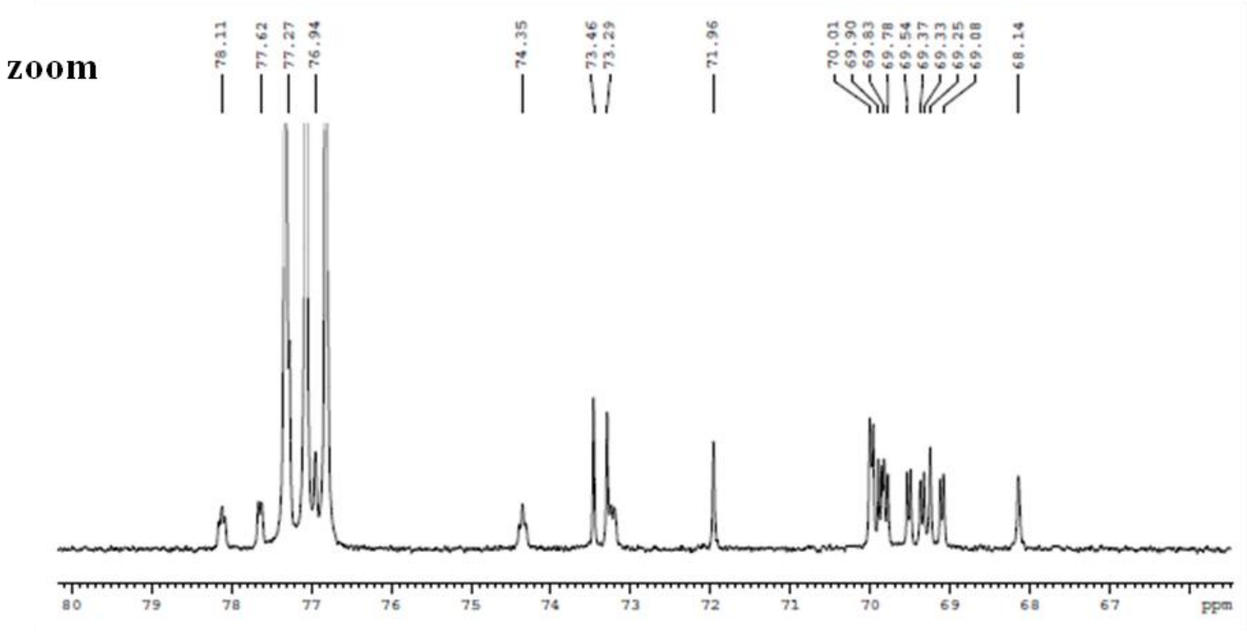

DEPT of 8 in $CDCl_3$

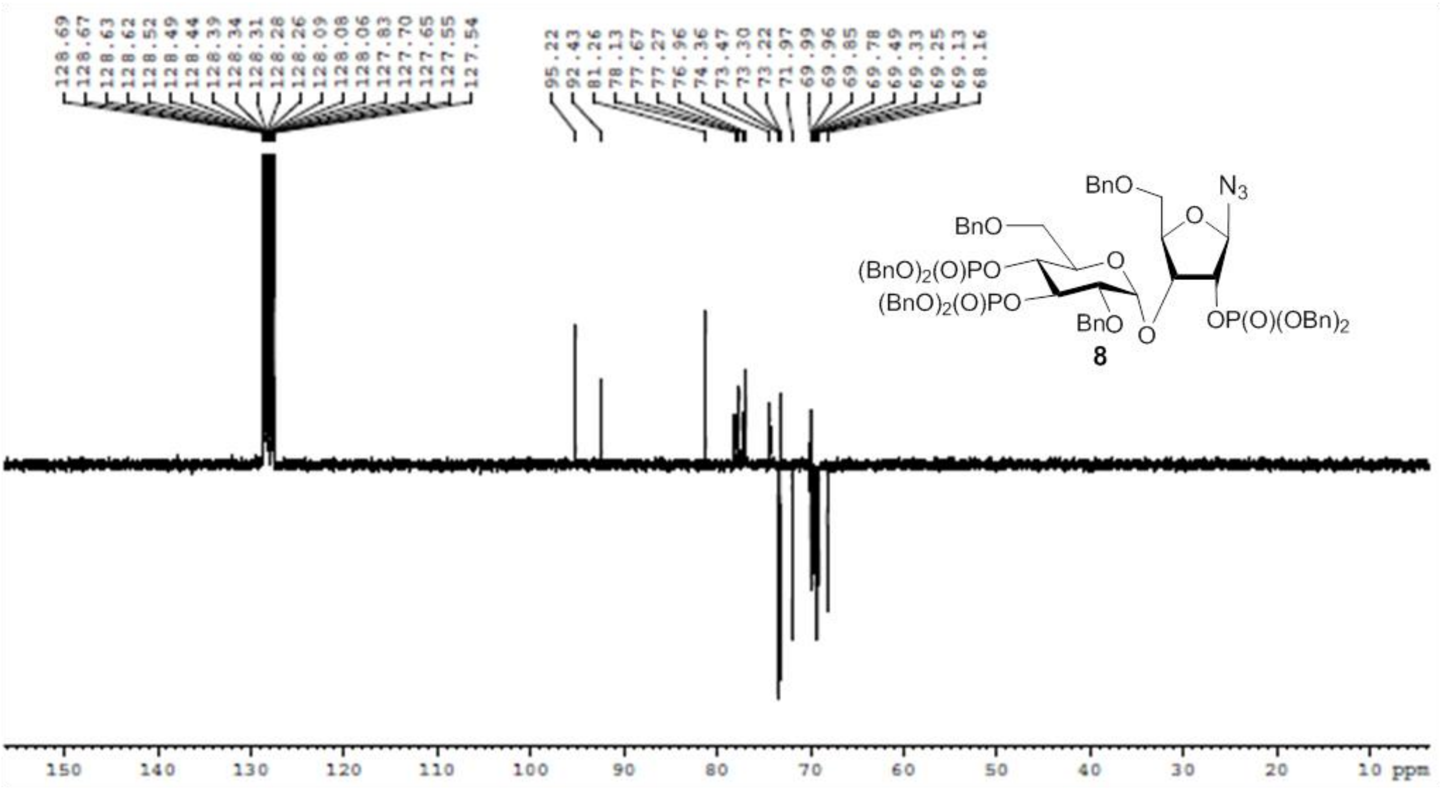

^{31}P NMR of 8 in $CDCl_3$

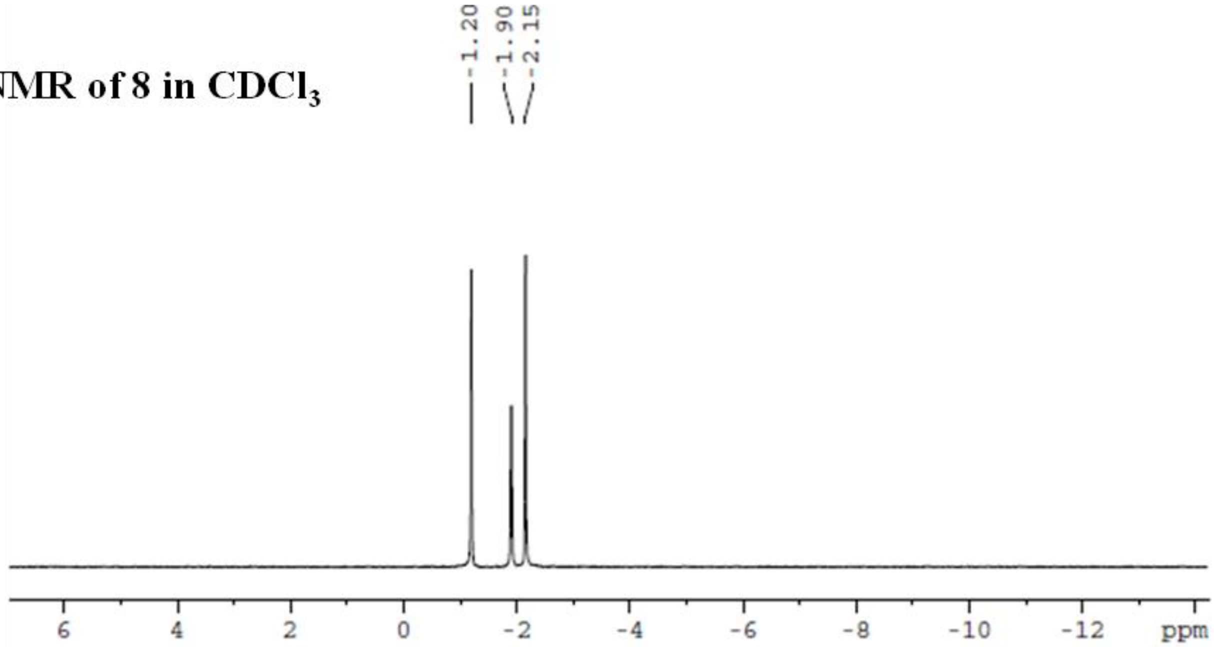

HMBC of 8 in $CDCl_3$

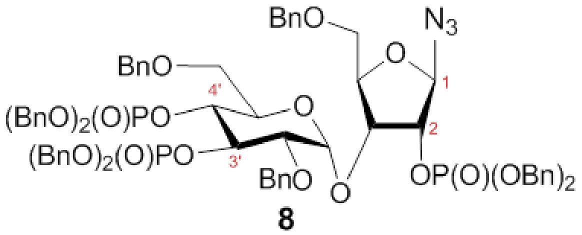

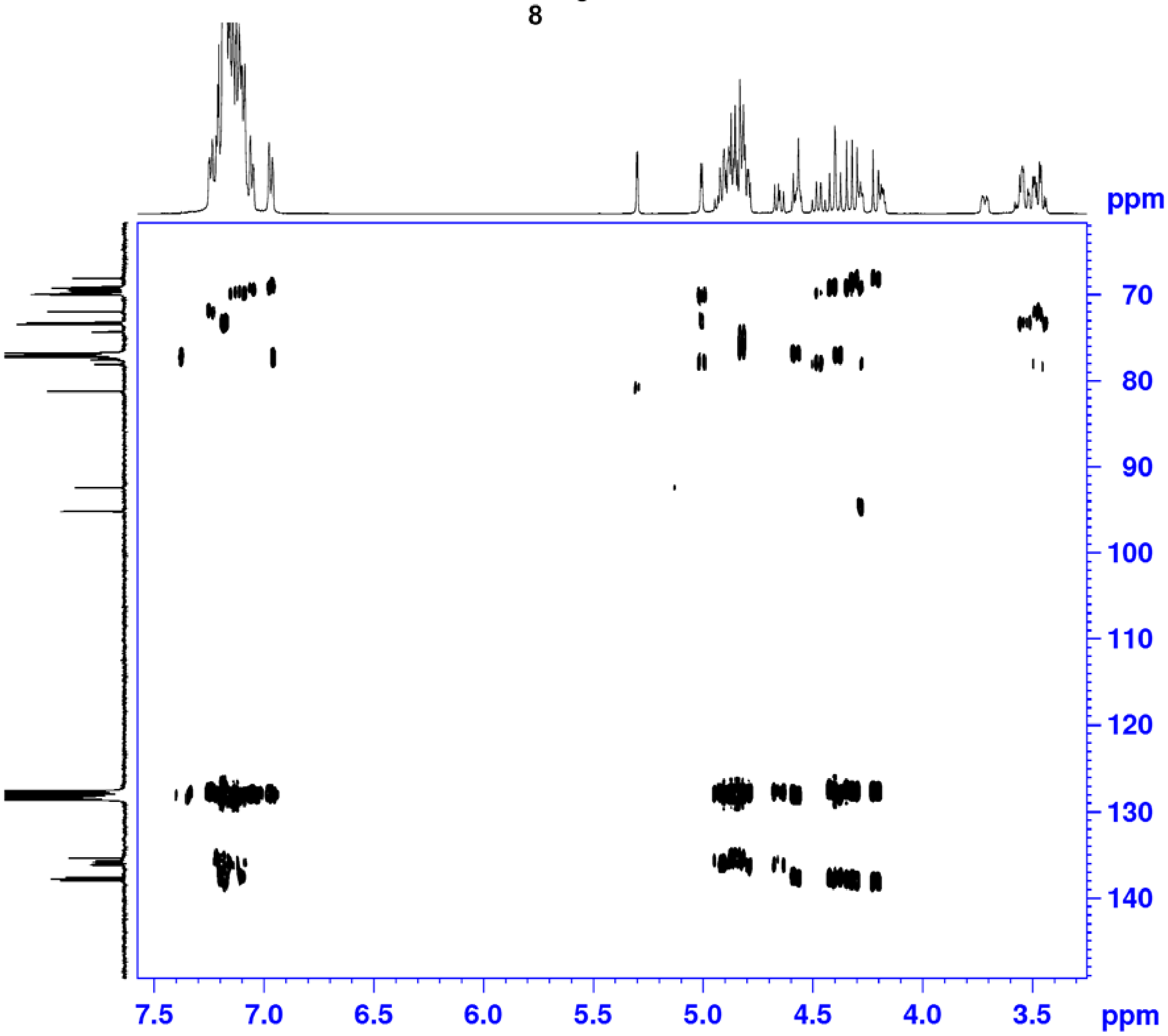

HMQC of 8 in $CDCl_3$

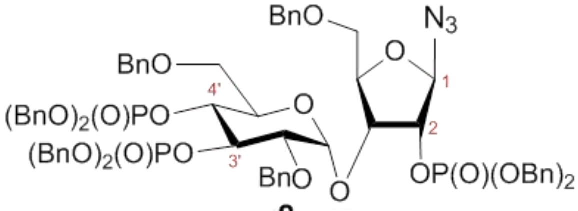

8

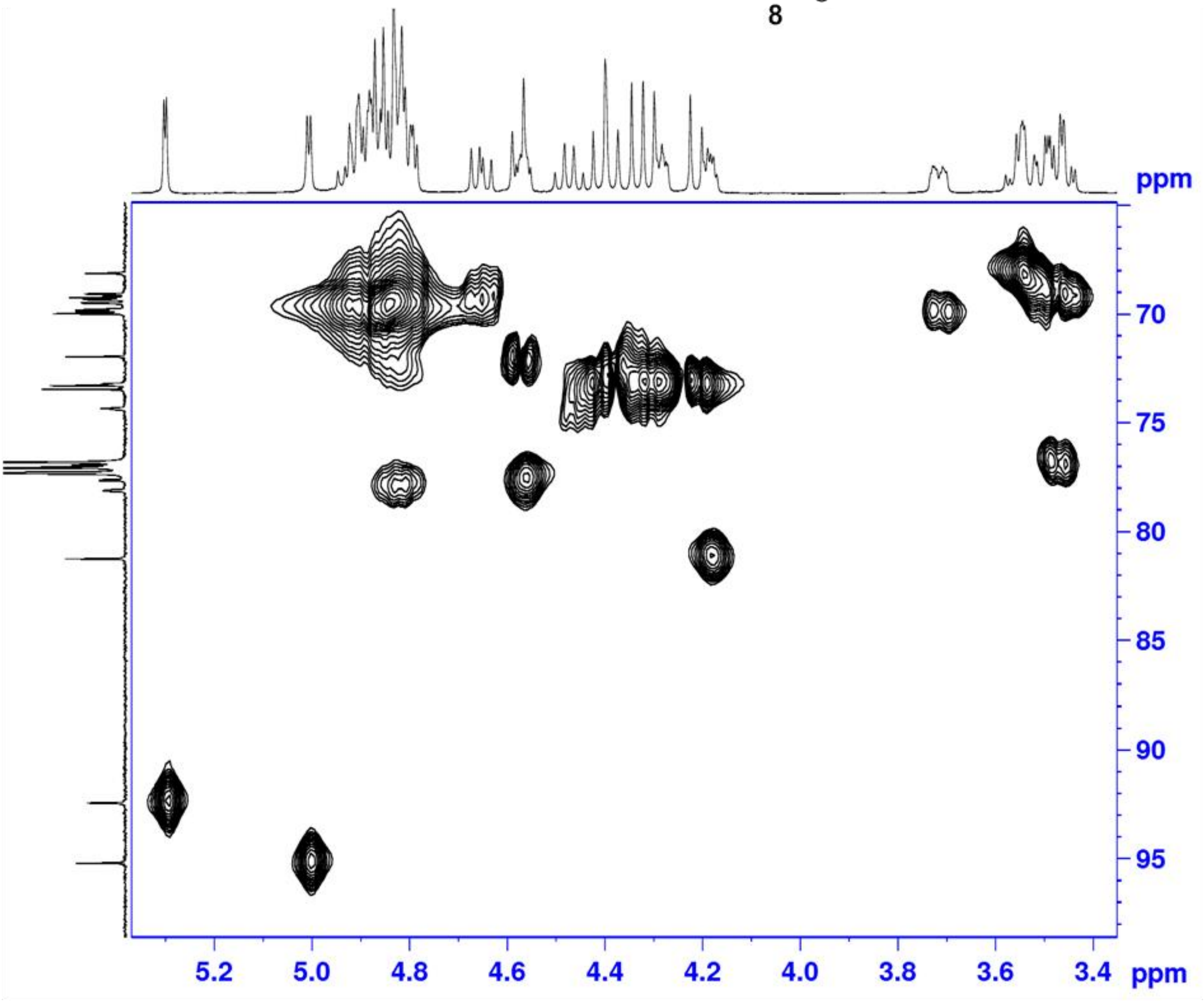

^{1}H NMR of 9a in CDCl$_3$

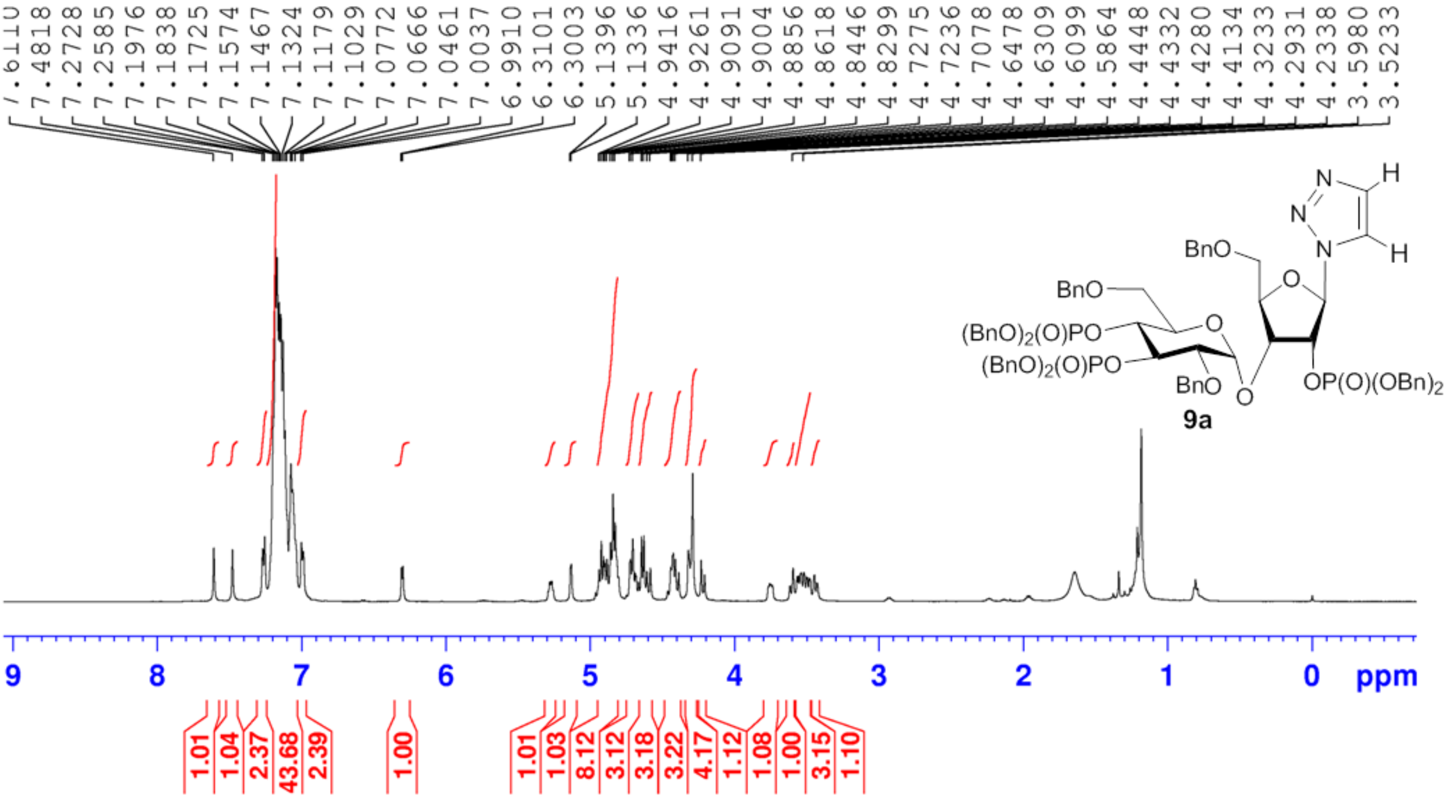

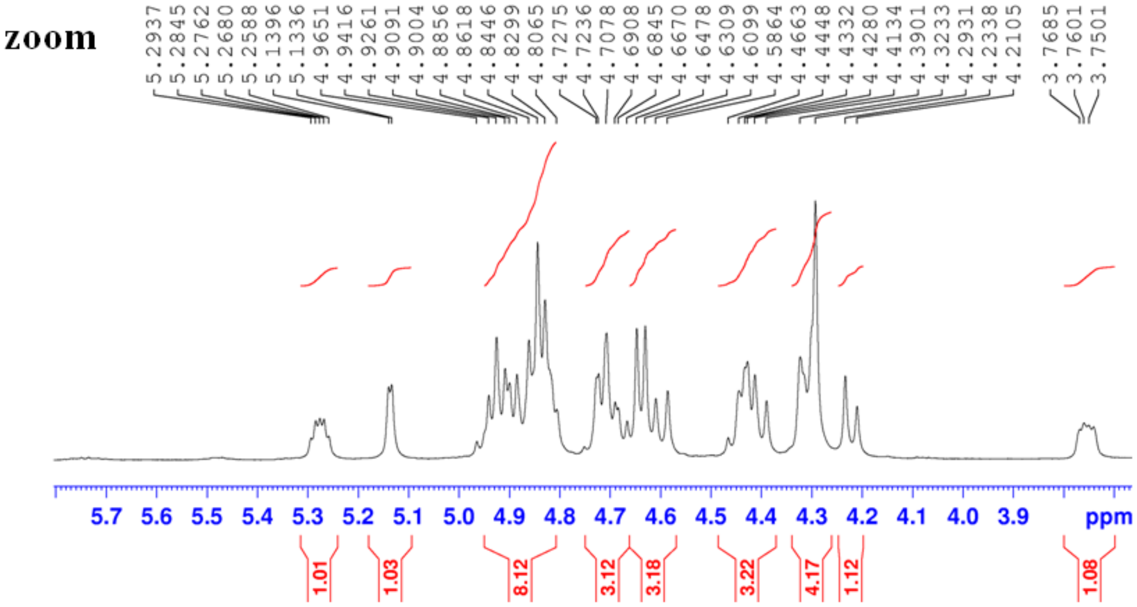

COSY of 9a in $CDCl_3$

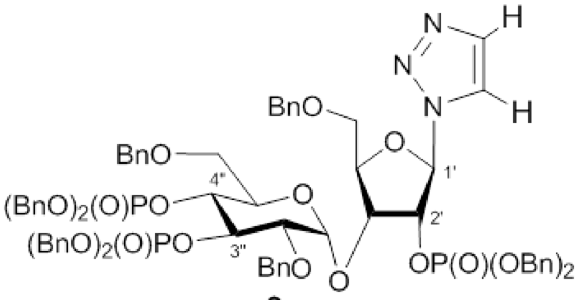

9a

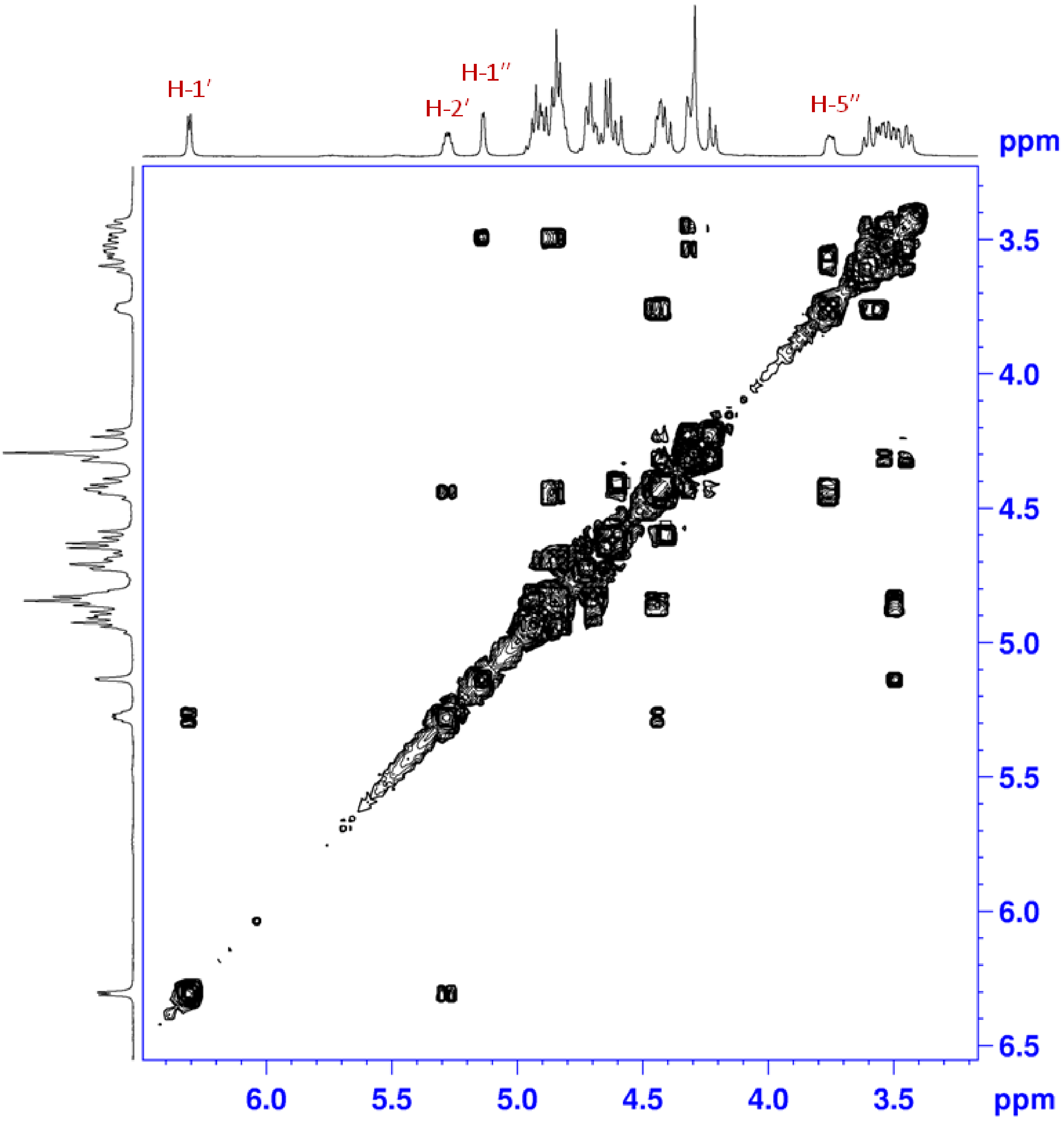

^{13}C of 9a in $CDCl_3$

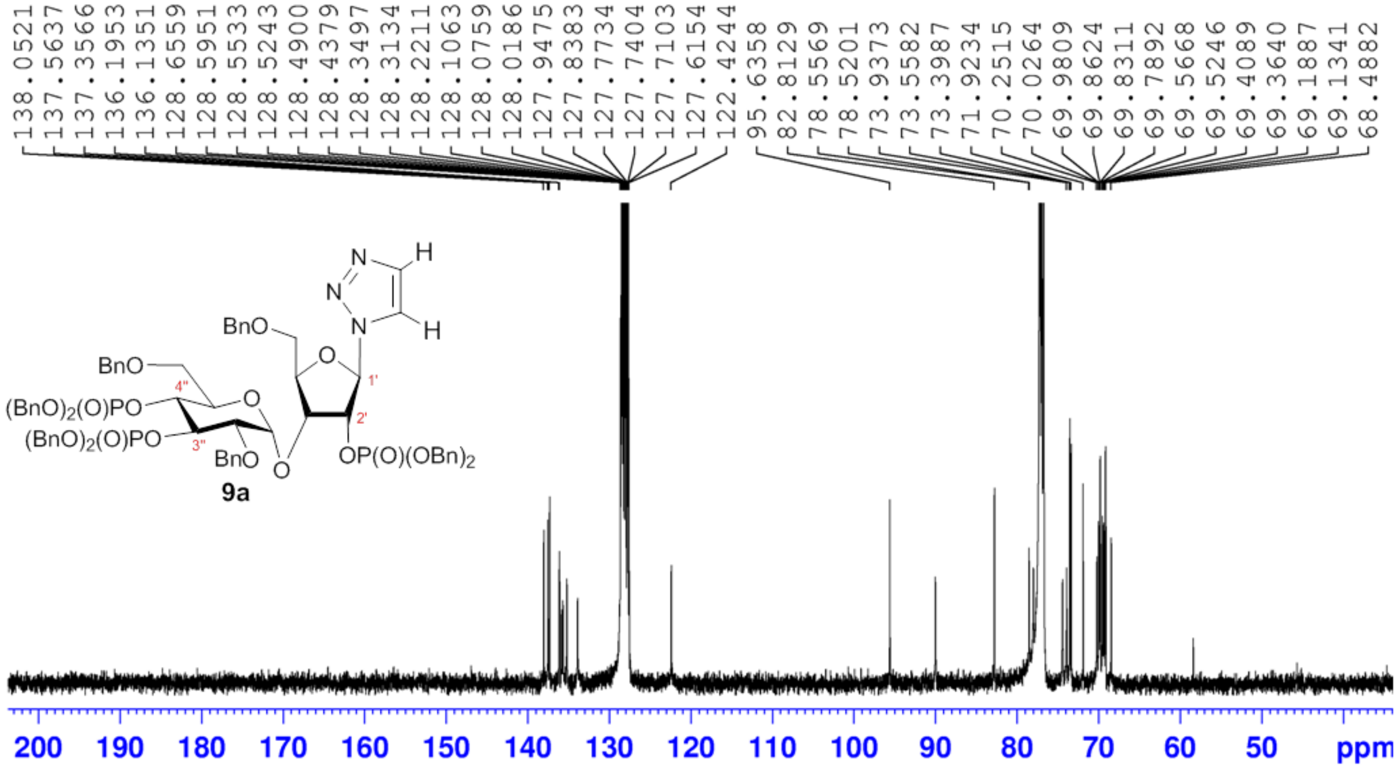

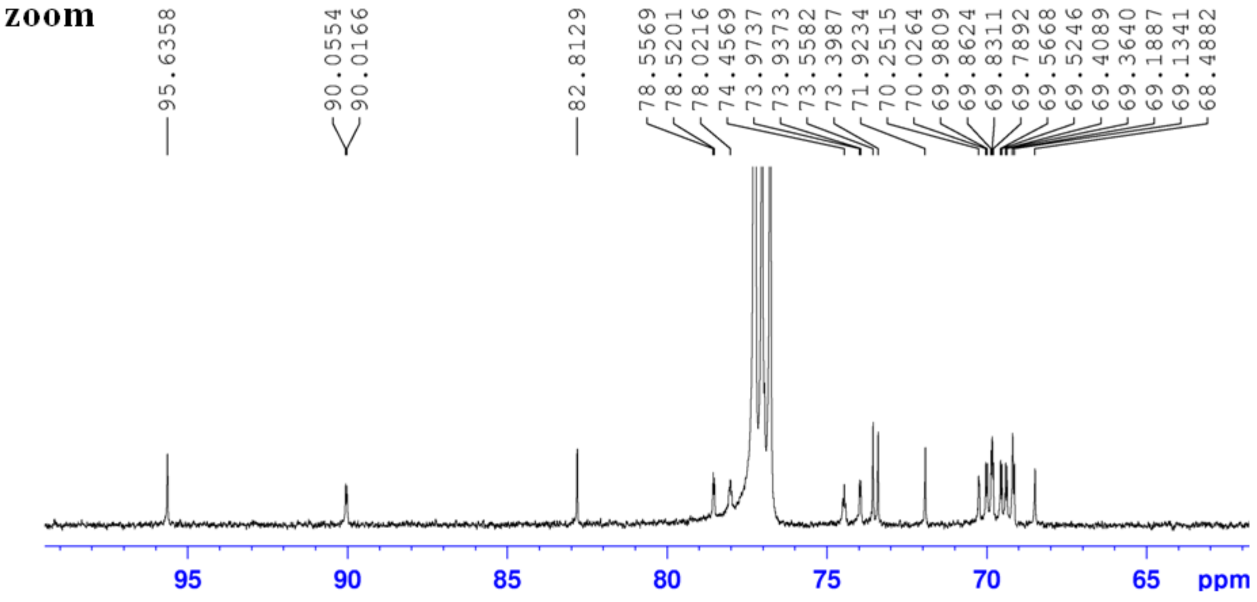

DEPT of 9a in $CDCl_3$

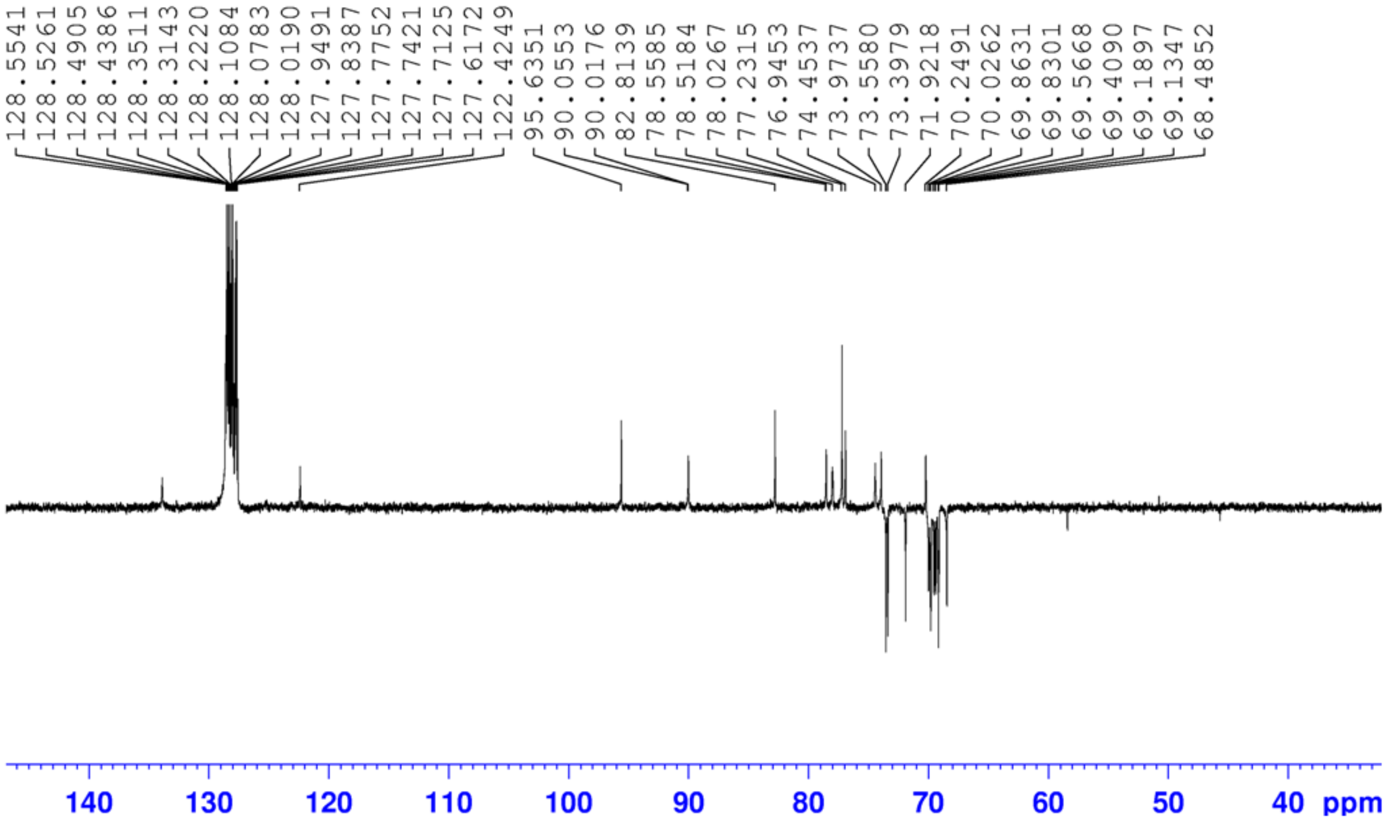

^{31}P NMR of 9a in $CDCl_3$

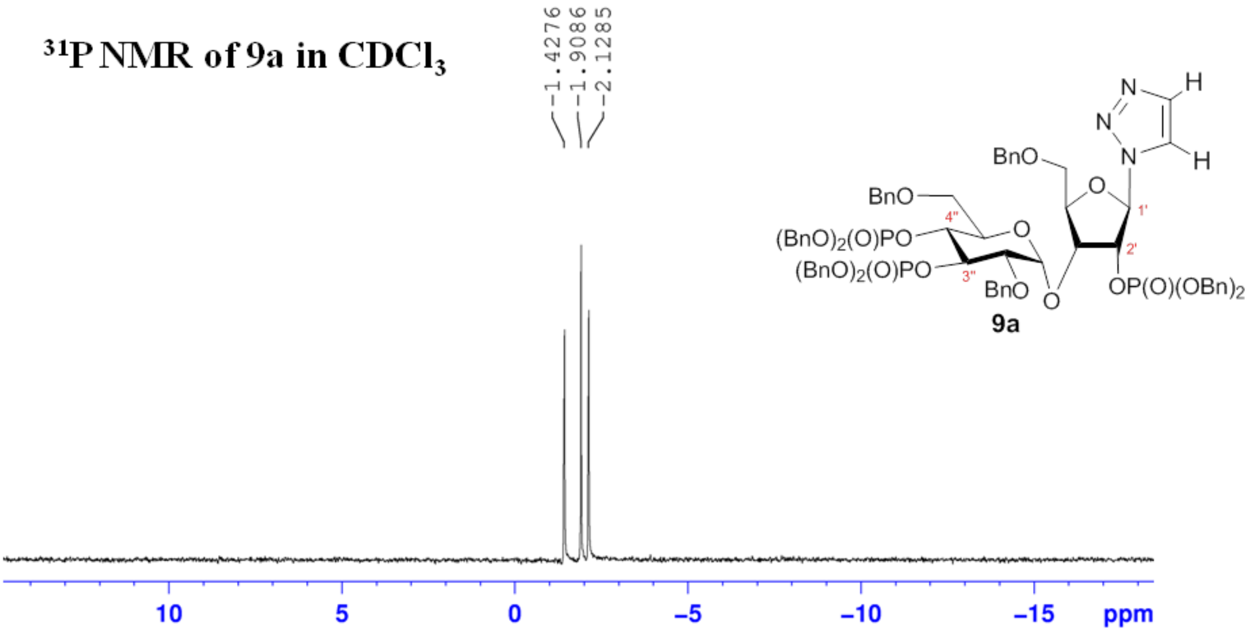

9a

HMBC of 9a in $CDCl_3$

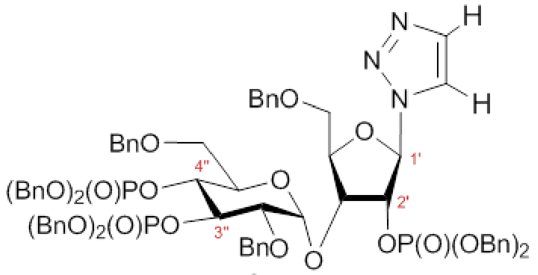

9a

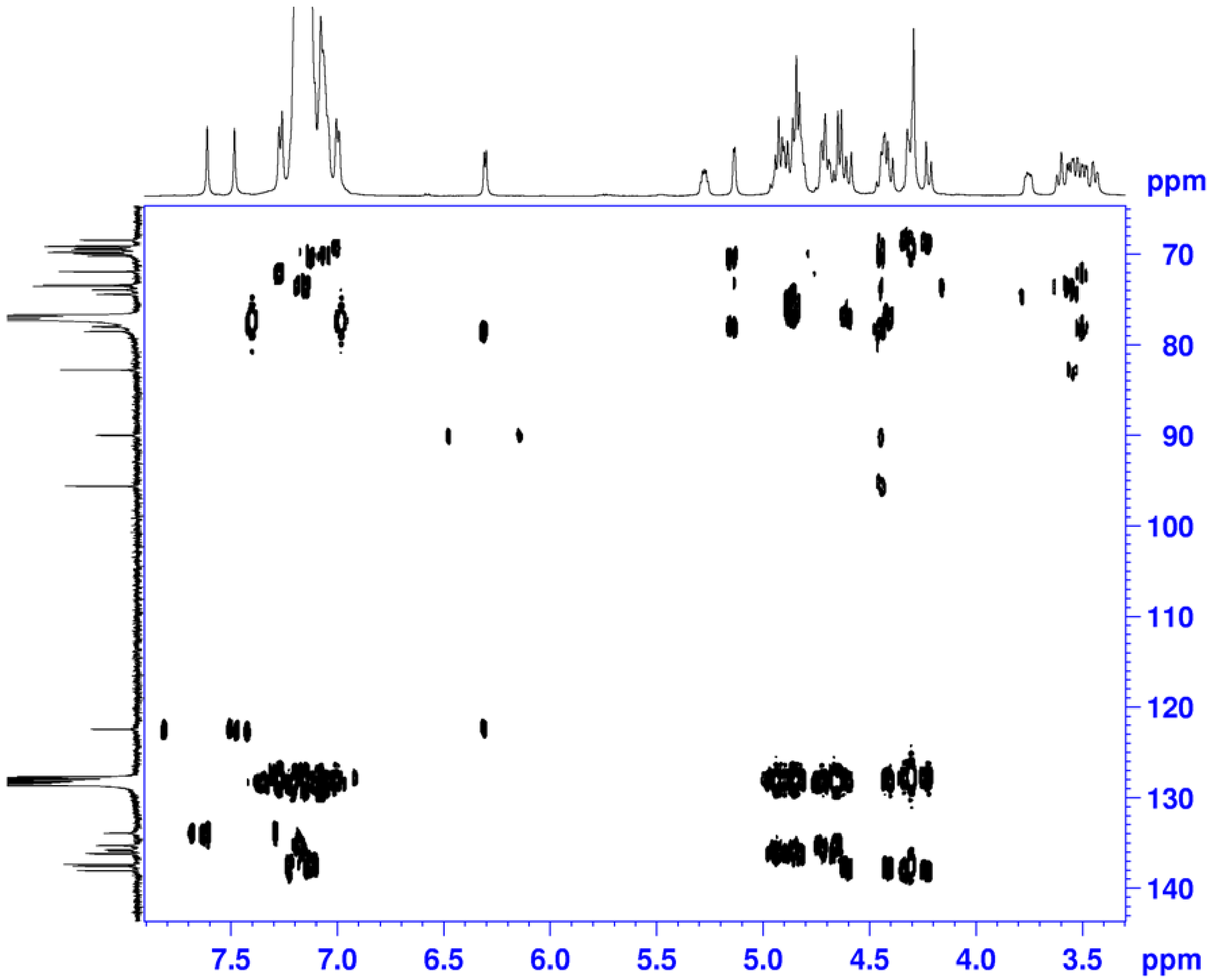

HMQC of 9a in $CDCl_3$

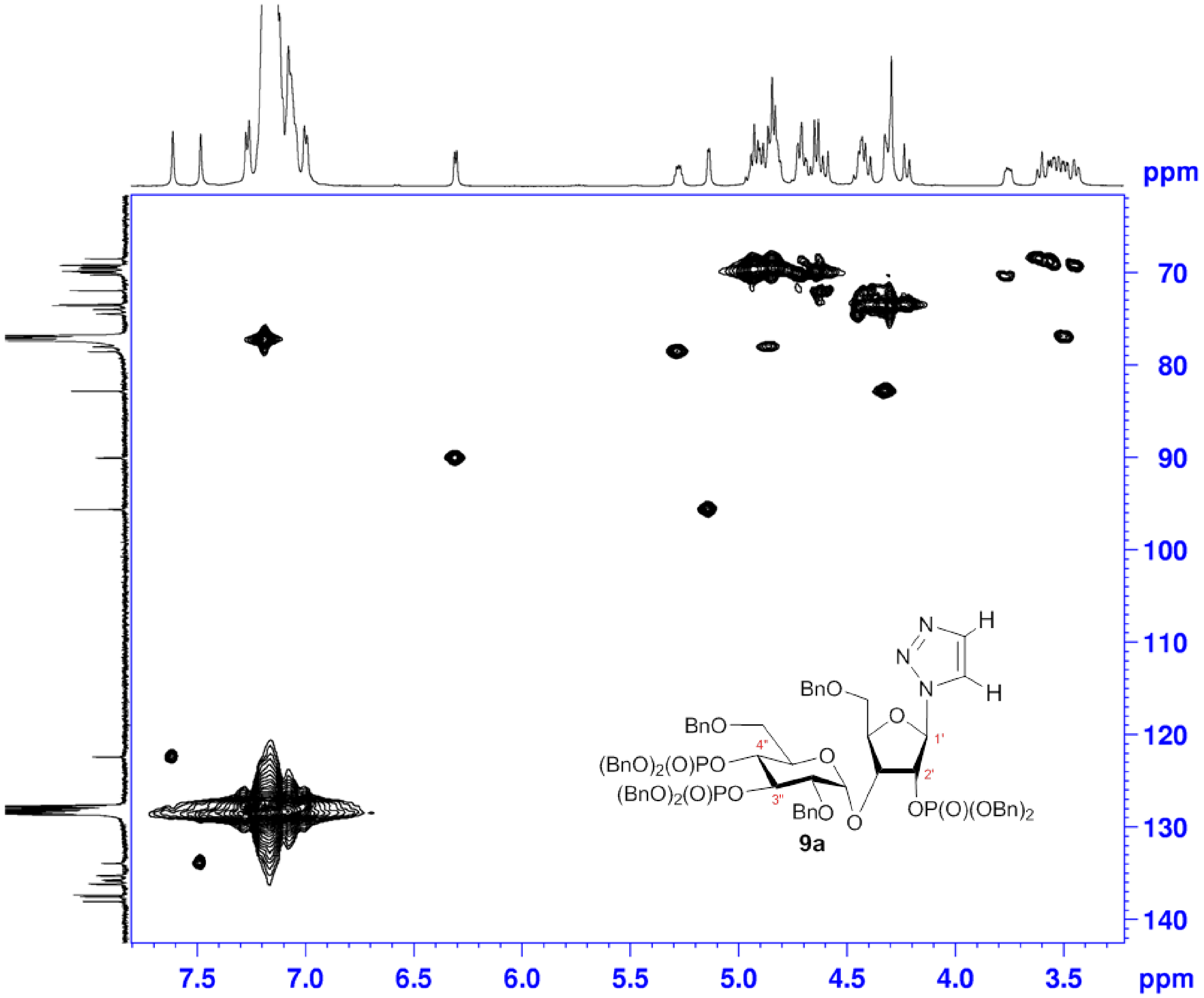

^{1}H NMR of 10a in D_2O

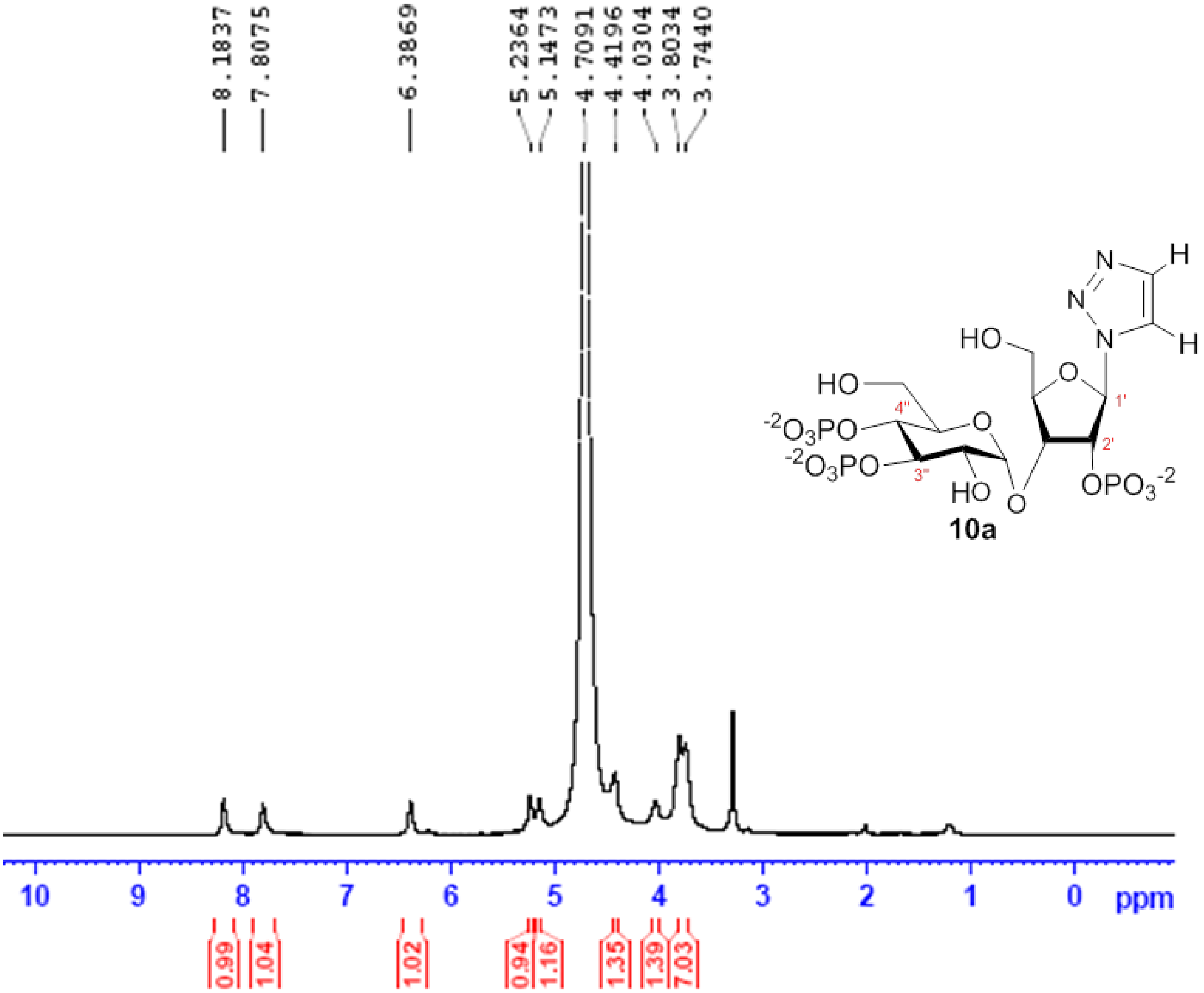

COSY of 10a in D_2O

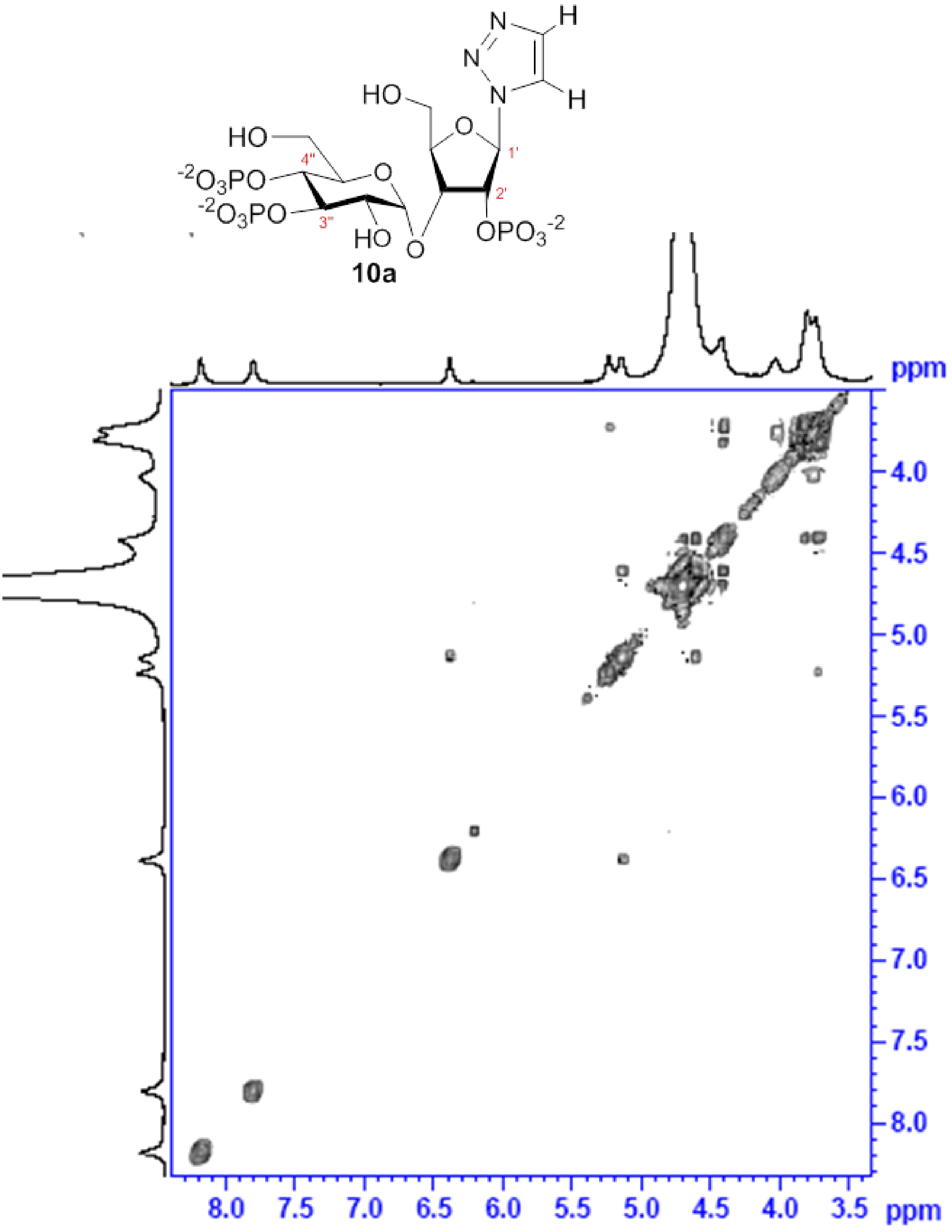

^{13}C NMR of 10a in D_2O

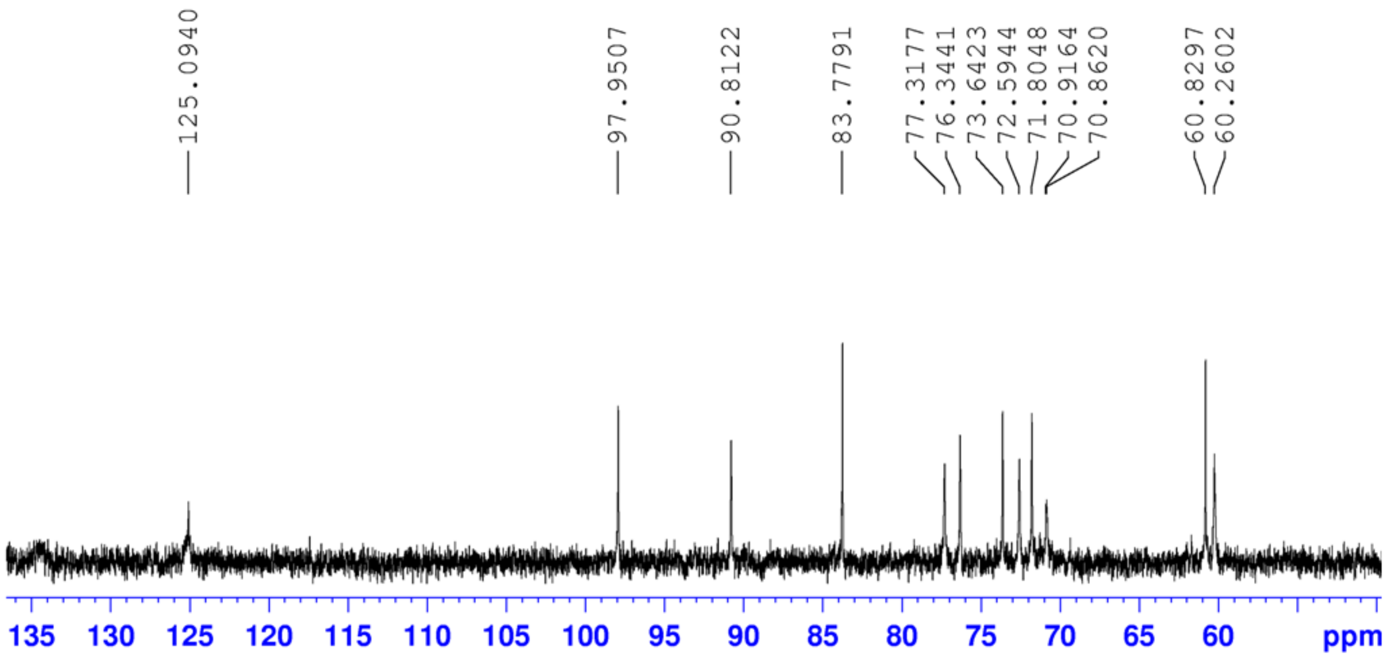

DEPT of 10a in D_2O

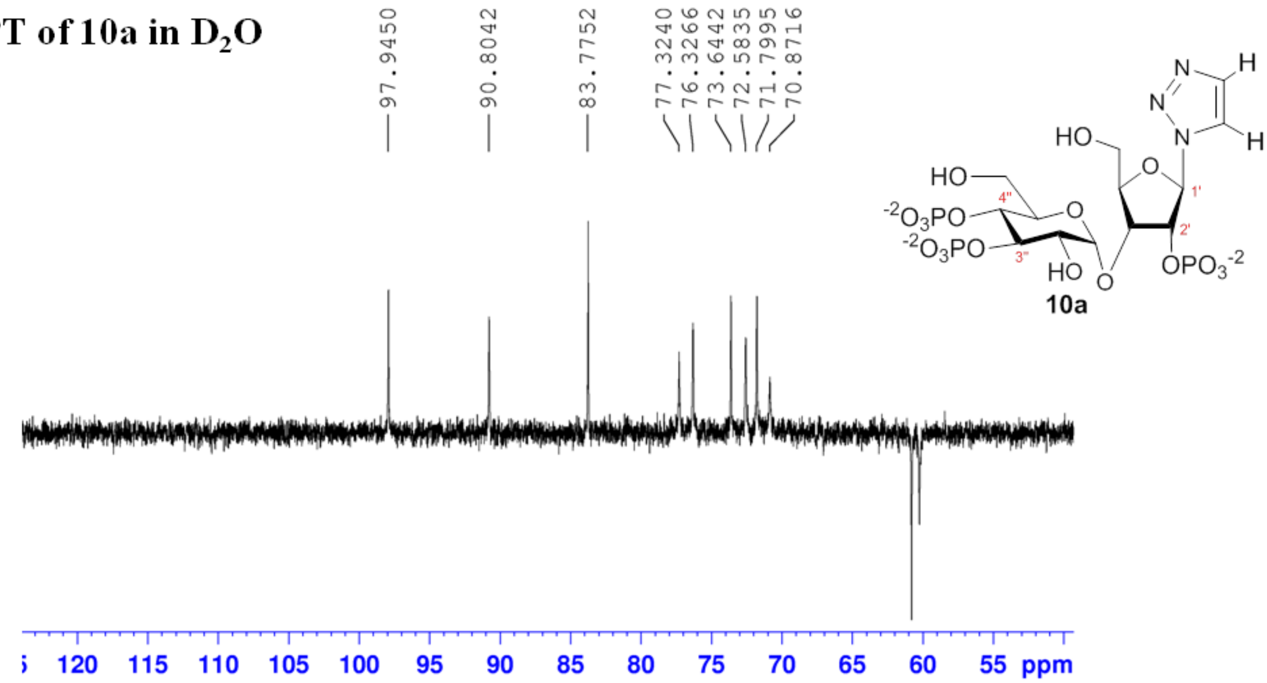

10a

^{31}P NMR of 10a in D_2O

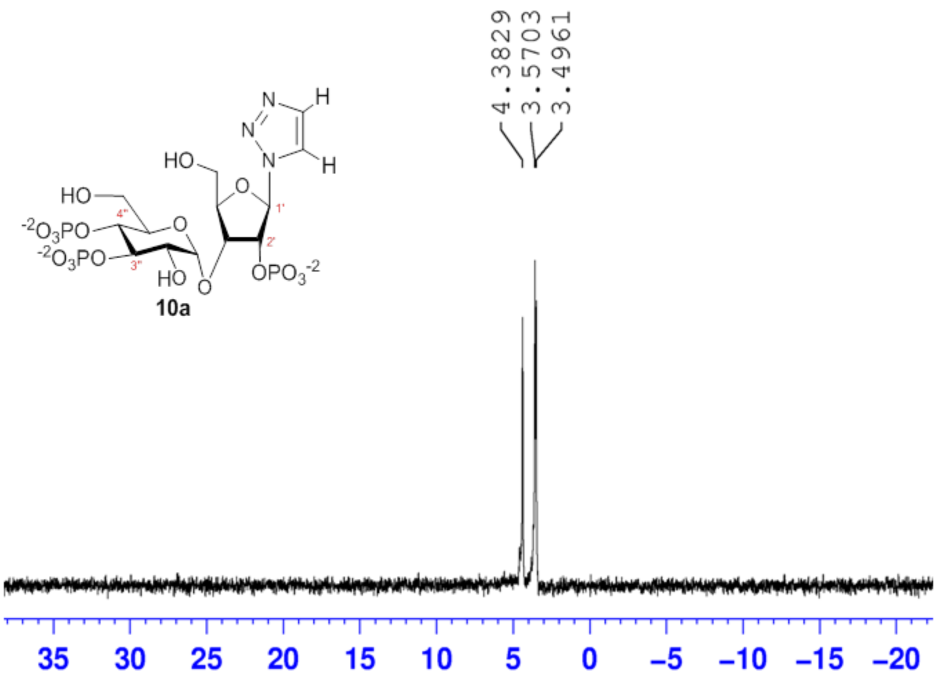

HMQC of 10a in D_2O

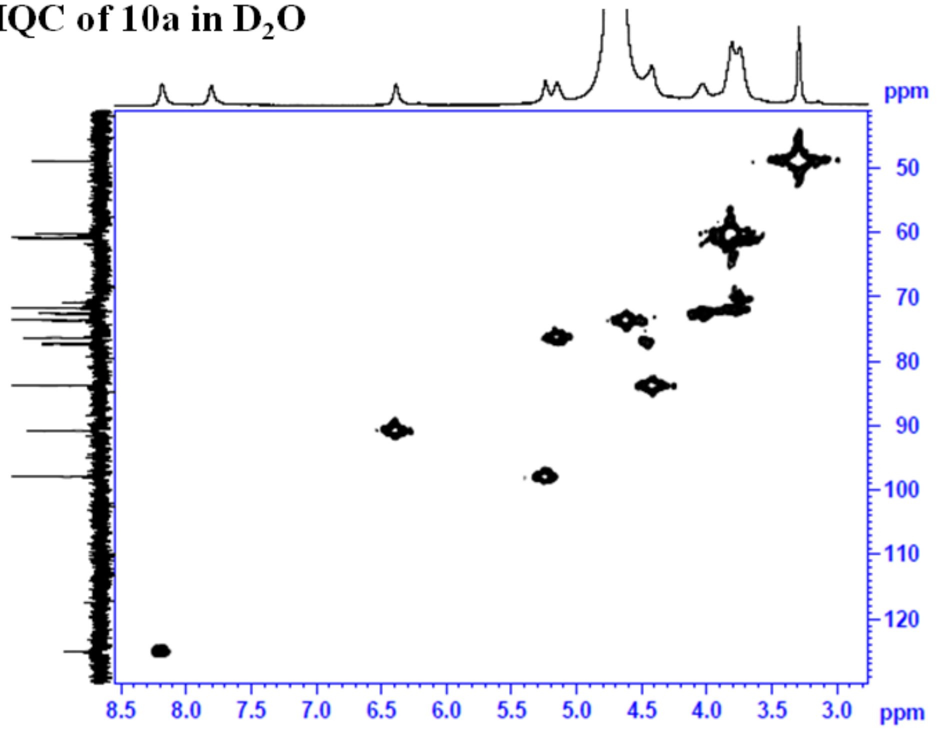

^{1}H NMR of 9b in $CDCl_3$

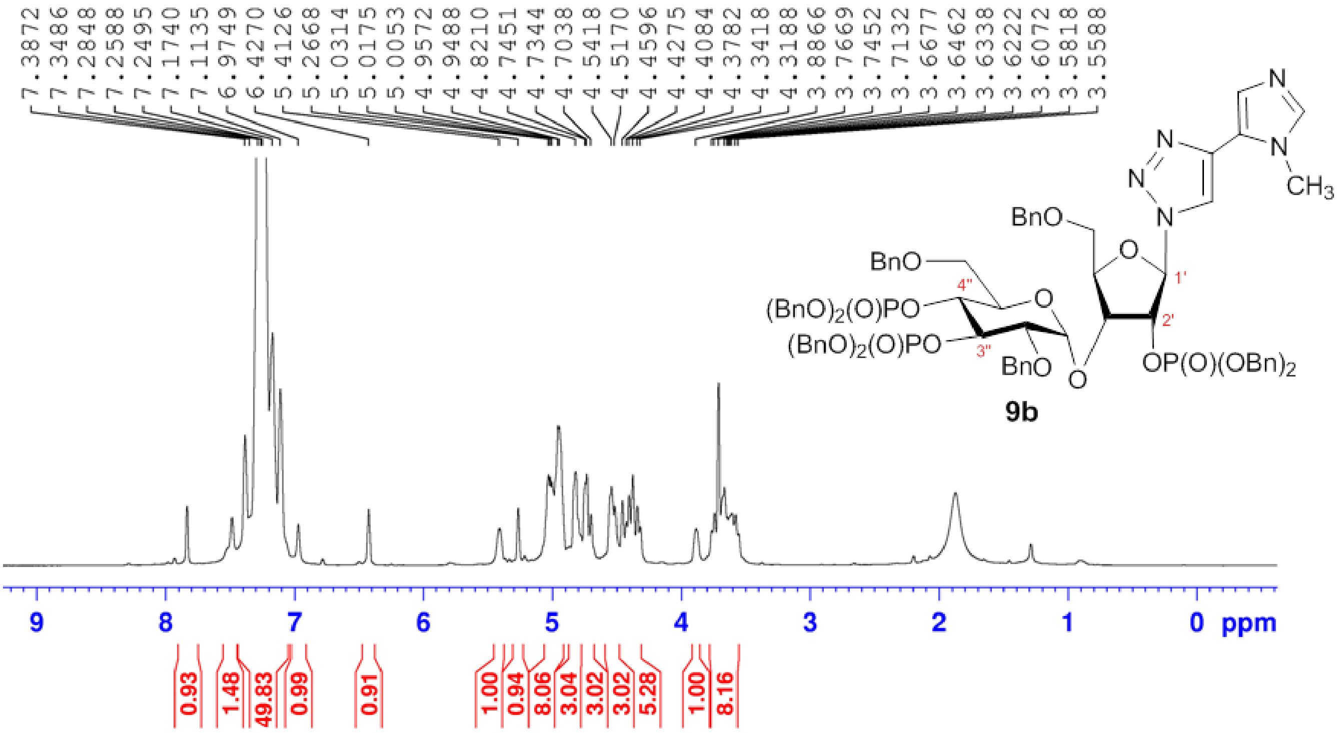

zoom

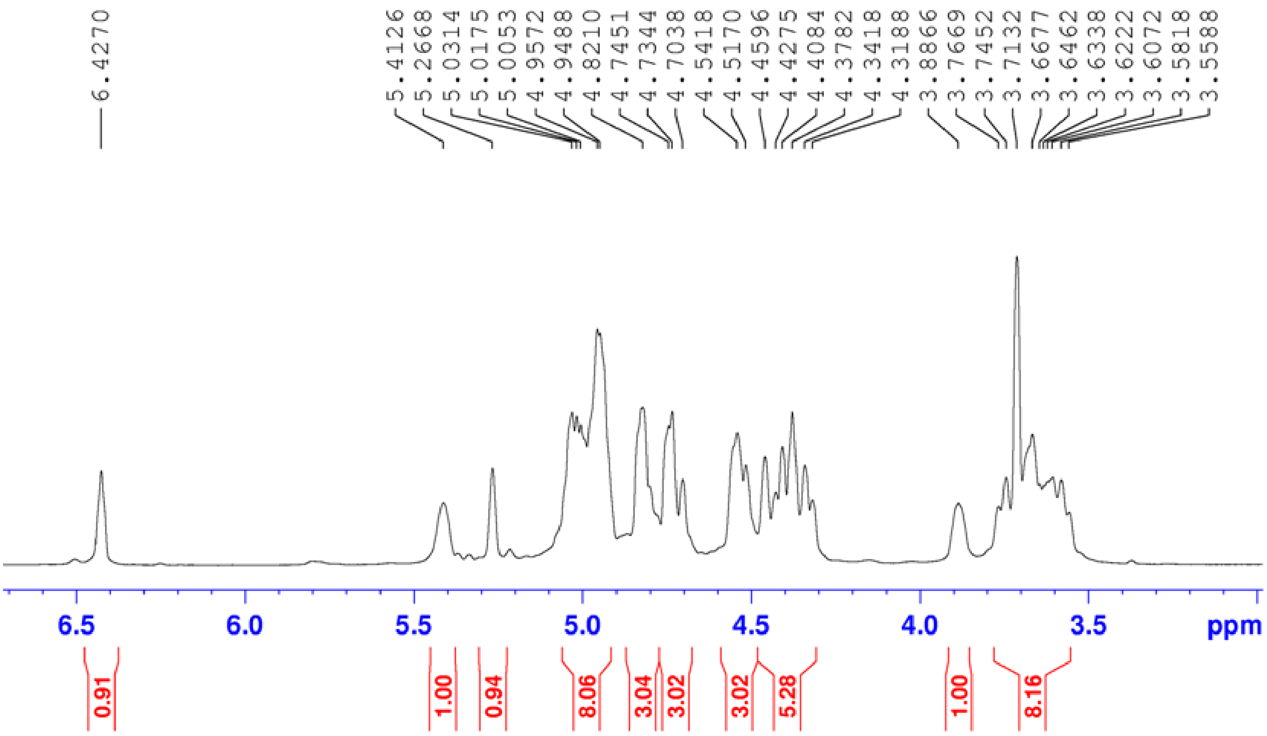

COSY of 9b in $CDCl_3$

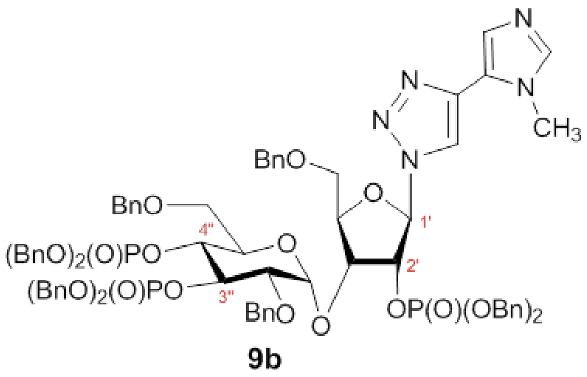

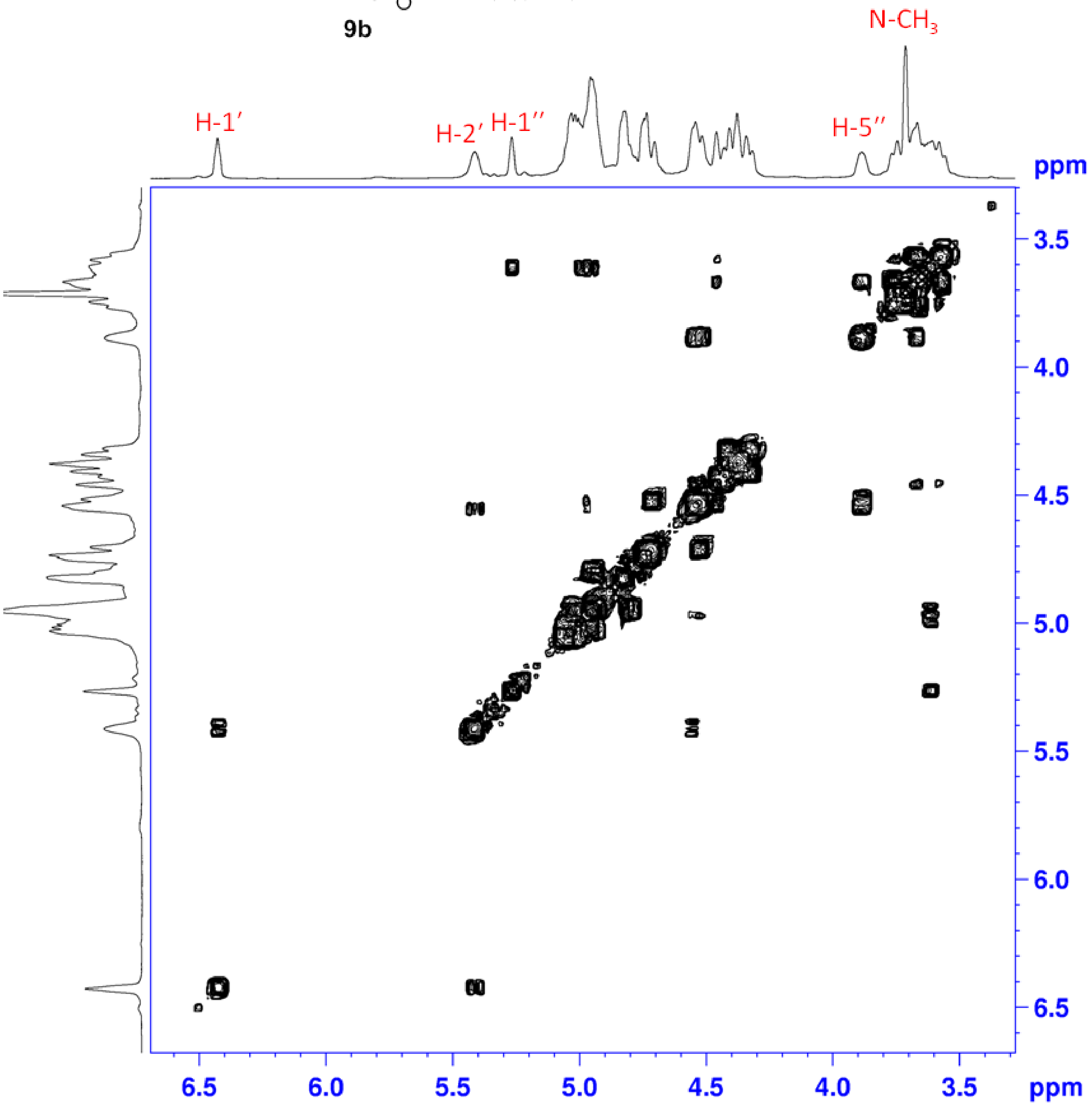

^{13}C NMR of 9b in $CDCl_3$

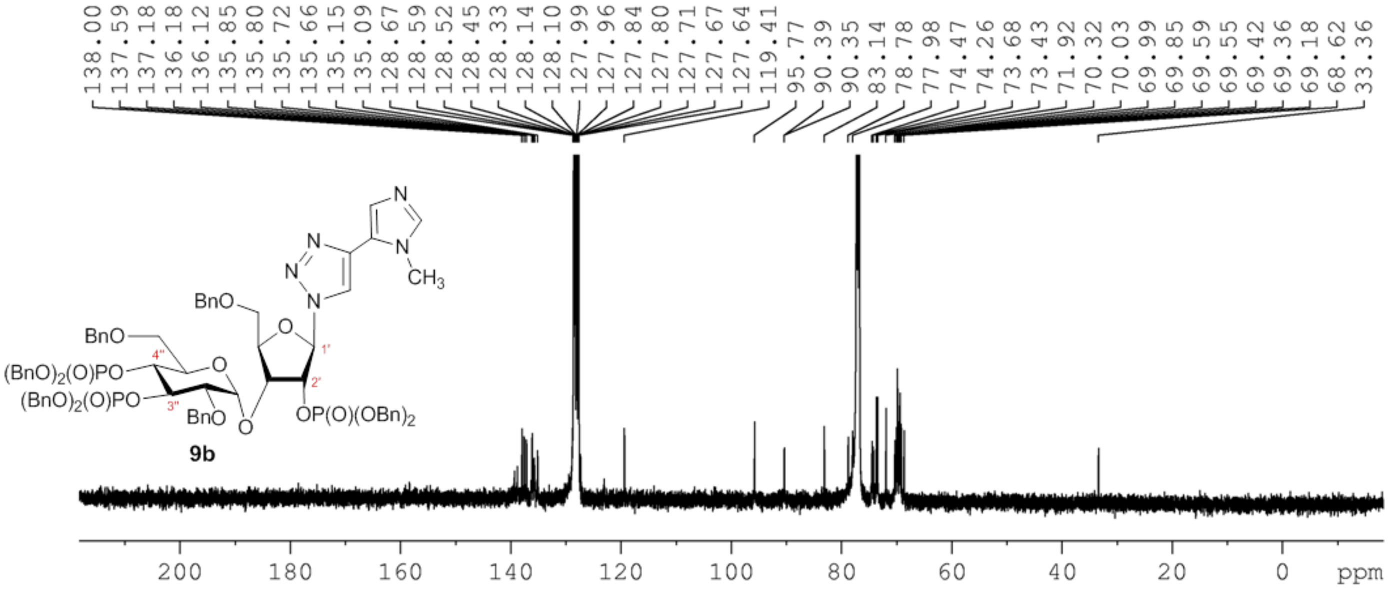

zoom

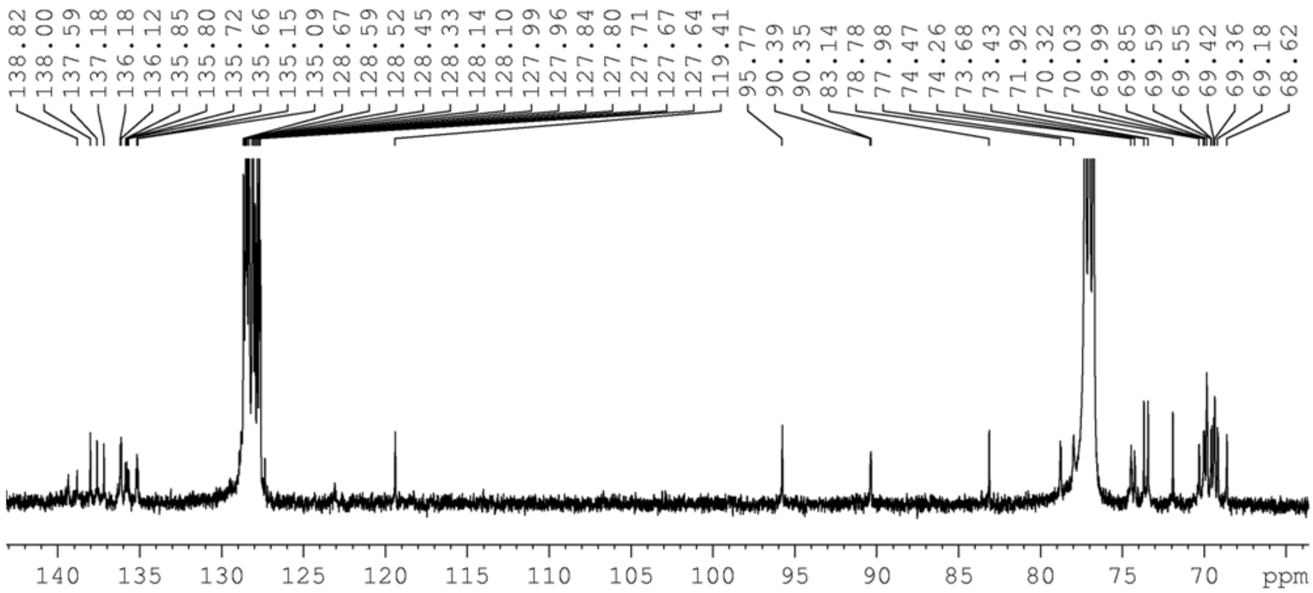

DEPT of 9b in $CDCl_3$

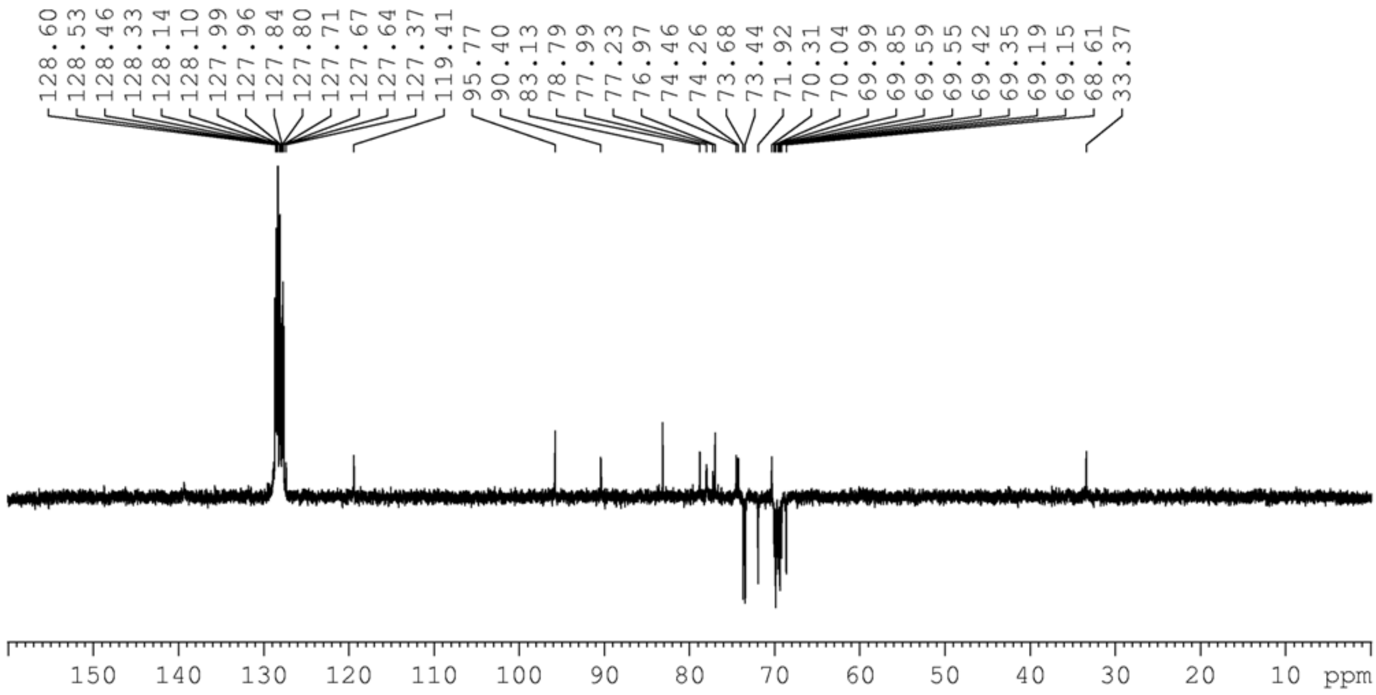

^{31}P NMR of 9b in $CDCl_3$

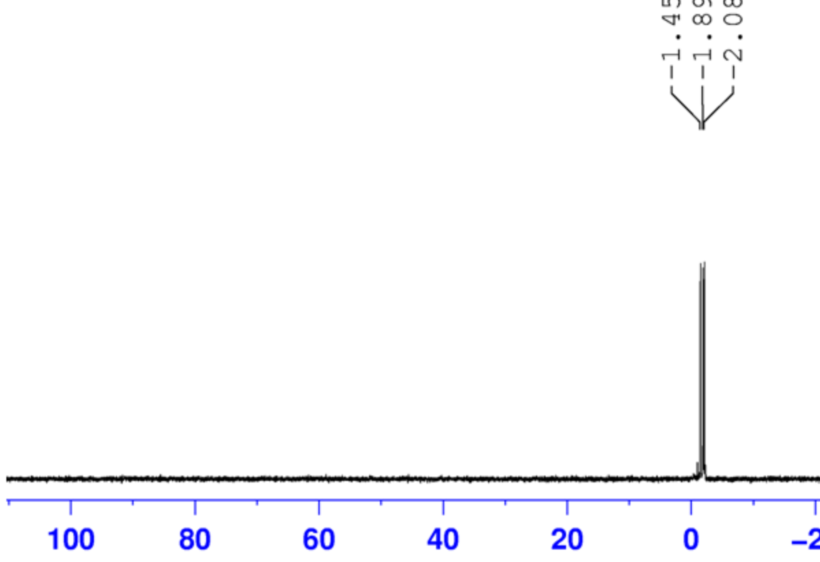

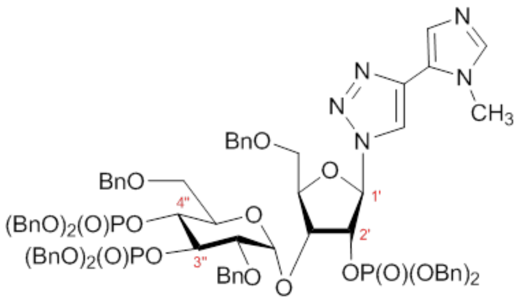

9b

HMBC of 9b in $CDCl_3$

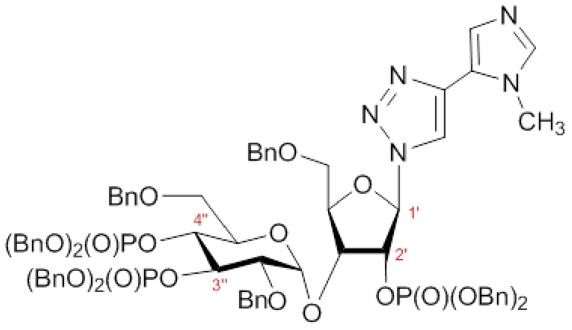

9b

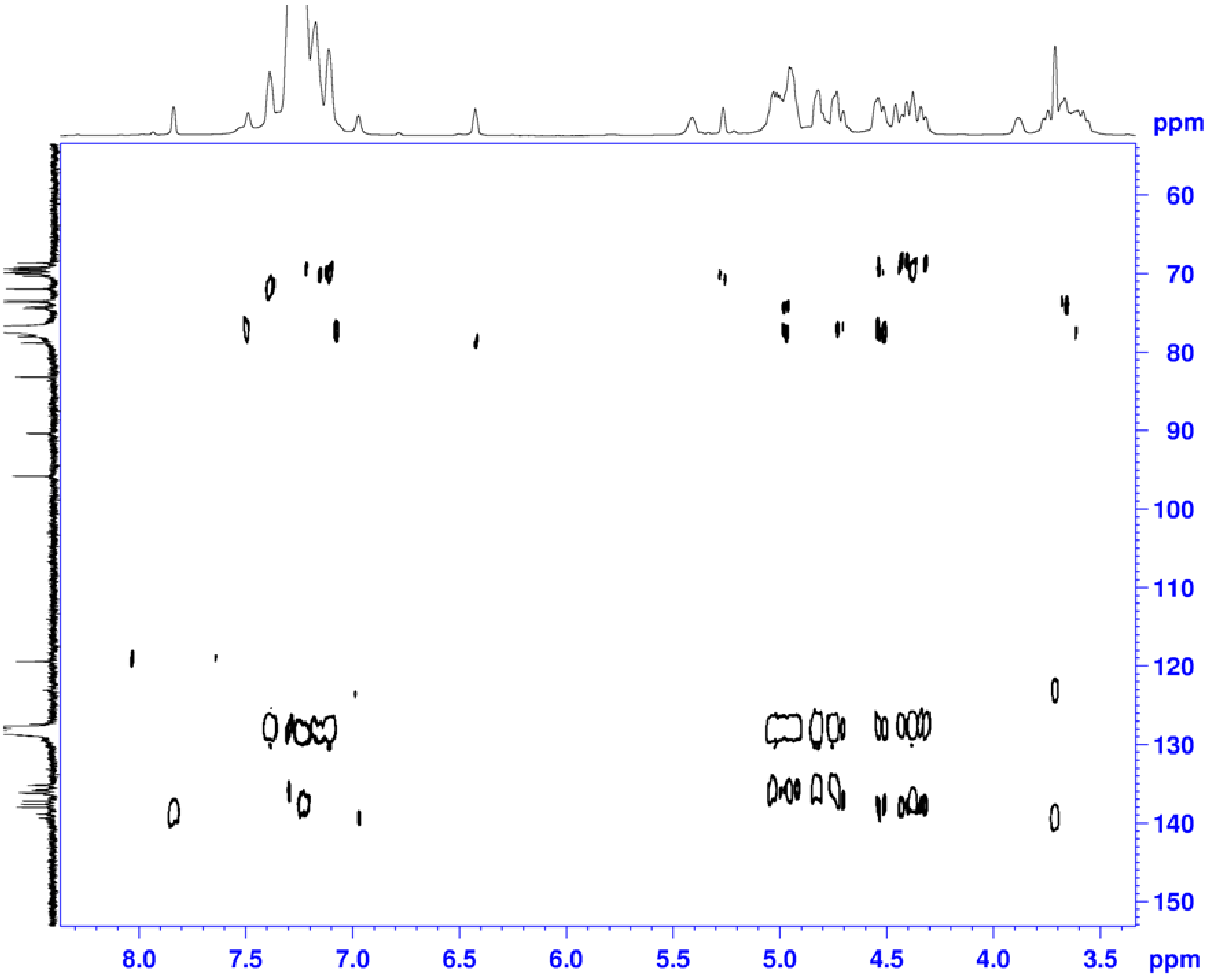

^{1}H NMR of 10b in D_2O

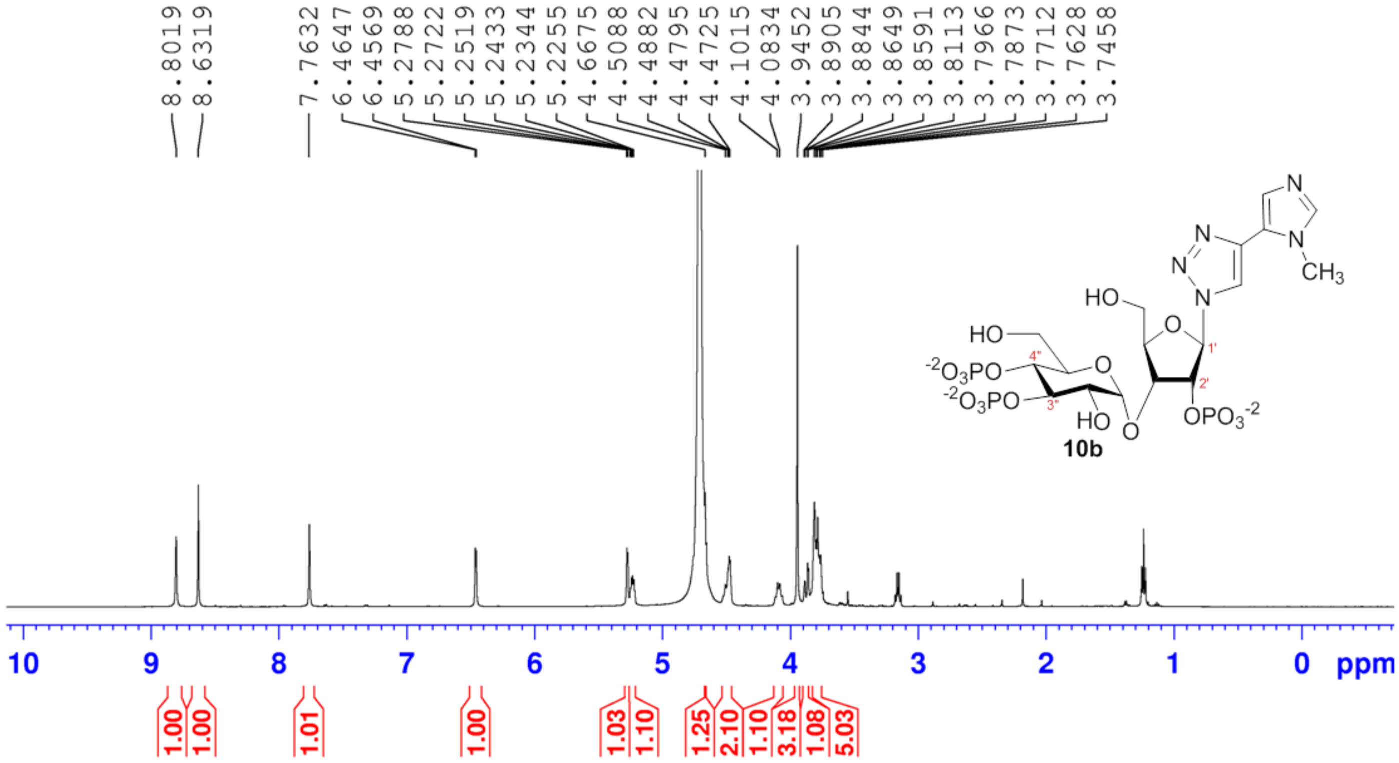

zoom

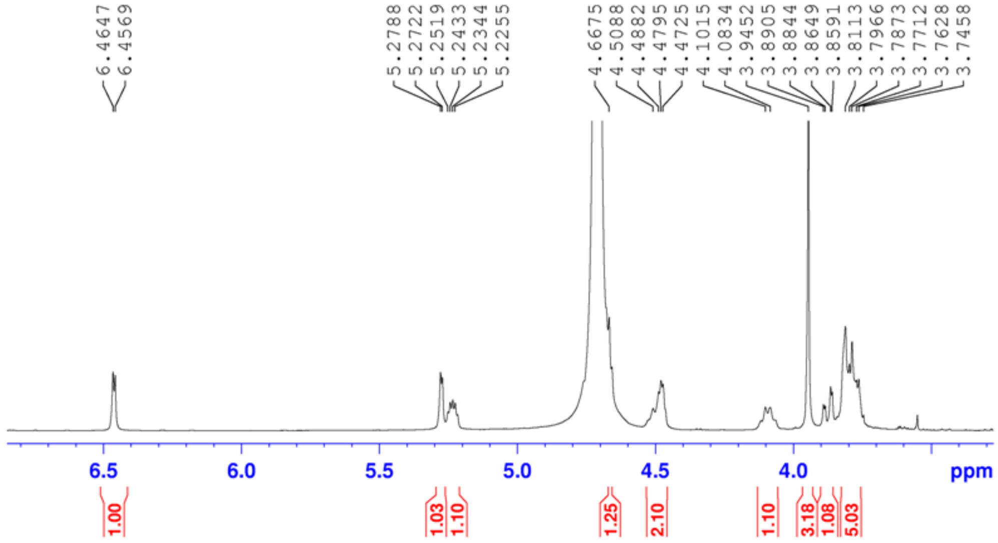

COSY of 10b in D_2O

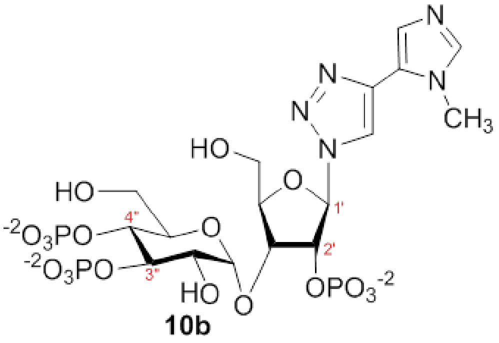

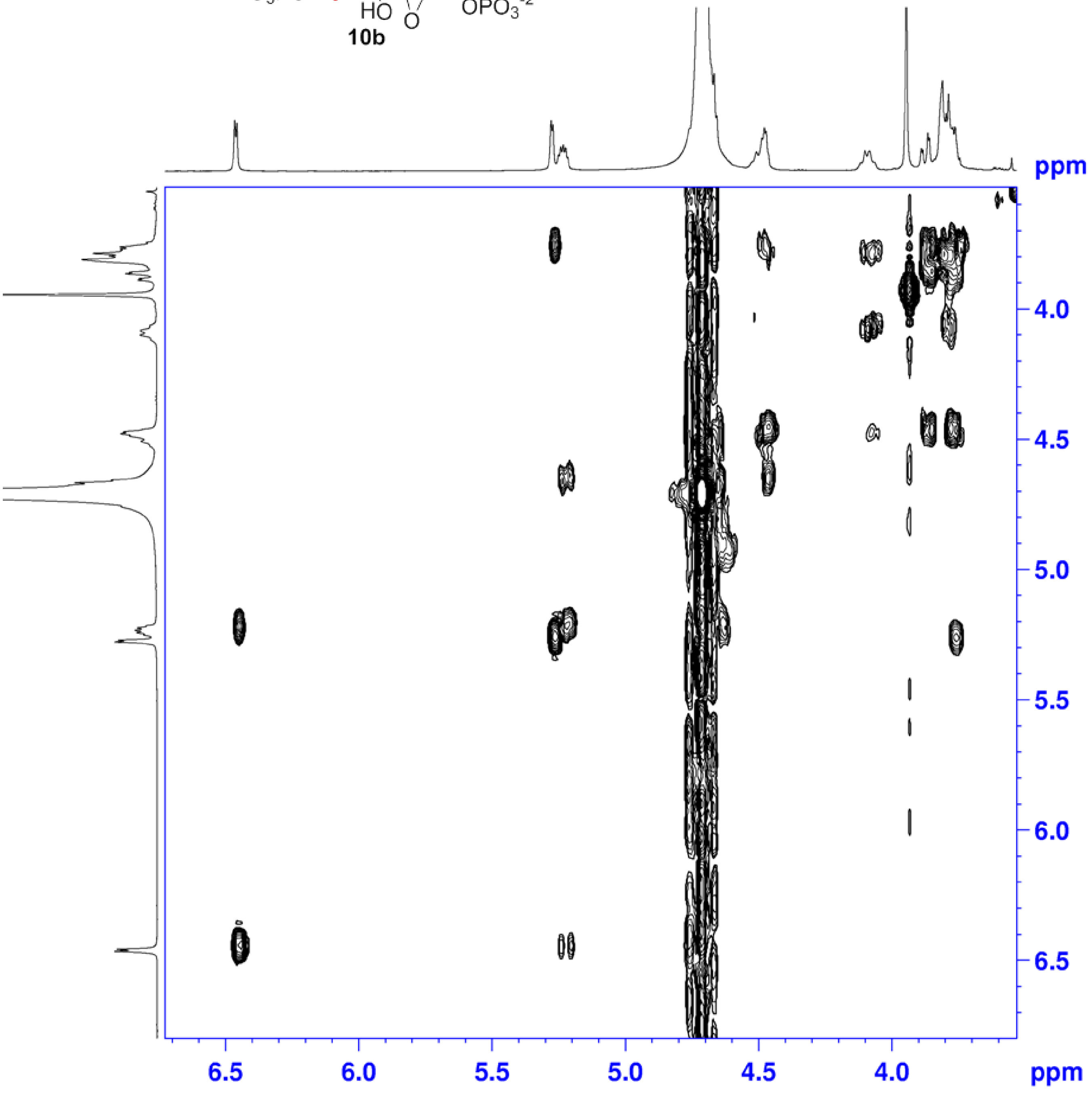

^{13}C NMR of 10b in D_2O

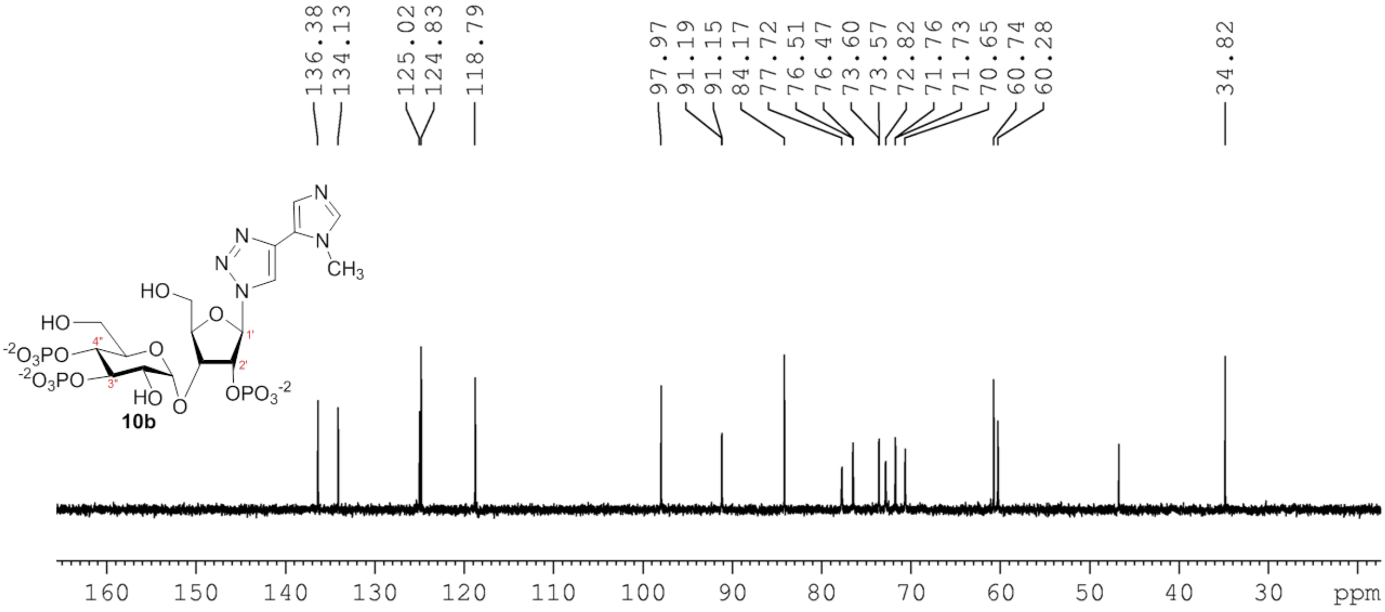

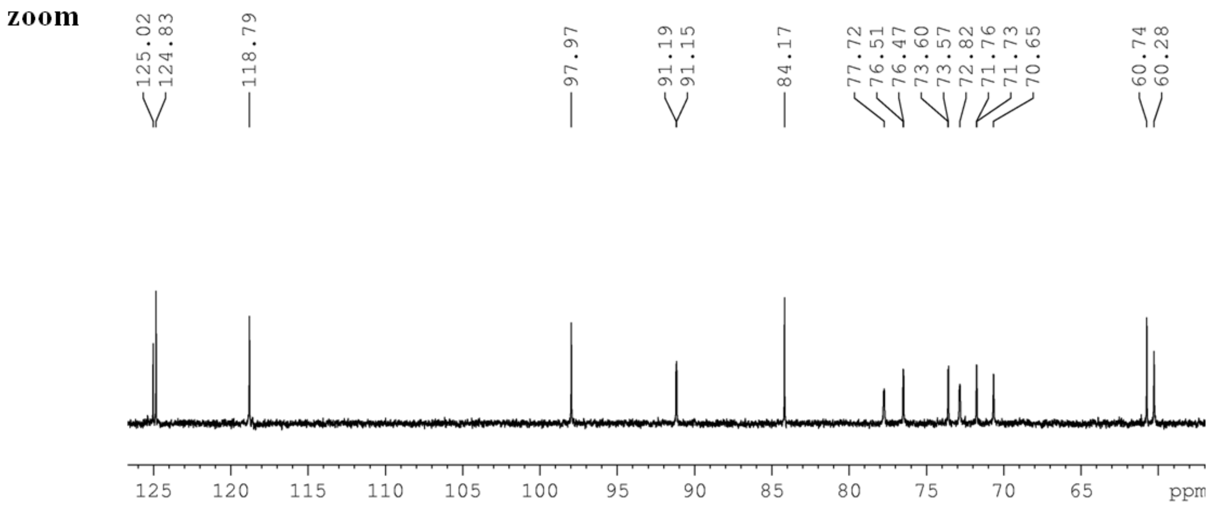

DEPT of 10b in D_2O

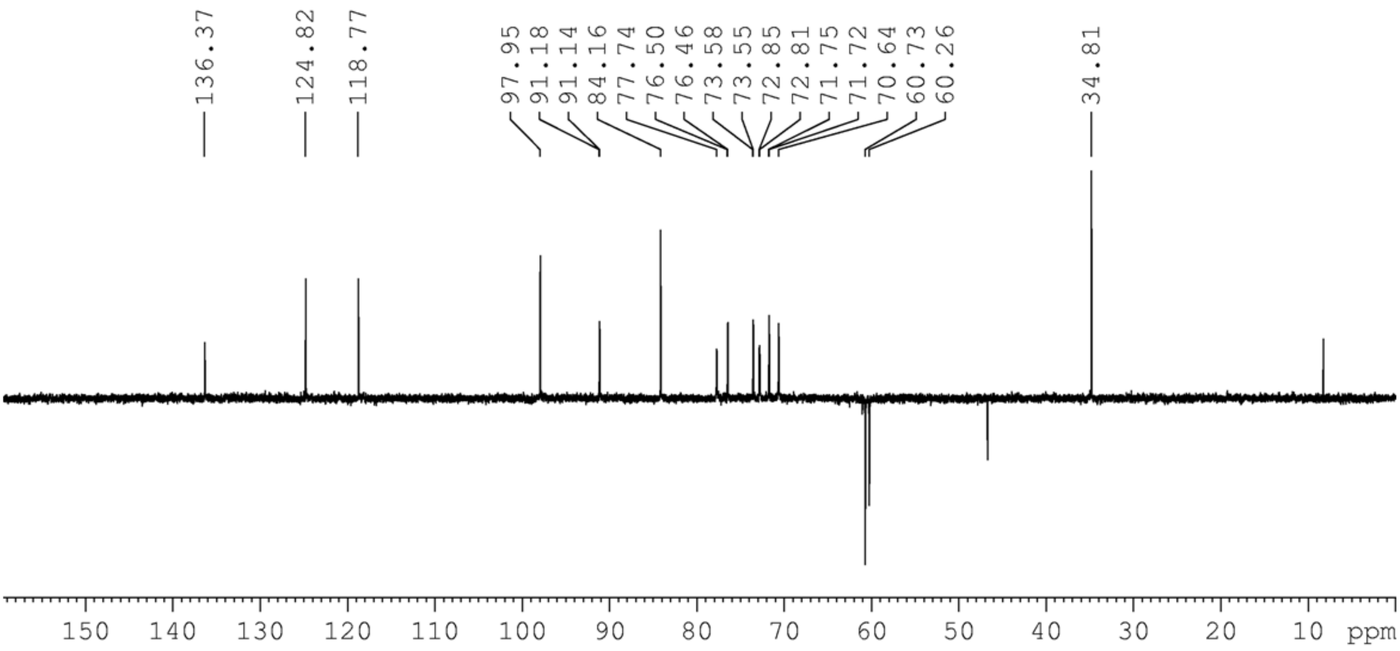

^{13}P NMR of 10b in D_2O

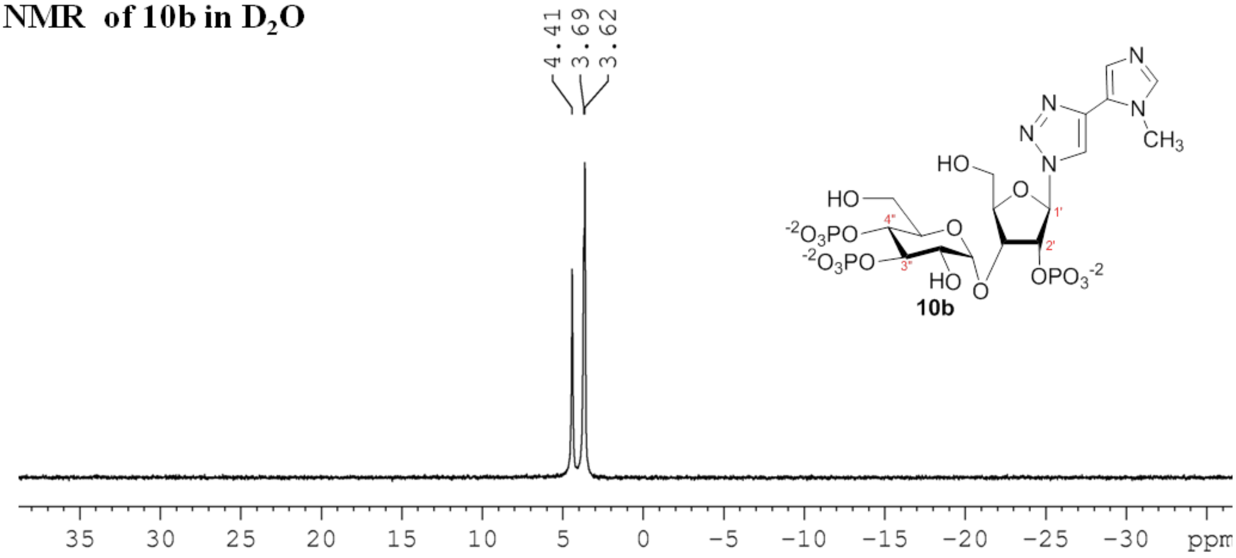

HMBC of 10b in D_2O

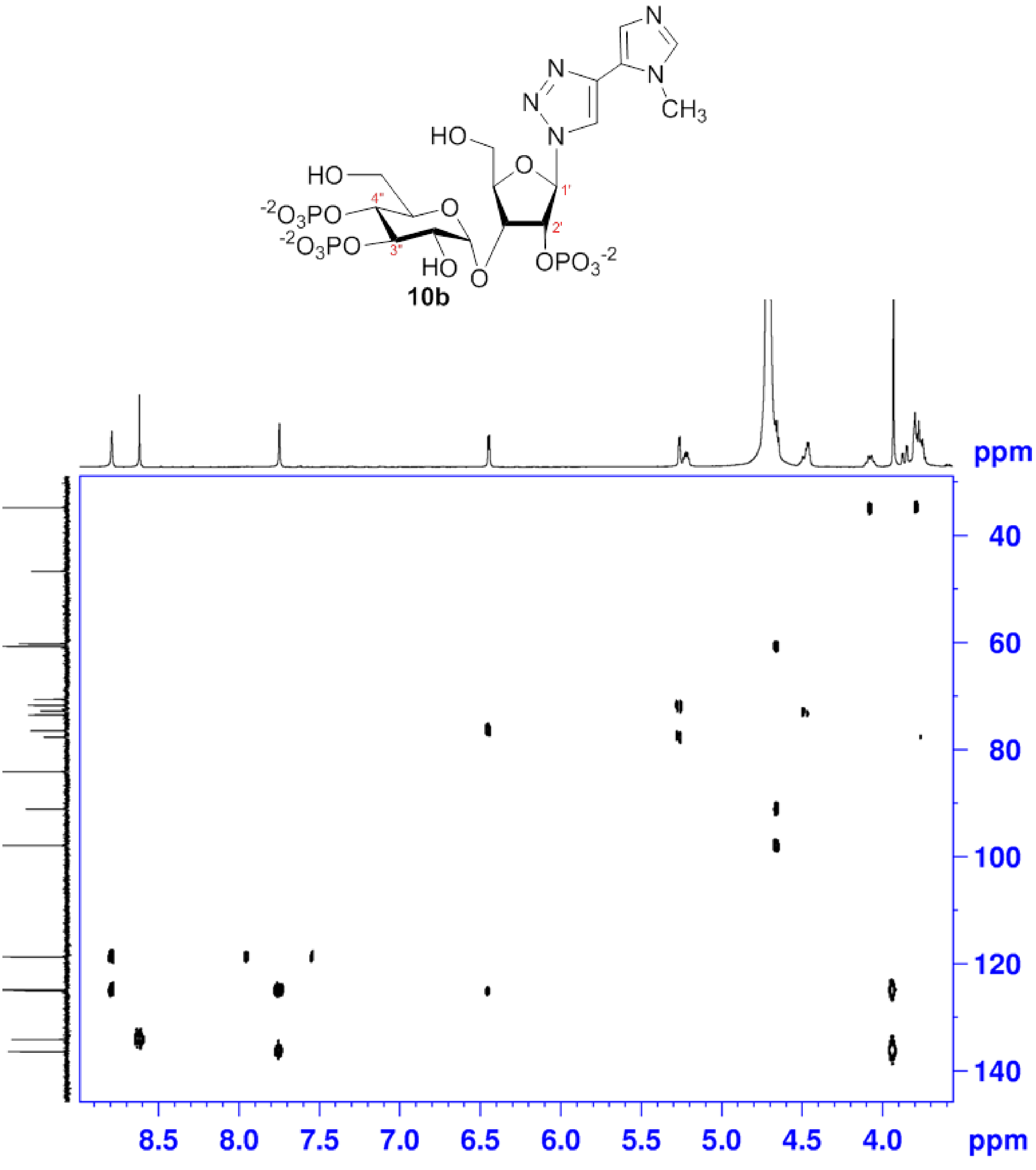

^{1}H NMR of 9c in $CDCl_3$

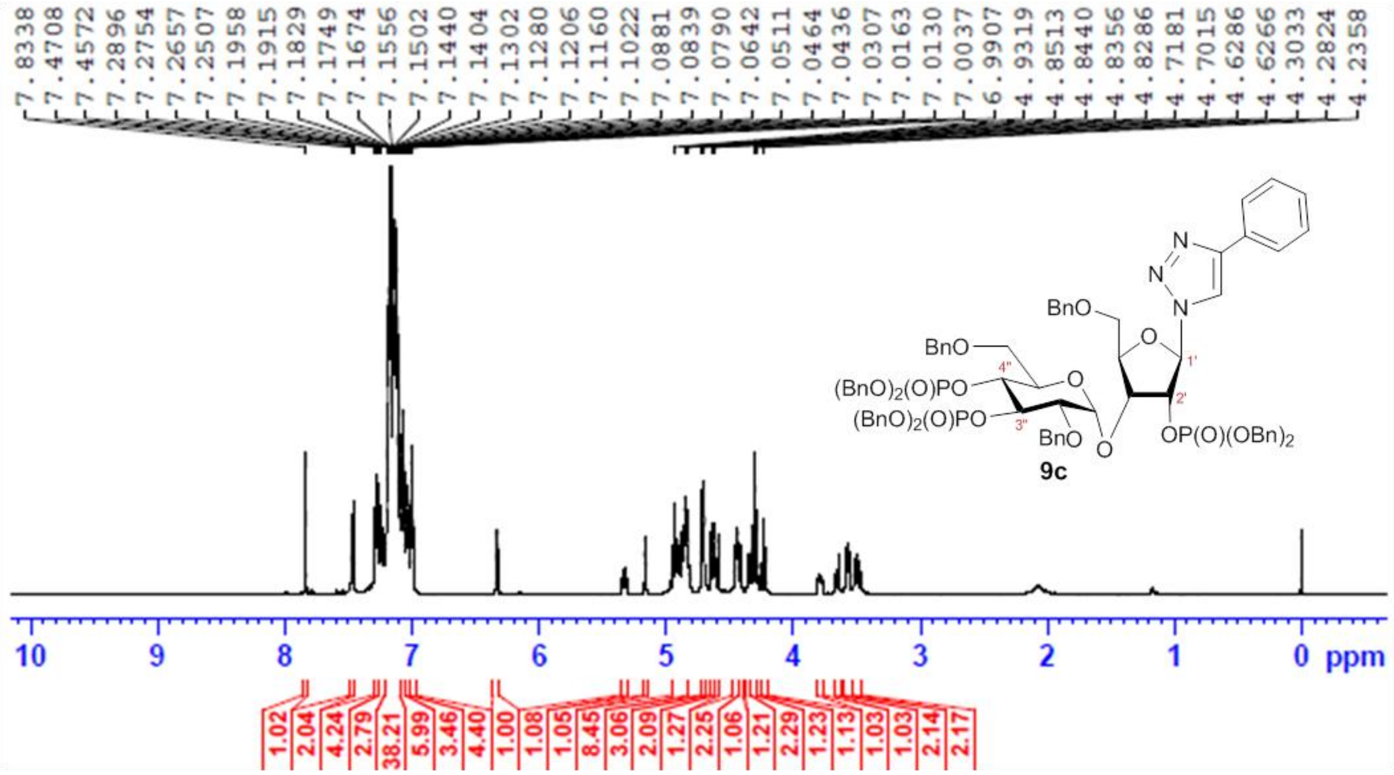

zoom

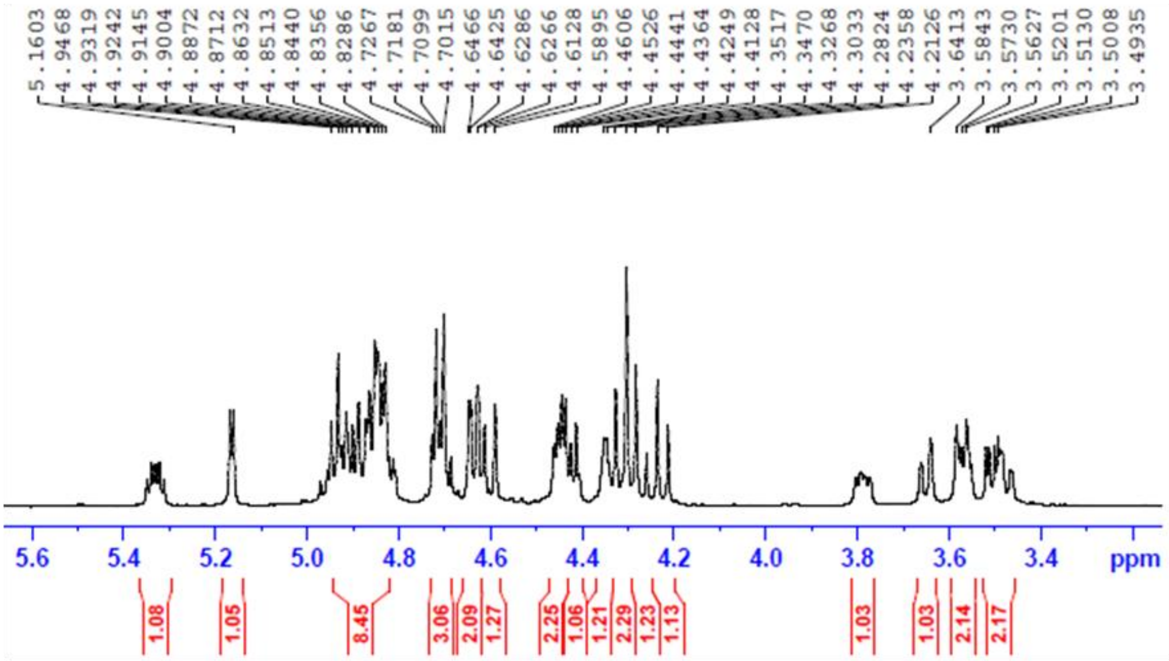

COSY of 9c in $CDCl_3$

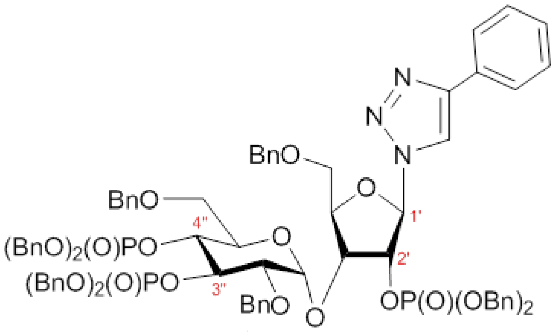

9c

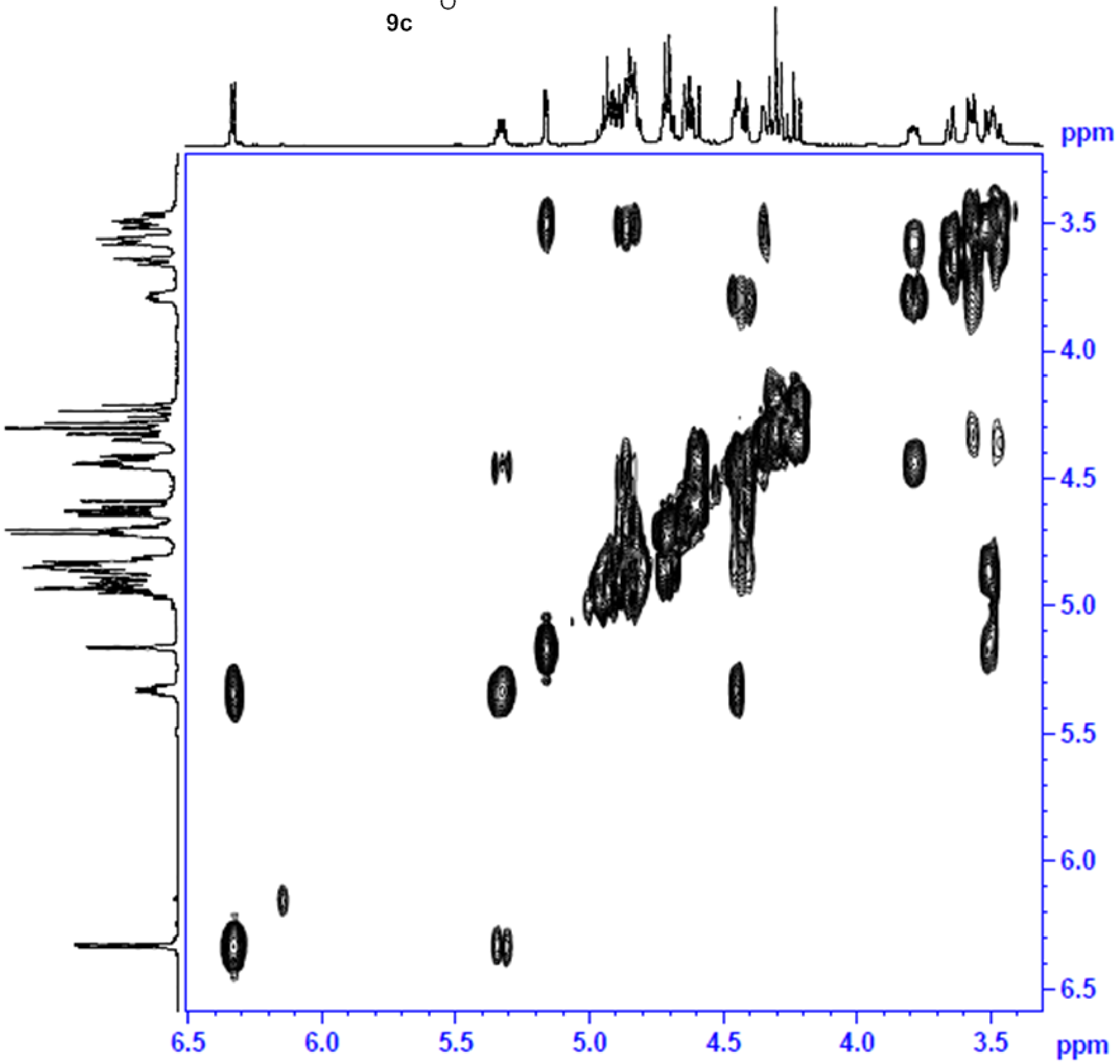

^{13}C NMR of 9c in $CDCl_3$

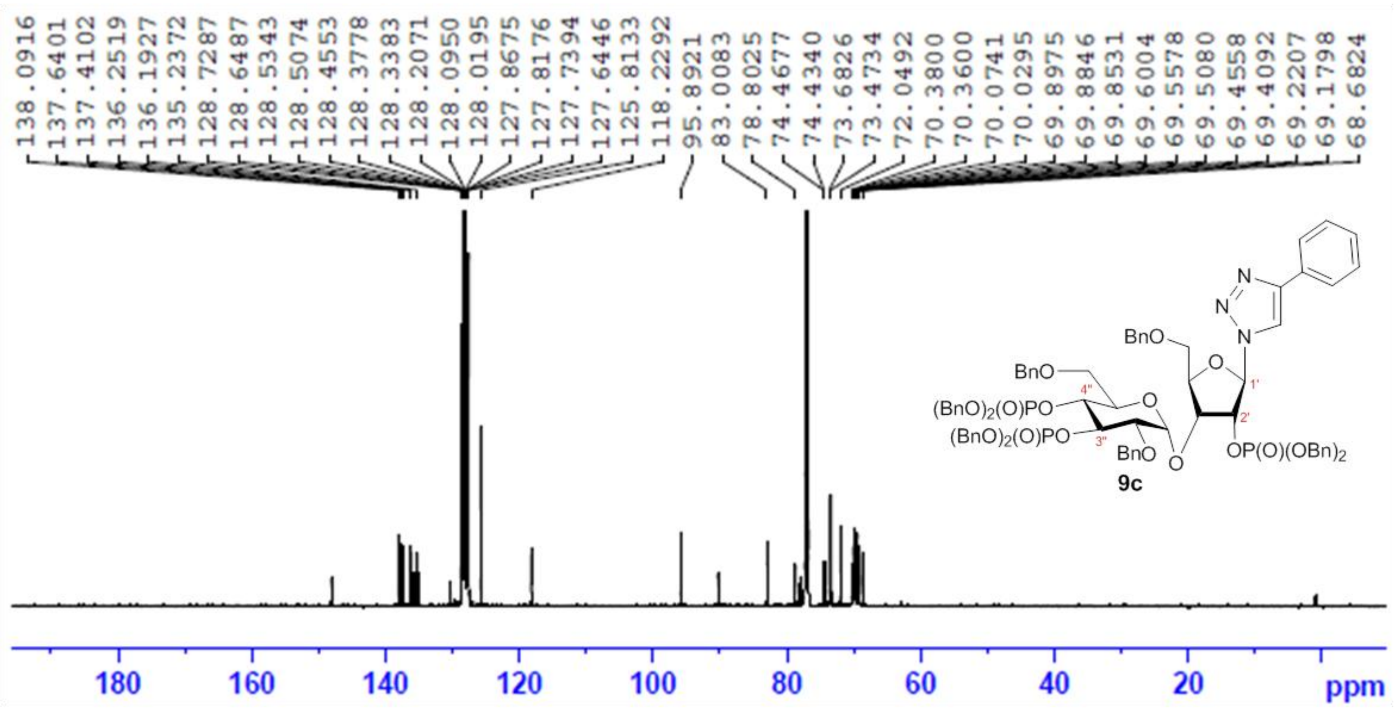

zoom

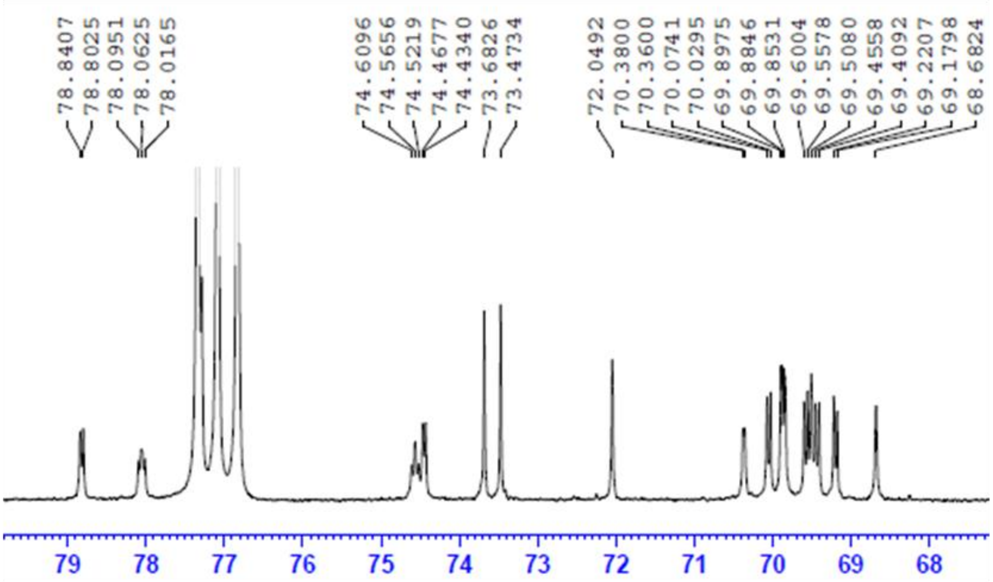

DEPT of 9c in $CDCl_3$

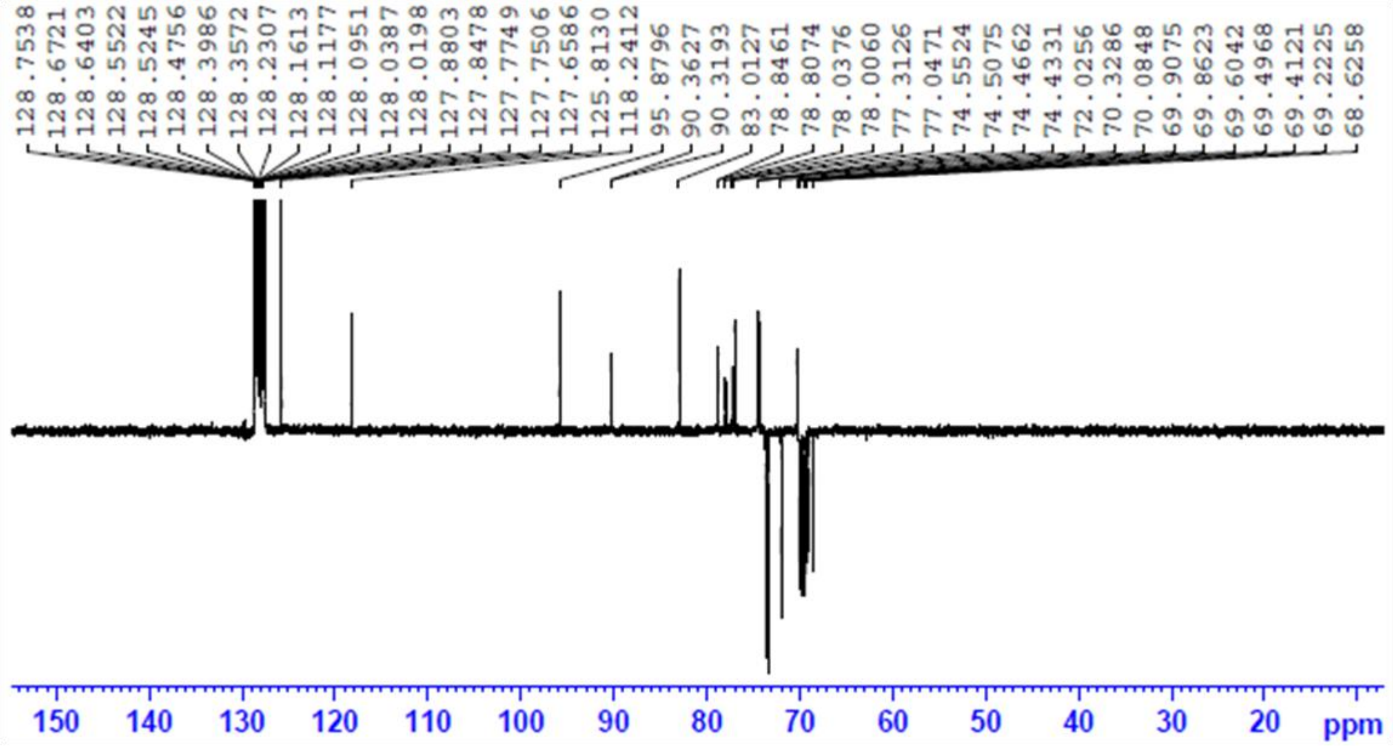

^{31}P NMR of 9c in $CDCl_3$

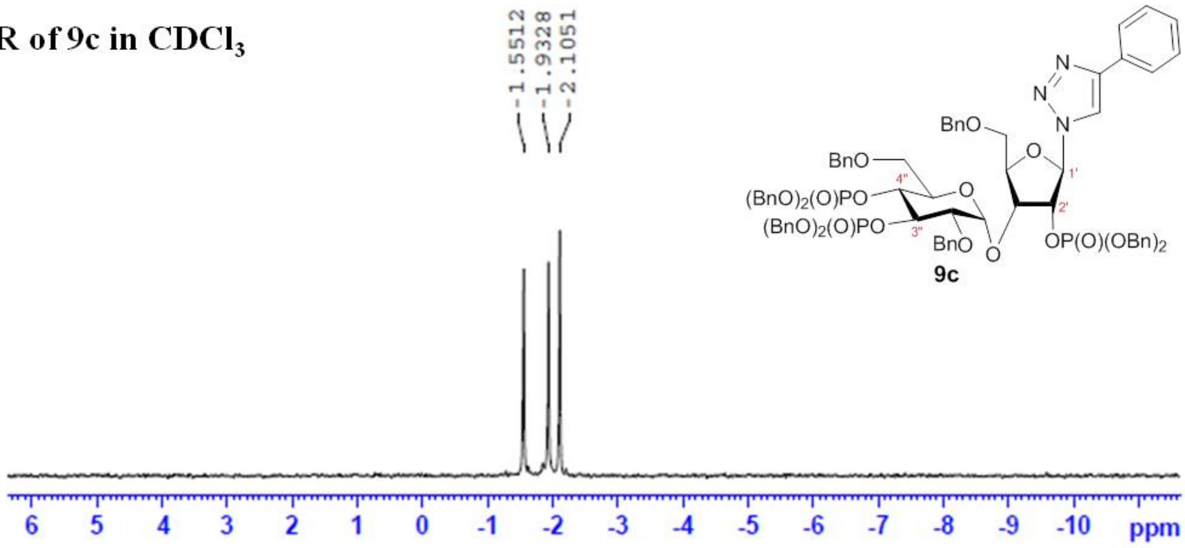

HMBC of 9c in $CDCl_3$

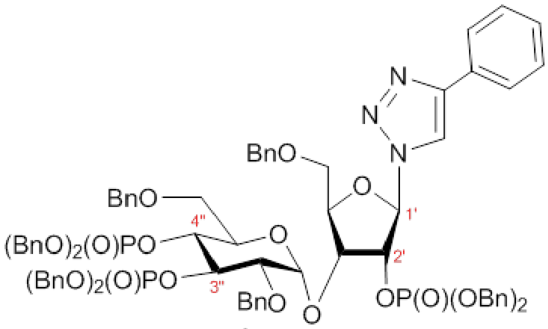

9c

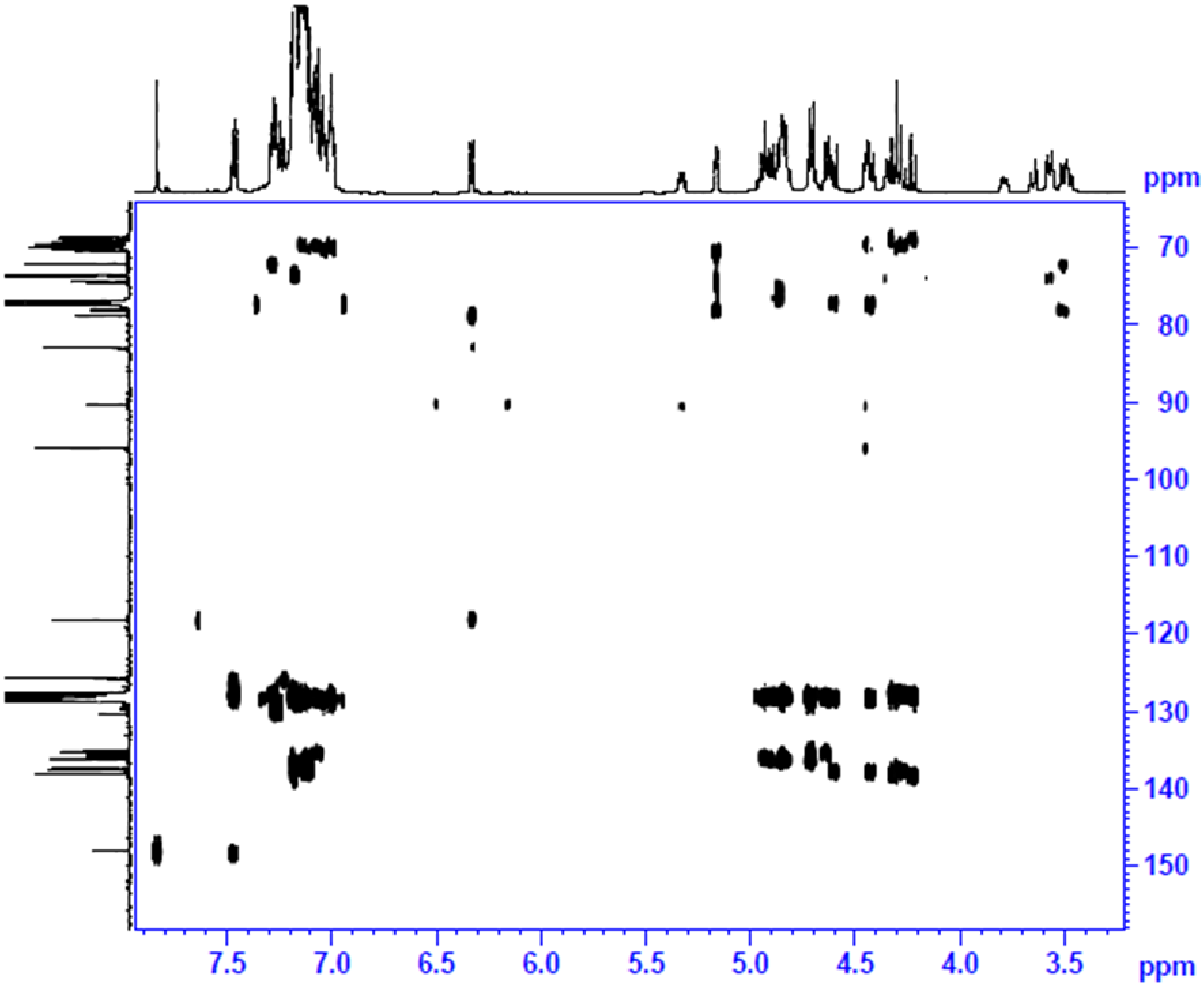

HMQC of 9c in $CDCl_3$

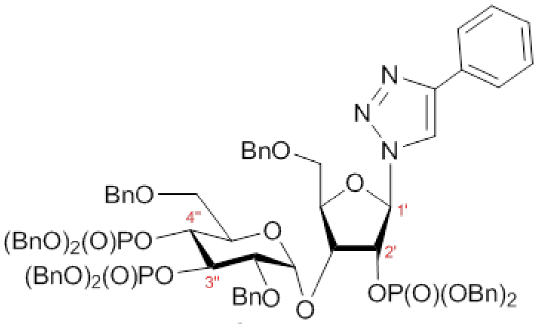

9c

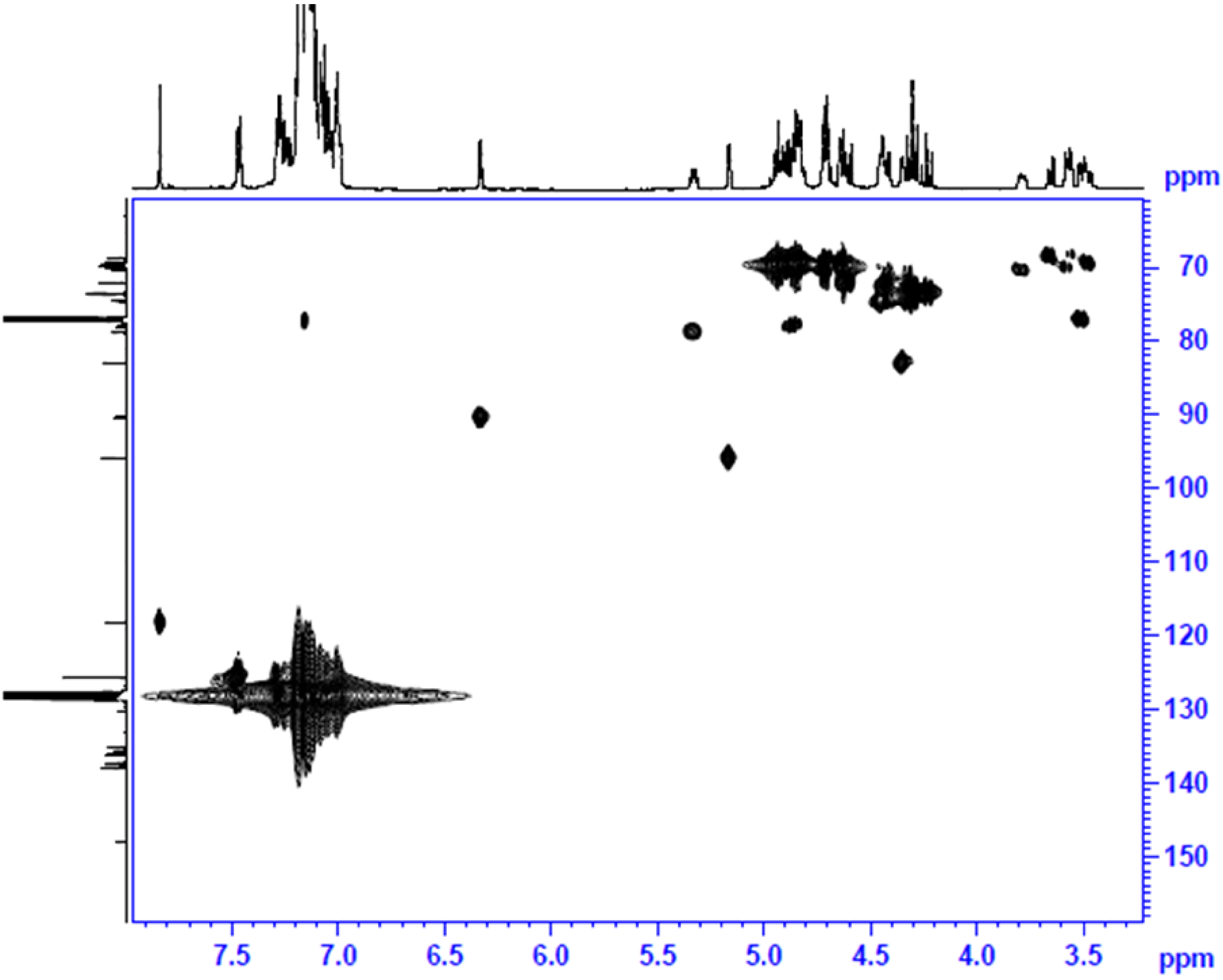

^{1}H NMR of 10c in D_2O

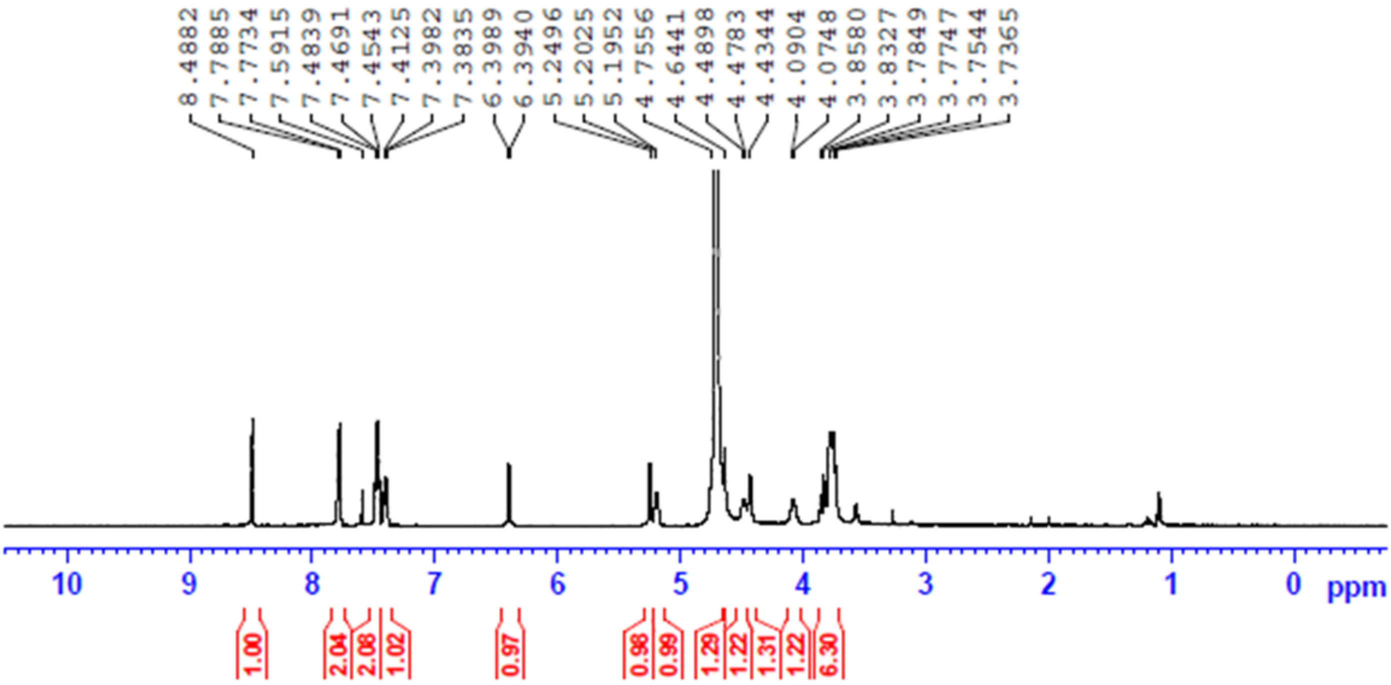

^{31}P NMR of 10c in D_2O

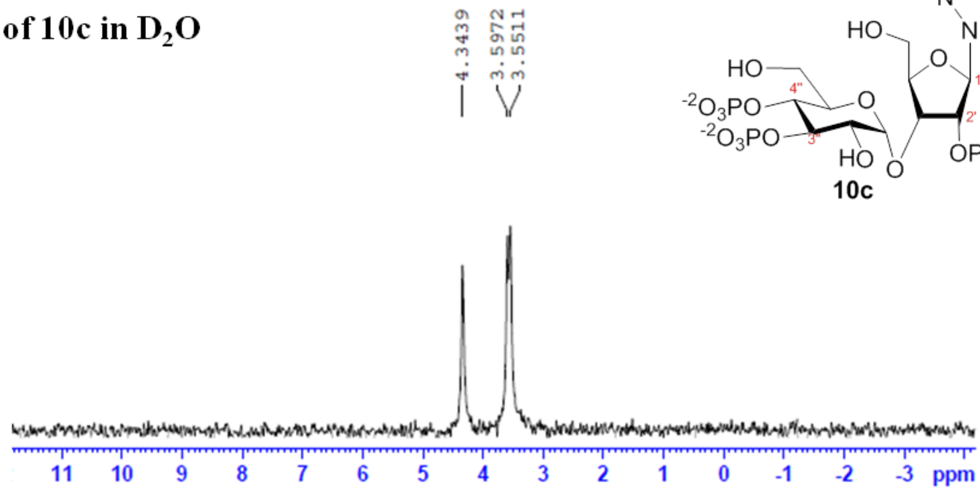

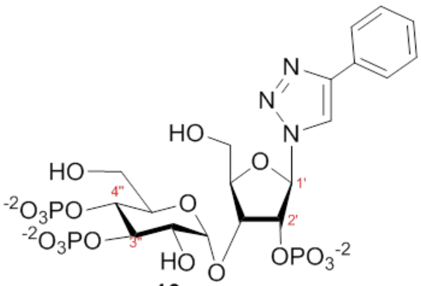

10c

COSY of 10c in D_2O

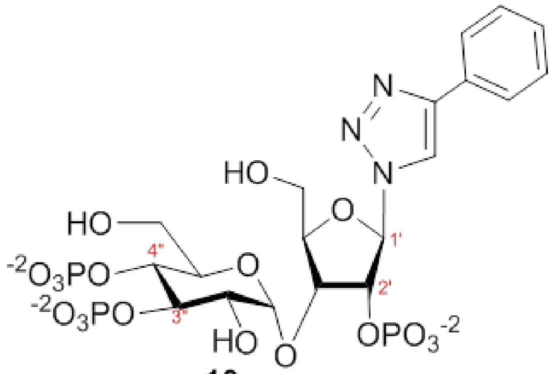

10c

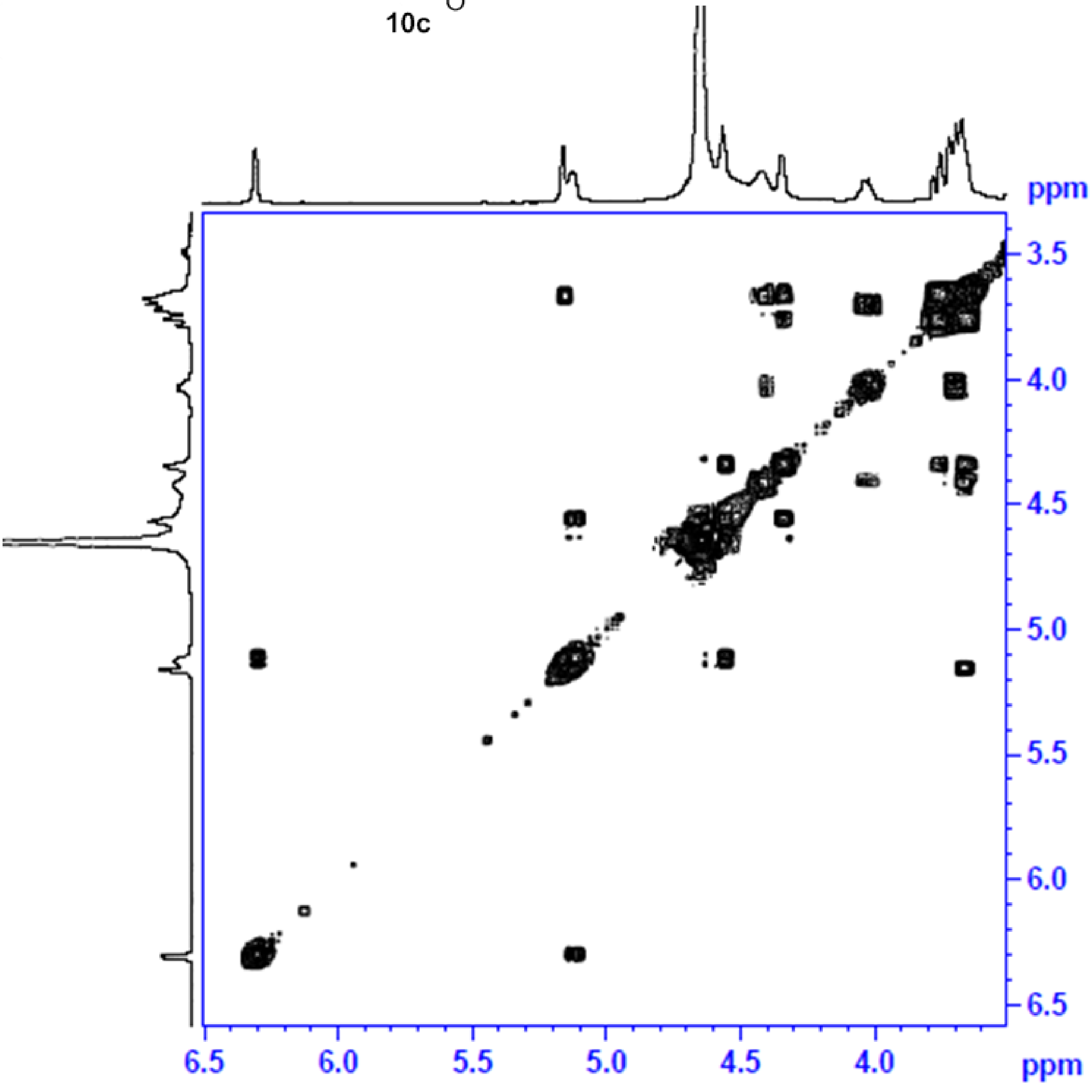

^{13}C NMR of 10c in D_2O

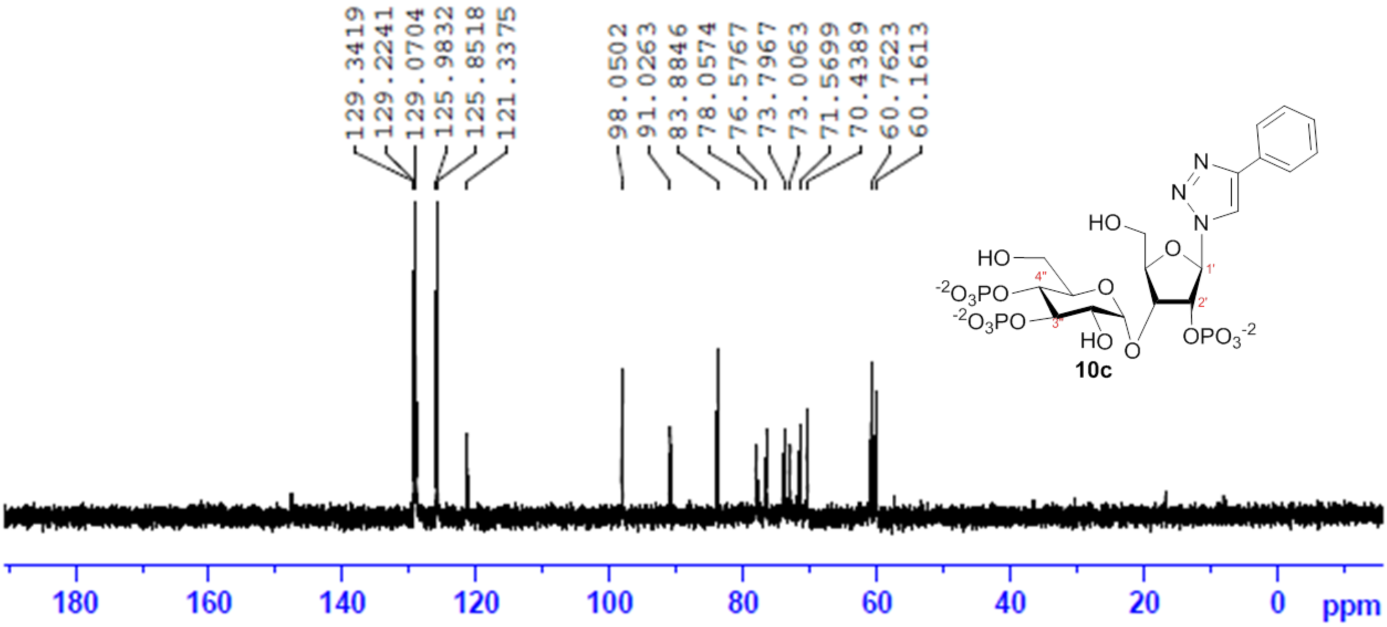

DEPT of 10c in D_2O

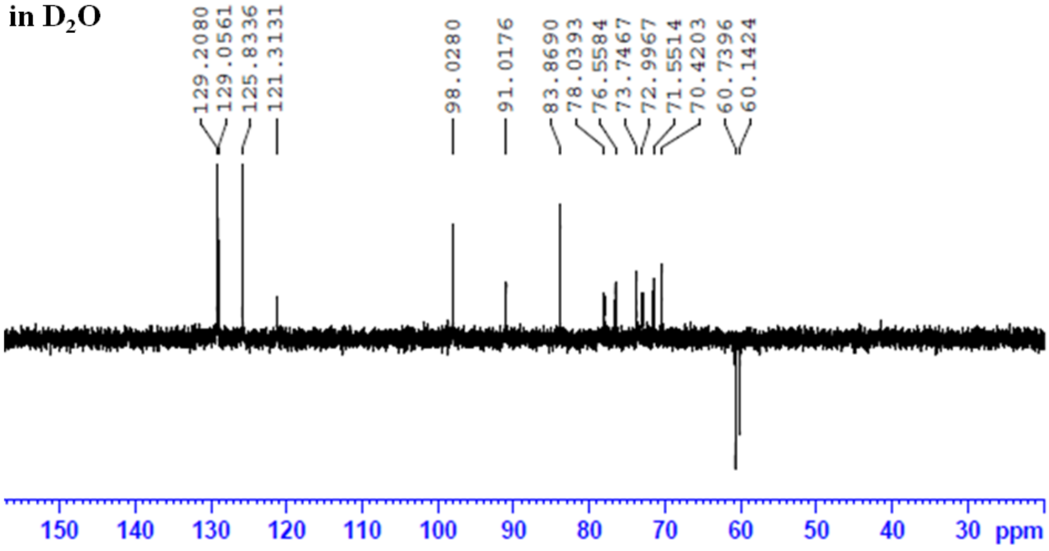

HMBC of 10c in D_2O

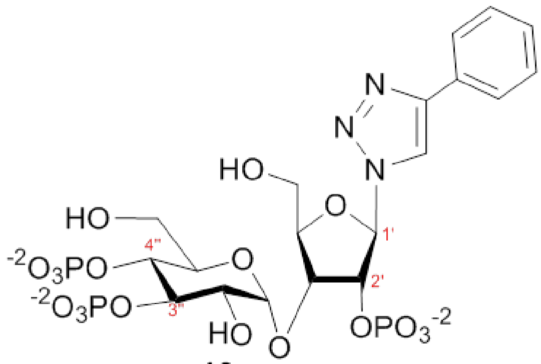

10c

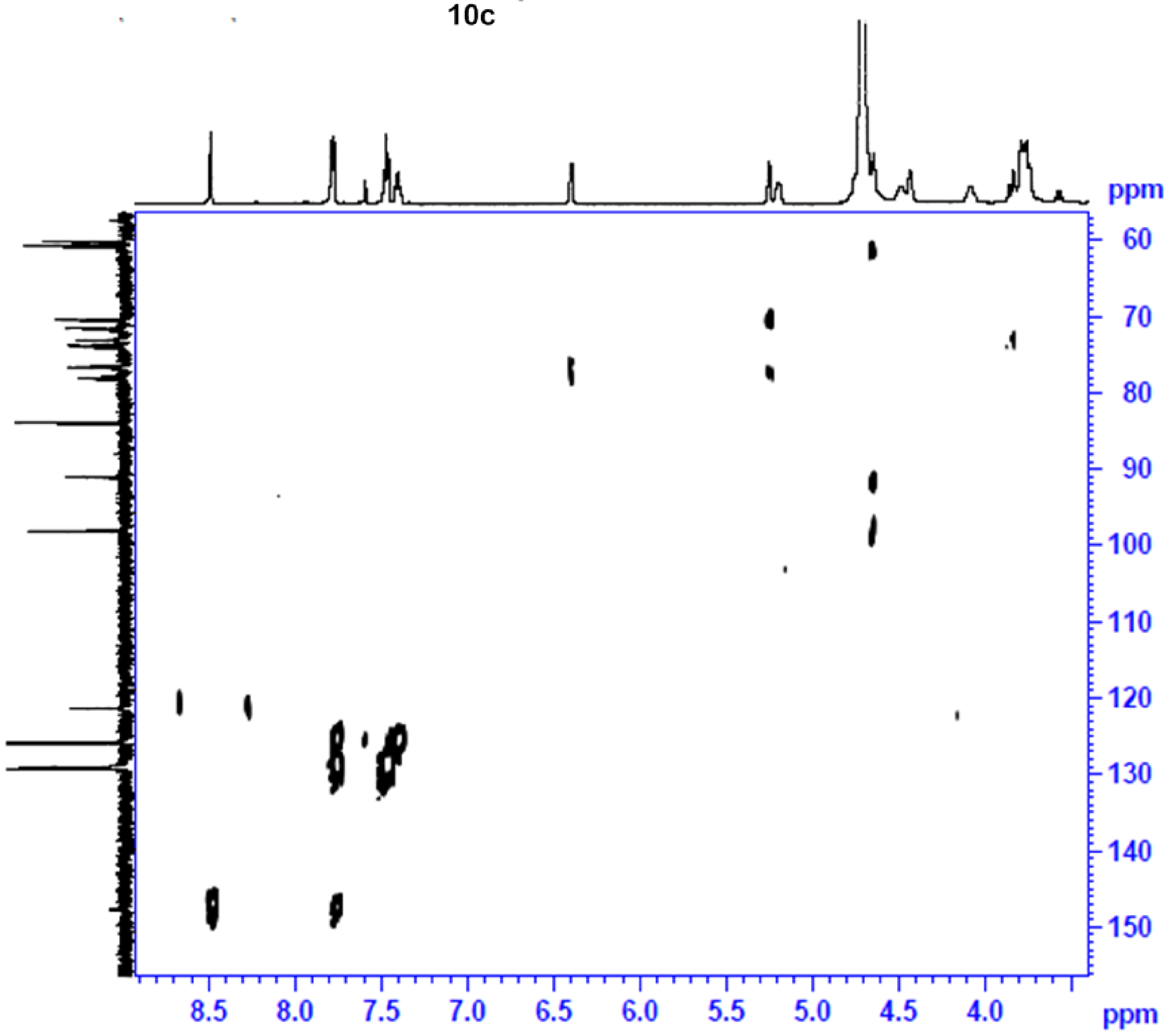

HMQC of 10c in D_2O

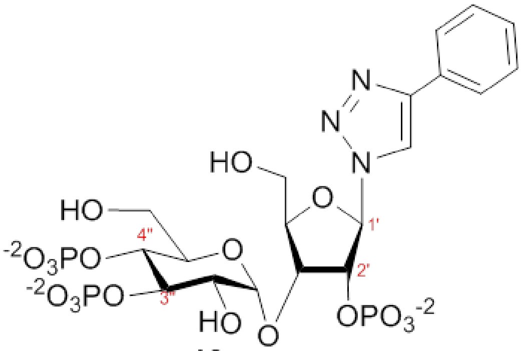

10c

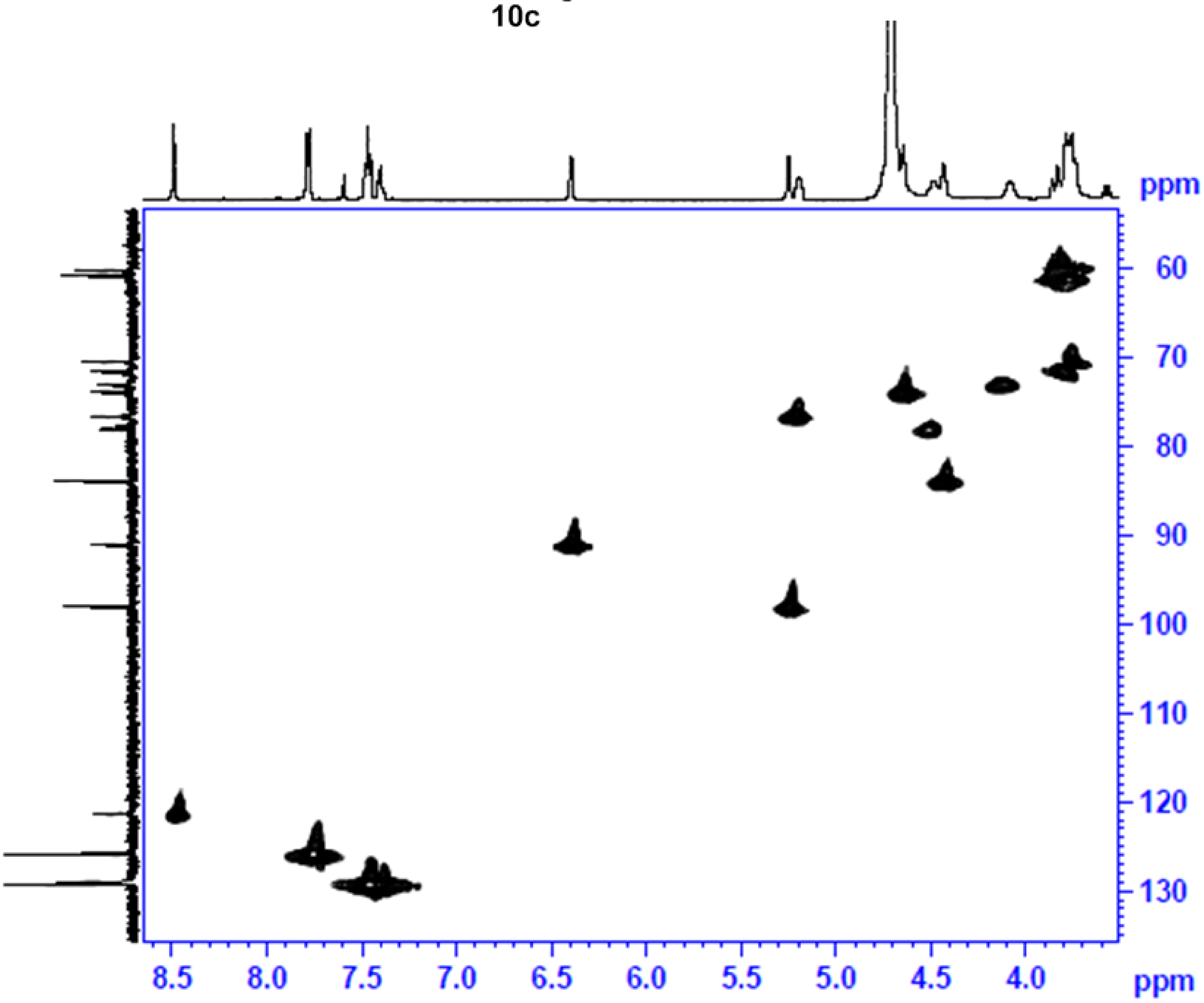

^{1}H NMR of 9d in $CDCl_3$

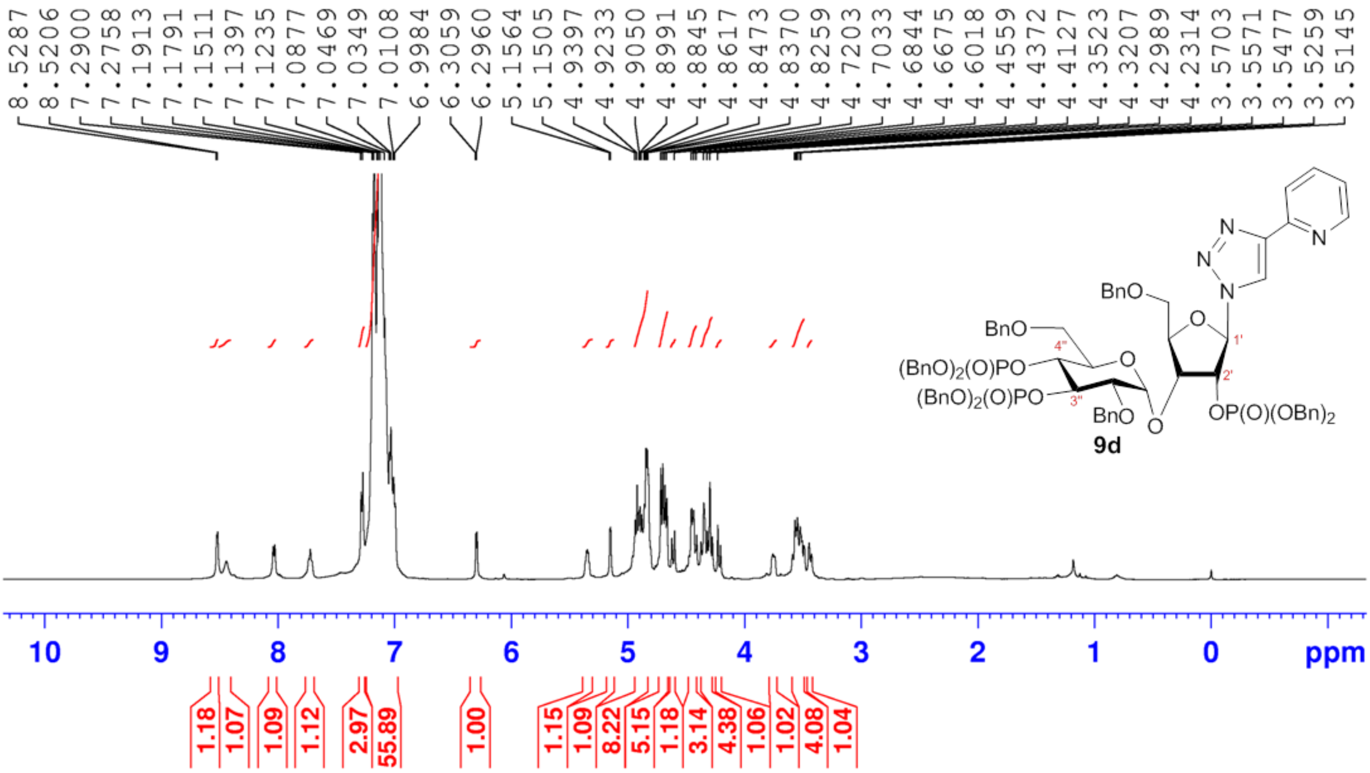

zoom

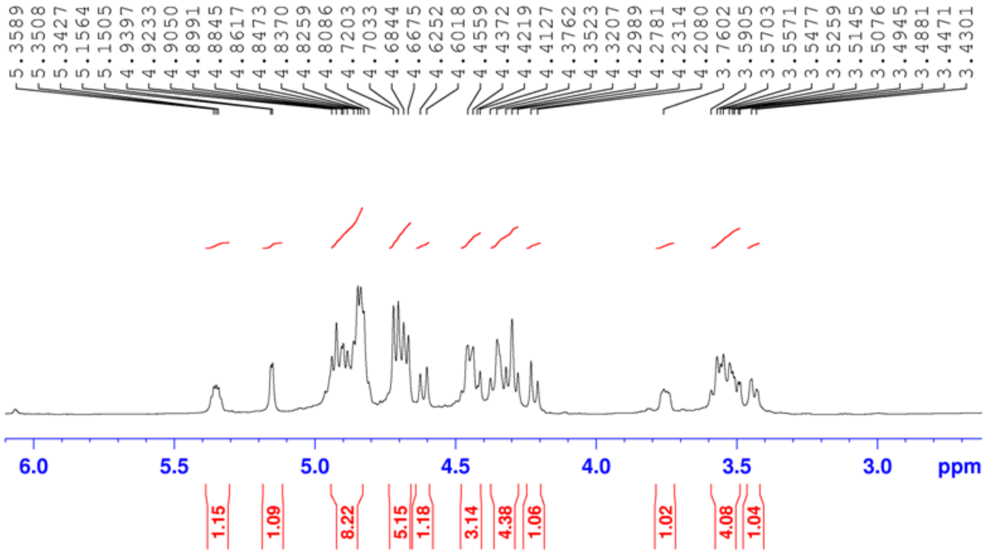

COSY of 9d in $CDCl_3$

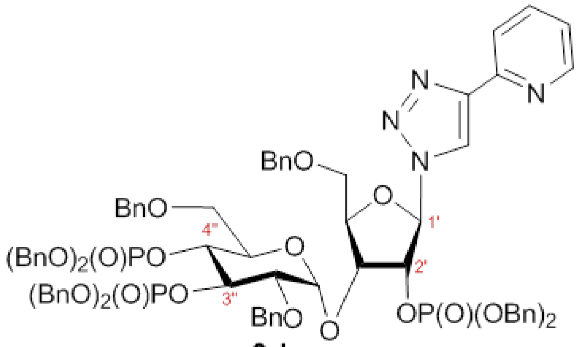

9d

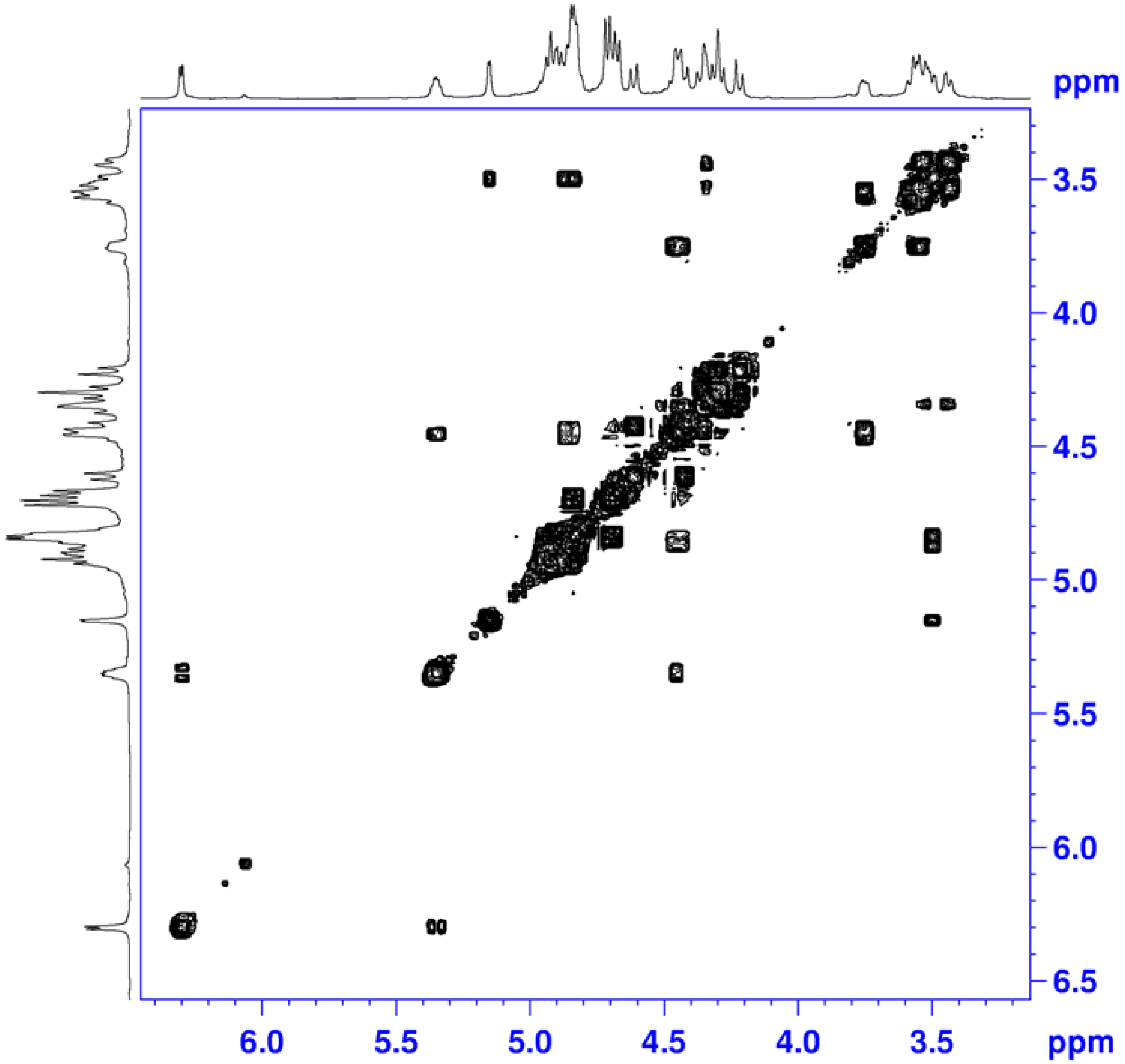

^{13}C NMR of 9d in $CDCl_3$

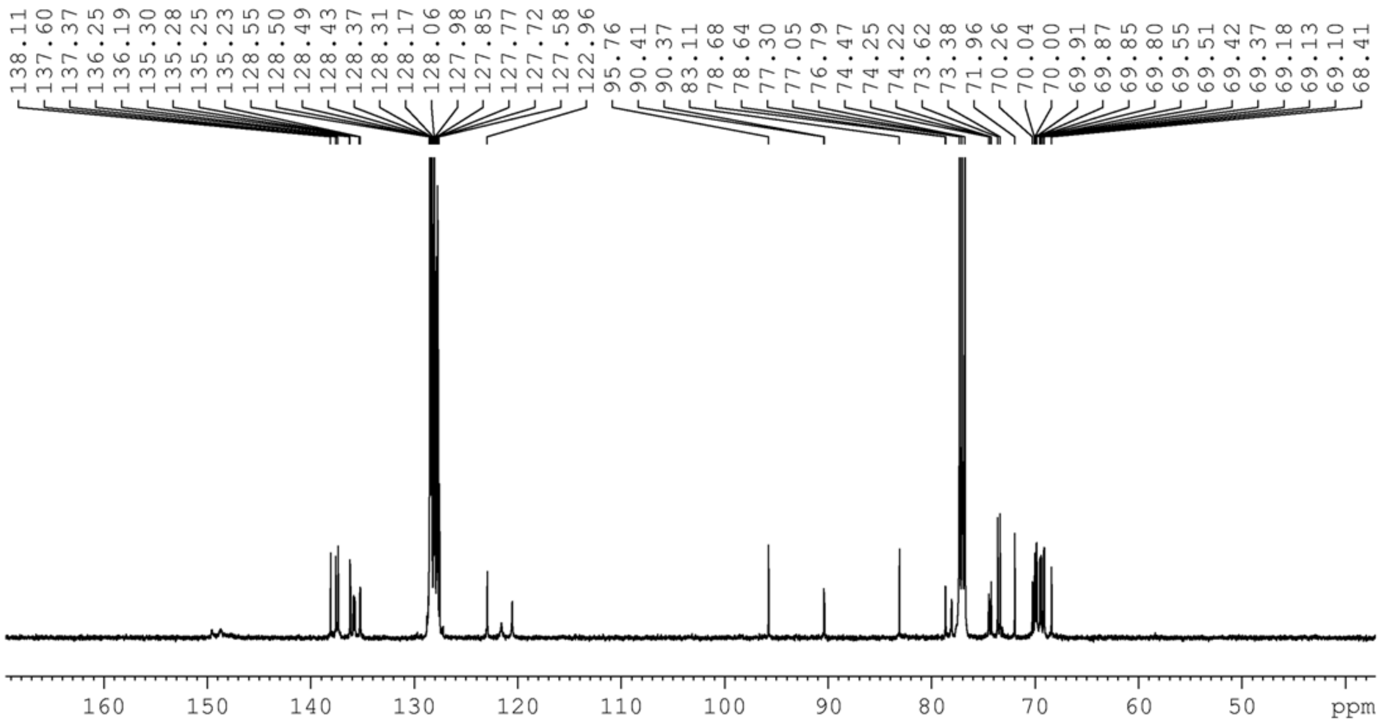

zoom

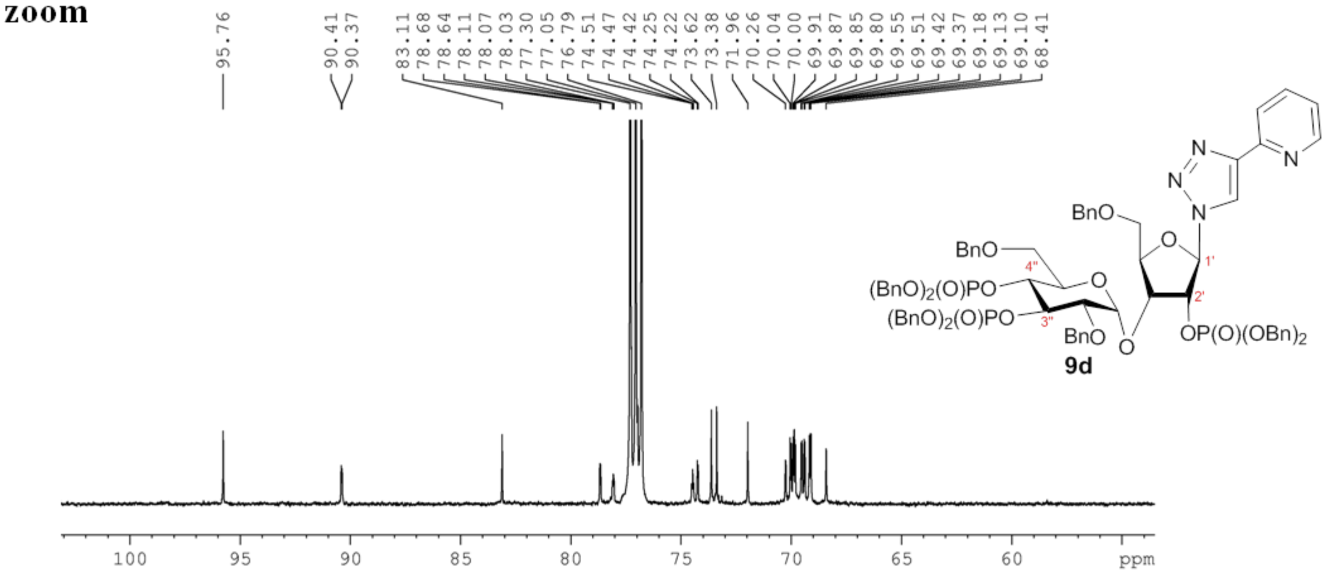

9d

DEPT of 9d in $CDCl_3$

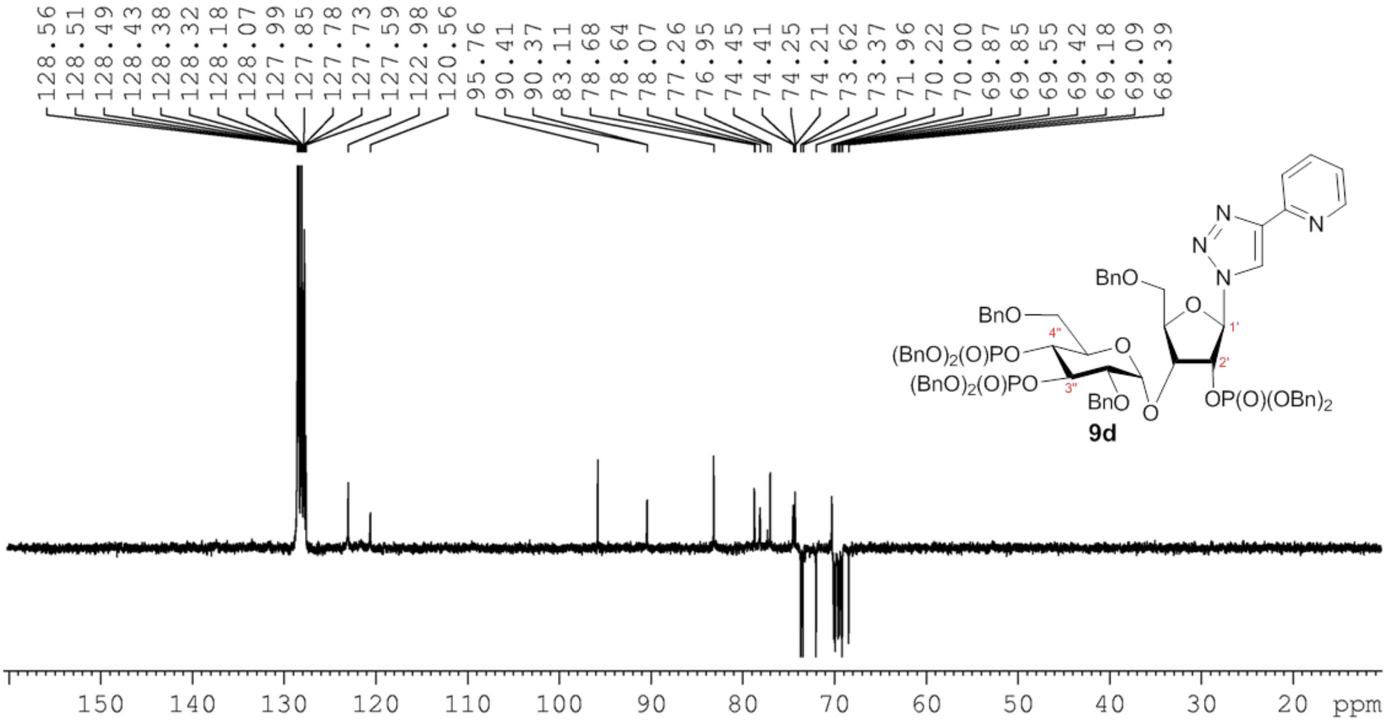

^{31}P NMR of 9d in $CDCl_3$

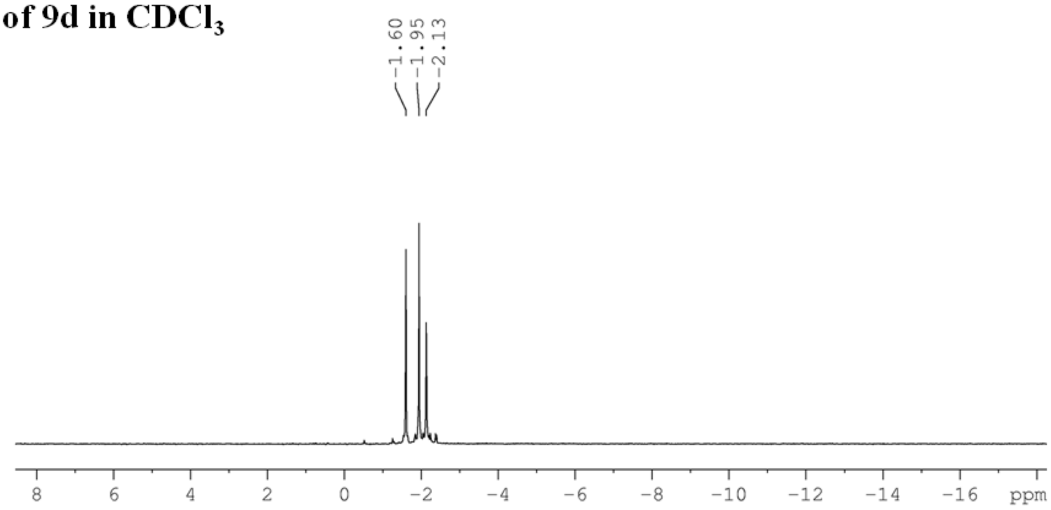

HMBC of 9d in CDCl$_3$

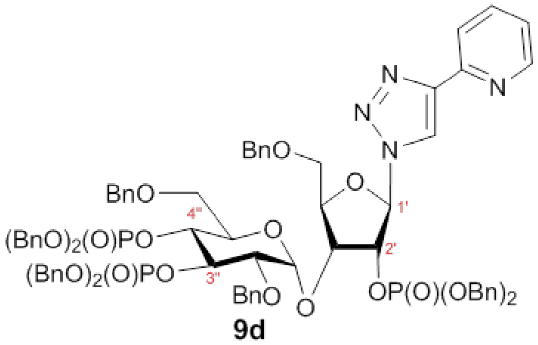

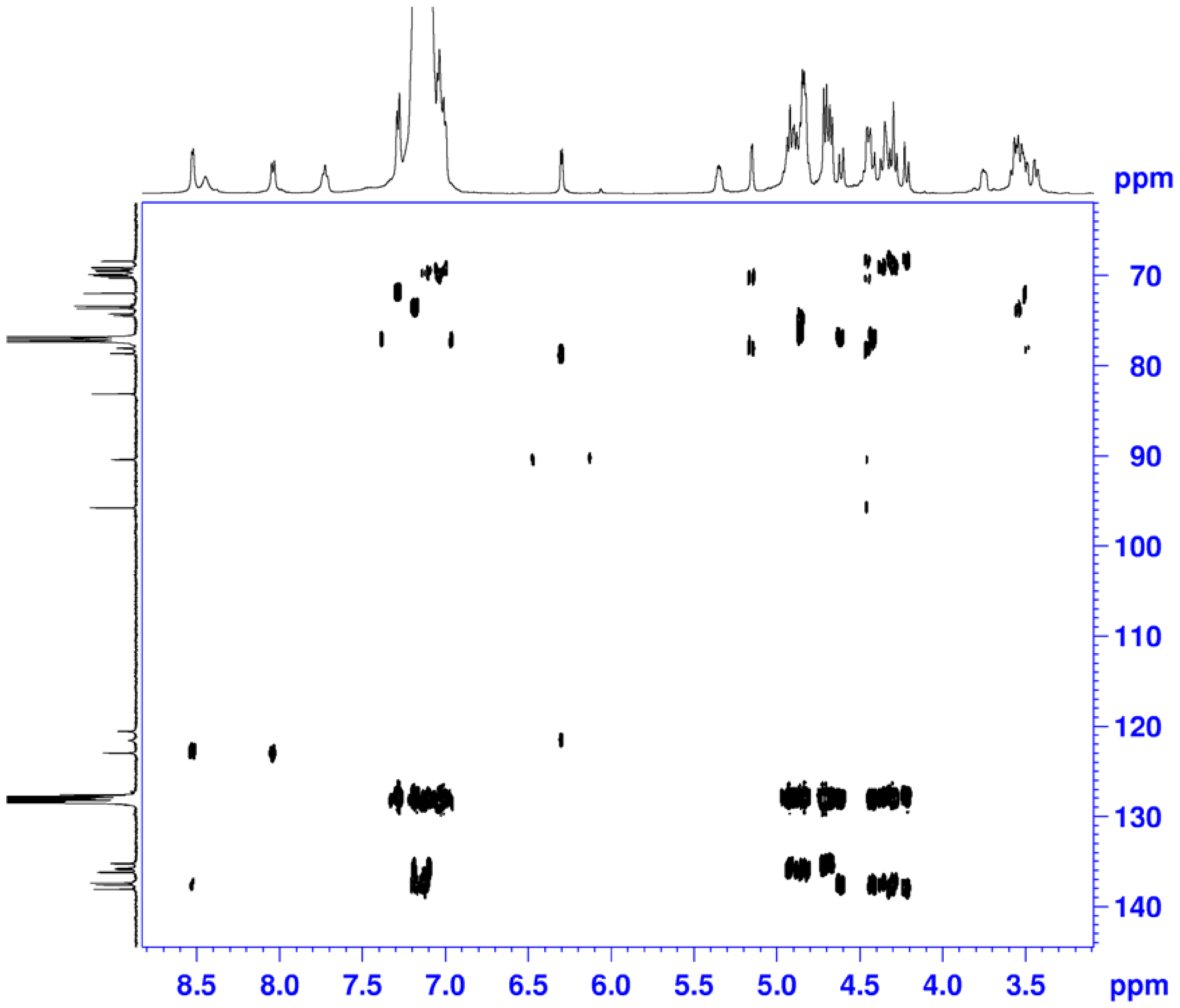

HMQC of 9d in $CDCl_3$

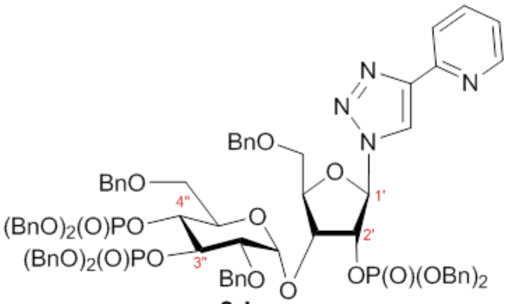

9d

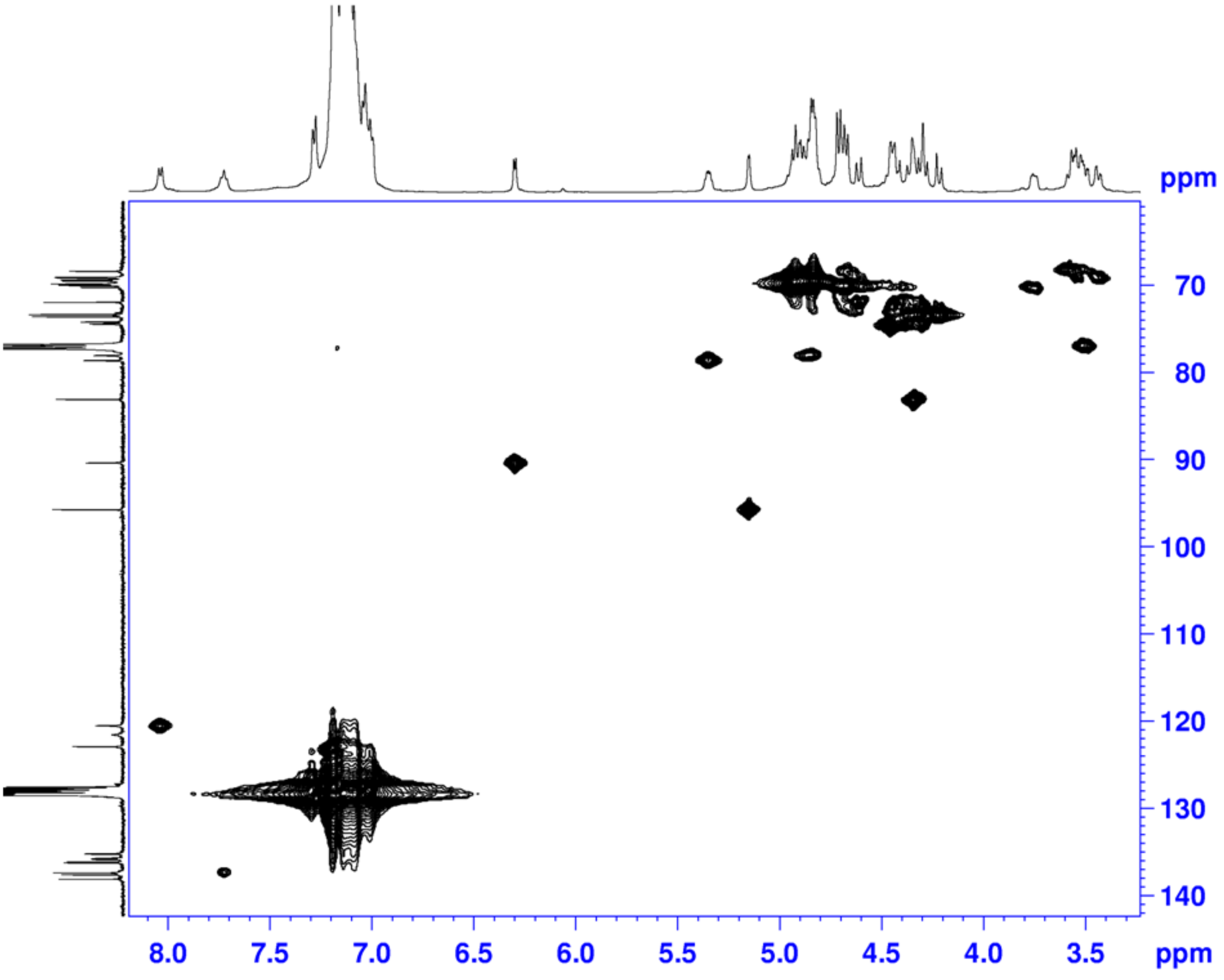

^1H NMR of 10d in D_2O

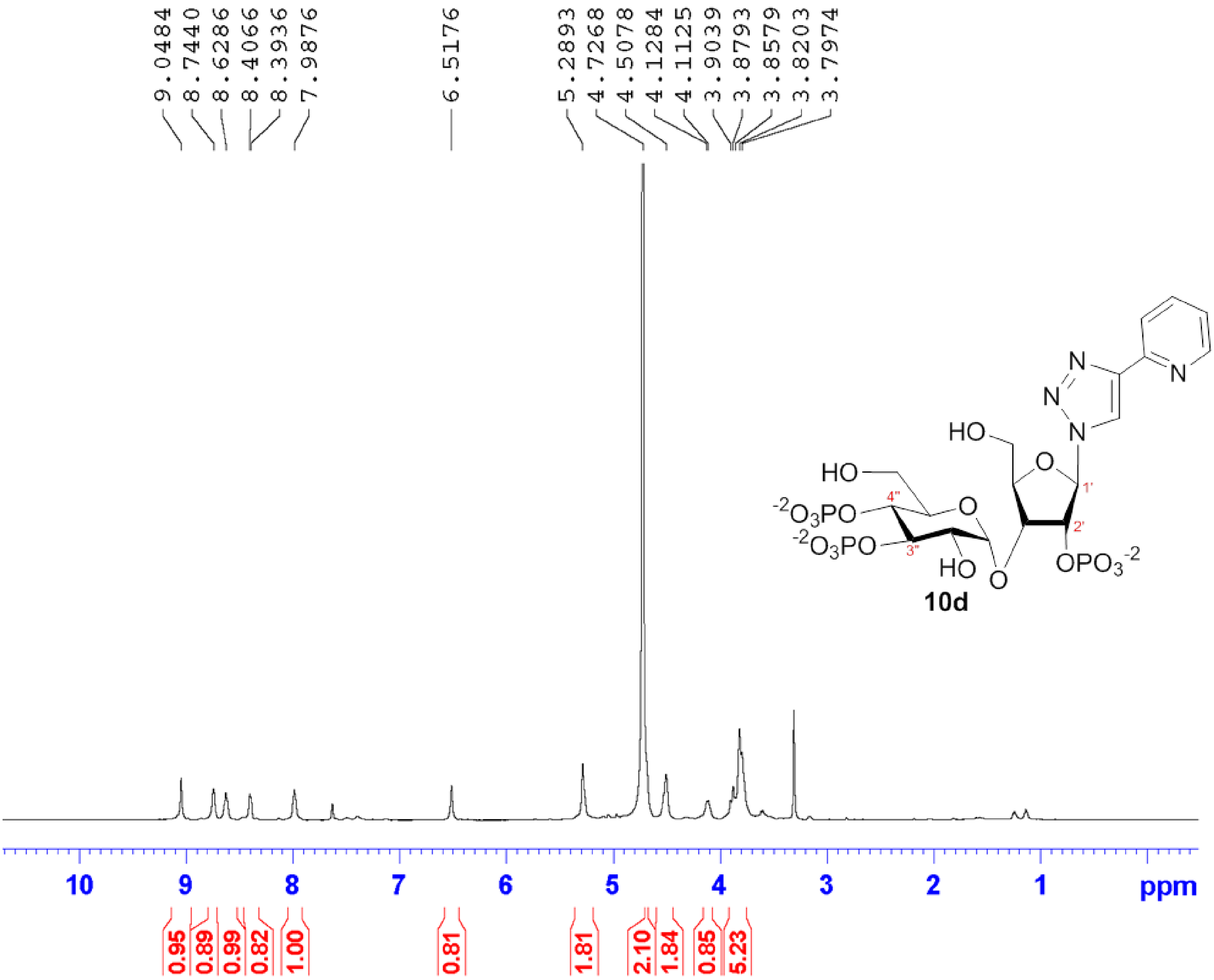

COSY of 10d in D_2O

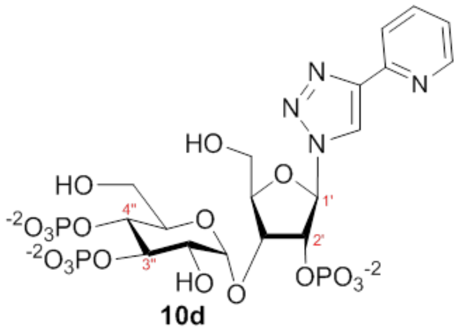

10d

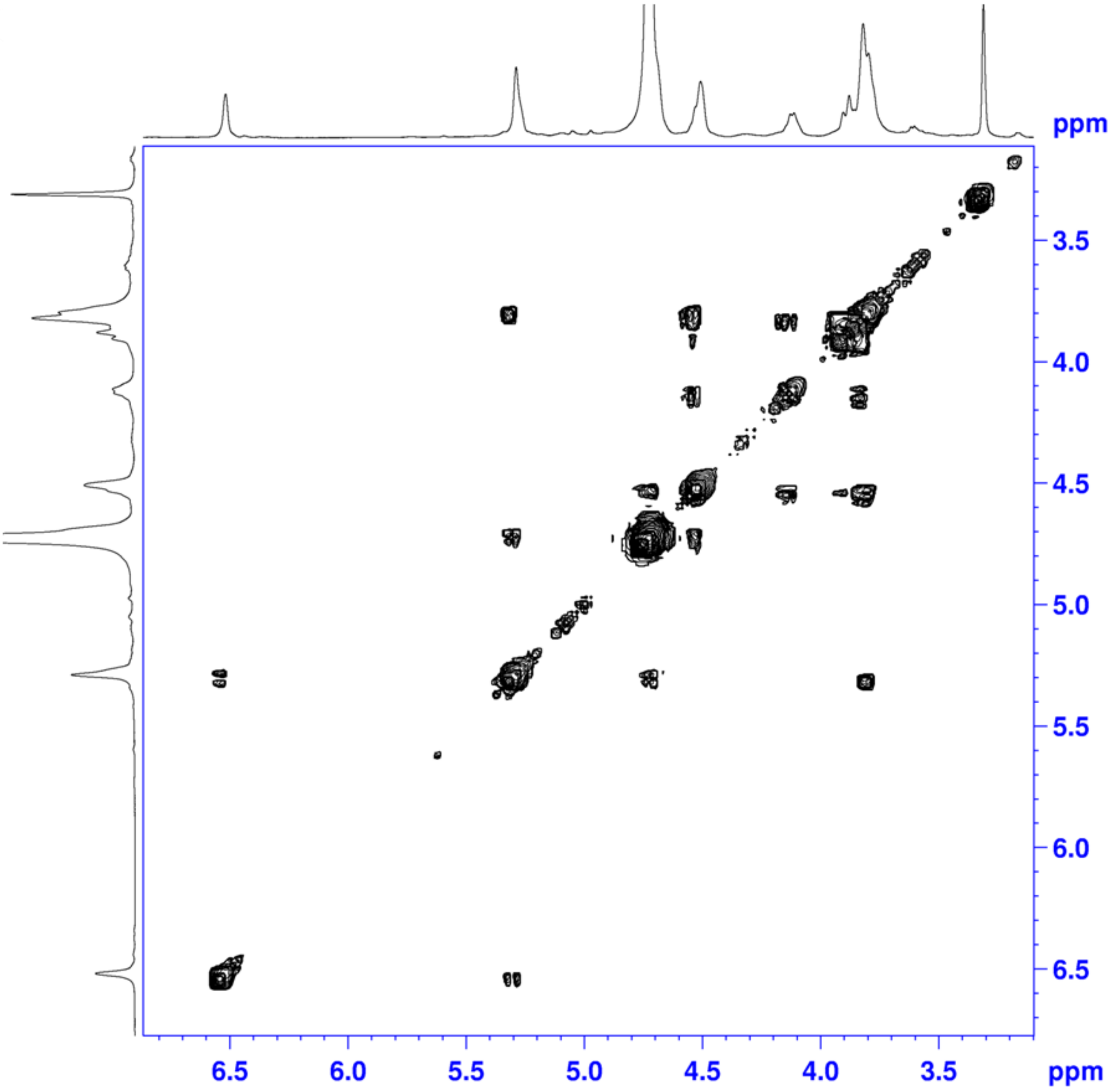

^{13}C NMR of 10d in D_2O

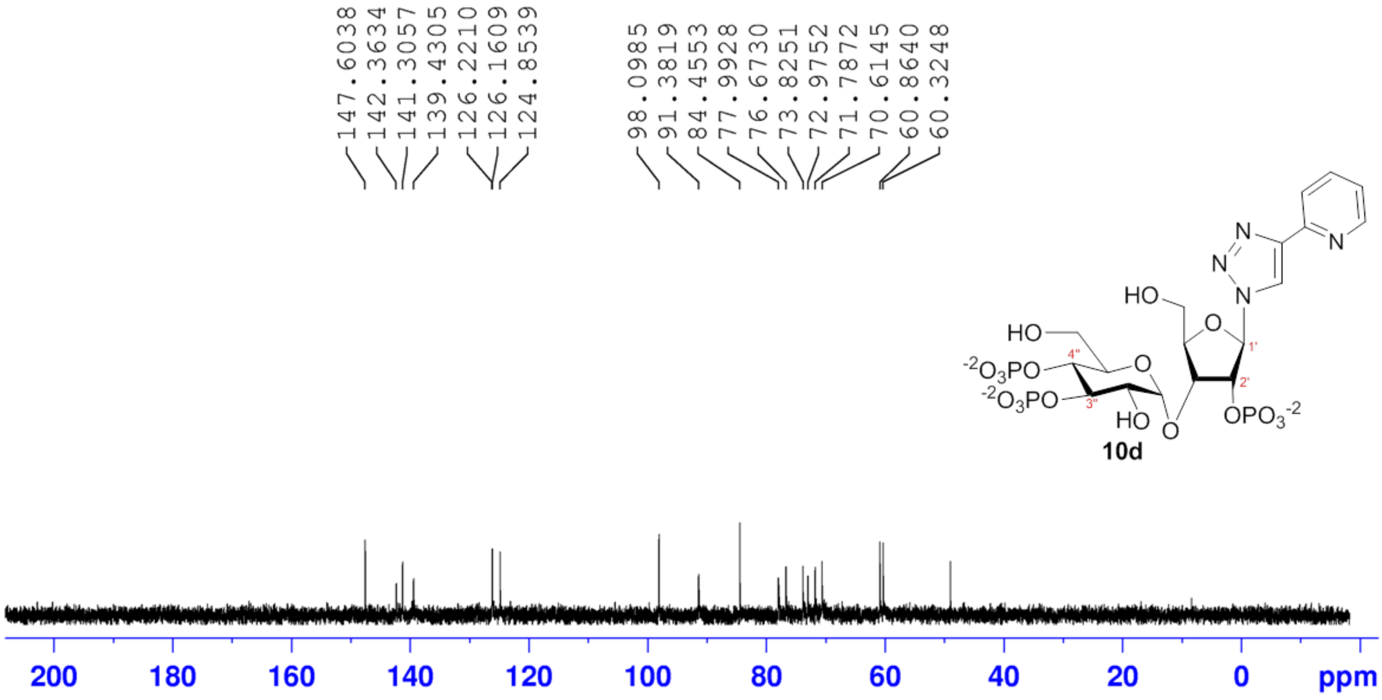

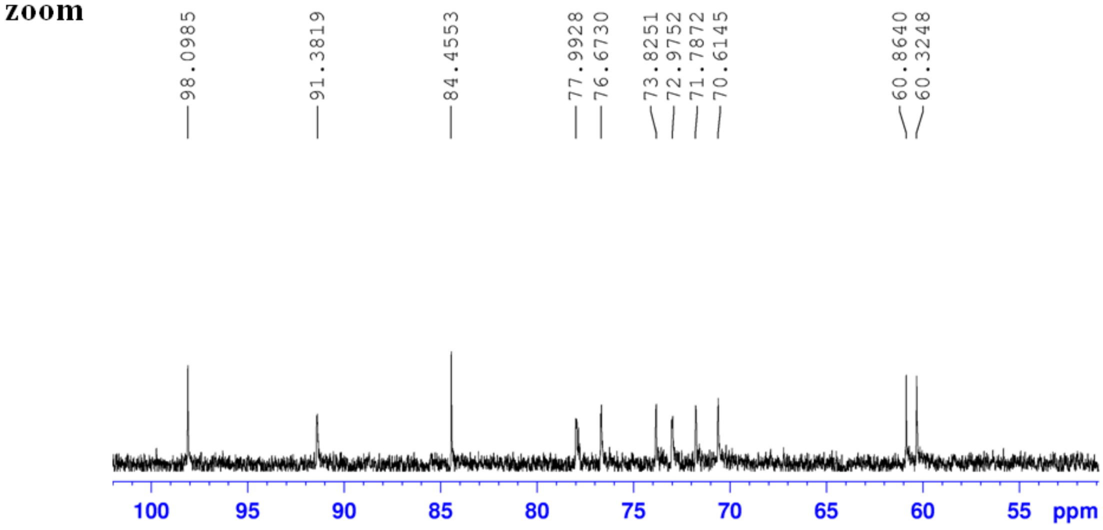

DEPT of 10d in D_2O

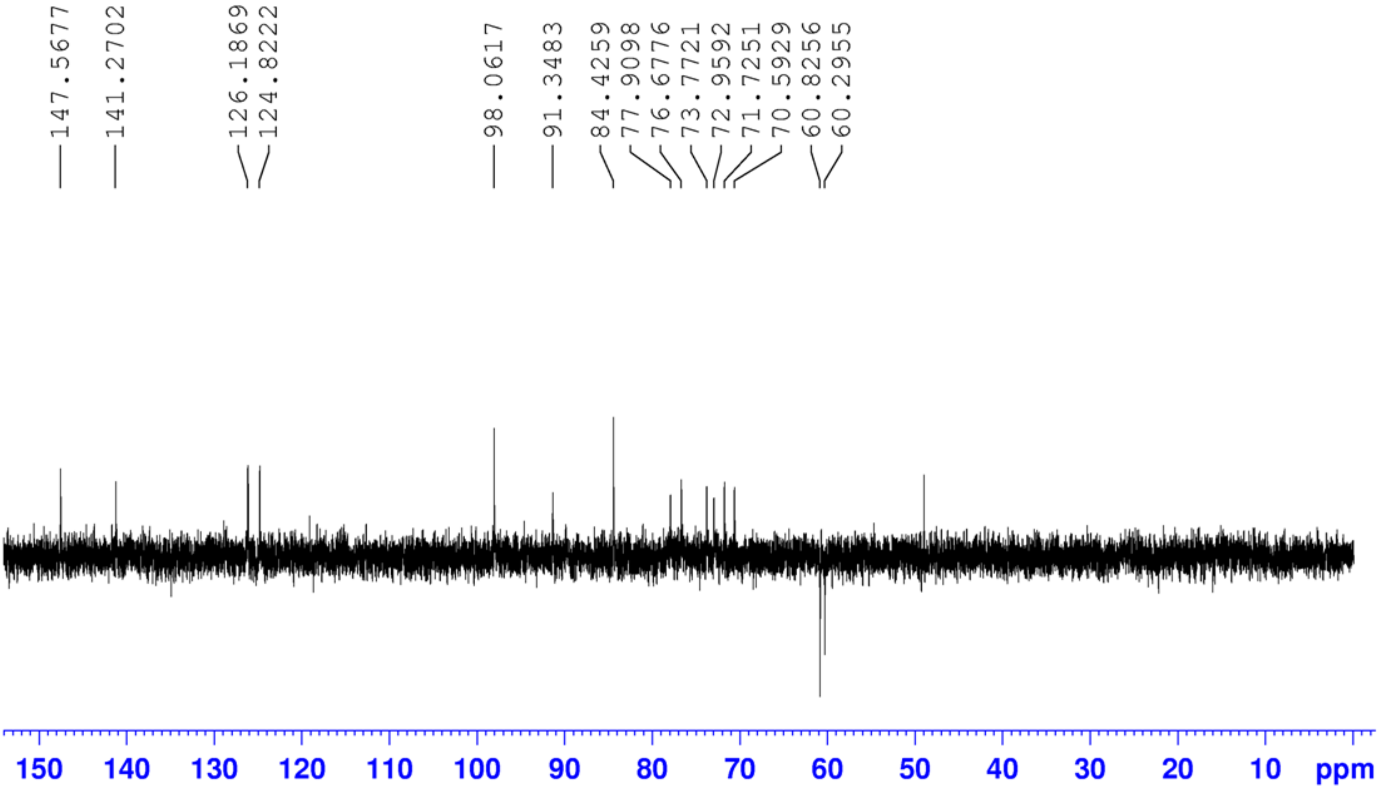

^{31}P NMR of 10d in D_2O

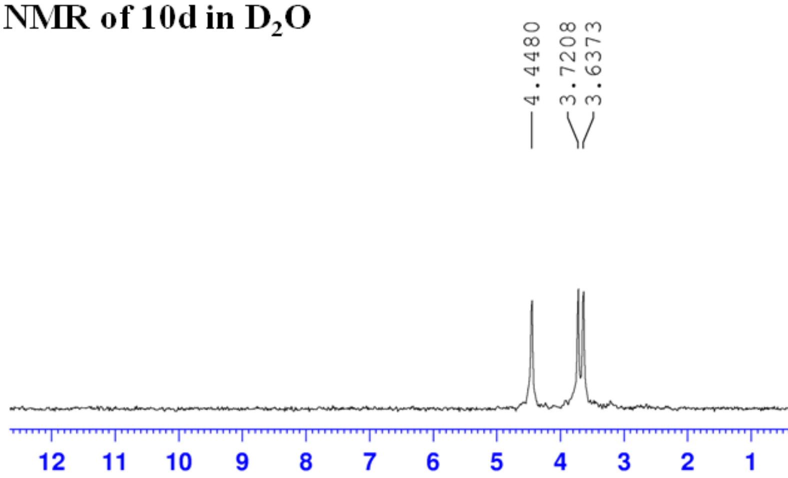

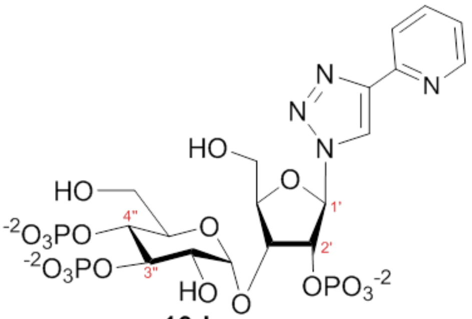

10d

HMBC of 10d in D_2O

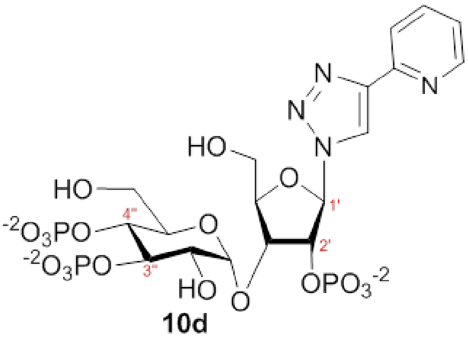

10d

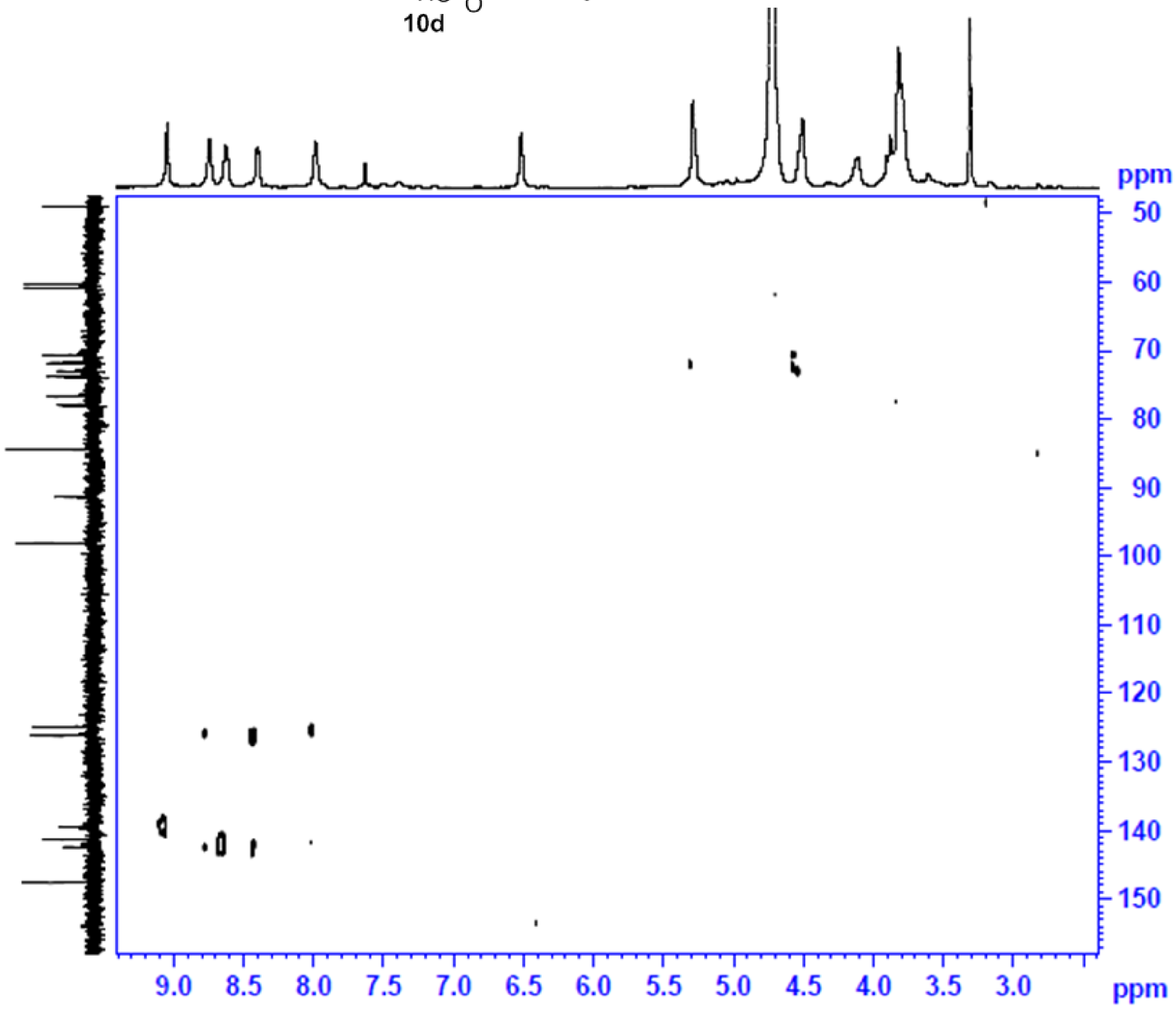

HMQC of 10d in D_2O

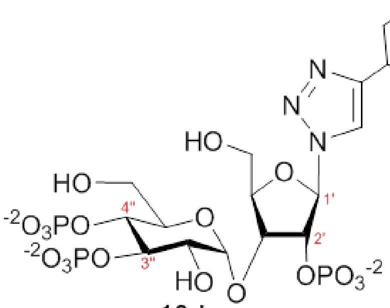

10d

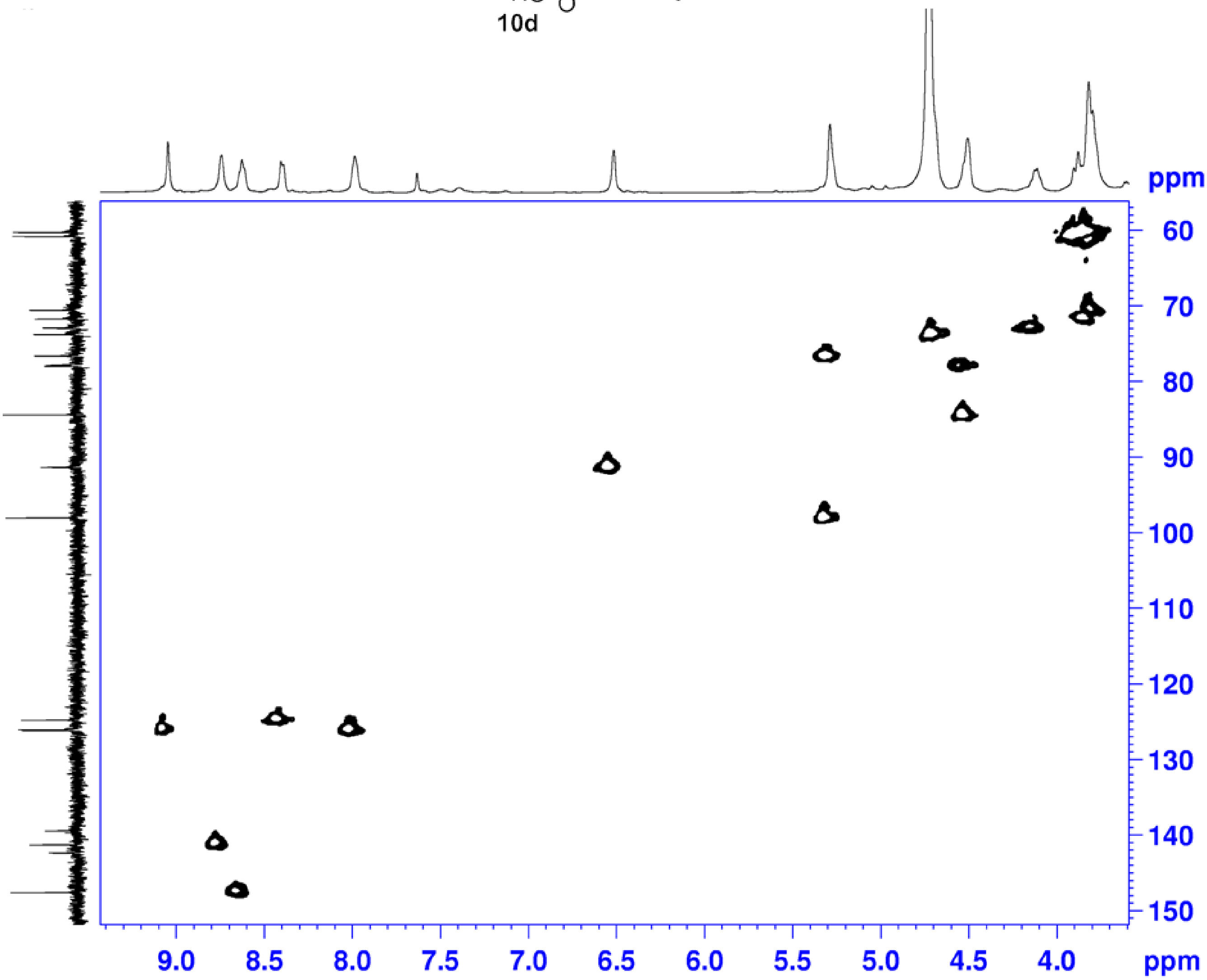

^{1}H NMR of 9e in CDCl$_3$

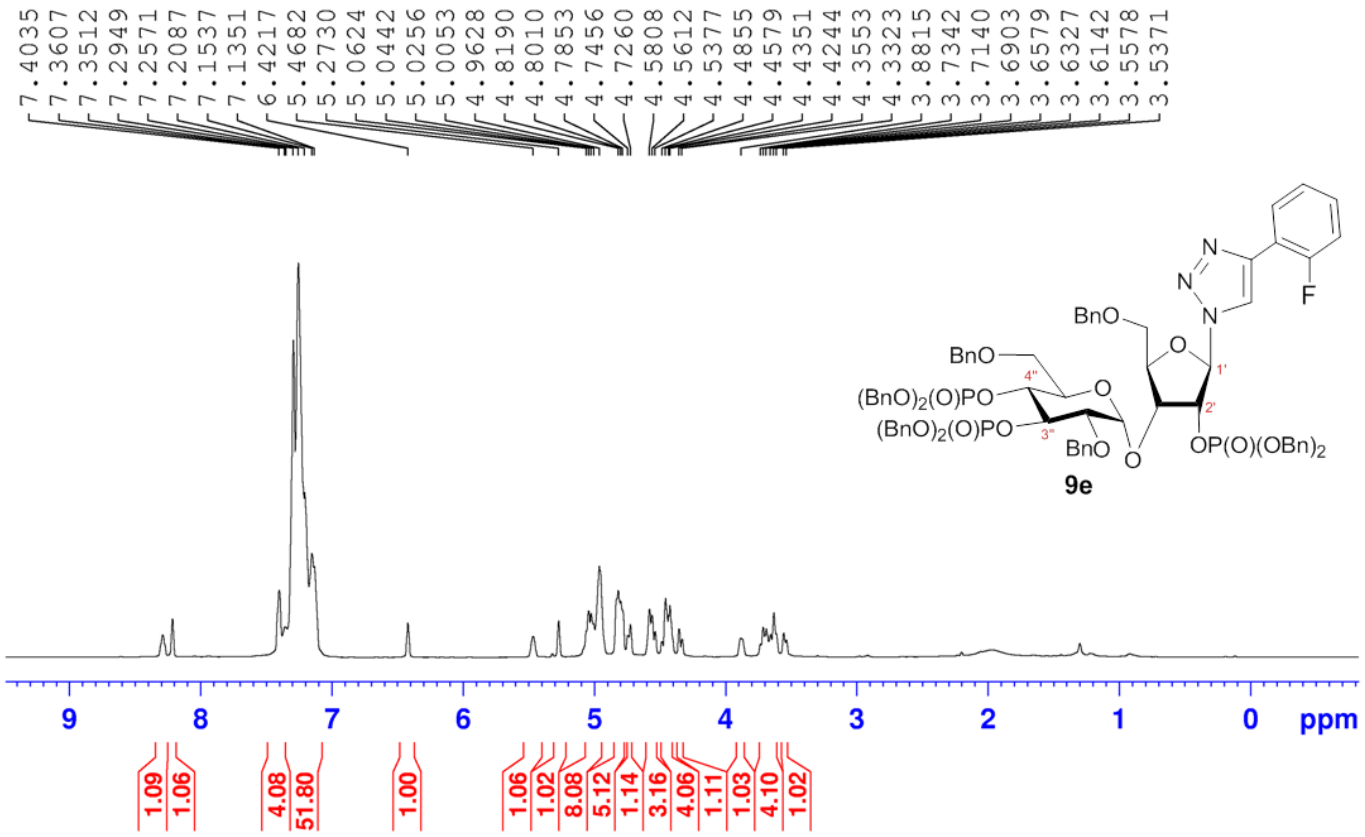

zoom

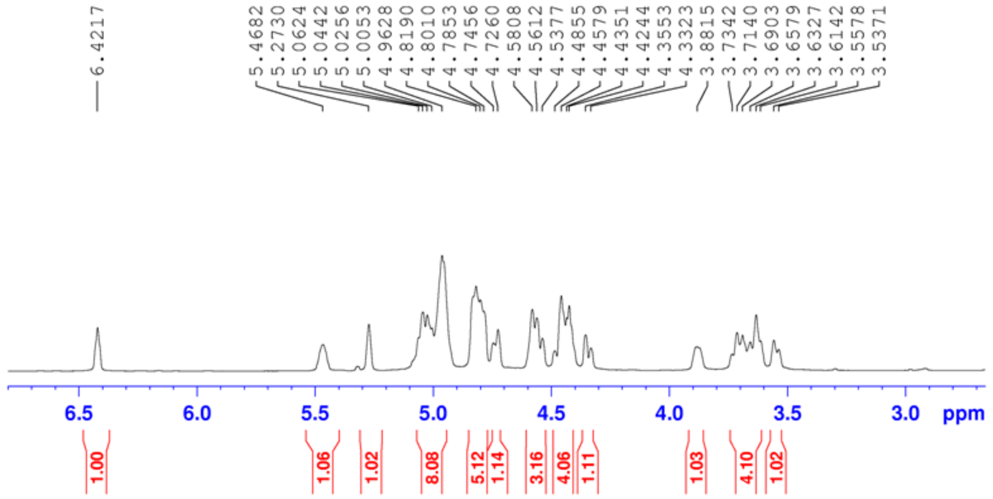

COSY of 9e in $CDCl_3$

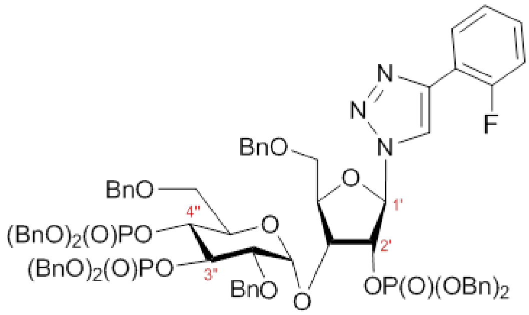

9e

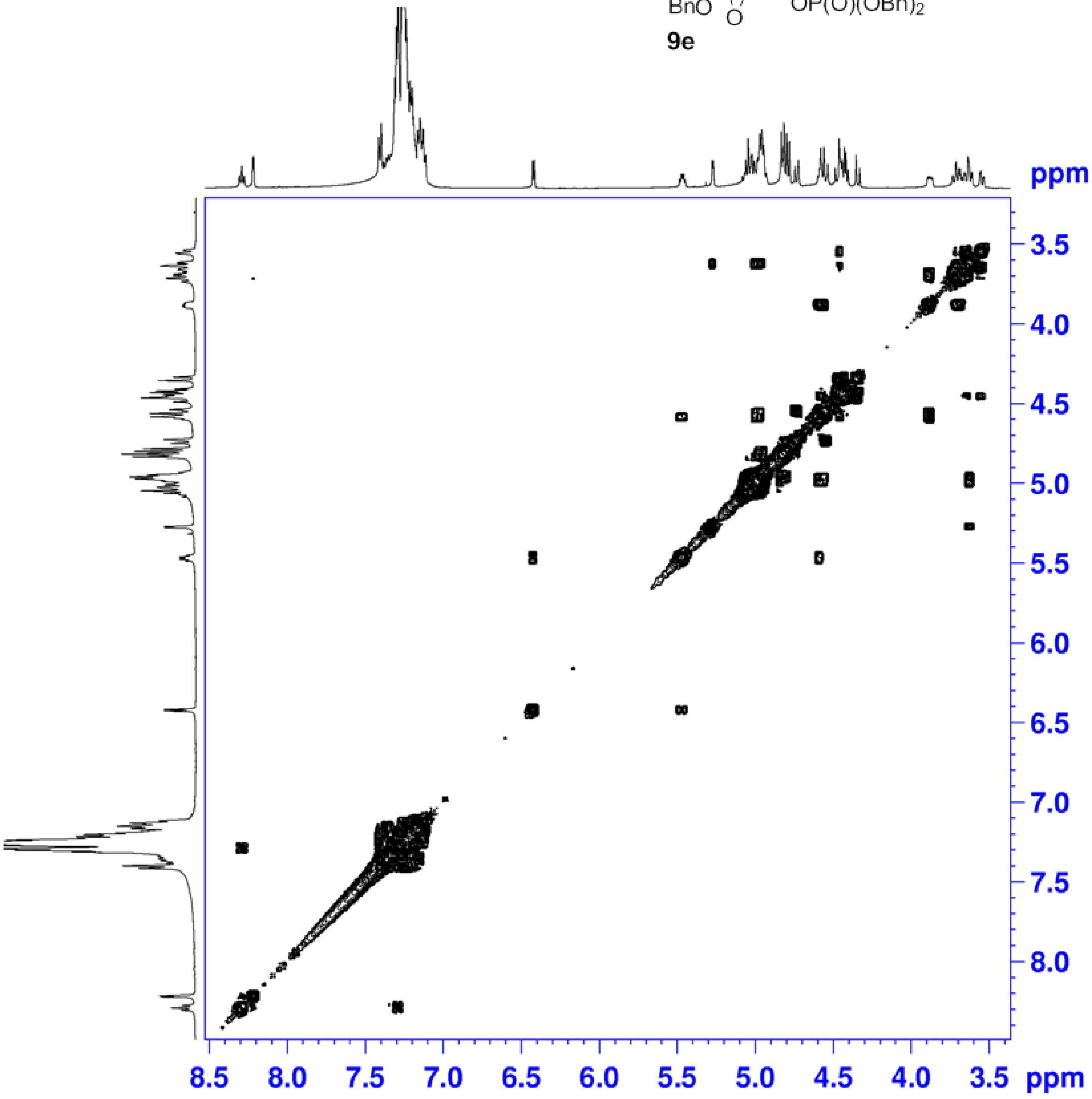

^{13}C NMR of 9e in $CDCl_3$

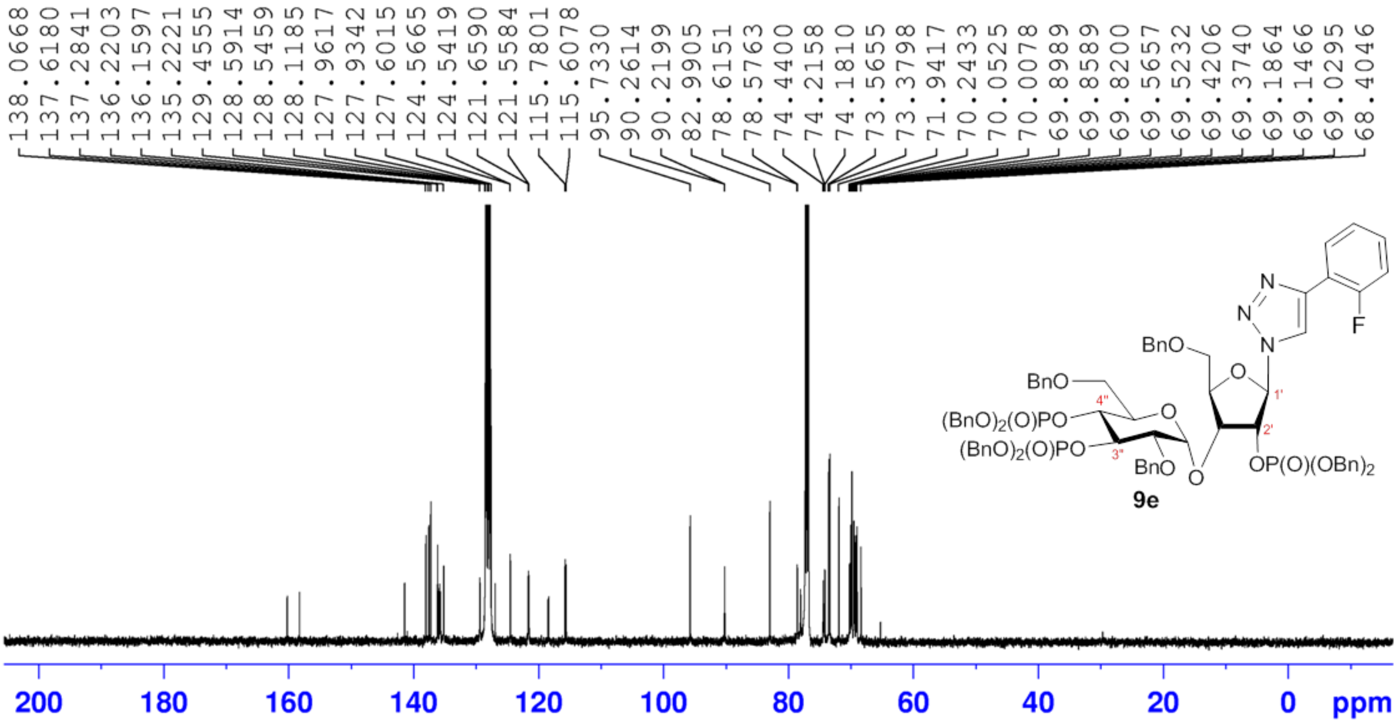

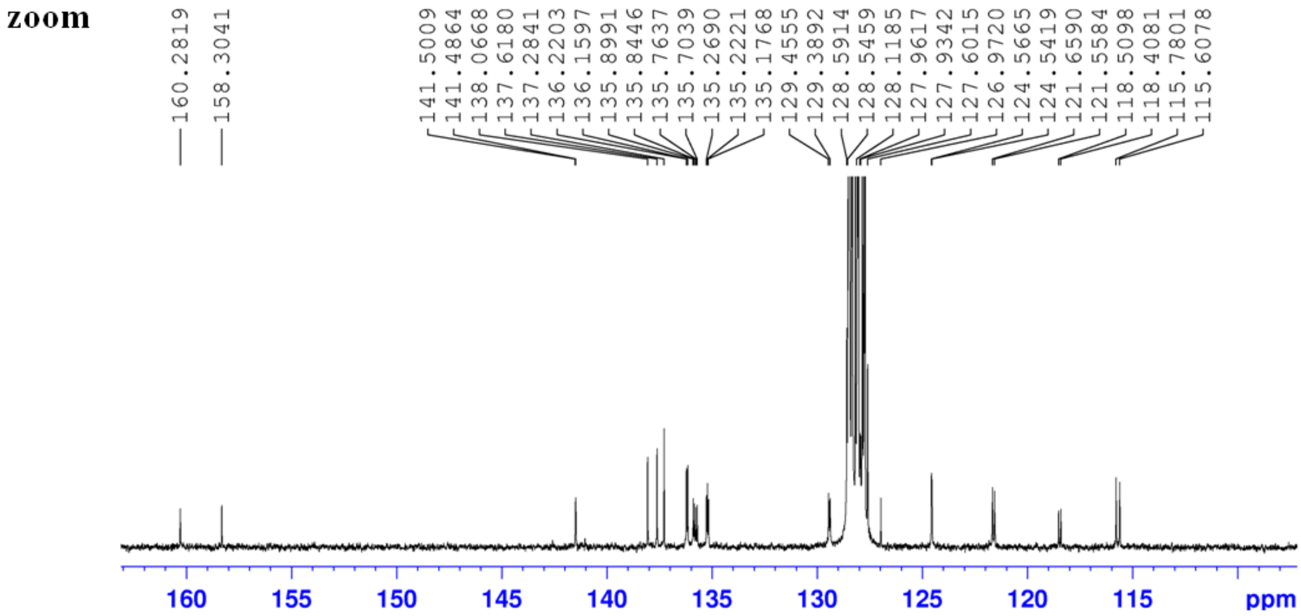

DEPT of 9e in $CDCl_3$

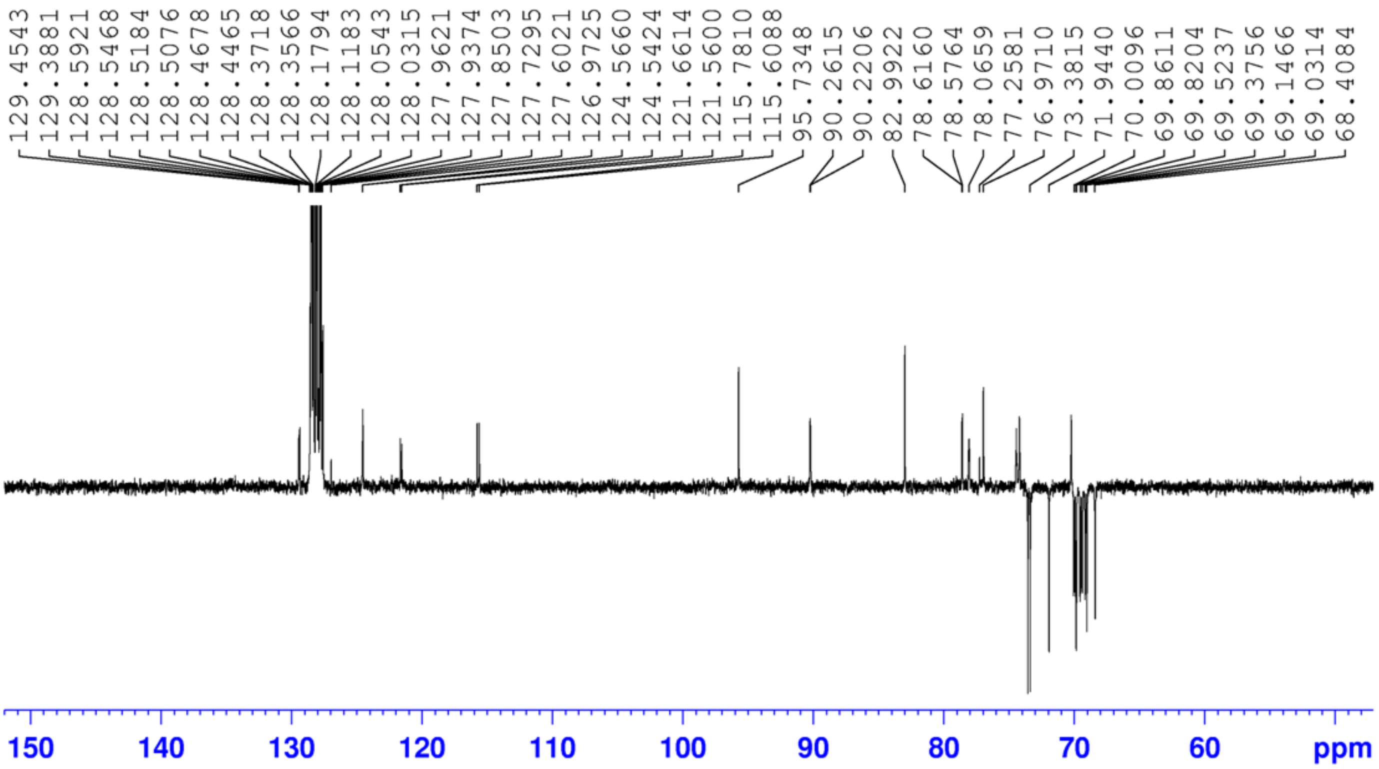

^{31}P NMR of 9e in $CDCl_3$

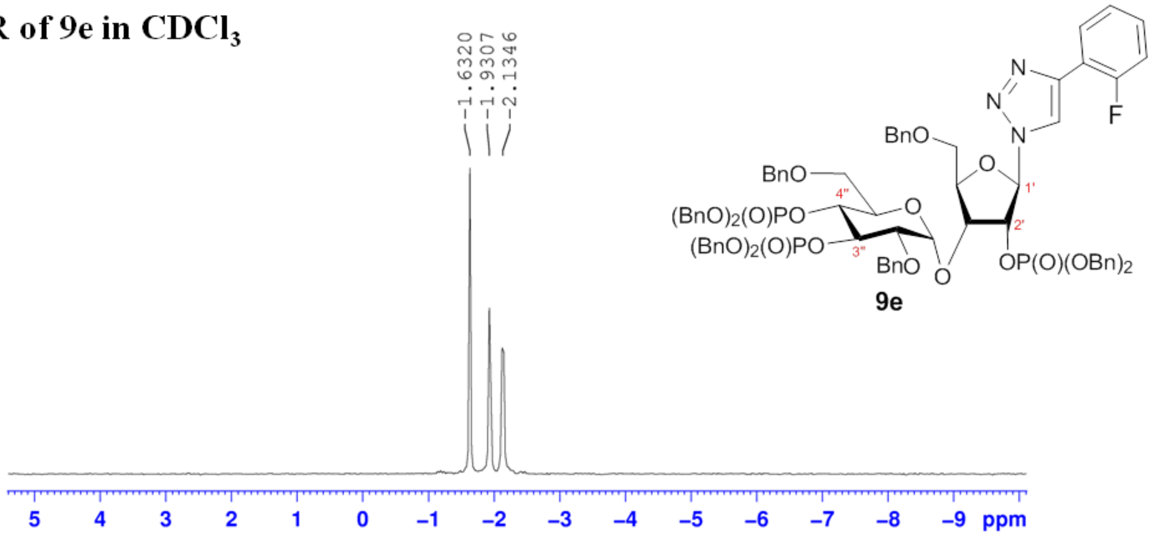

9e

^{19}F NMR of 9e in CDCl$_3$

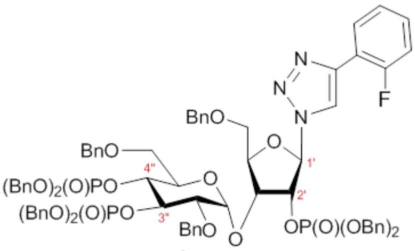

9e

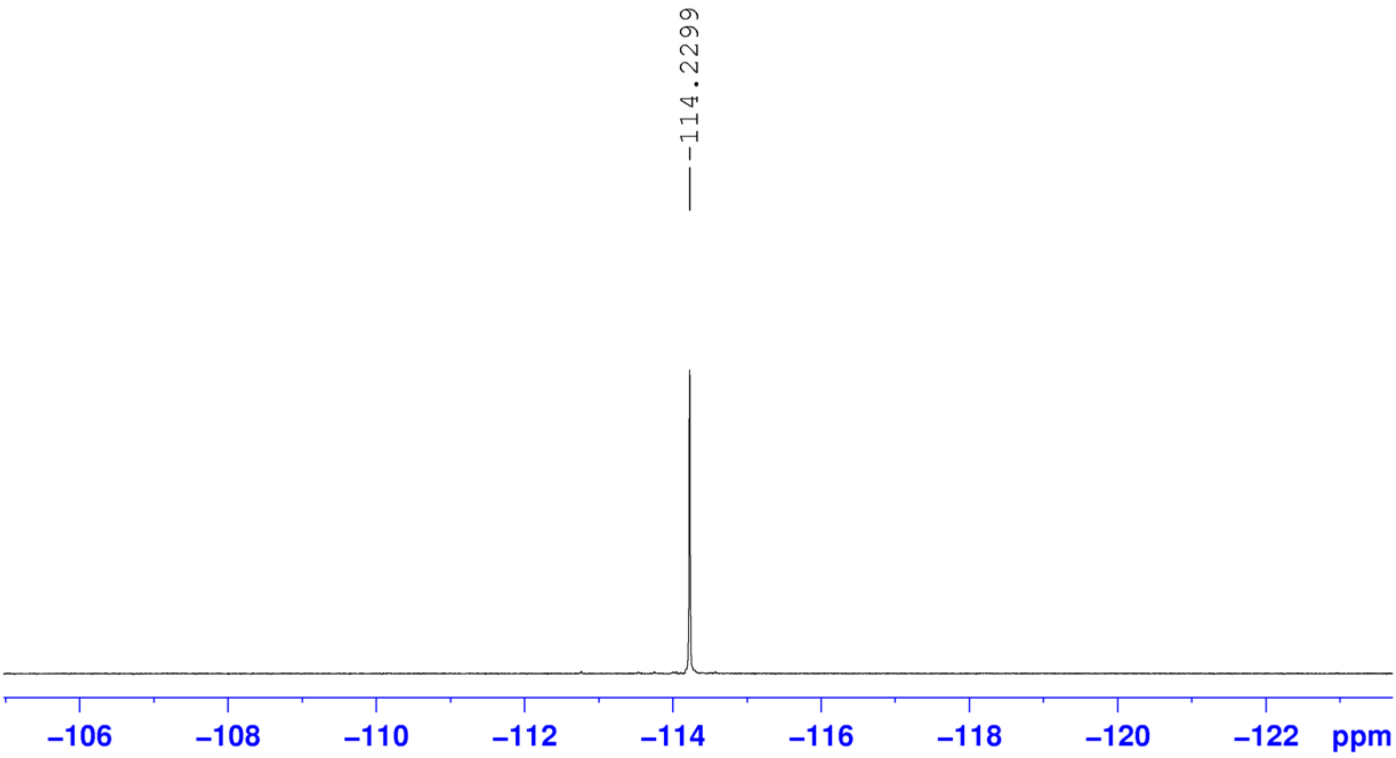

HMBC of 9e in $CDCl_3$

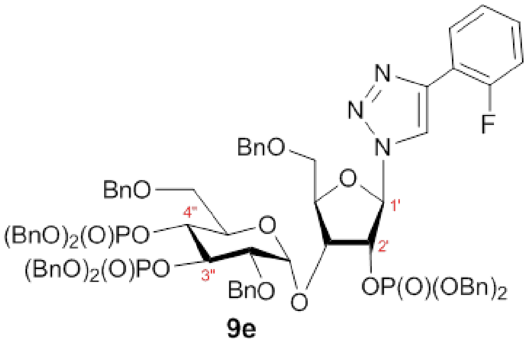

9e

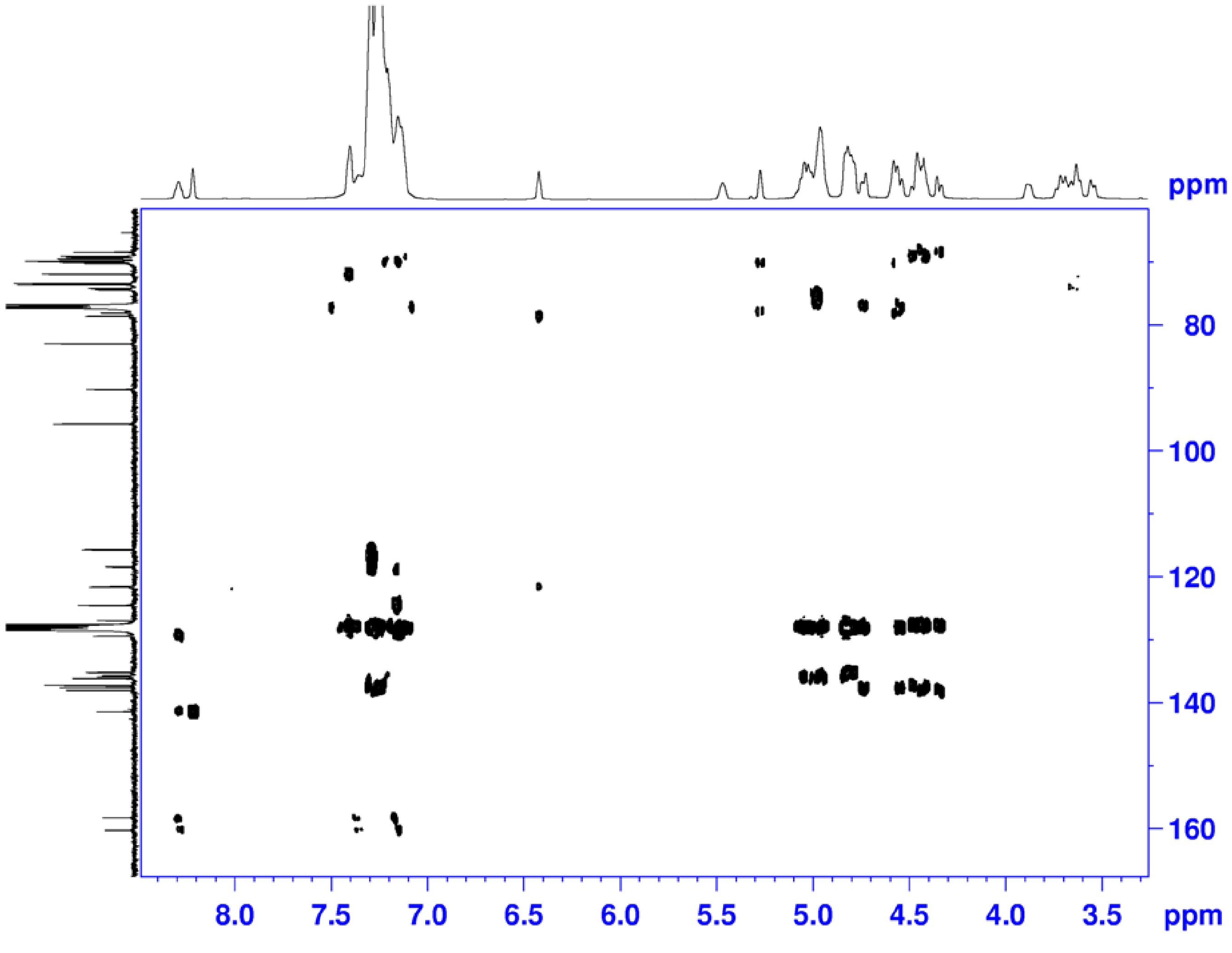

HMQC of 9e in $CDCl_3$

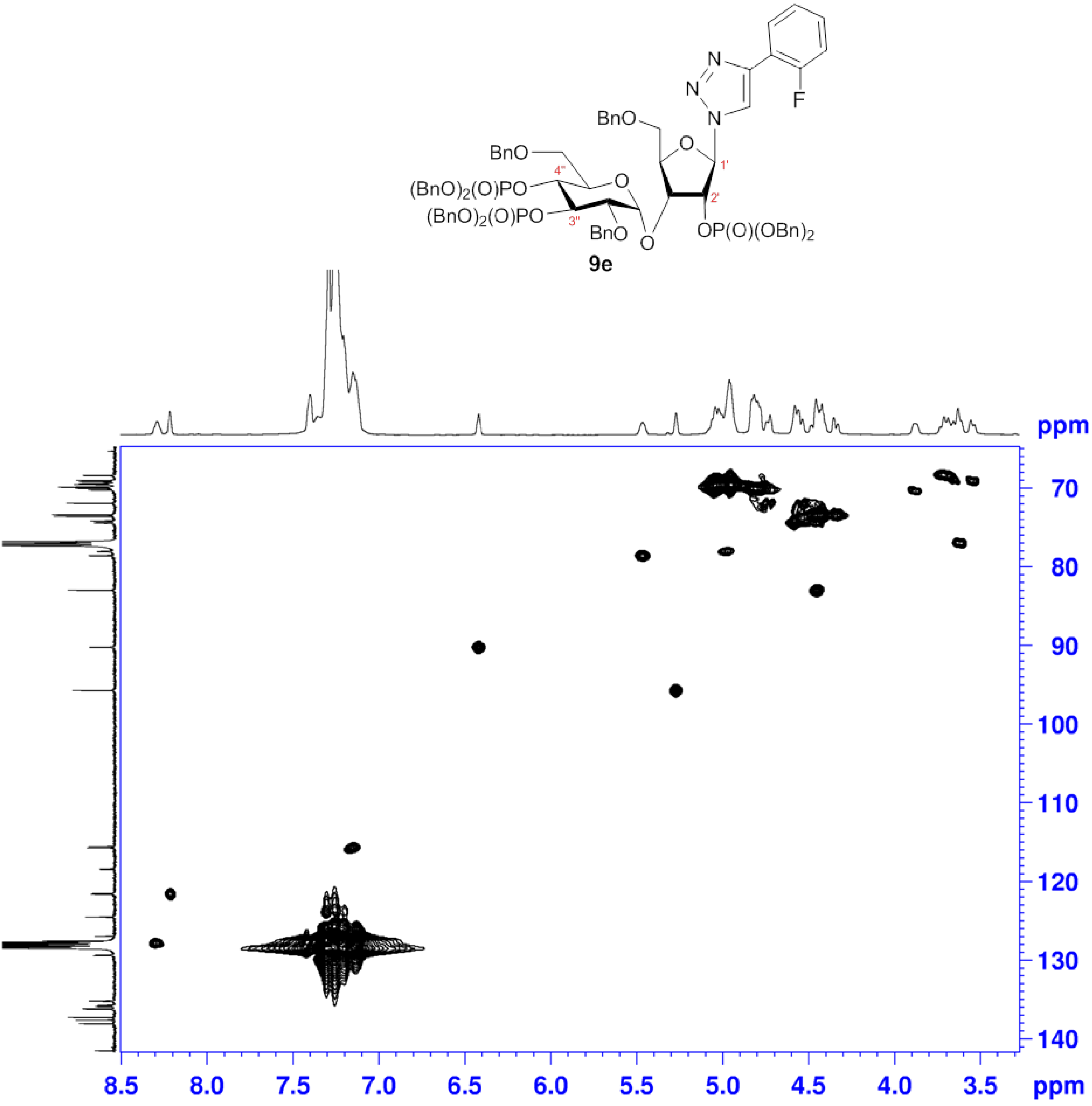

^{1}H NMR of 10e in MeOD

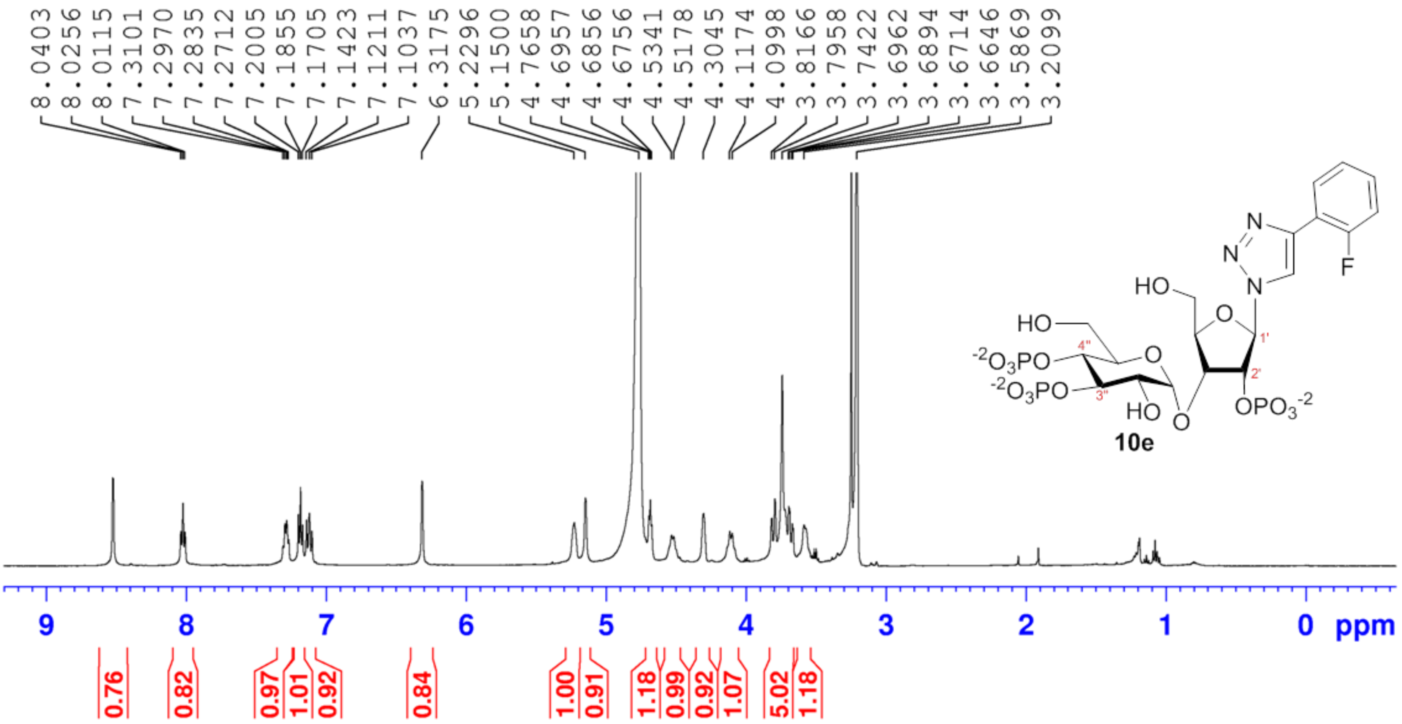

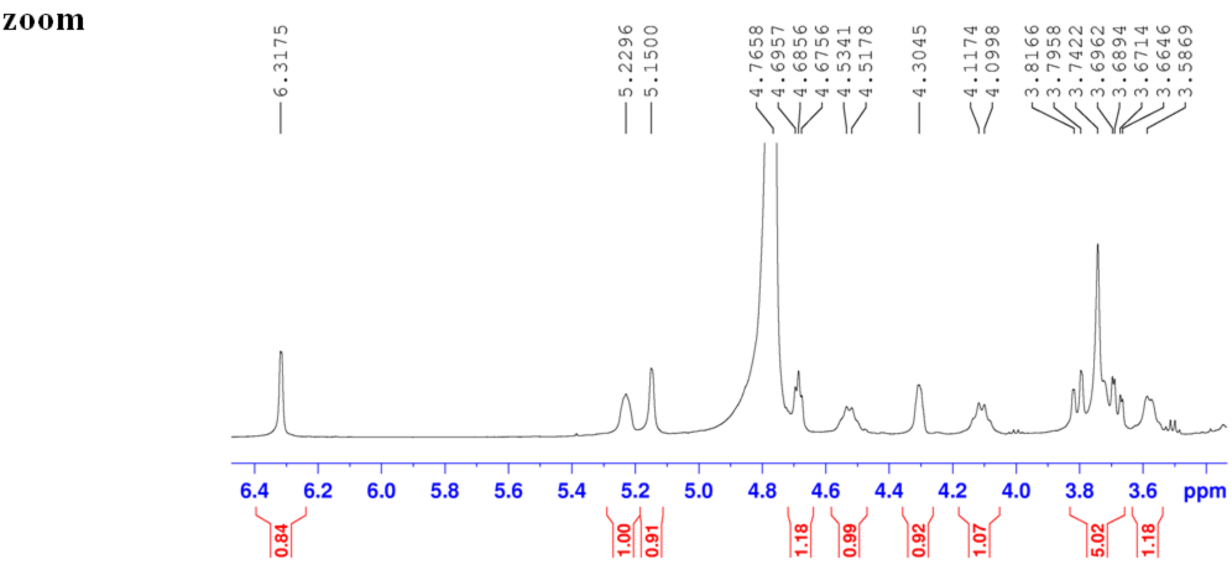

COSY of 10e in MeOD

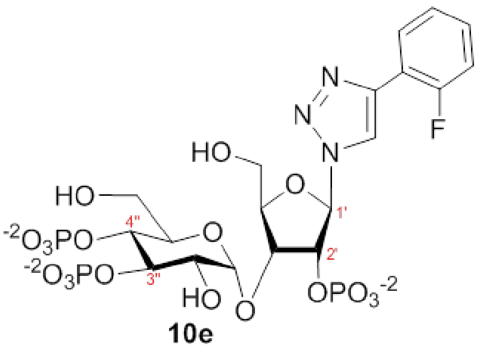

10e

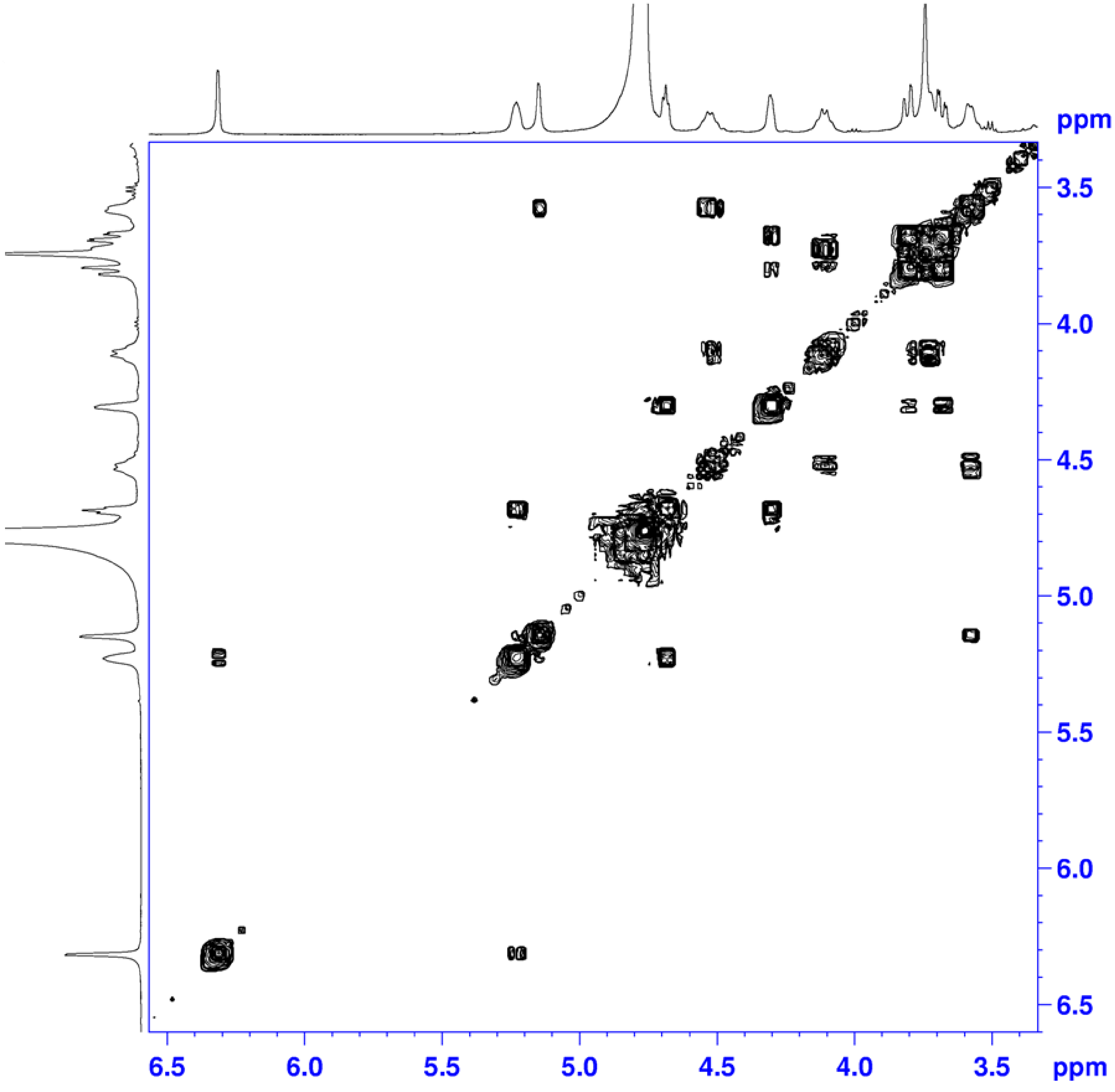

^{13}C NMR of 10e in MeOD

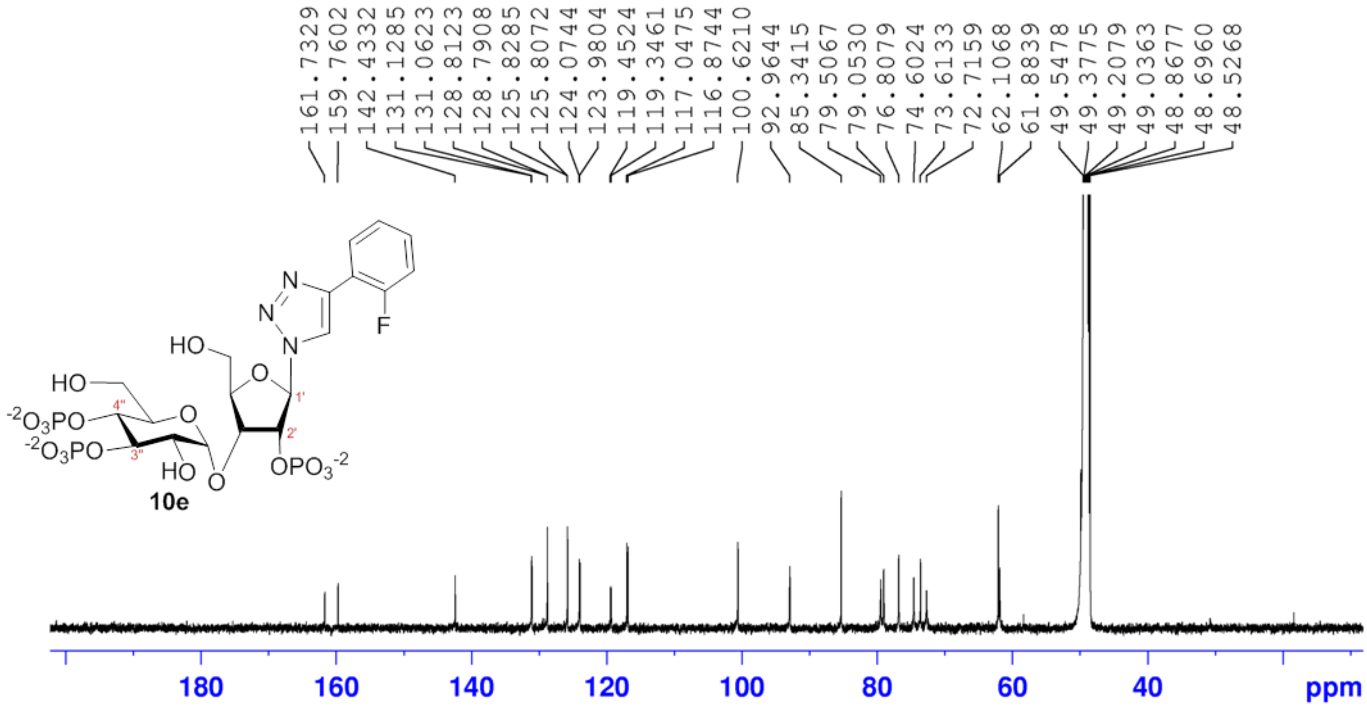

DEPT of 10e in MeOD

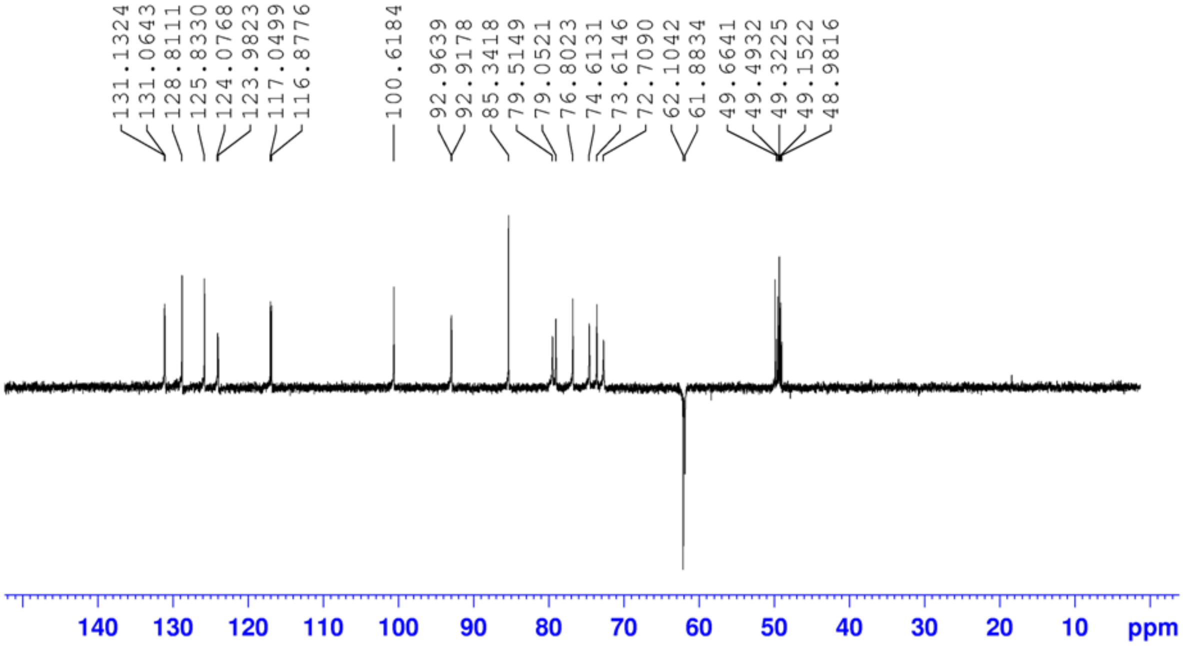

^{19}F NMR of 10e in MeOD

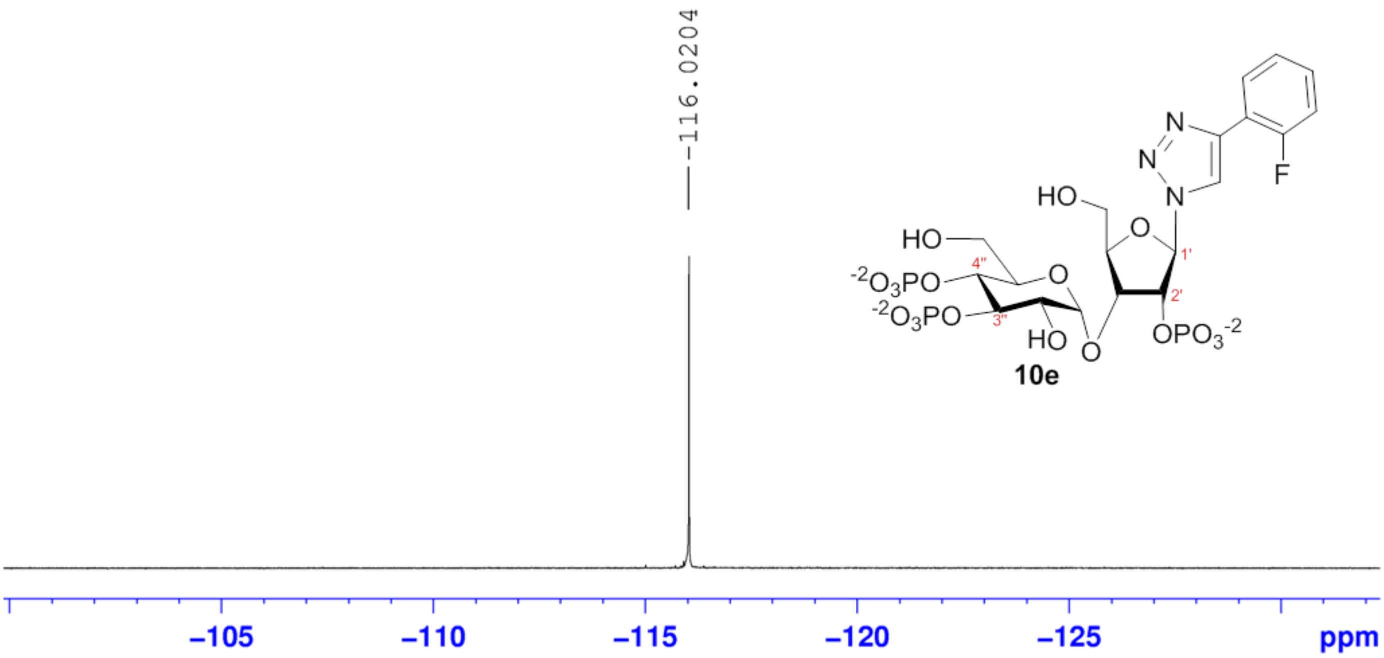

^{31}P NMR of 10e in MeOD

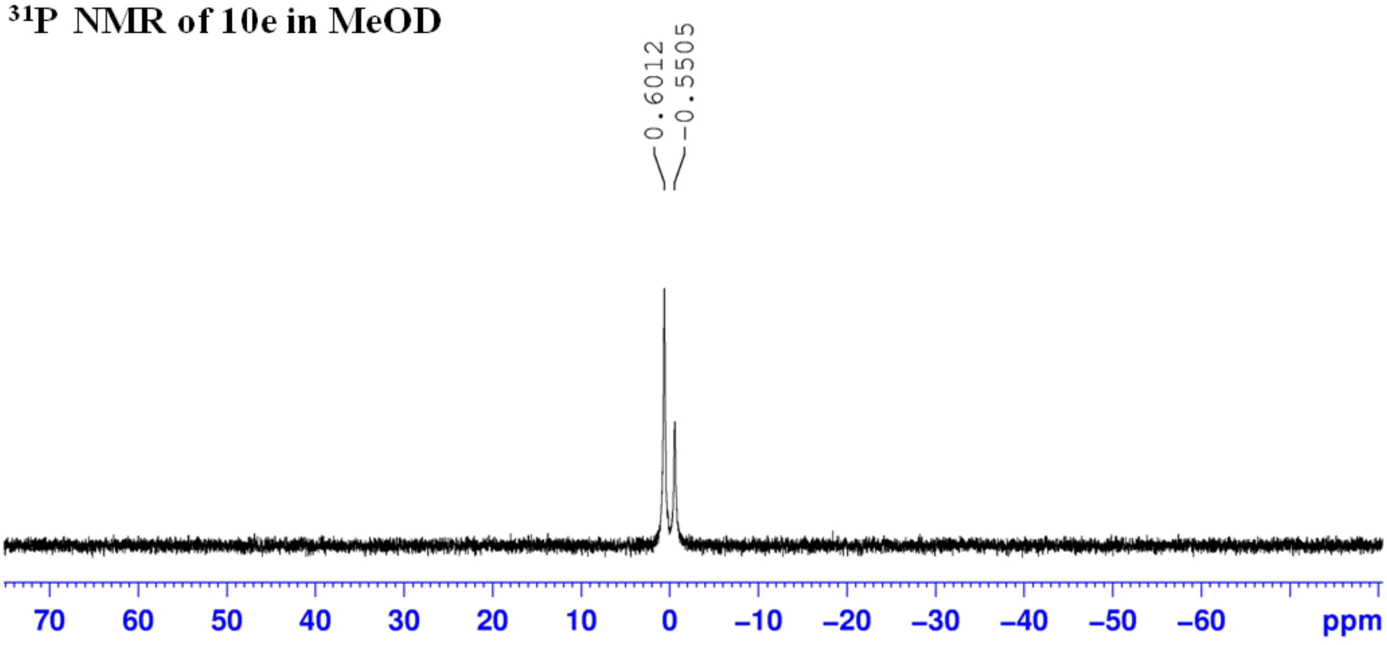

HMBC of 10e in MeOD

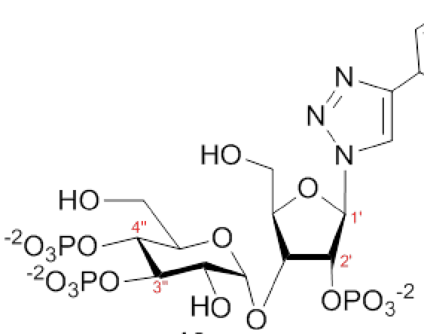

10e

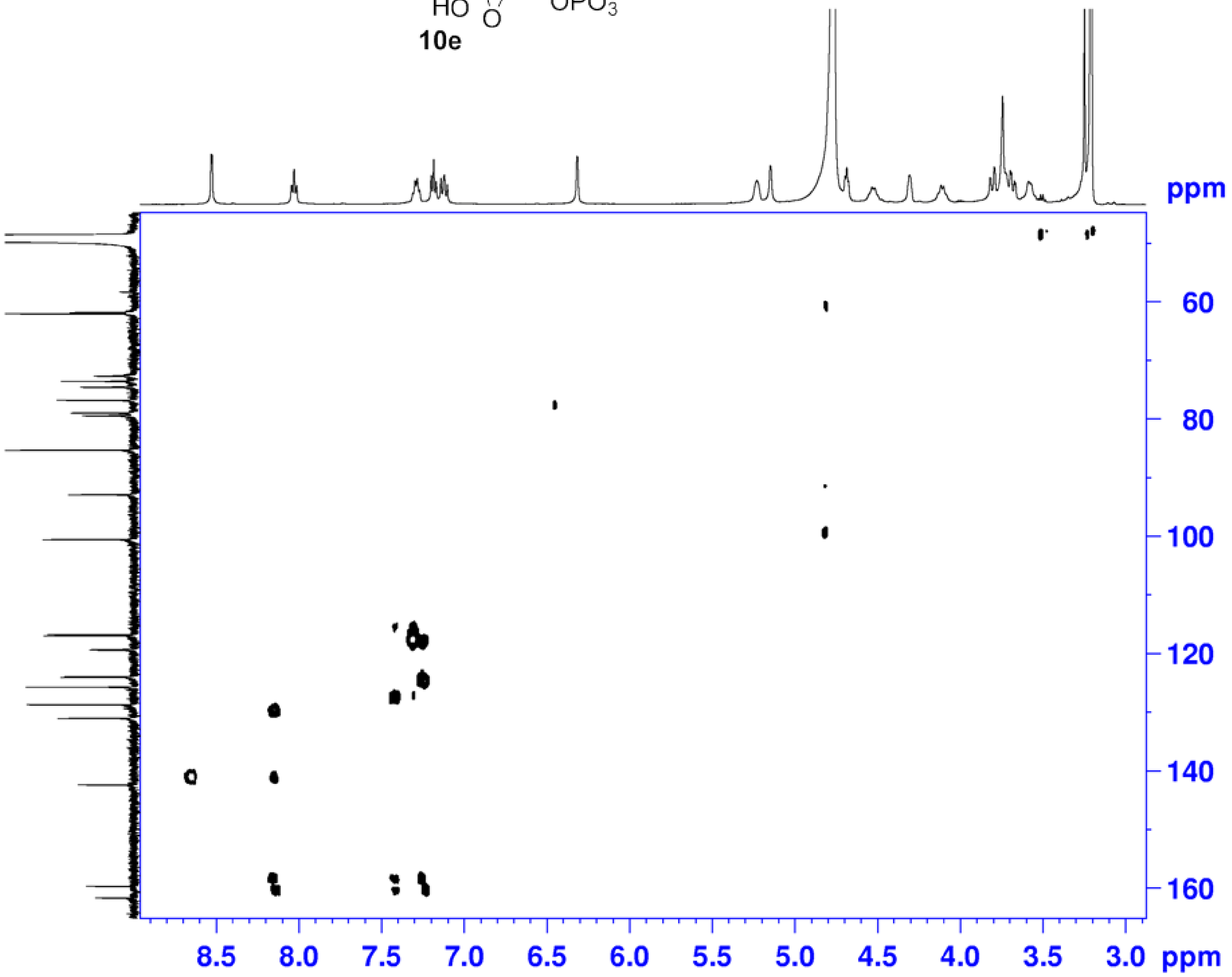

HMQC of 10e in MeOD

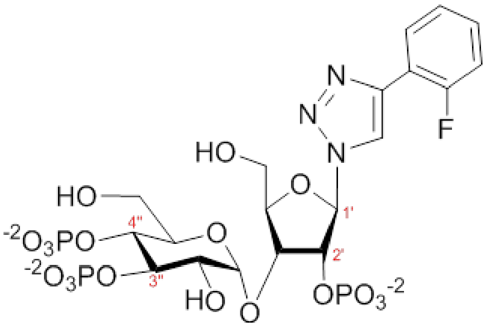

10e

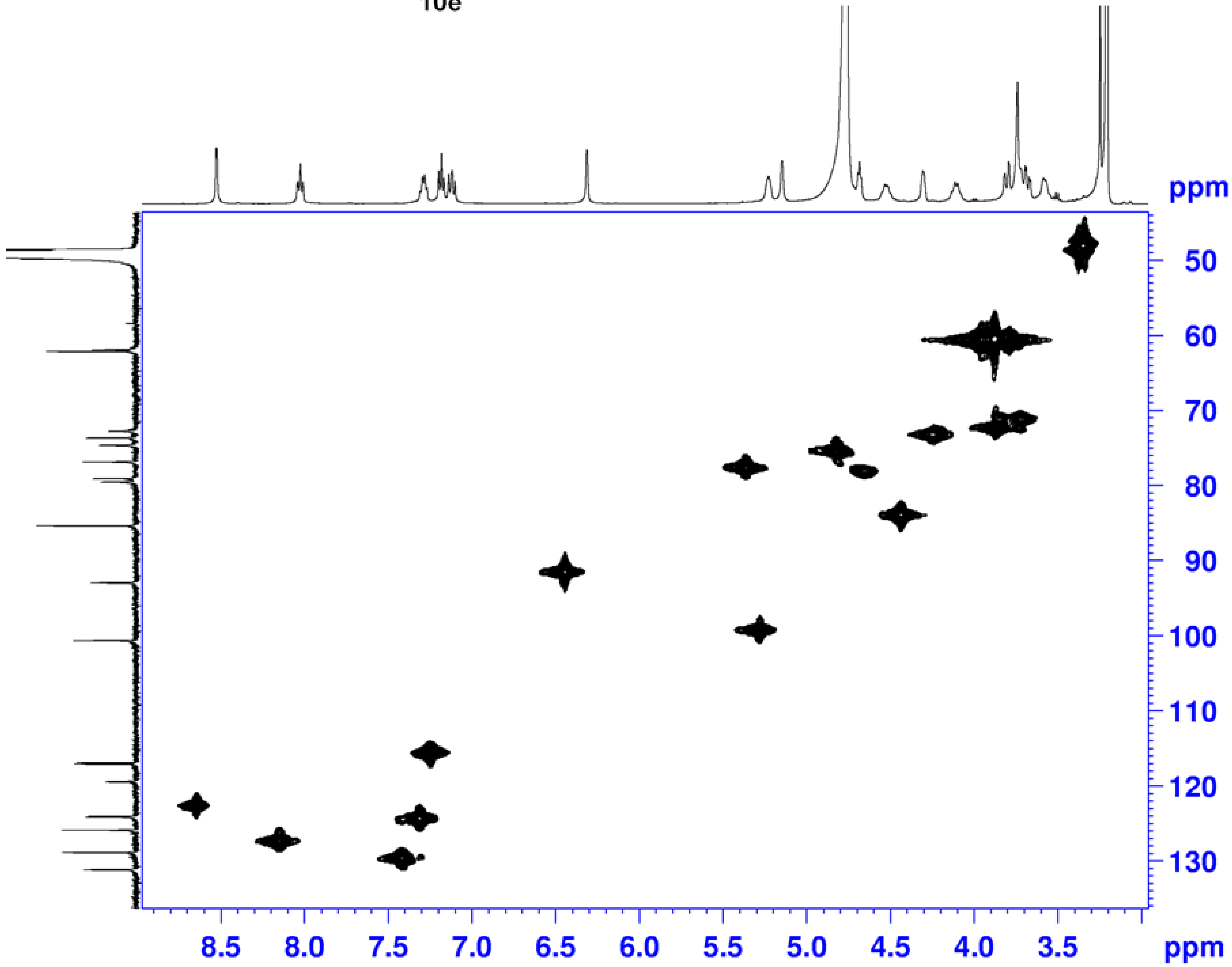

^{1}H NMR of 9f in $CDCl_3$

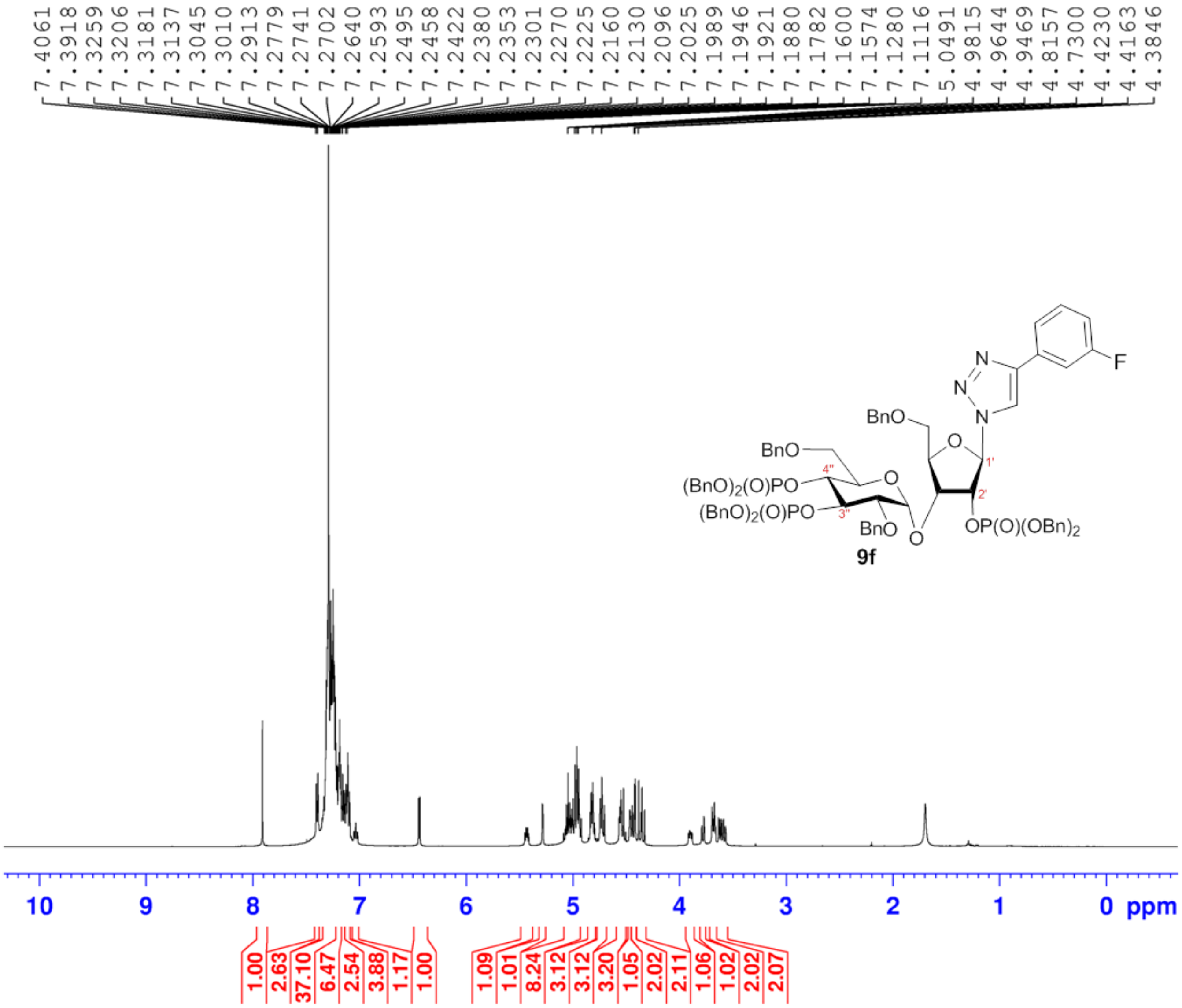

^{1}H NMR of 9f in $CDCl_3$ (zoom)

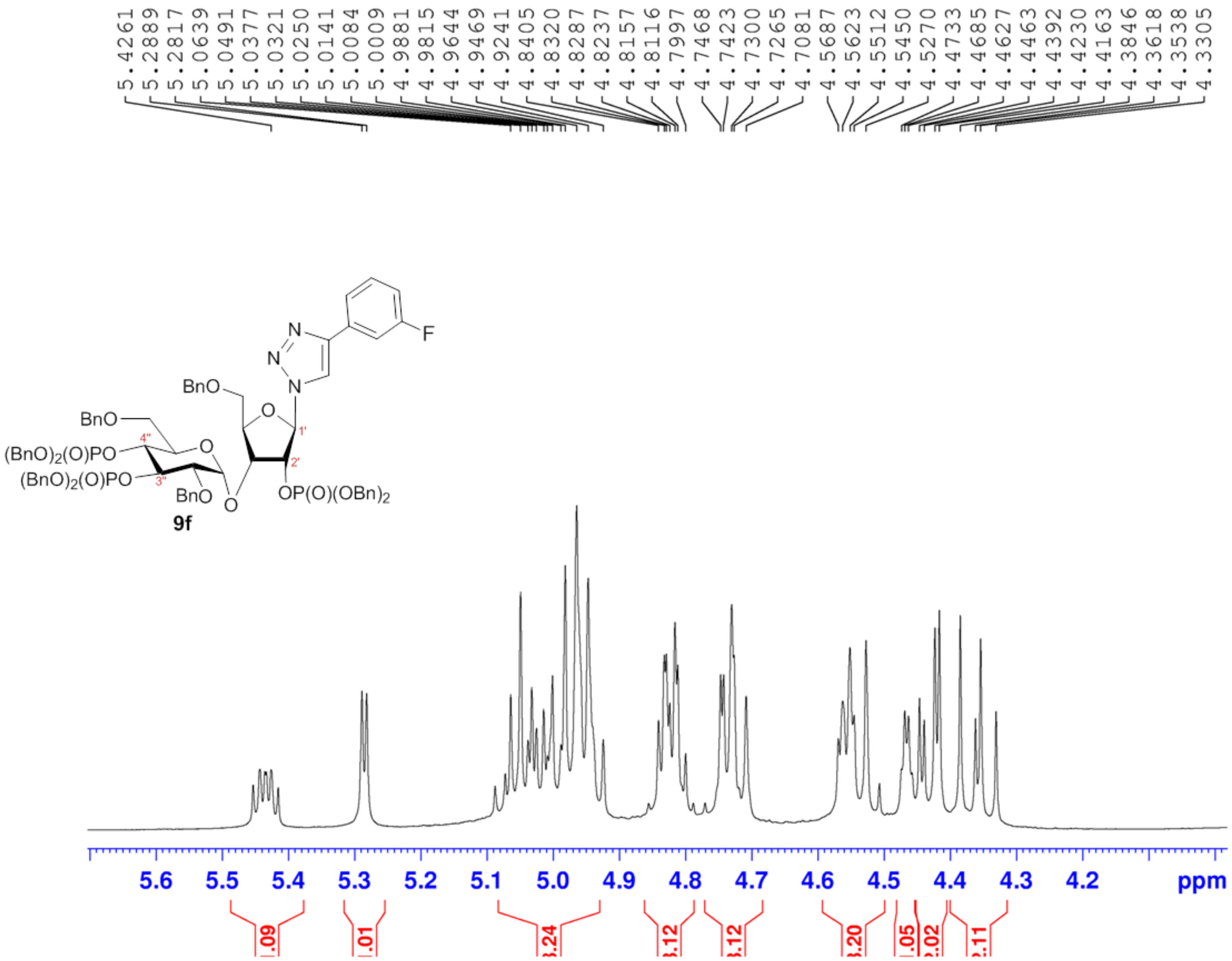

COSY of 9f in $CDCl_3$

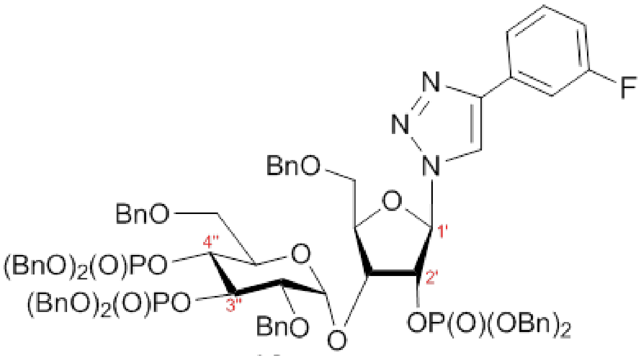

9f

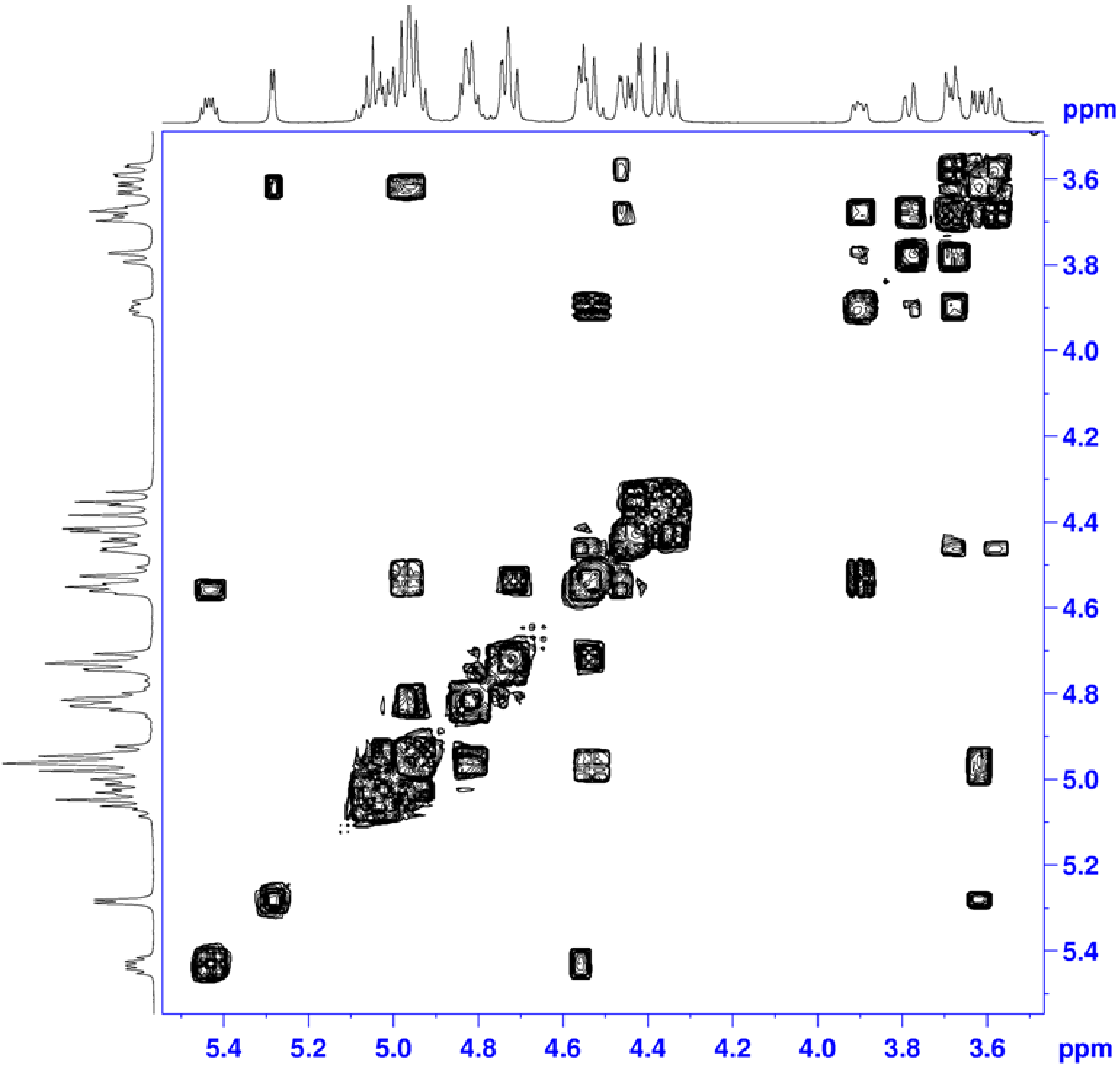

^{13}C NMR of 9f in $CDCl_3$

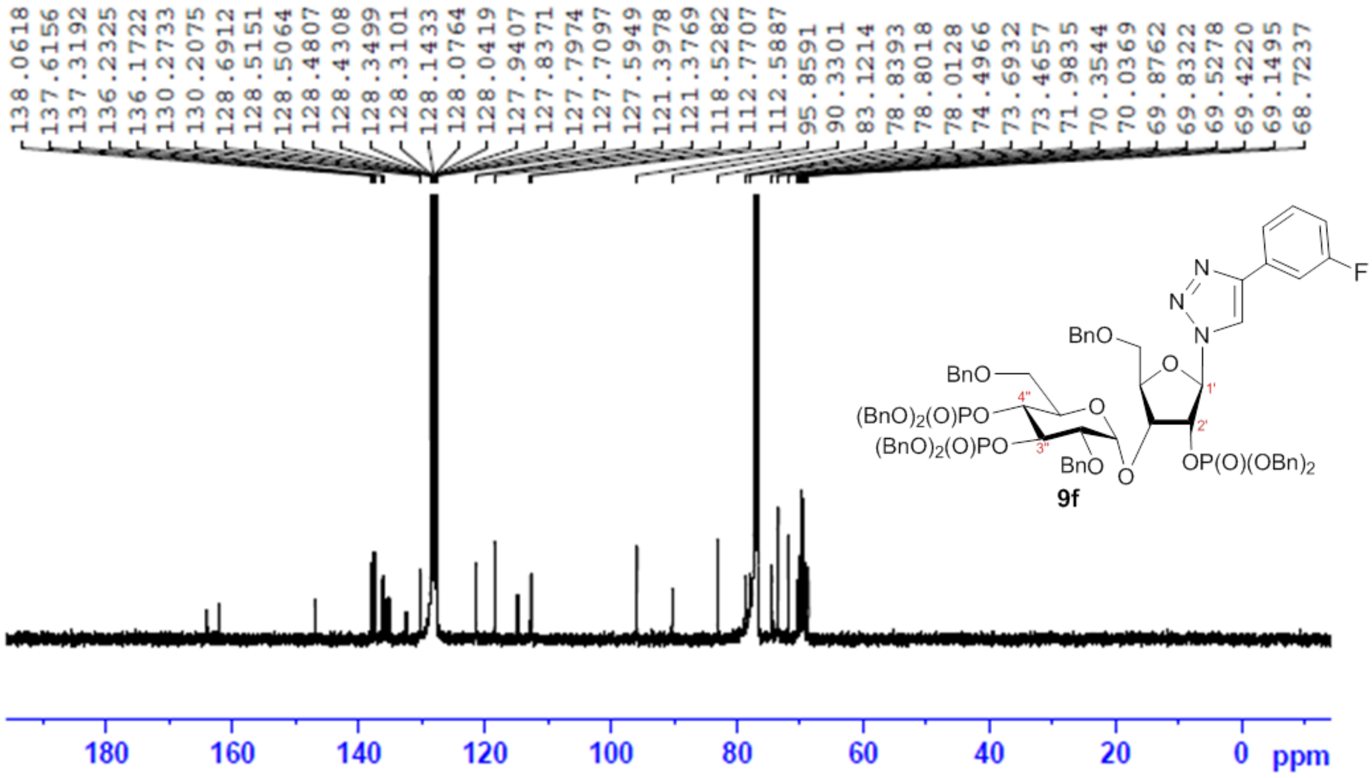

zoom

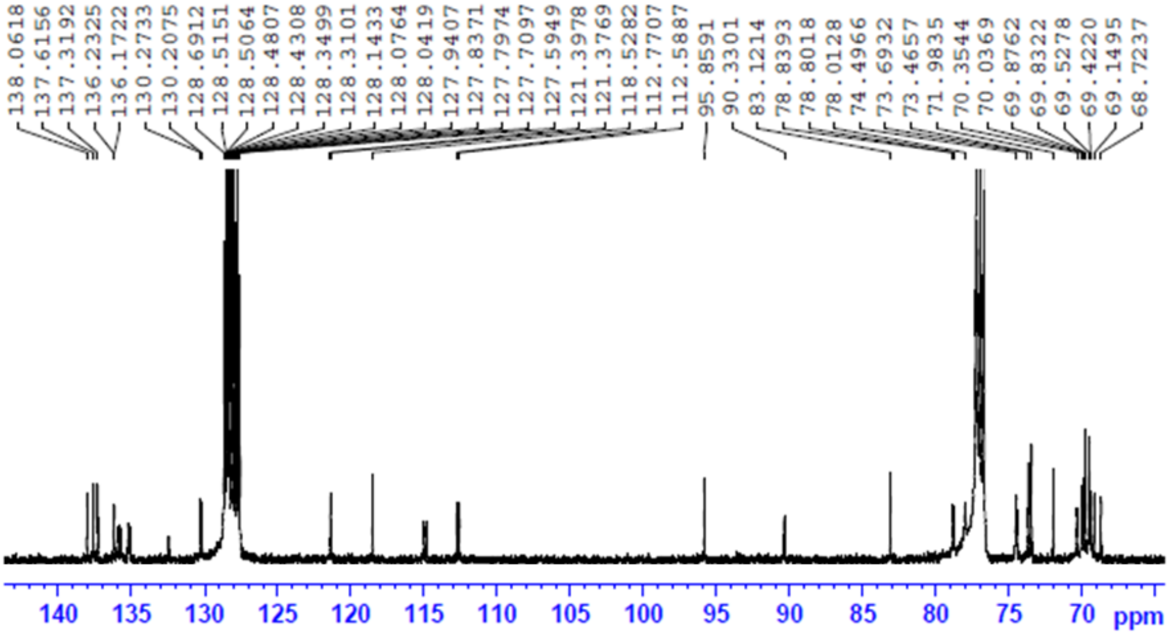

DEPT of 9f in $CDCl_3$

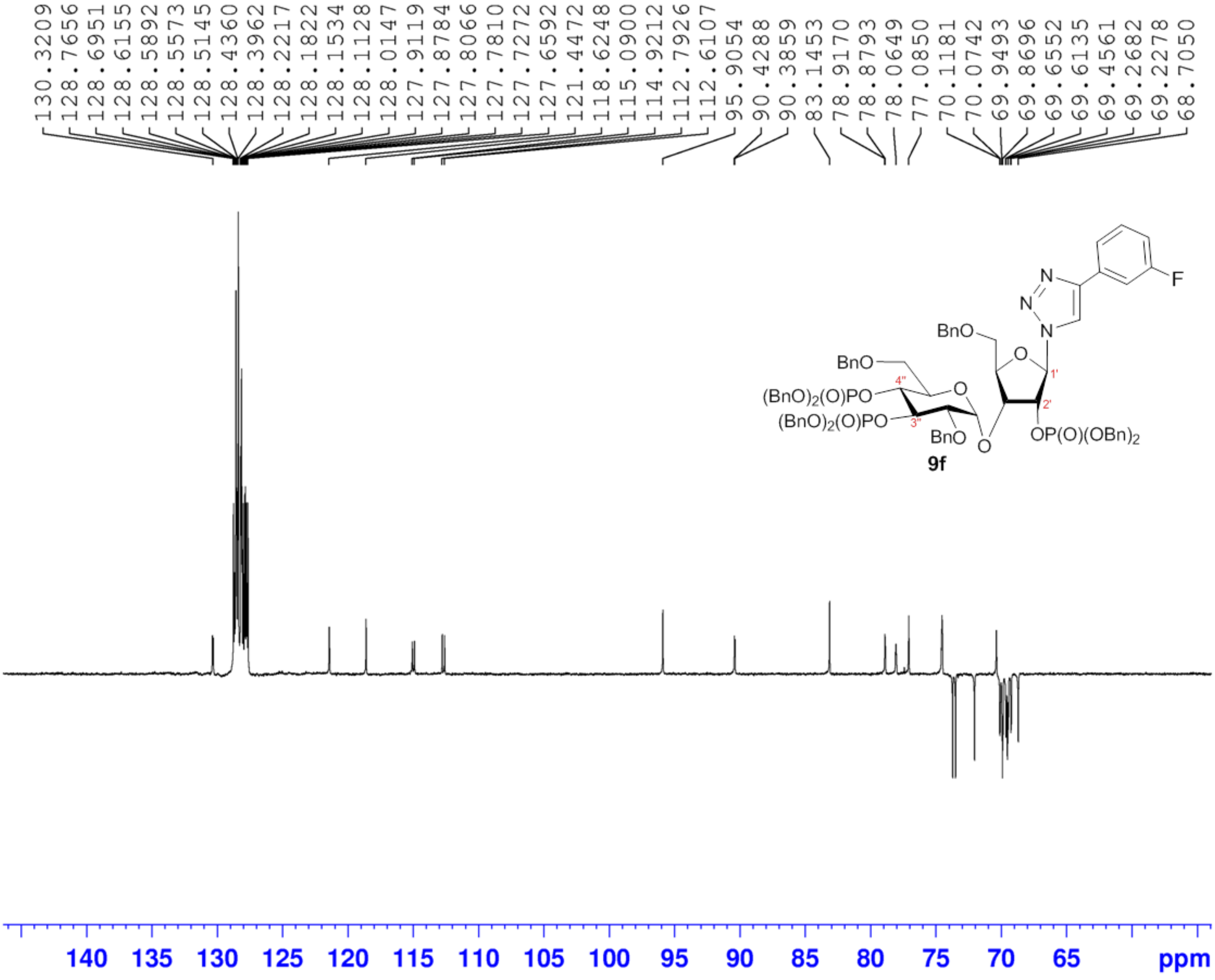

^{19}F NMR of 9f in $CDCl_3$

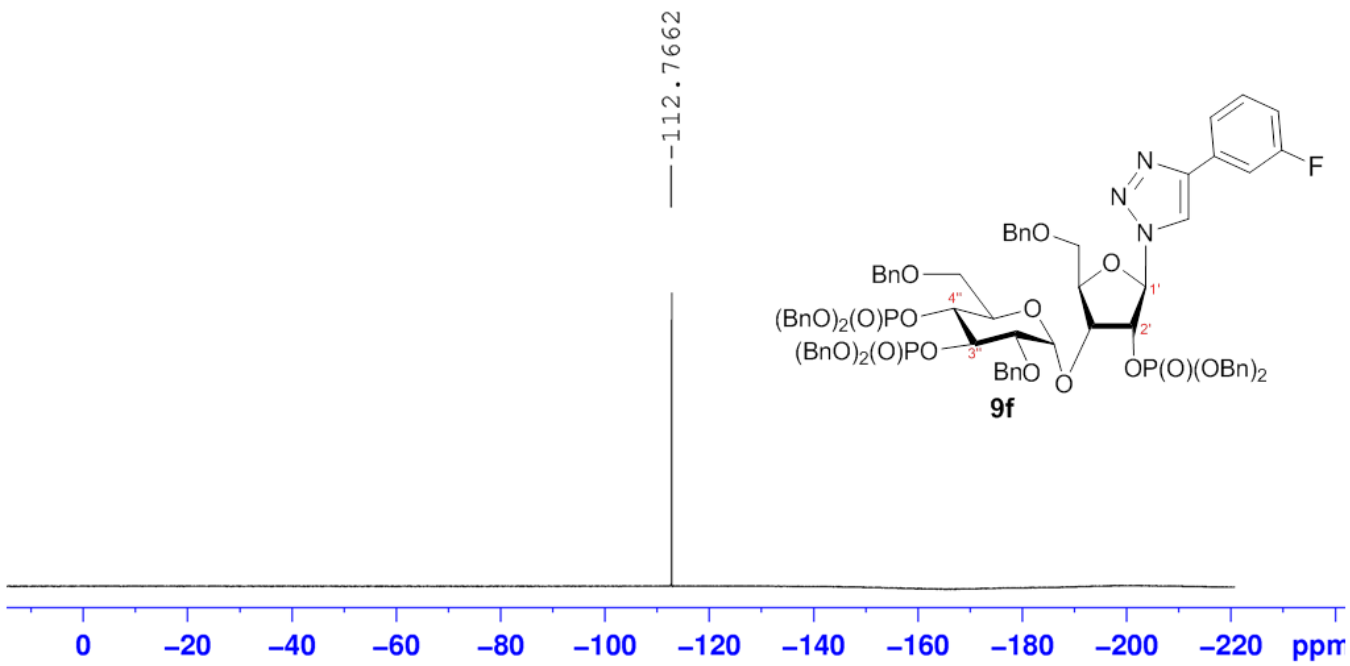

^{31}P NMR of 9f in $CDCl_3$

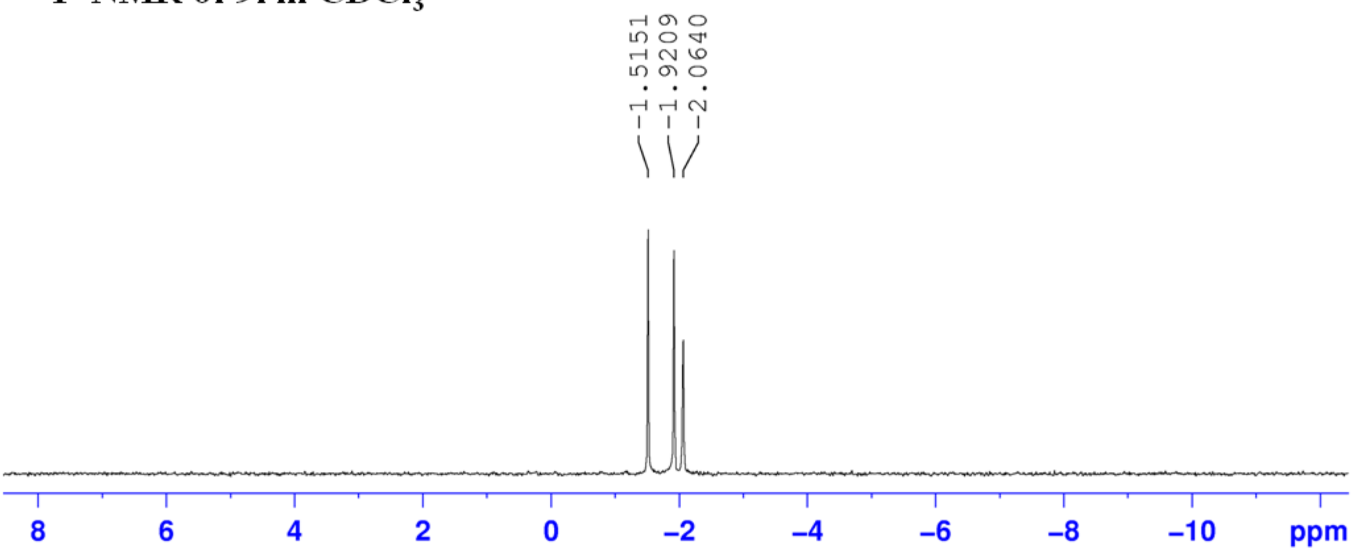

HMBC of 9f in $CDCl_3$

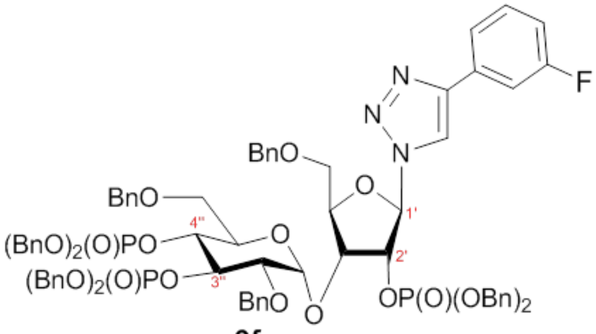

9f

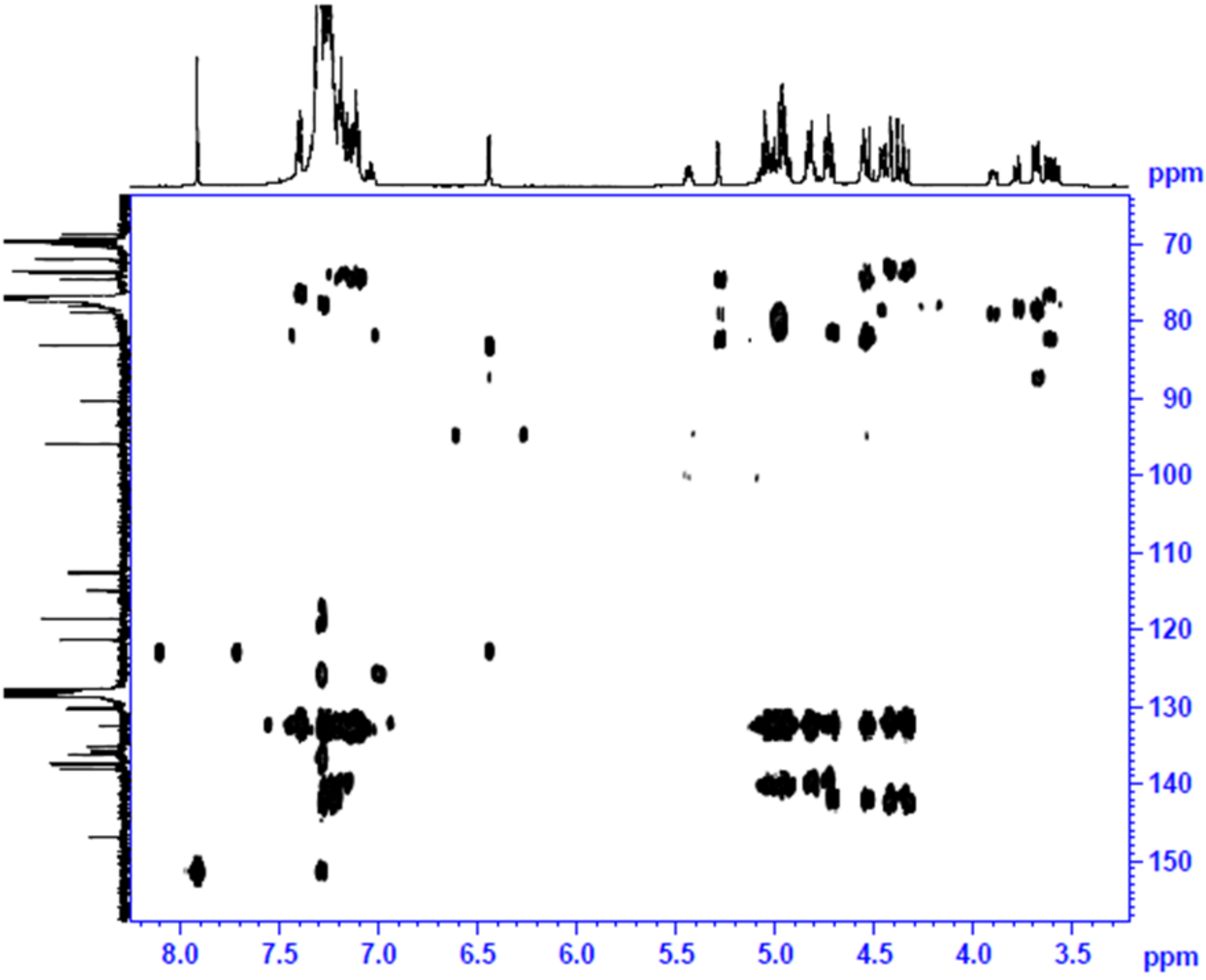

HMQC of 9f in $CDCl_3$

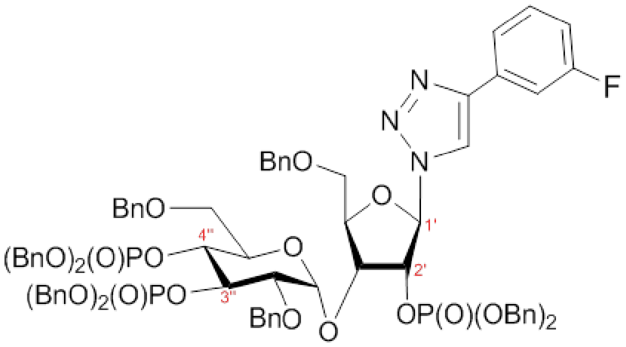

9f

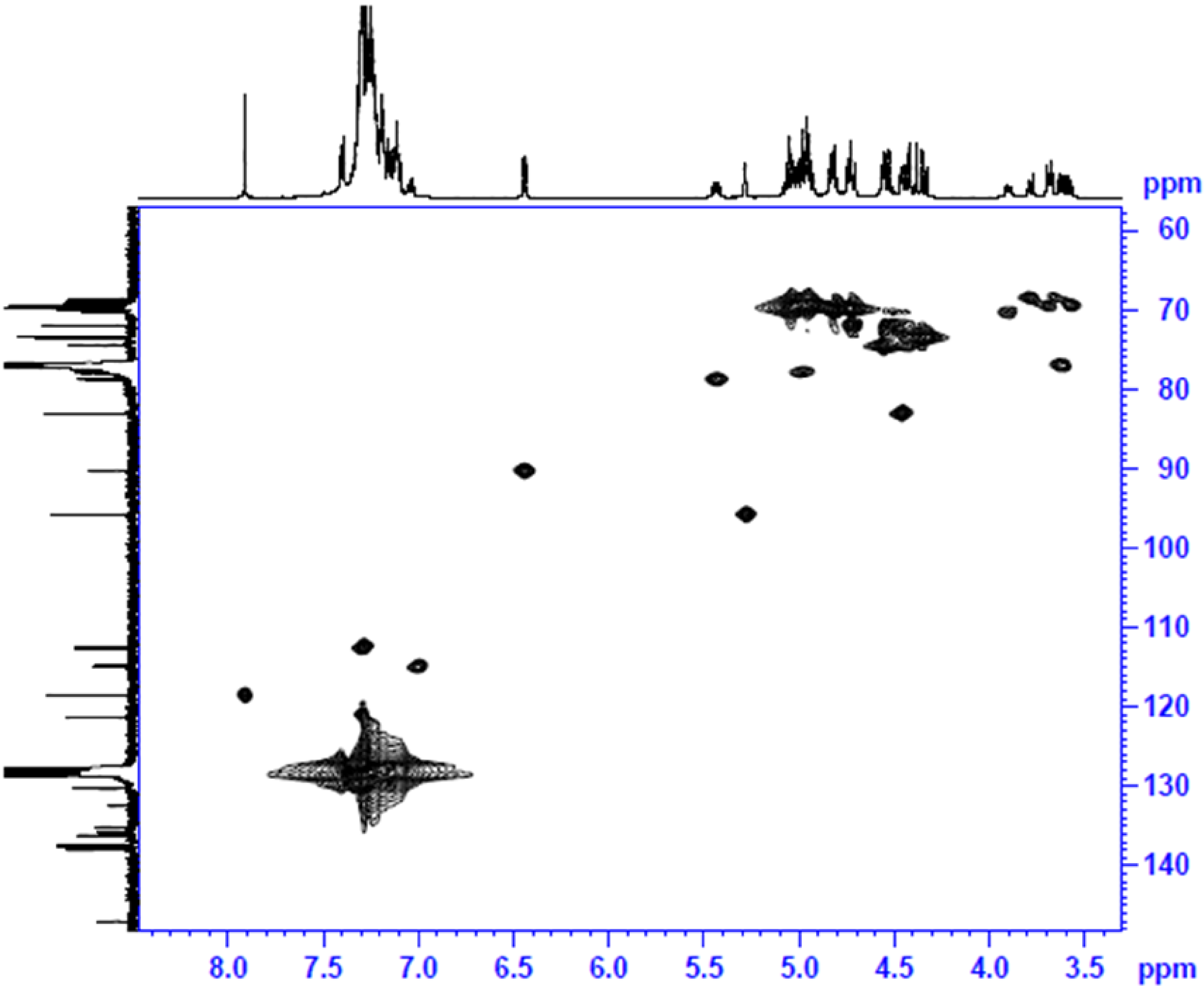

^{1}H NMR of 10f in MeOD

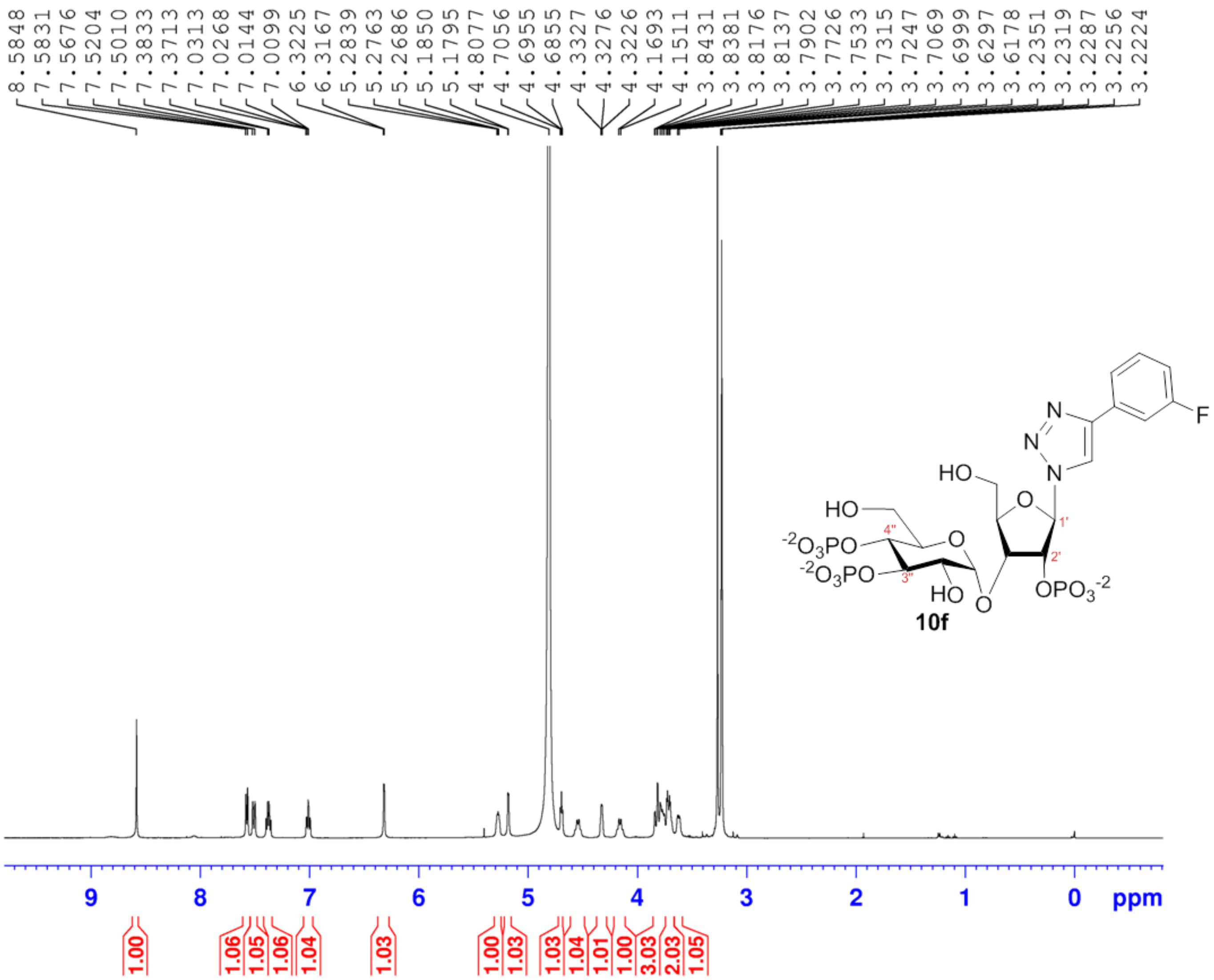

^{1}H NMR of 10f in MeOD (zoom)

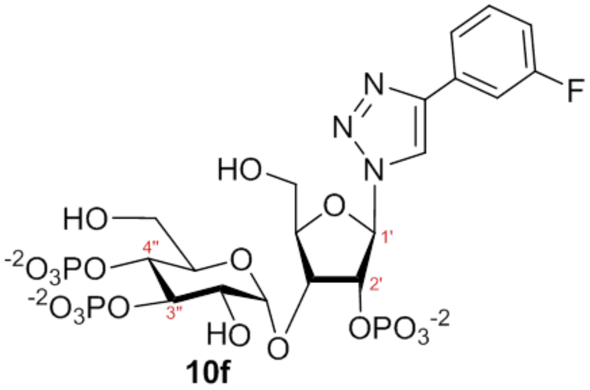

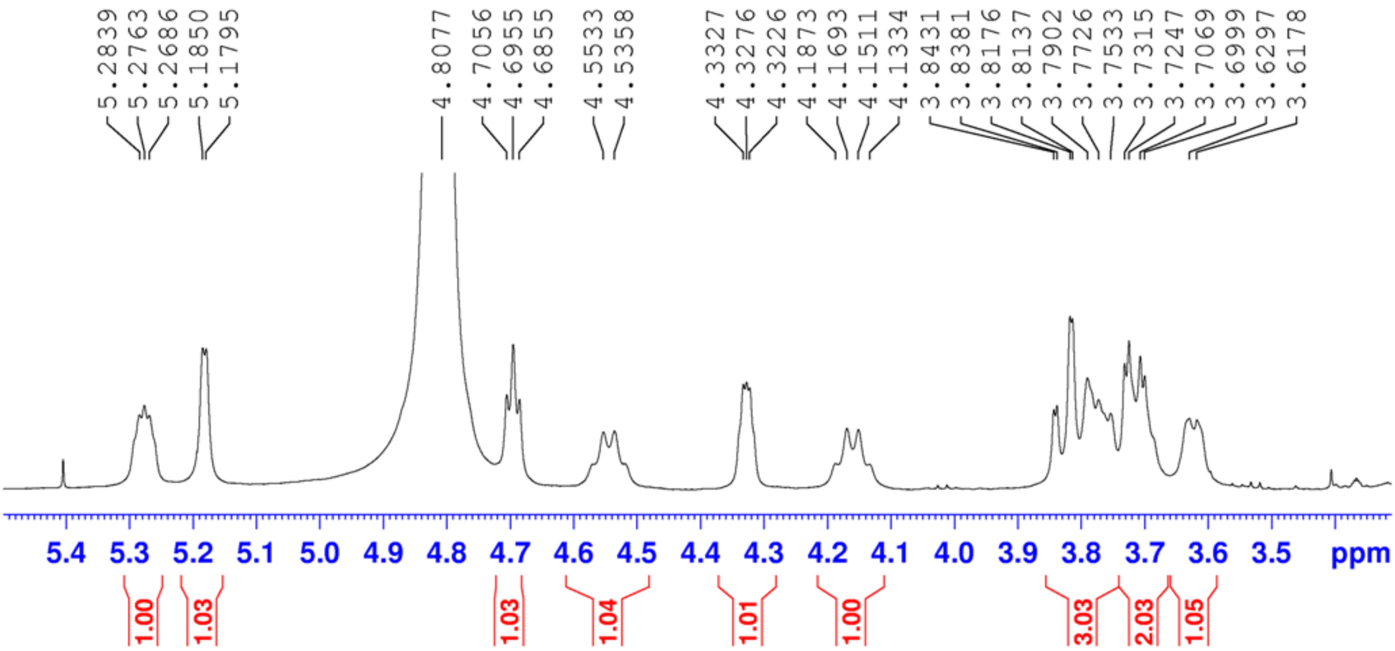

COSY of 10f in MeOD

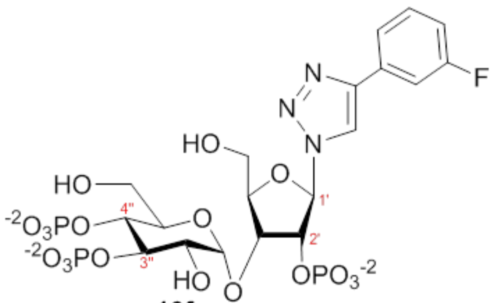

10f

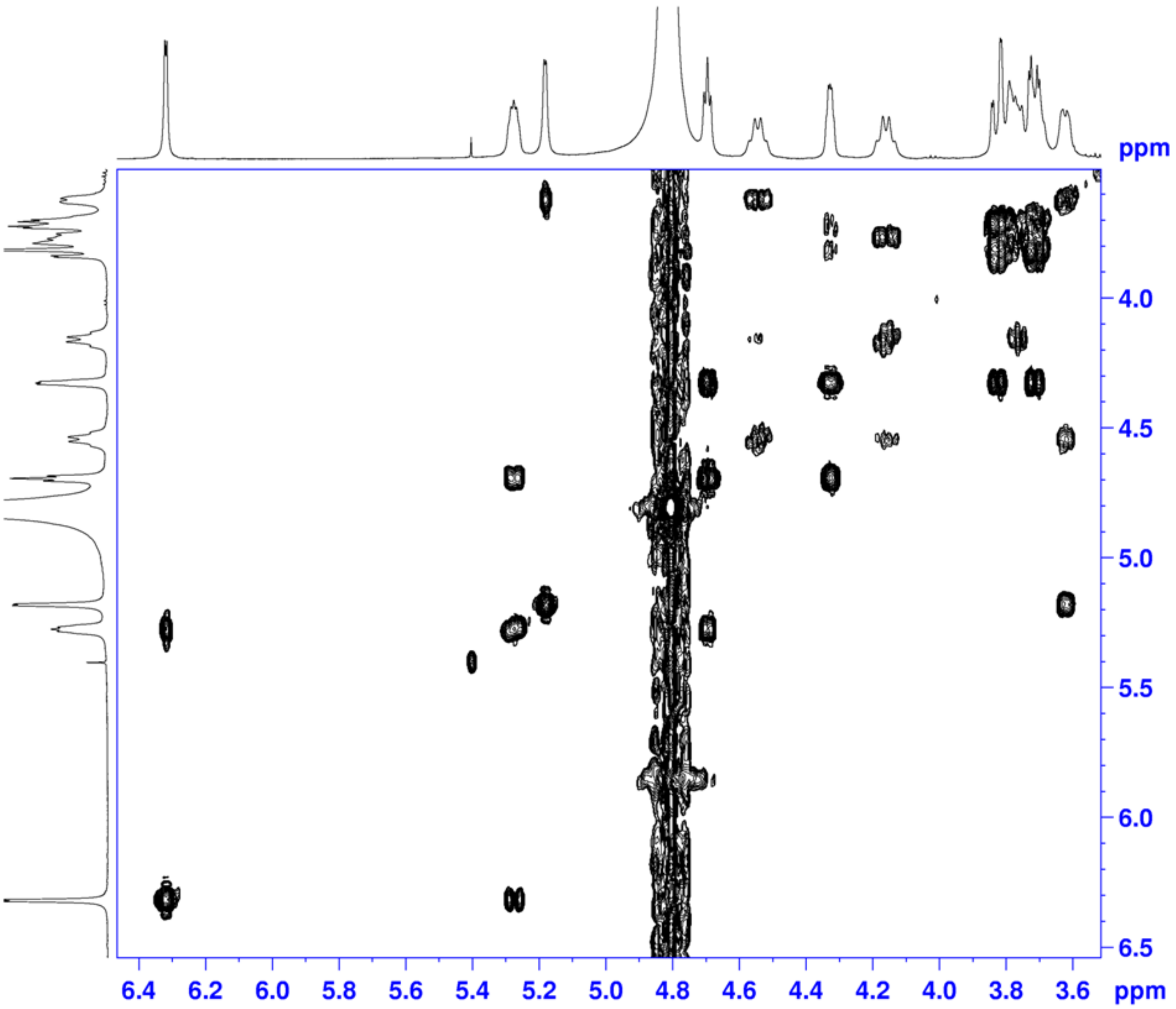

^{13}C NMR of 10f in MeOD

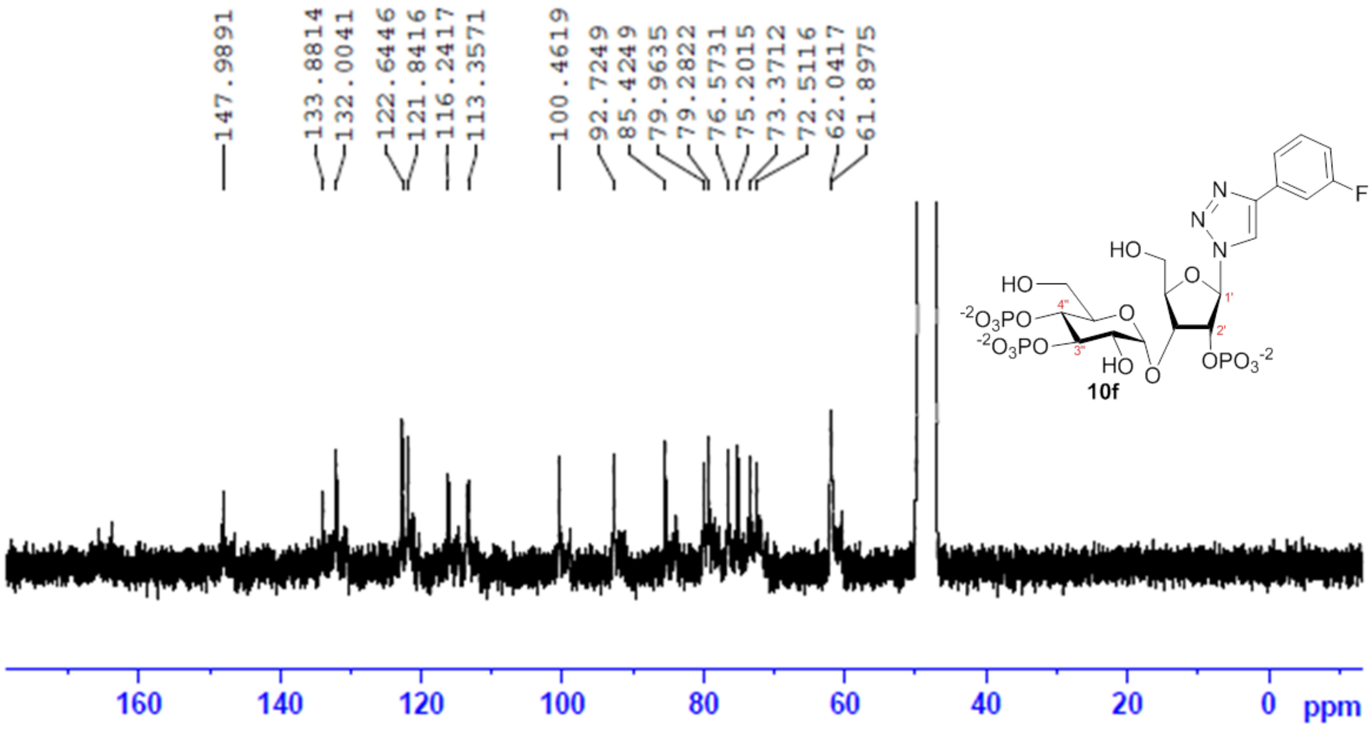

DEPT of 10f in MeOD

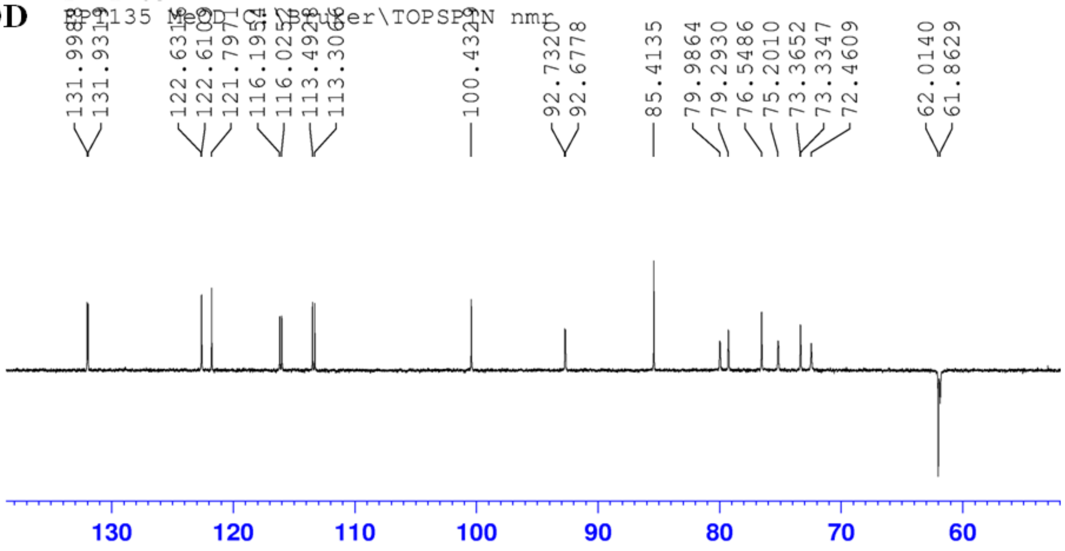

^{19}F NMR of 10f in MeOD

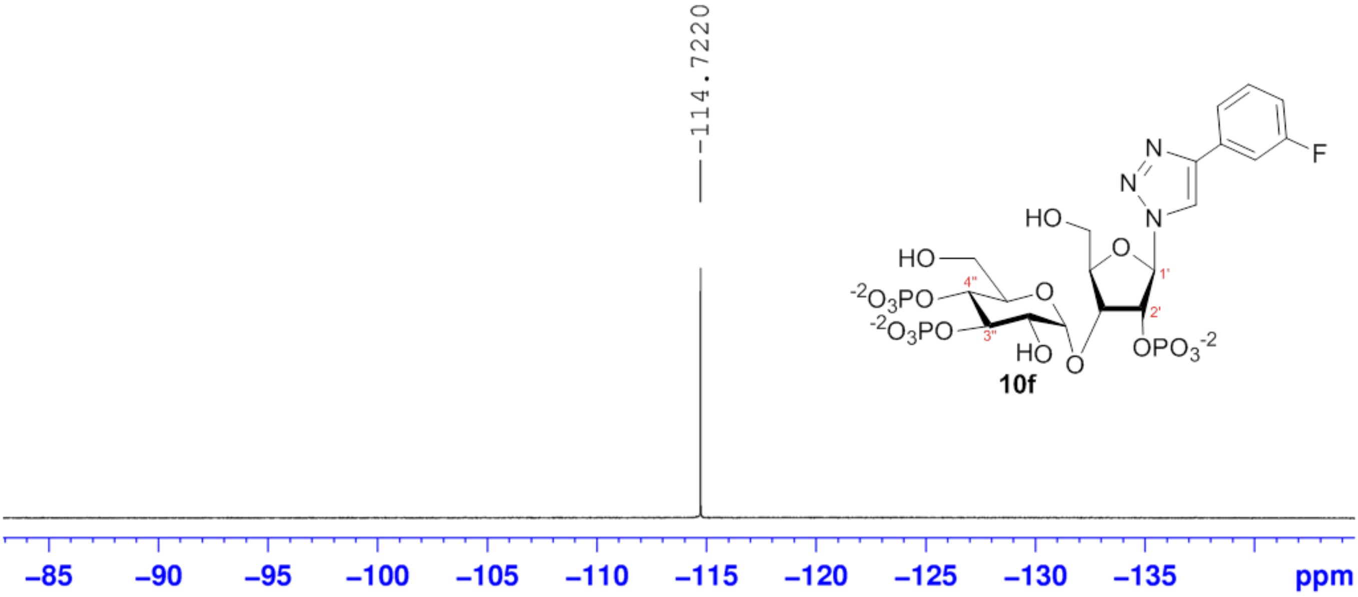

^{31}P NMR of 10f in MeOD

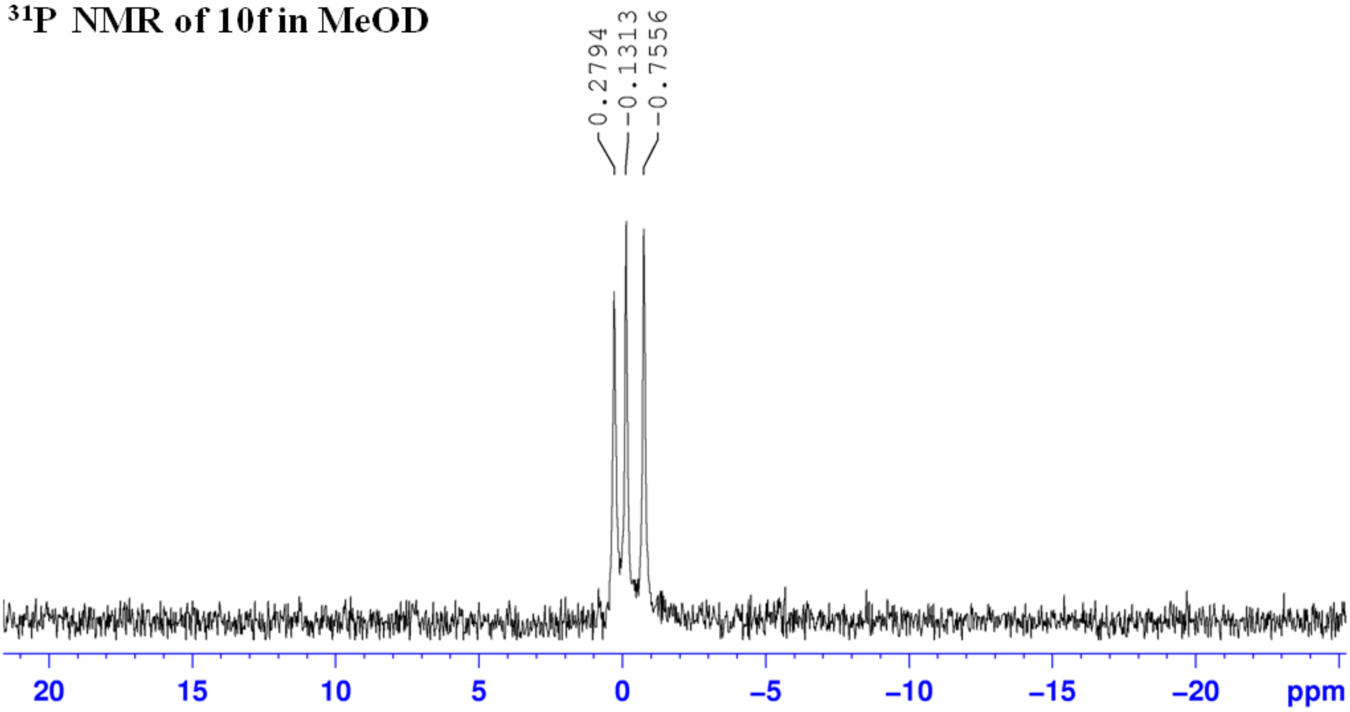

HMBC of 10f in MeOD

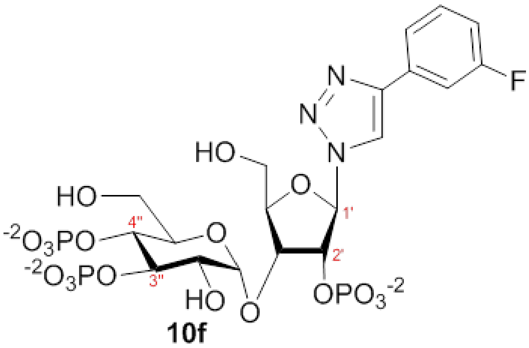

10f

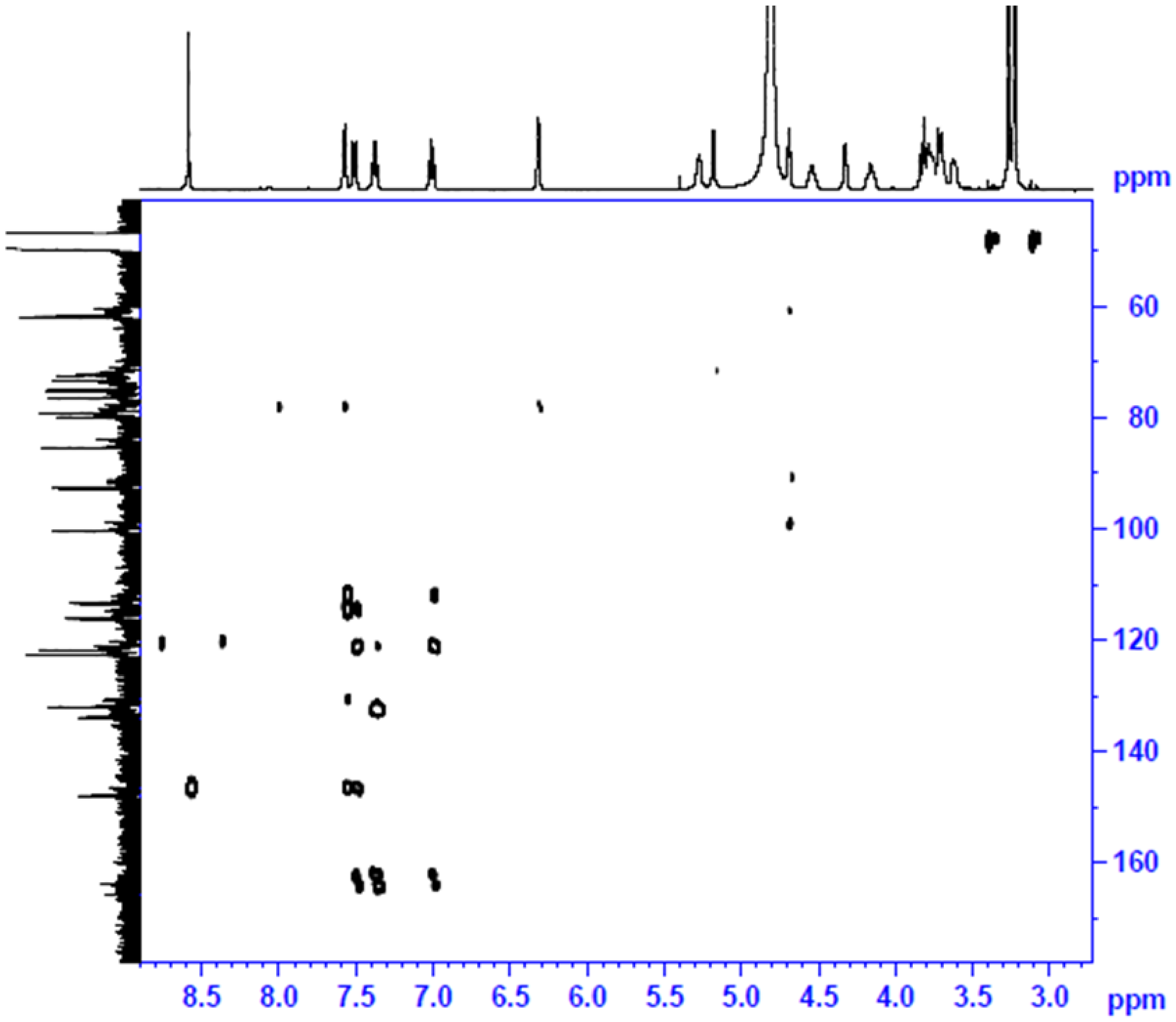

HMQC of 10f in MeOD

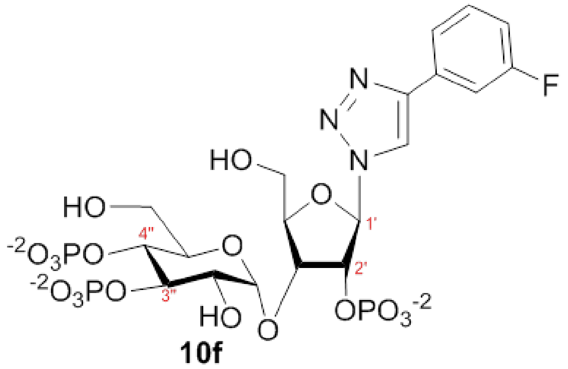

10f

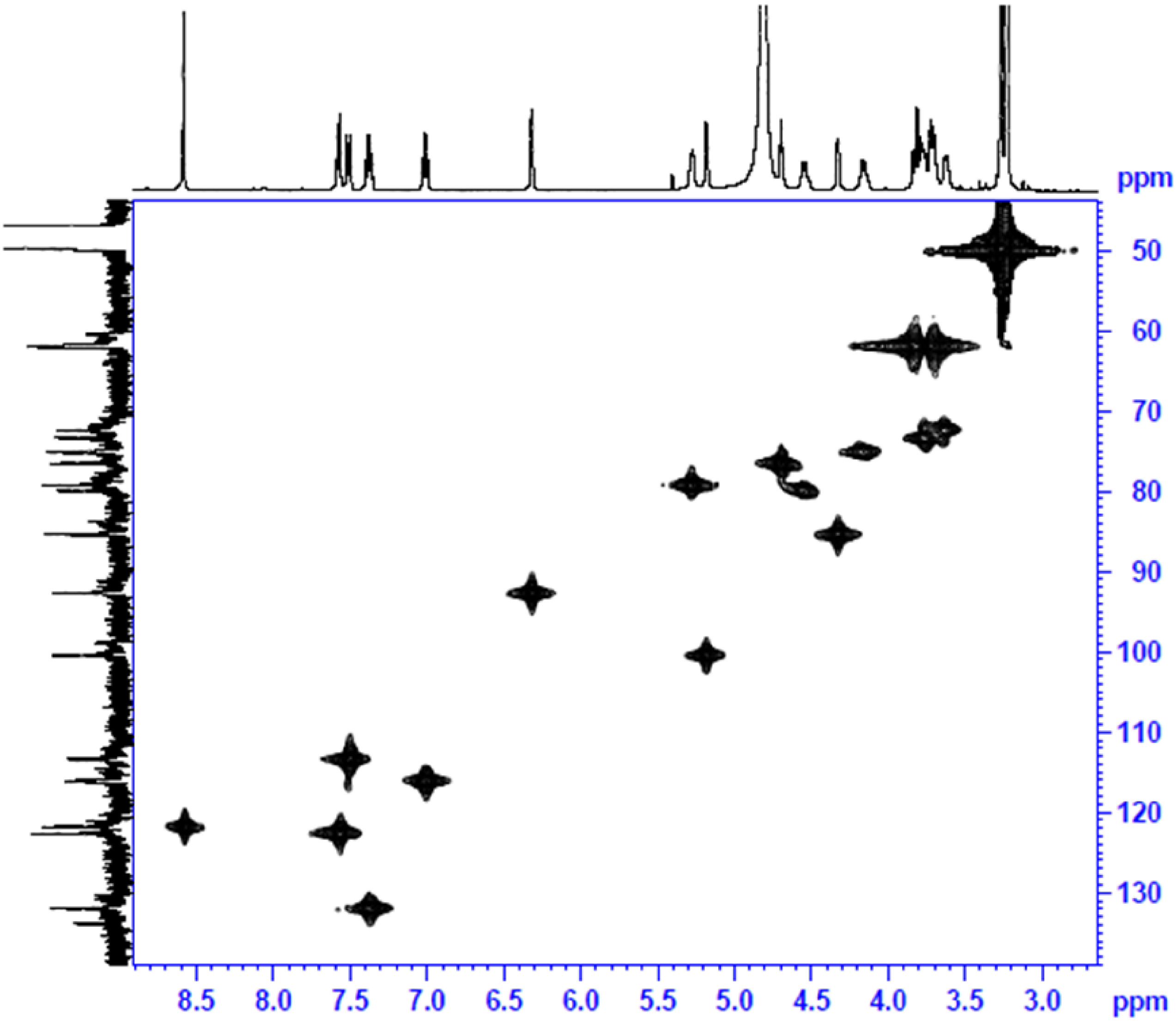

^{1}H NMR of 9g in $CDCl_3$

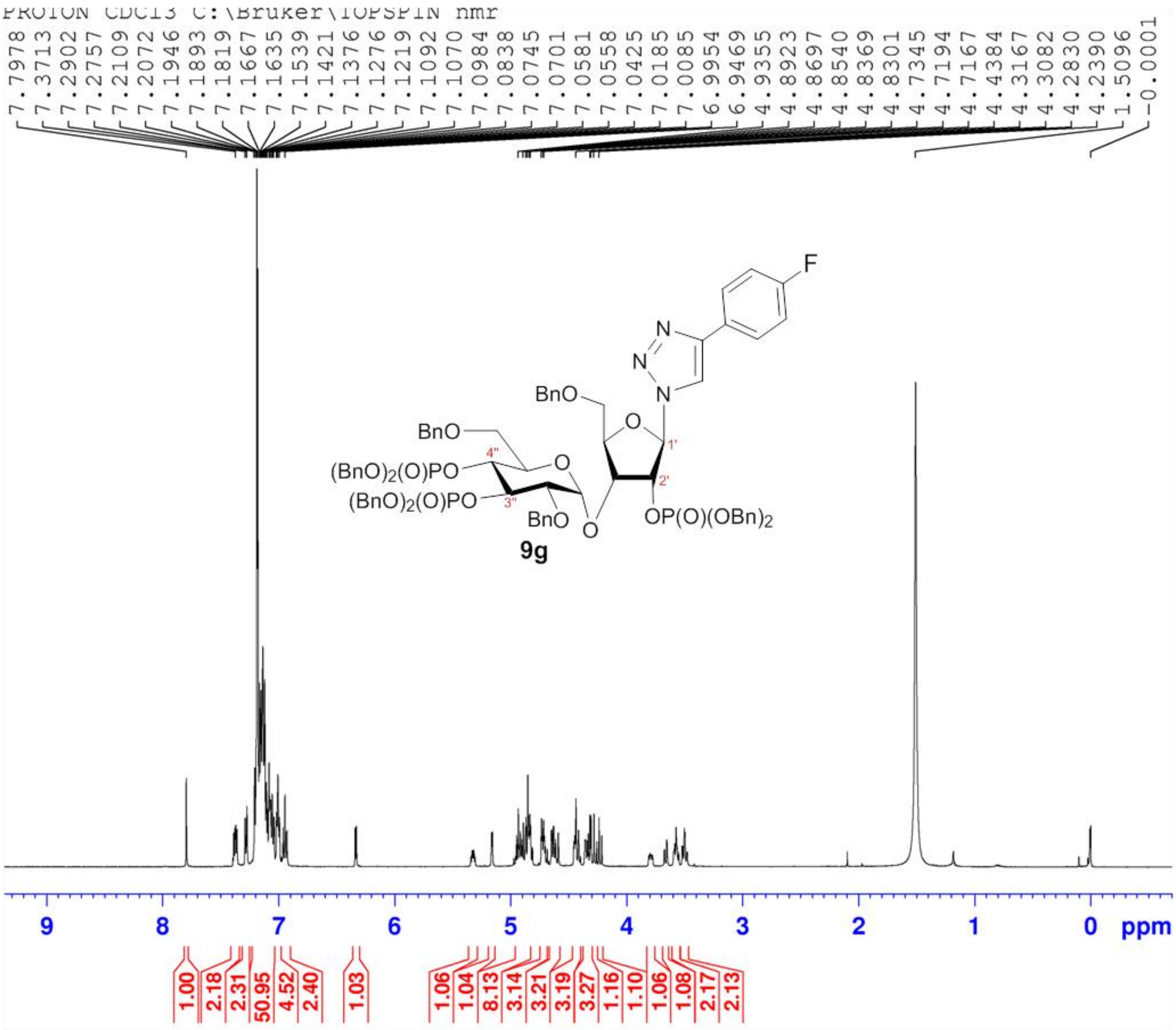

^{1}H NMR of 9g in $CDCl_3$ (zoom)

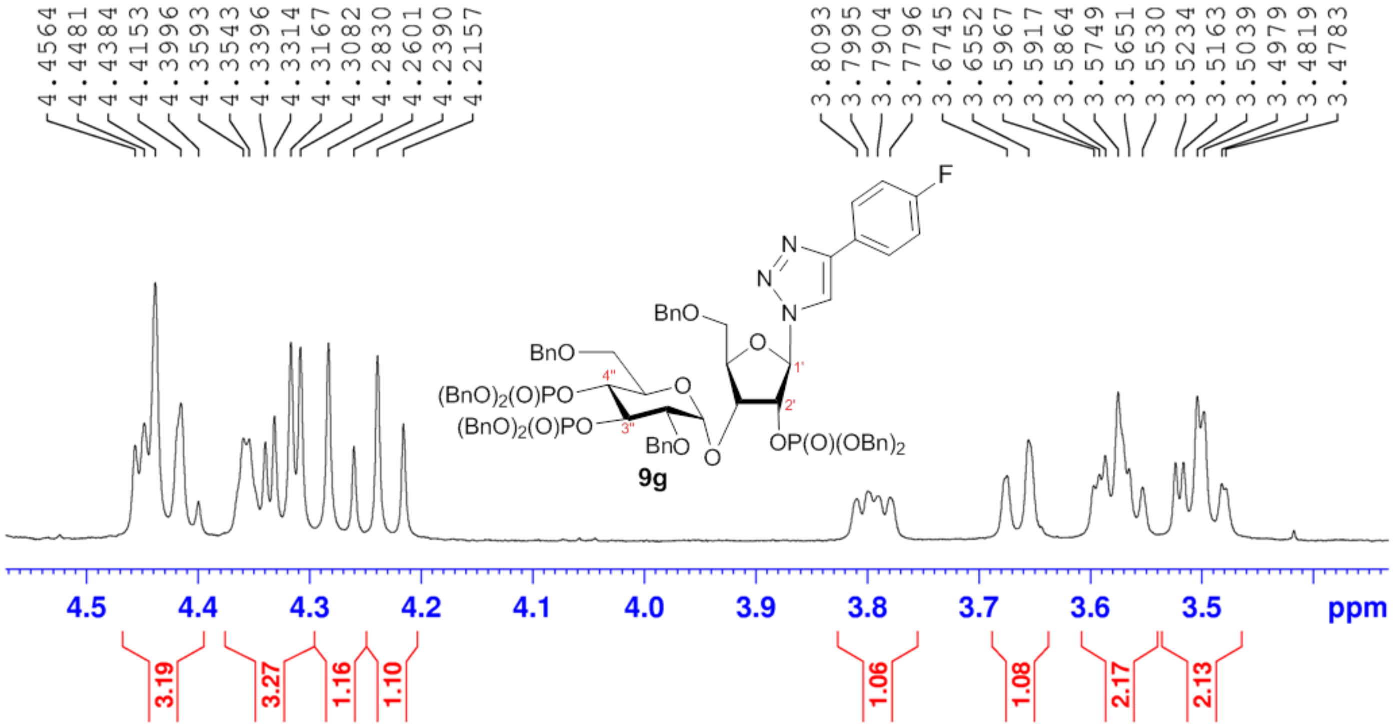

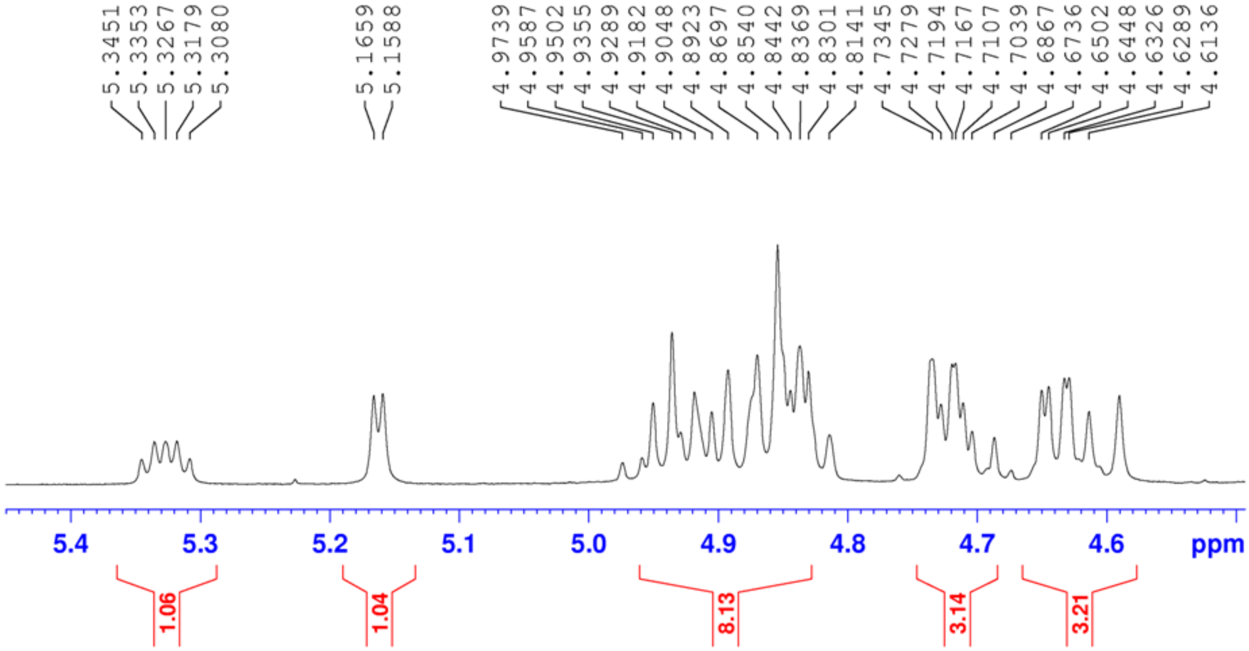

COSY of 9g in $CDCl_3$

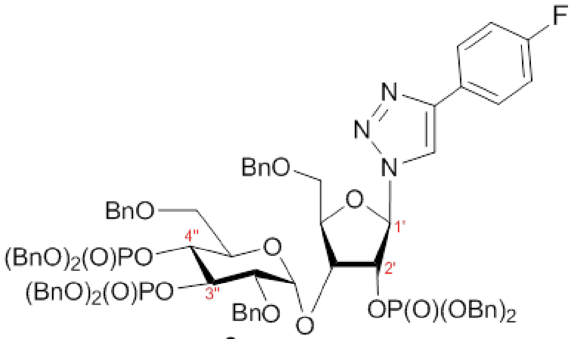

9g

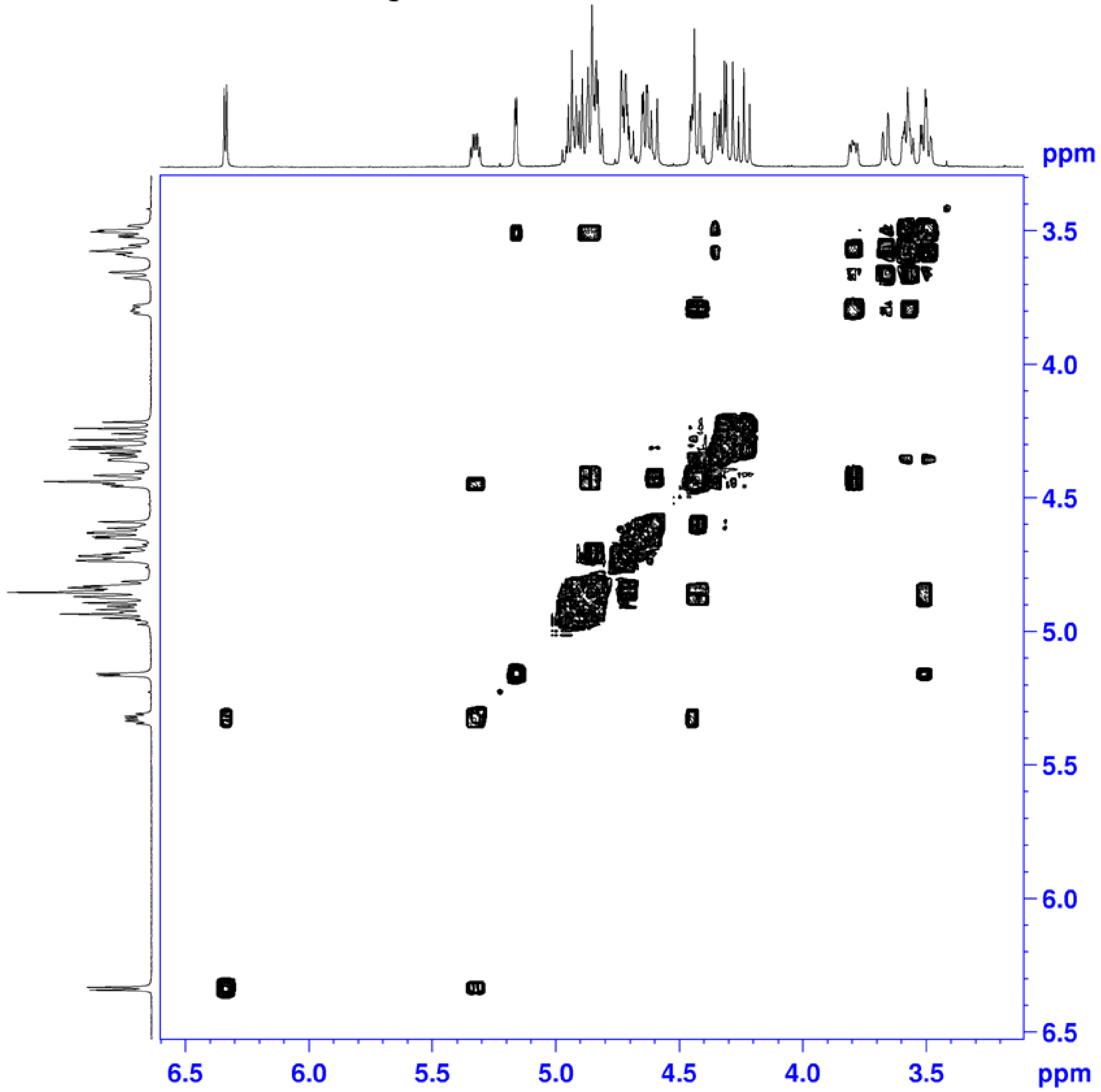

^{13}C NMR of 9g in CDCl$_3$

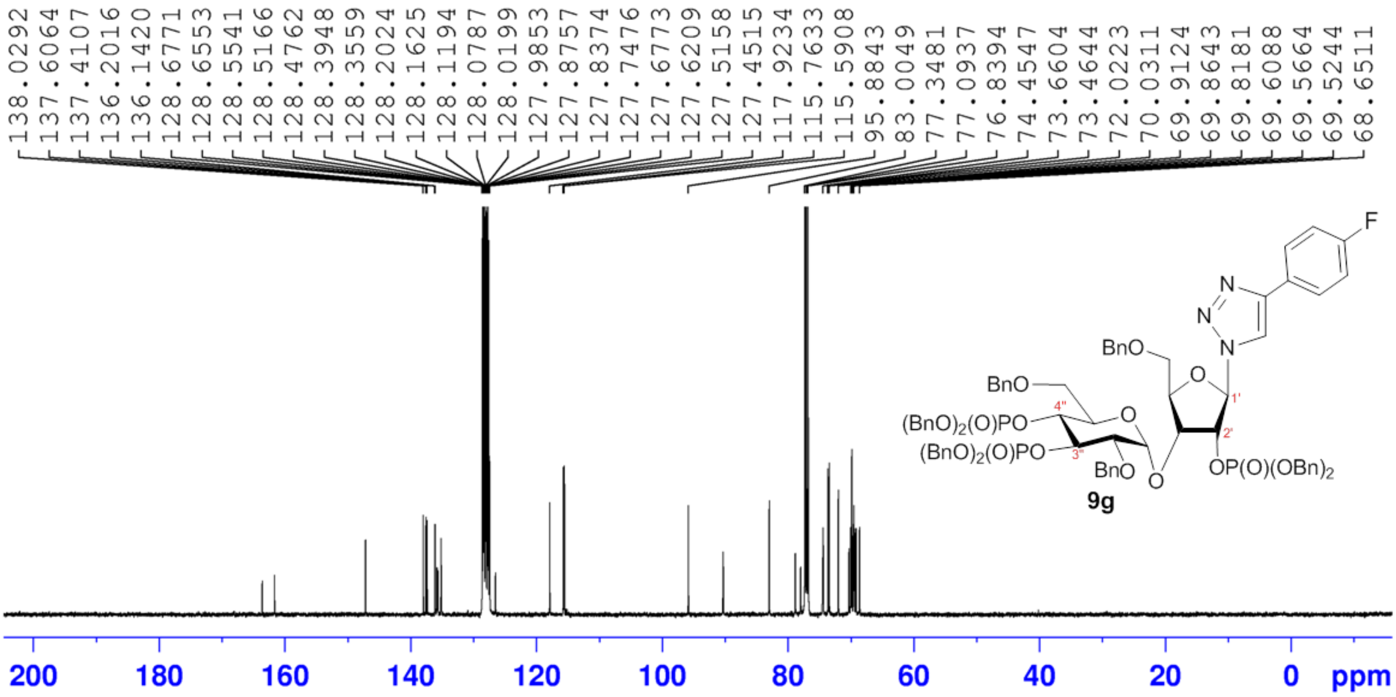

zoom

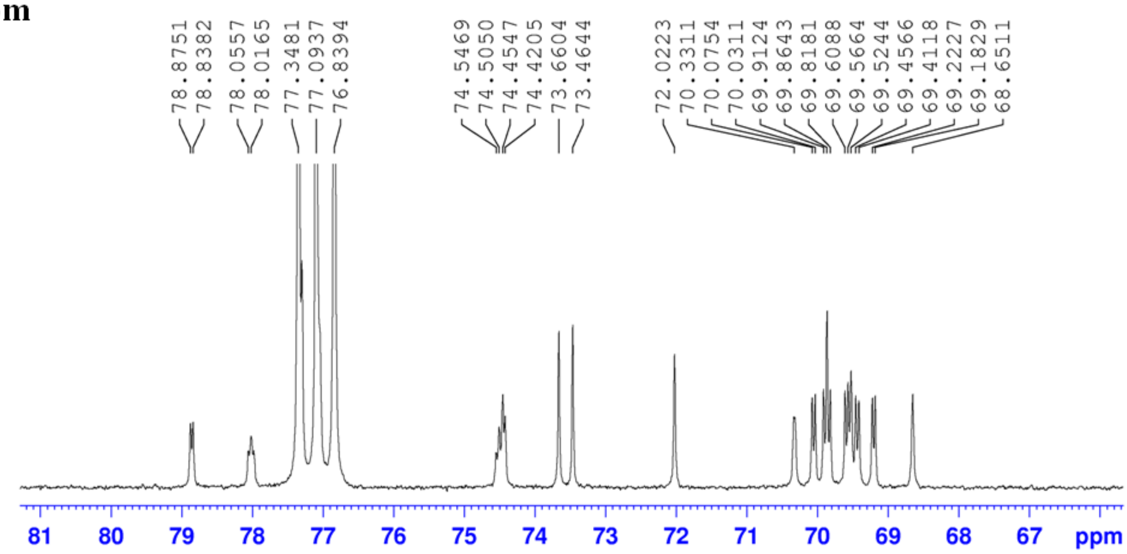

DEPT of 9g in $CDCl_3$

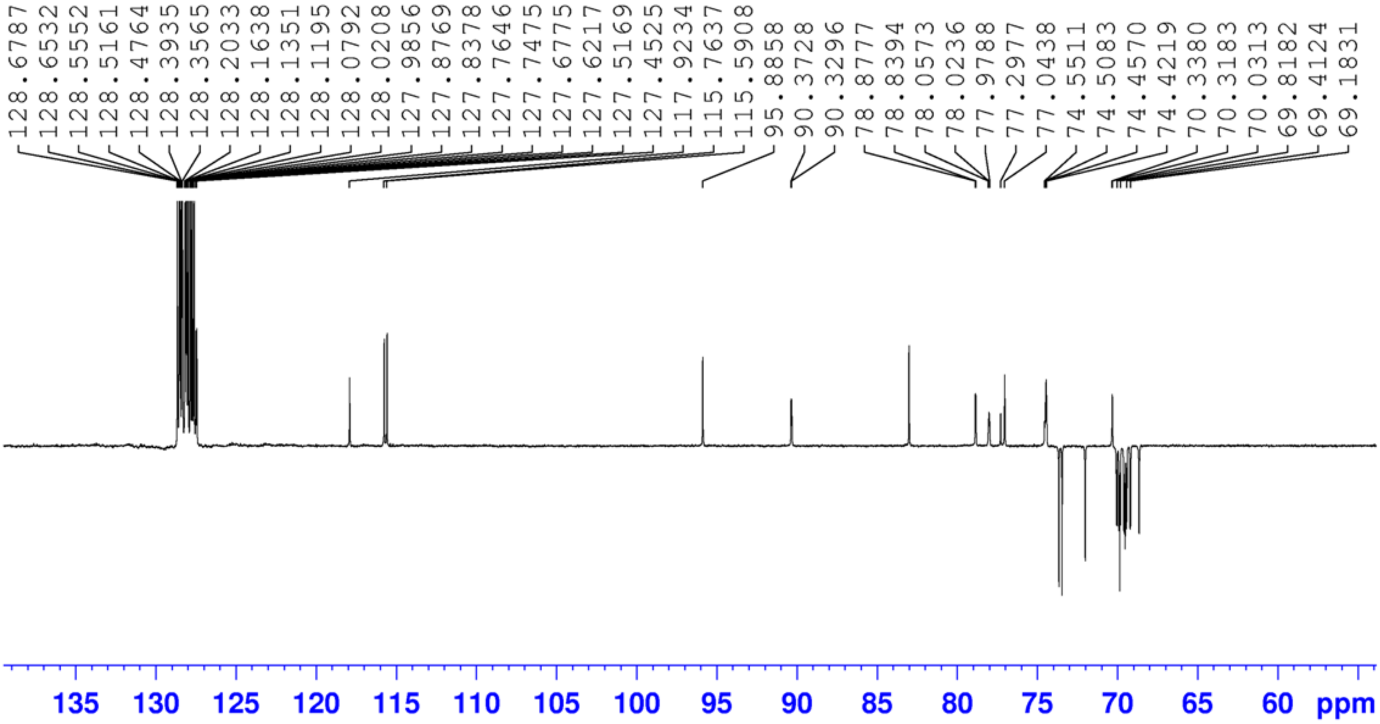

^{31}P NMR of 9g in $CDCl_3$

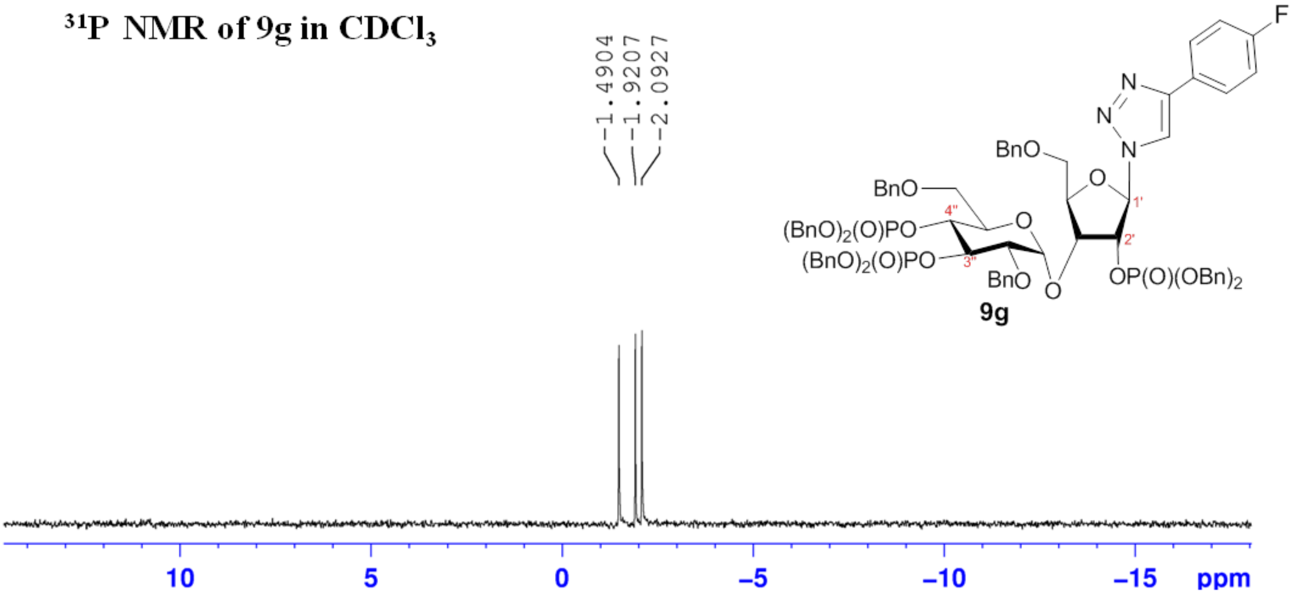

9g

^{19}F NMR of 9g in $CDCl_3$

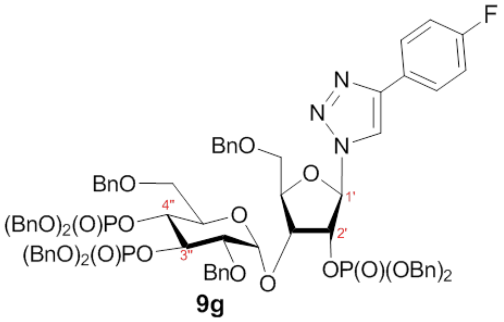

9g

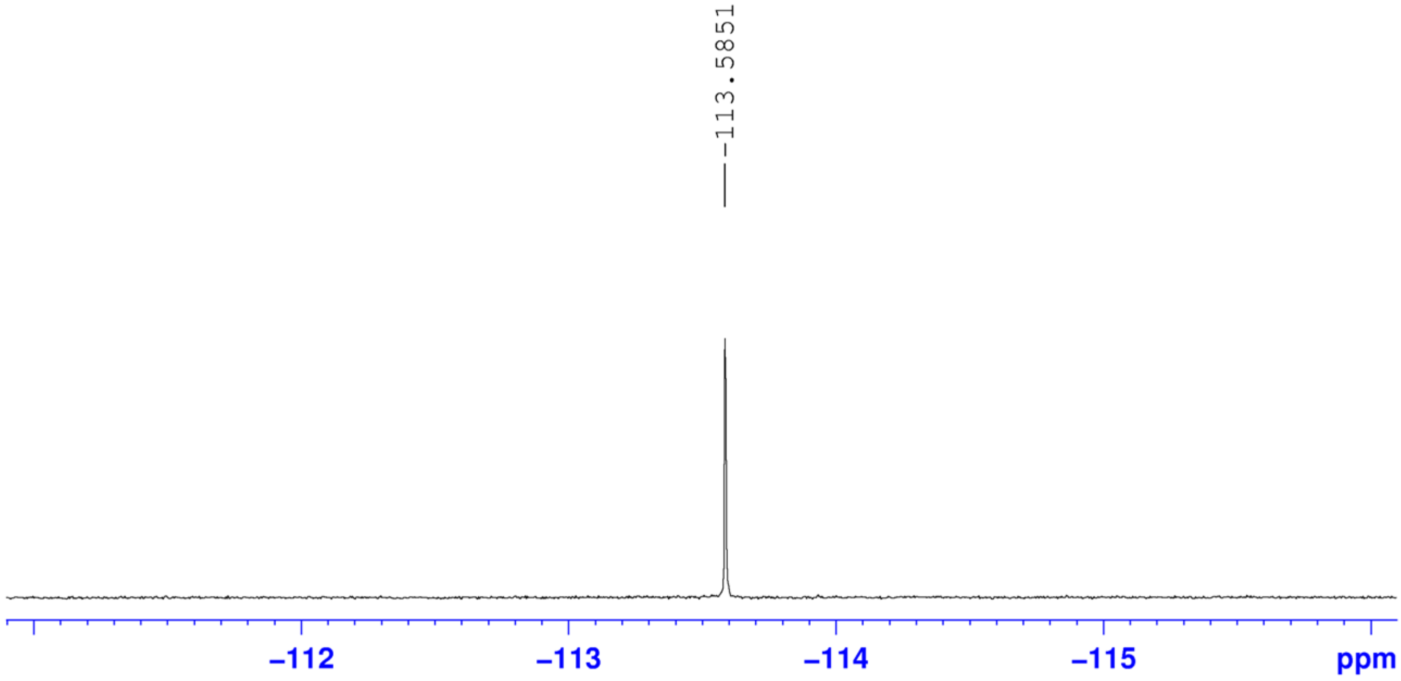

HMBC of 9g in $CDCl_3$

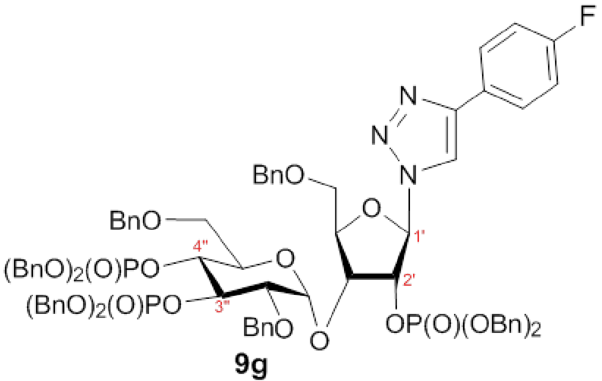

9g

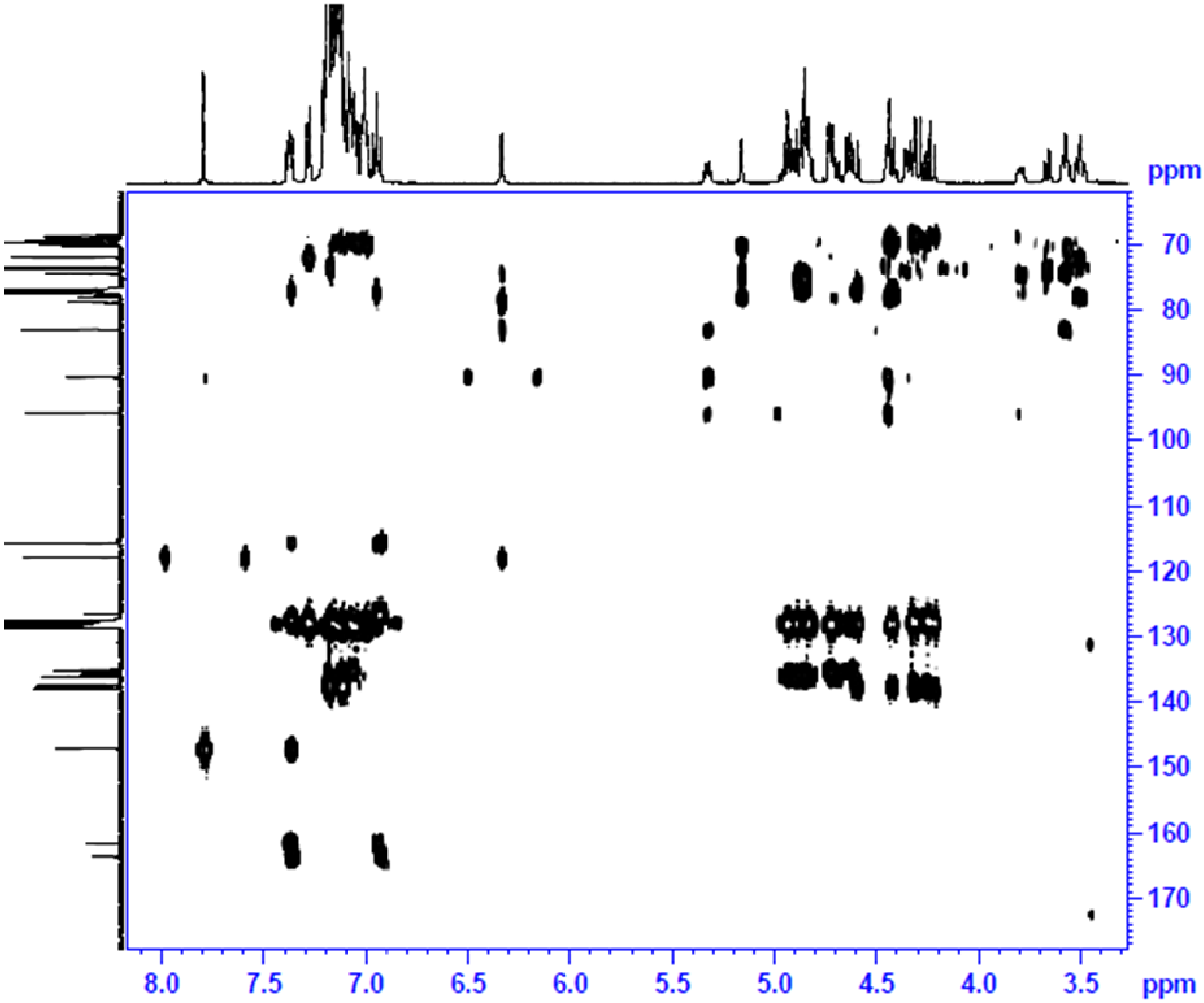

HMQC of 9g in $CDCl_3$

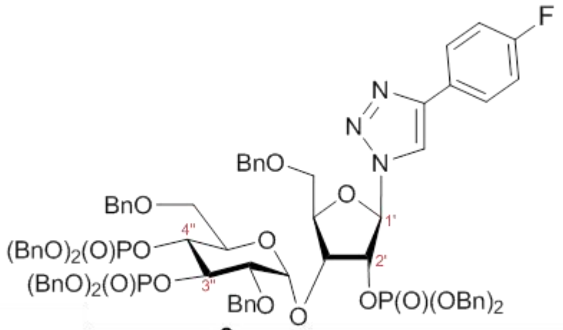

9g

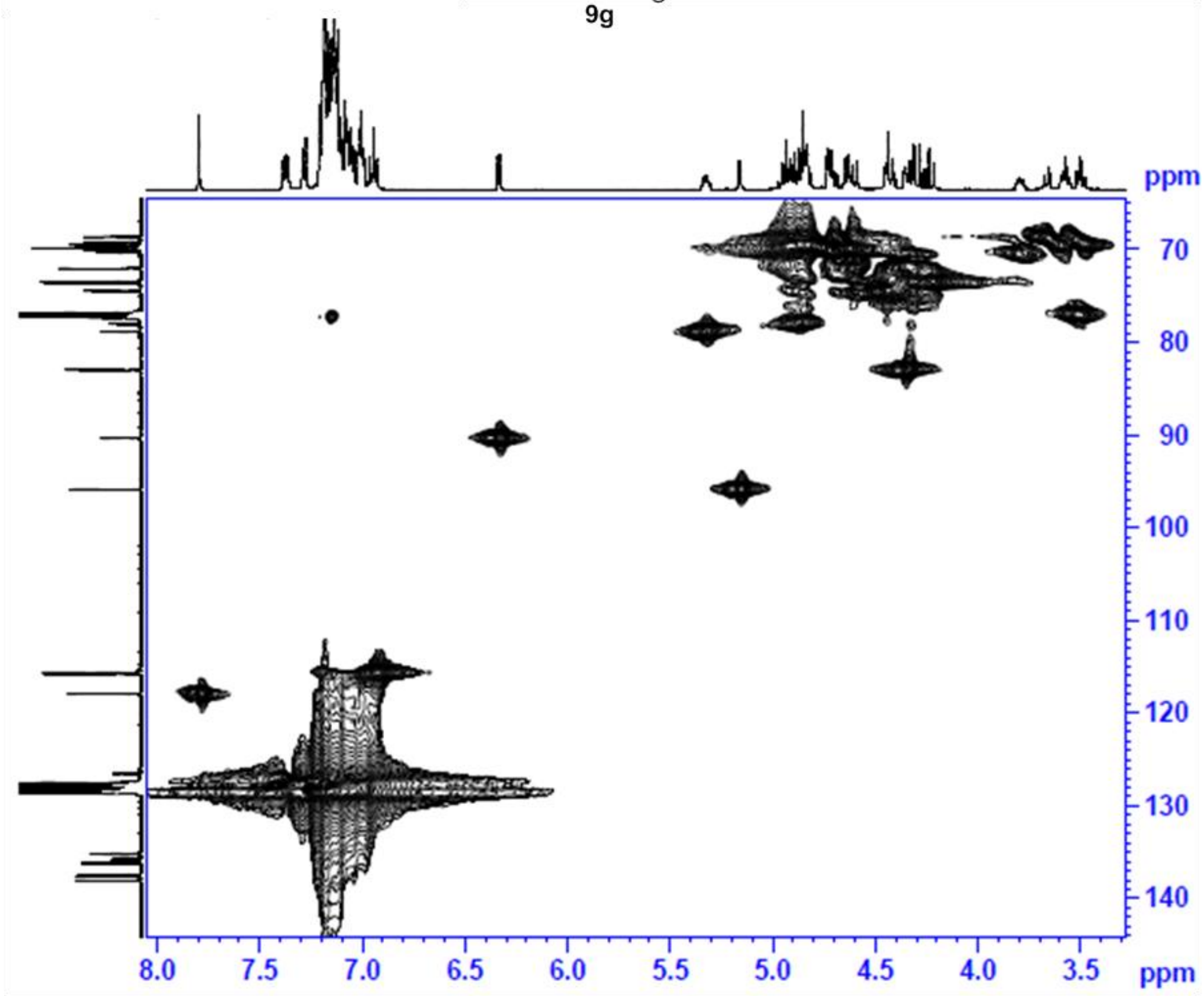

^{1}H NMR of 10g in MeOD

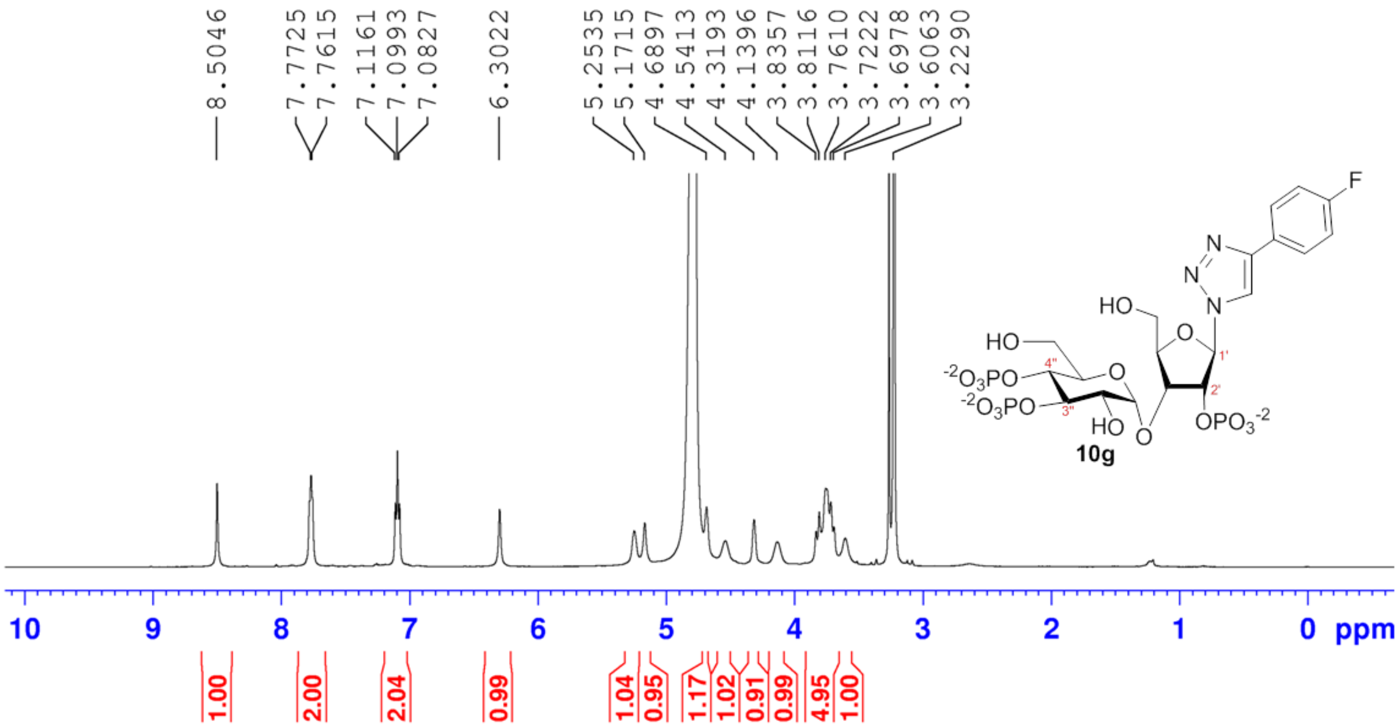

10g

zoom

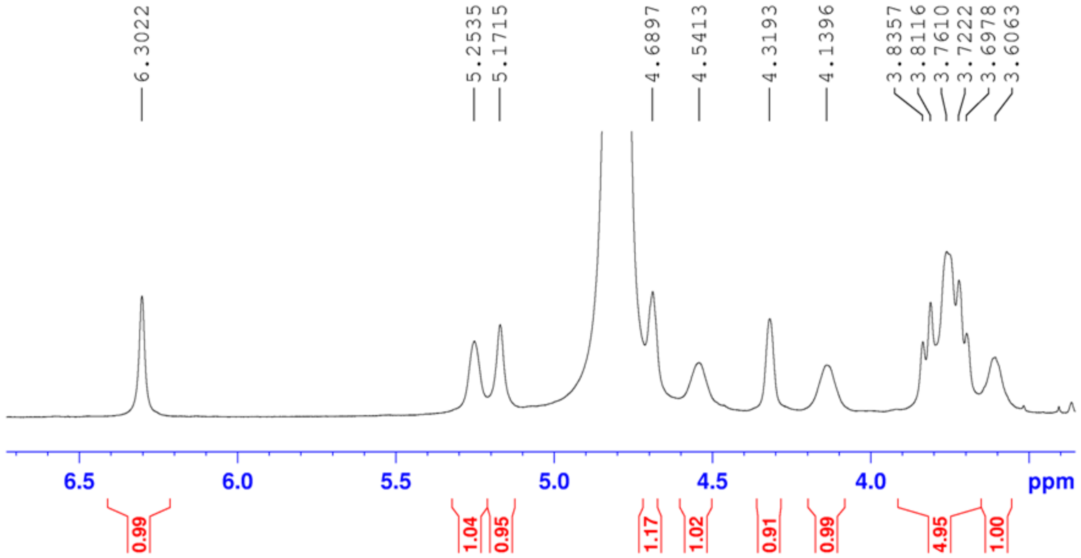

COSY of 10g in MeOD

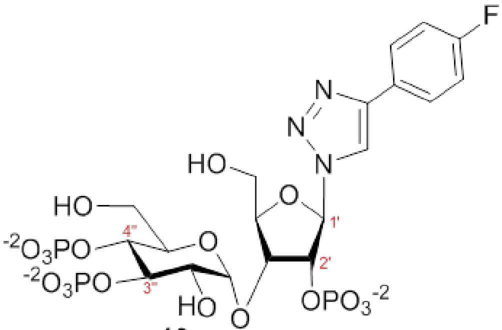

10g

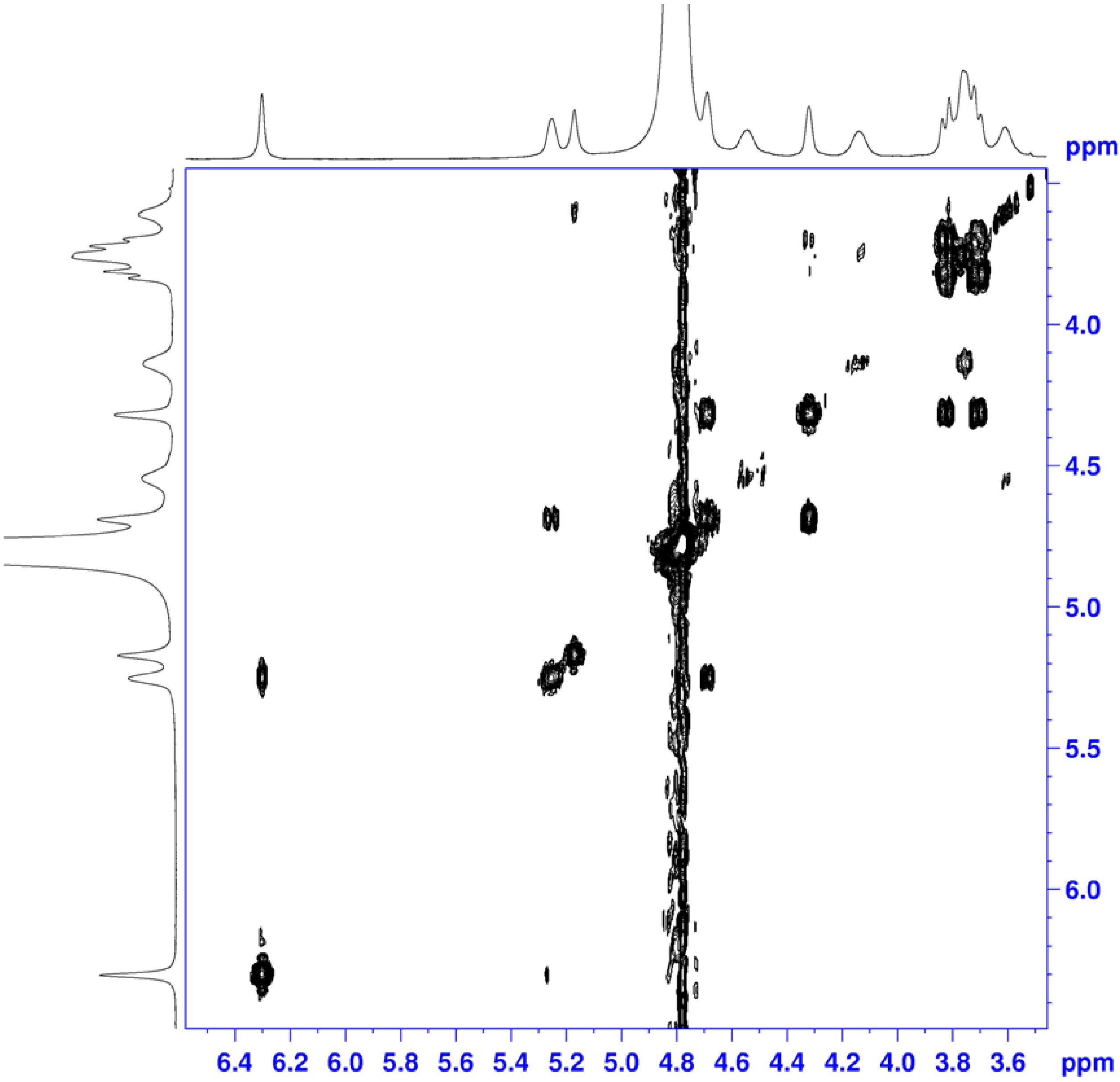

^{13}C NMR of 10g in MeOD

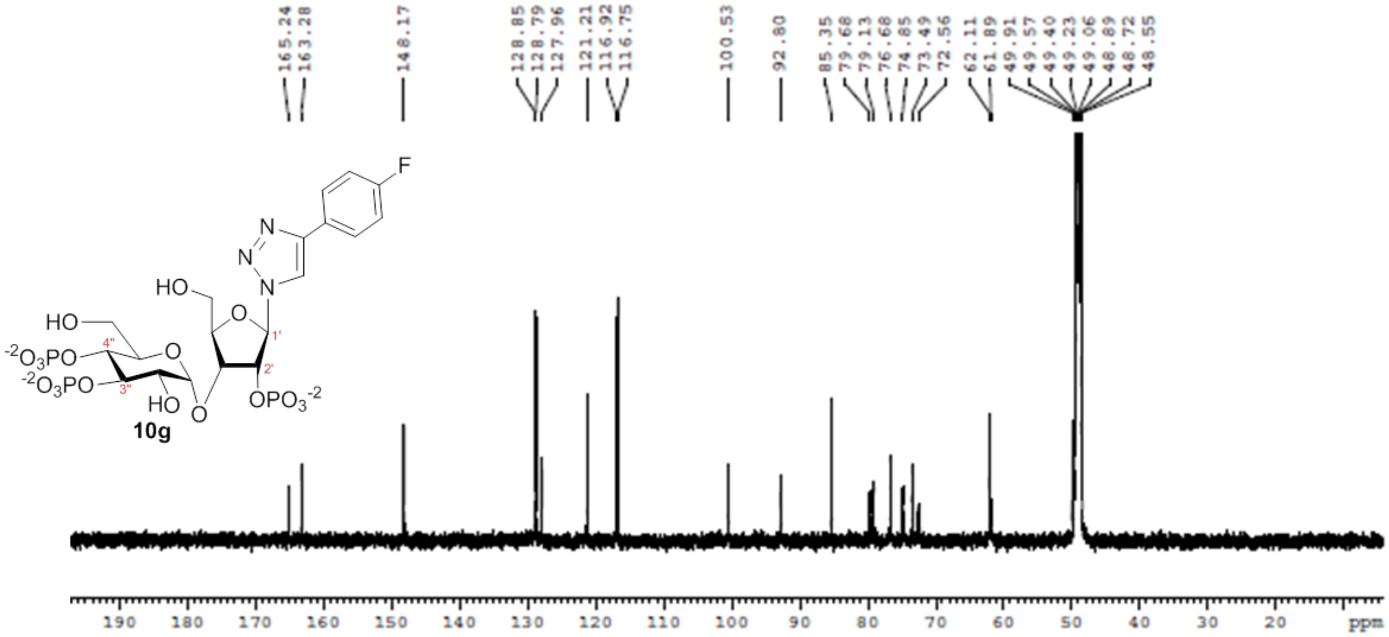

DEPT of 10g in MeOD

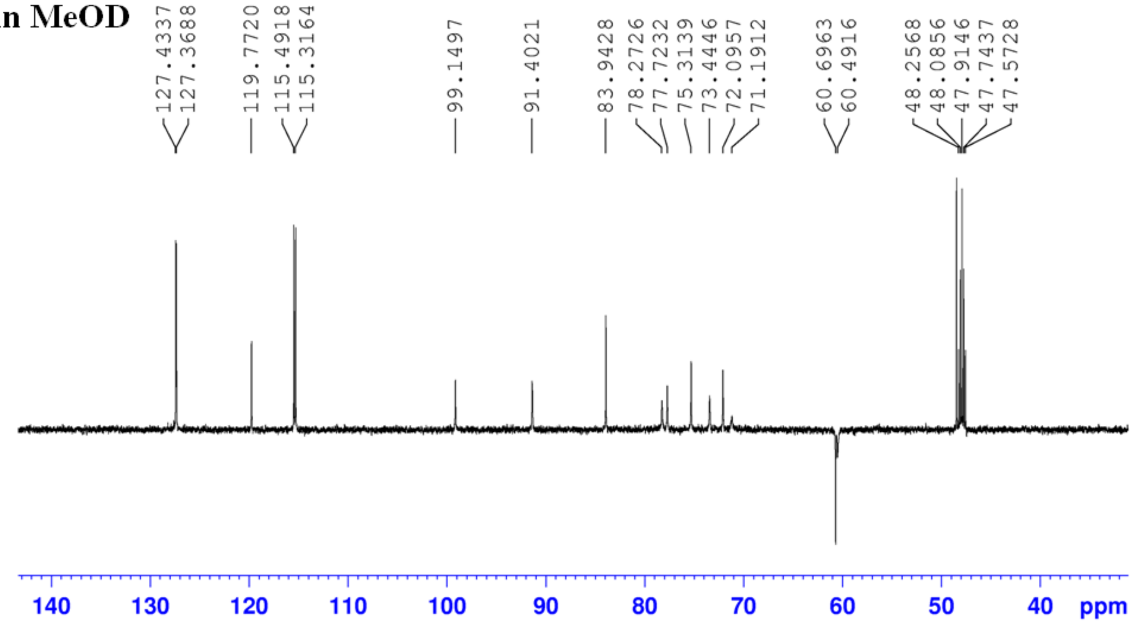

^{19}F NMR of 10g in MeOD

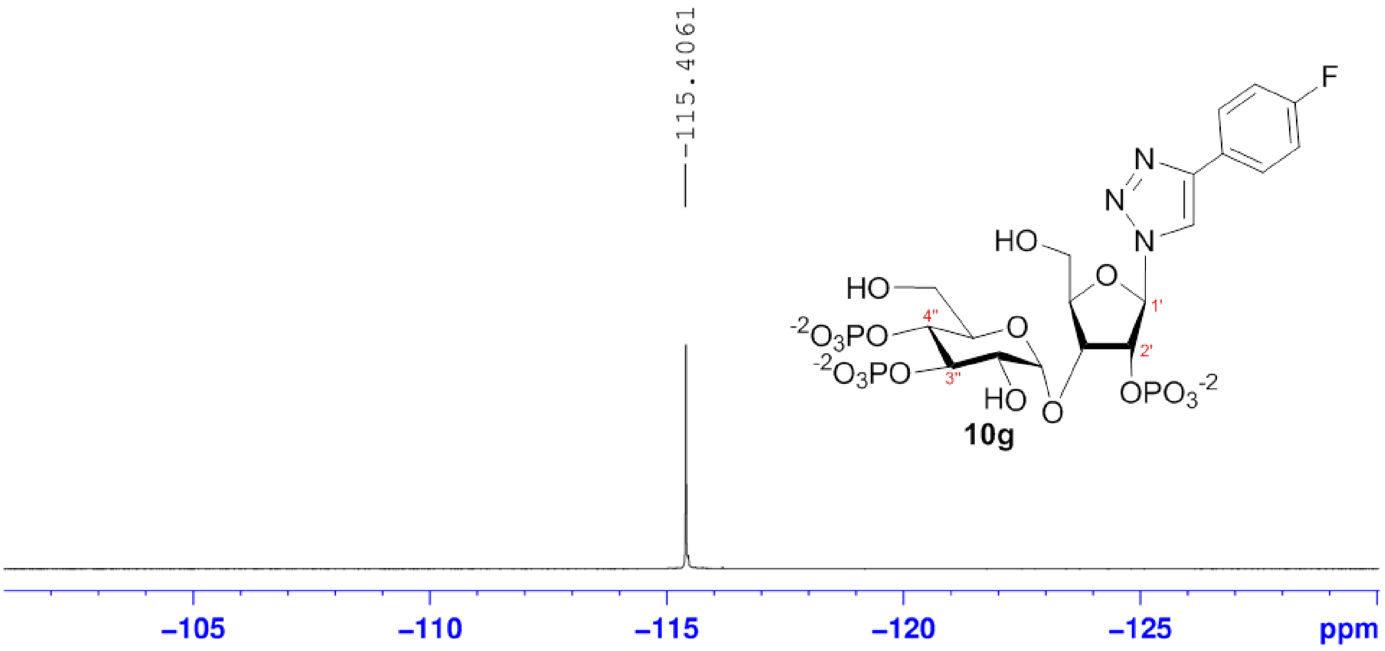

^{31}P NMR of 10g in MeOD

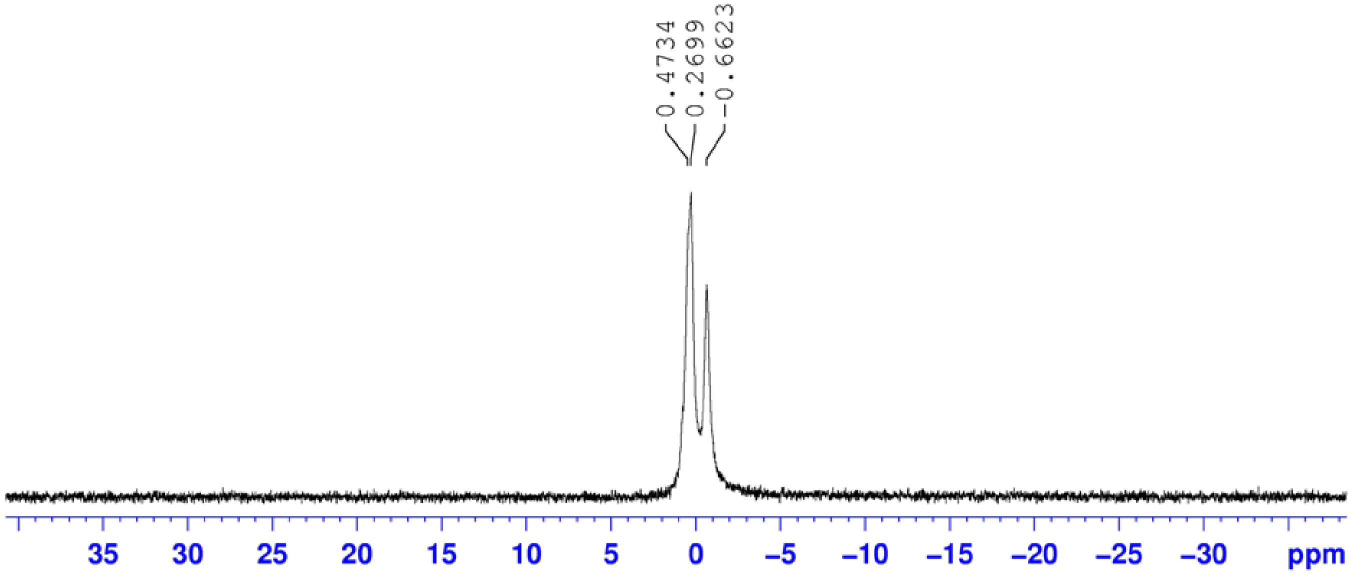

HMBC of 10g in MeOD

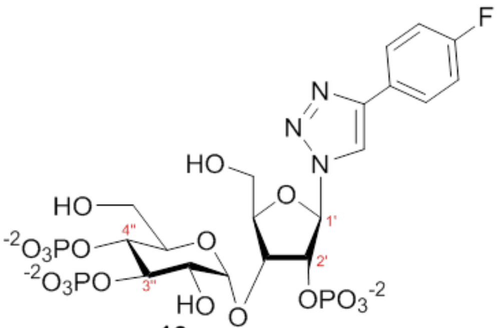

10g

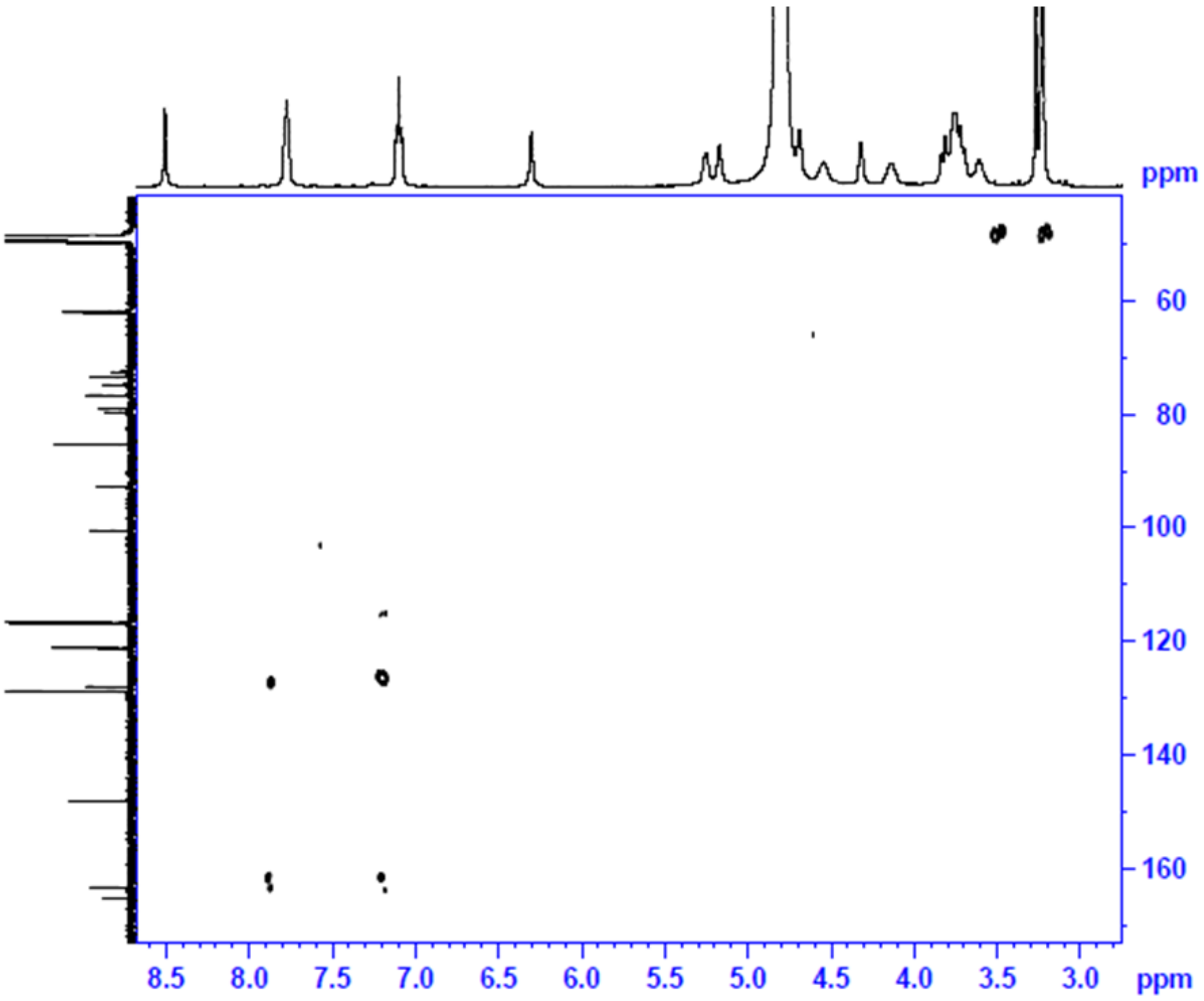

HMQC of 10g in MeOD

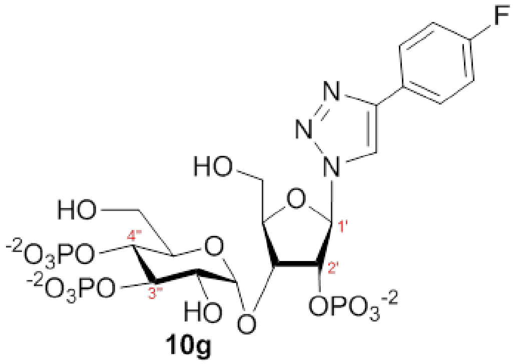

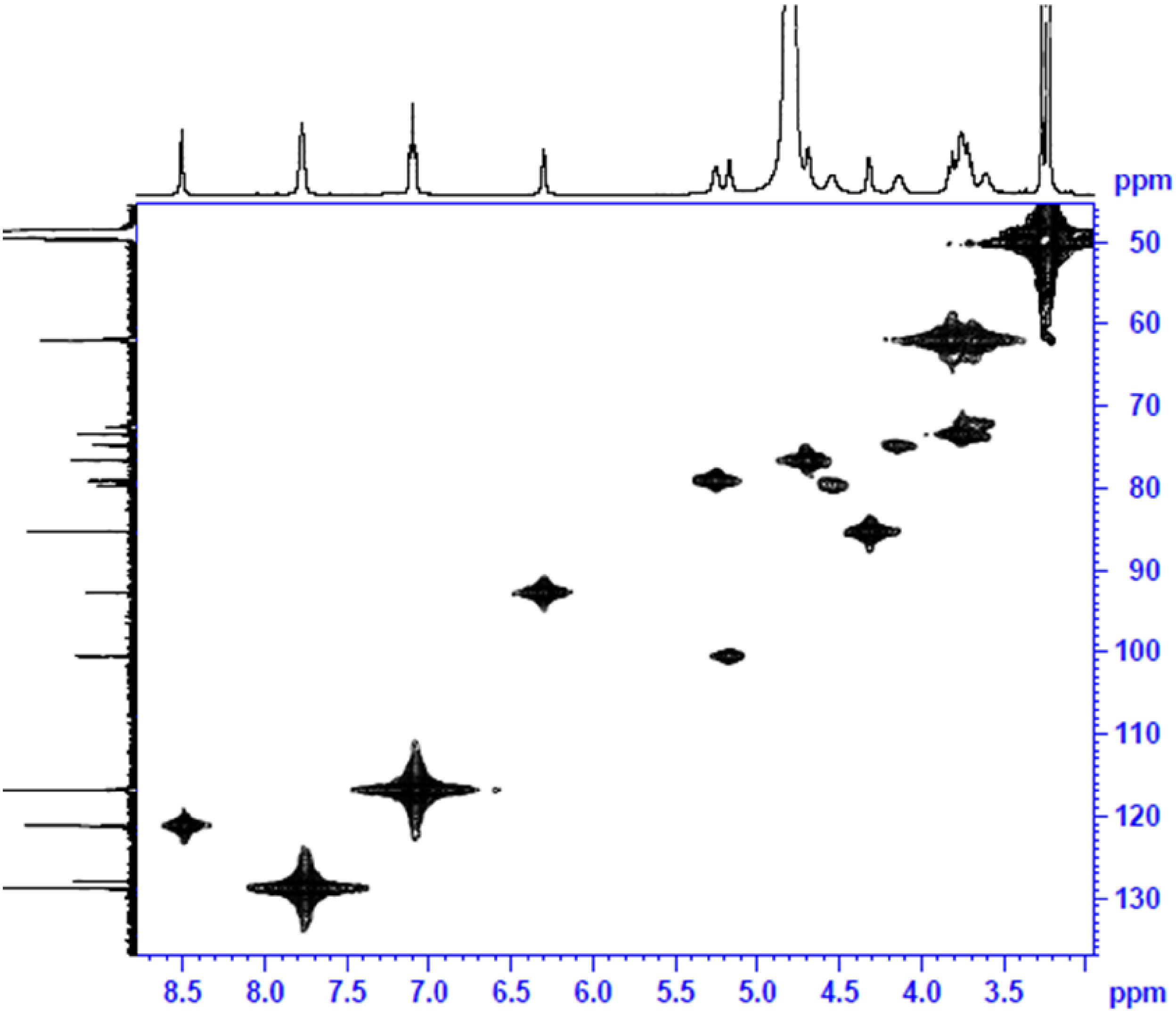

^{1}H NMR of 9h in $CDCl_3$

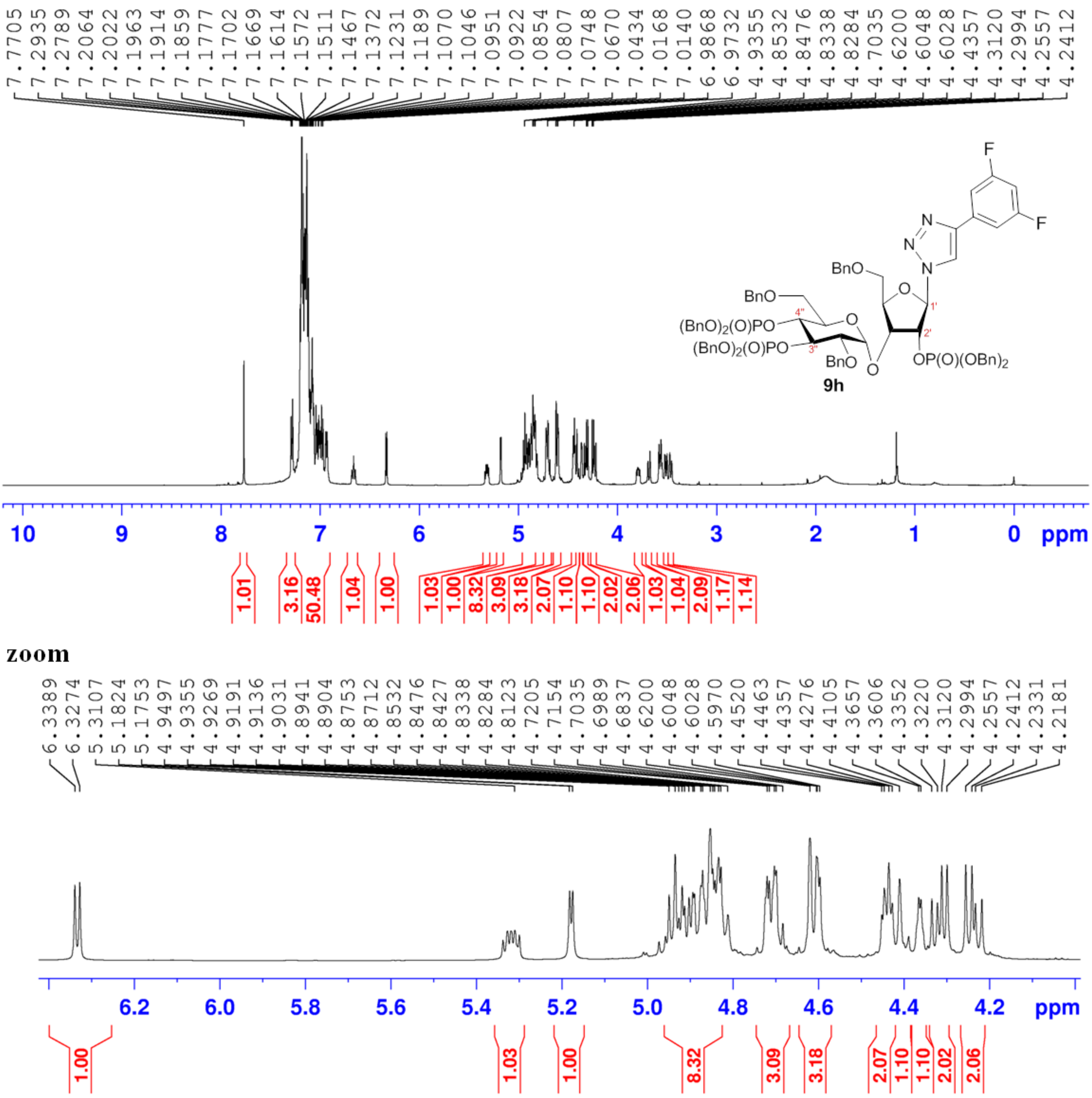

COSY of 9h in $CDCl_3$

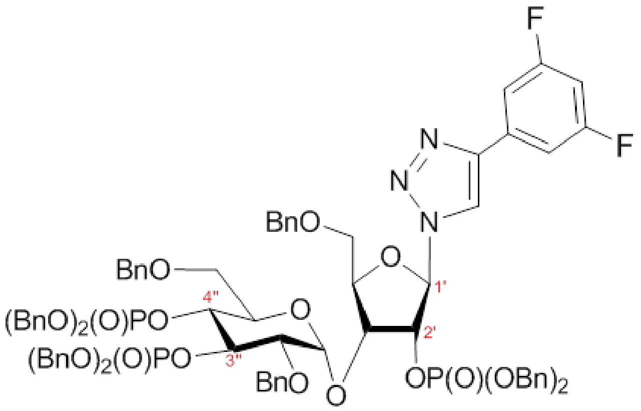

9h

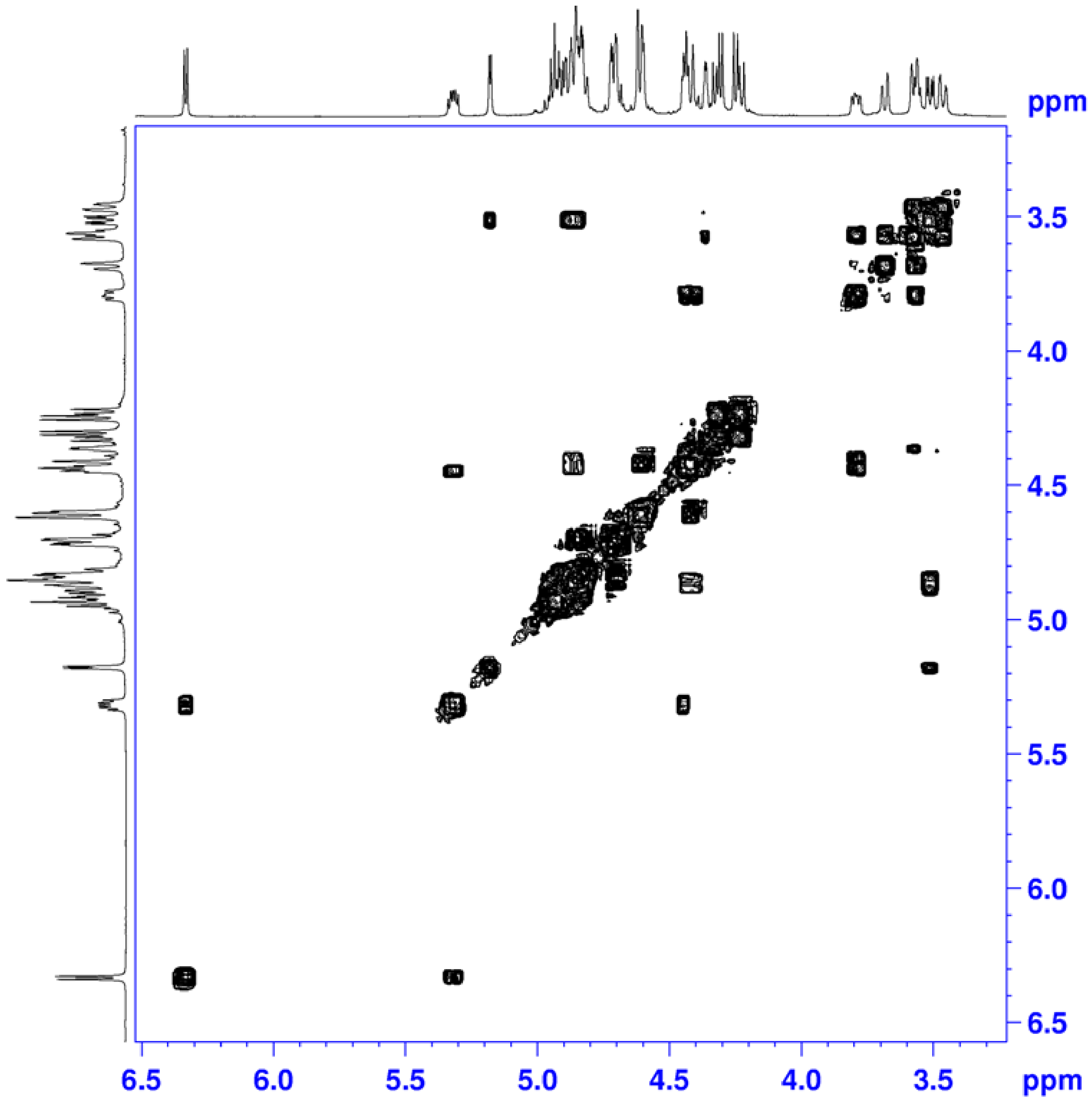

^{13}C NMR of 9h in $CDCl_3$

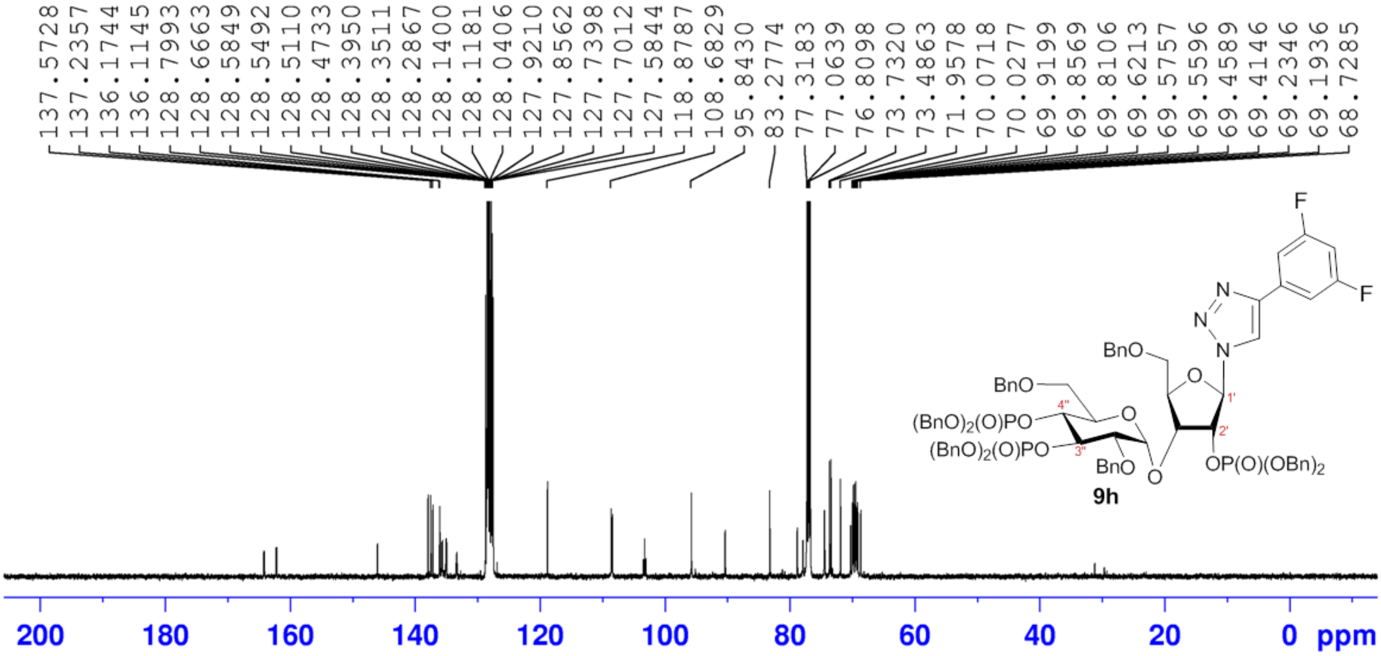

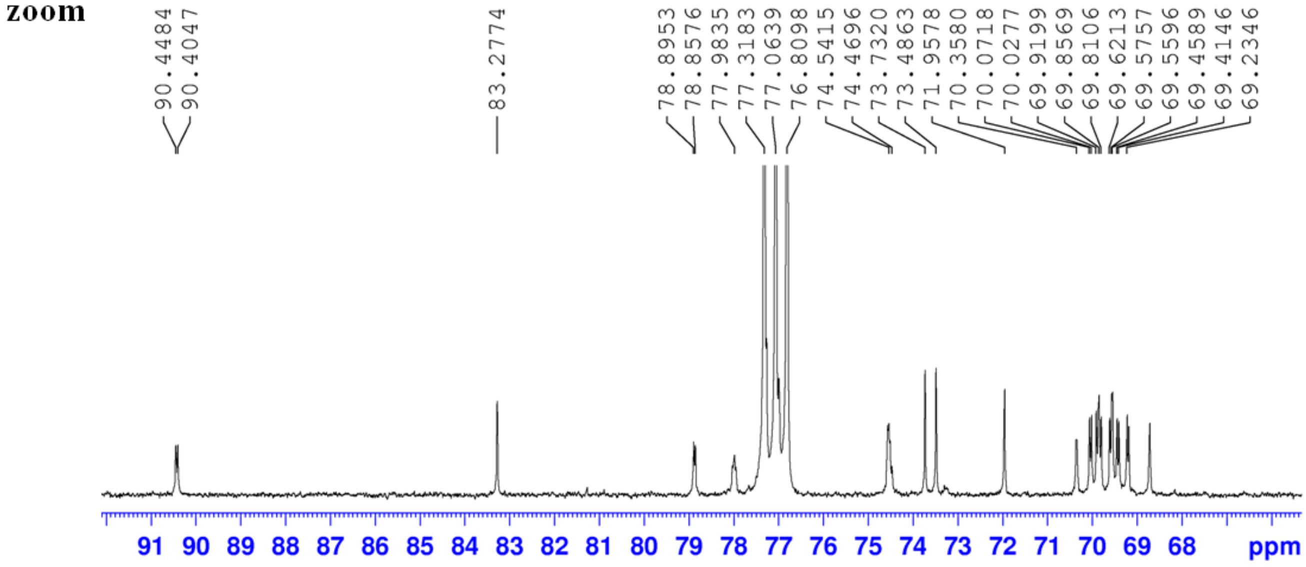

DEPT of 9h in $CDCl_3$

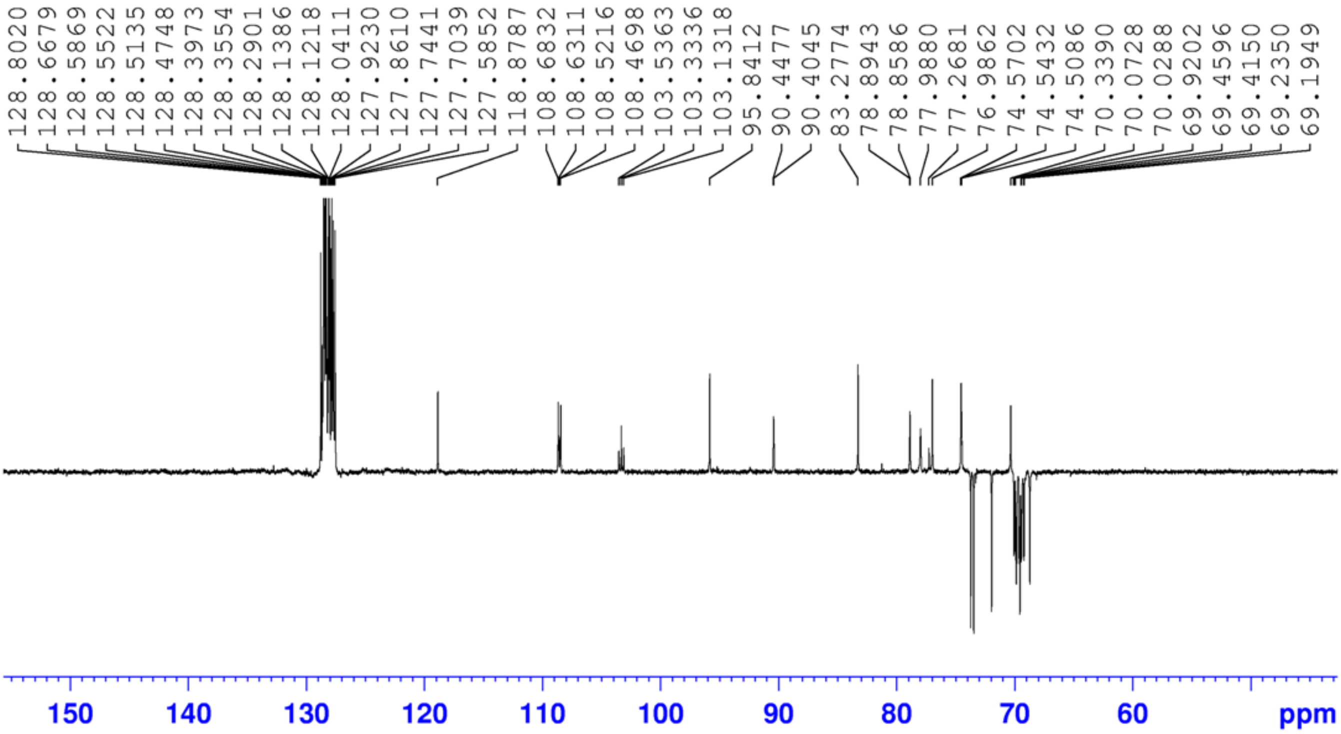

^{31}P NMR of 9h in $CDCl_3$

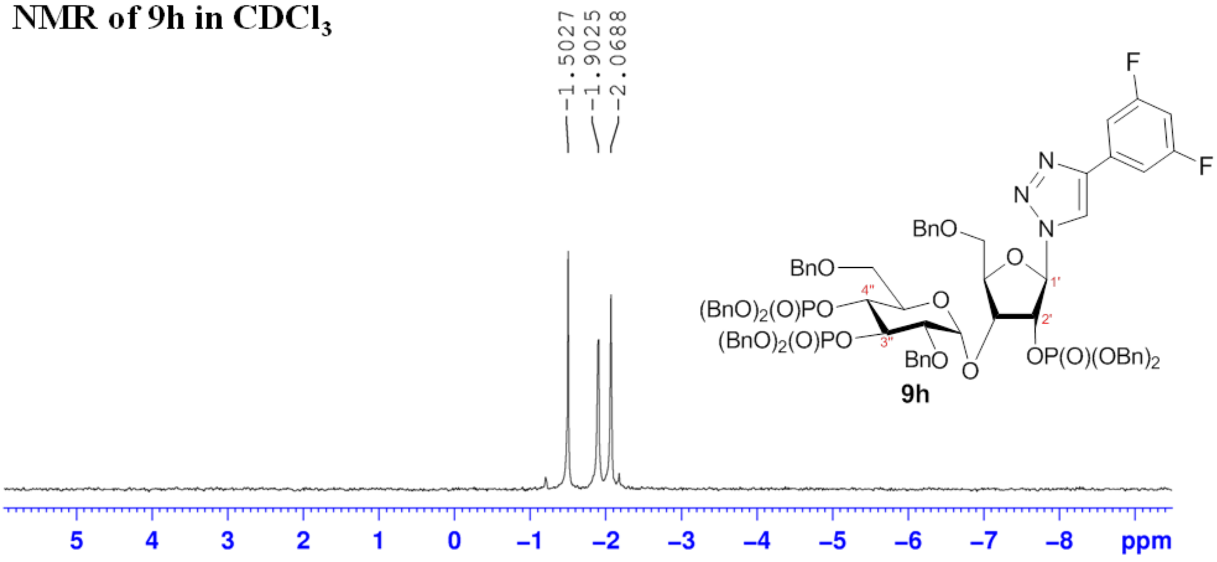

^{19}F NMR of 9h in $CDCl_3$

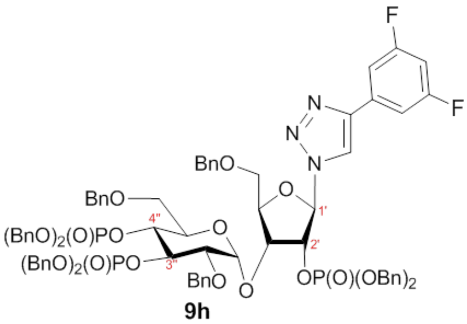

9h

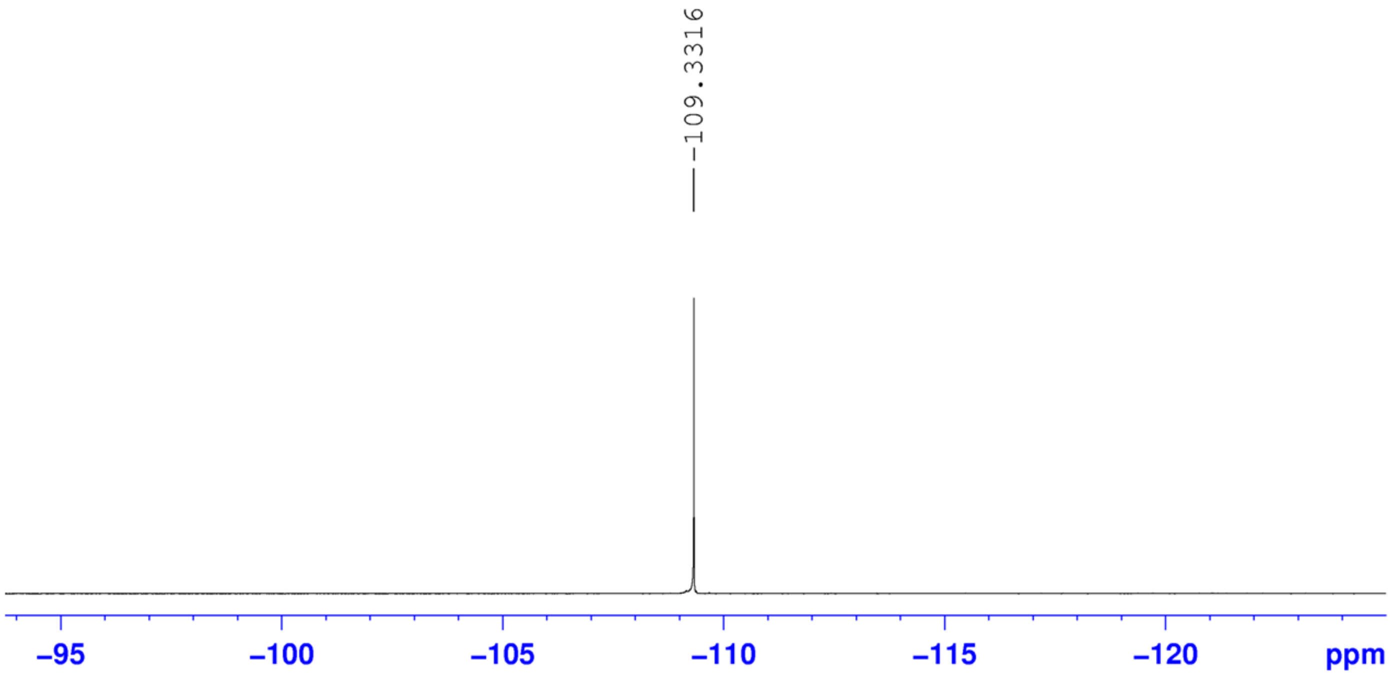

HMBC of 9h in $CDCl_3$

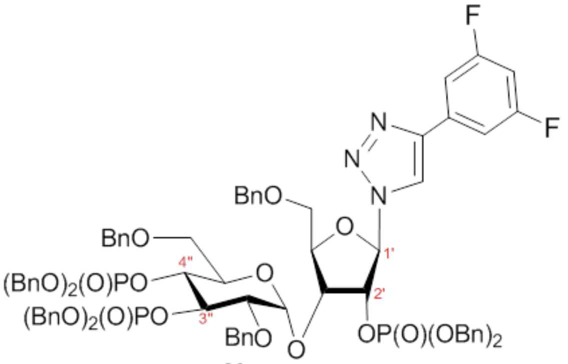

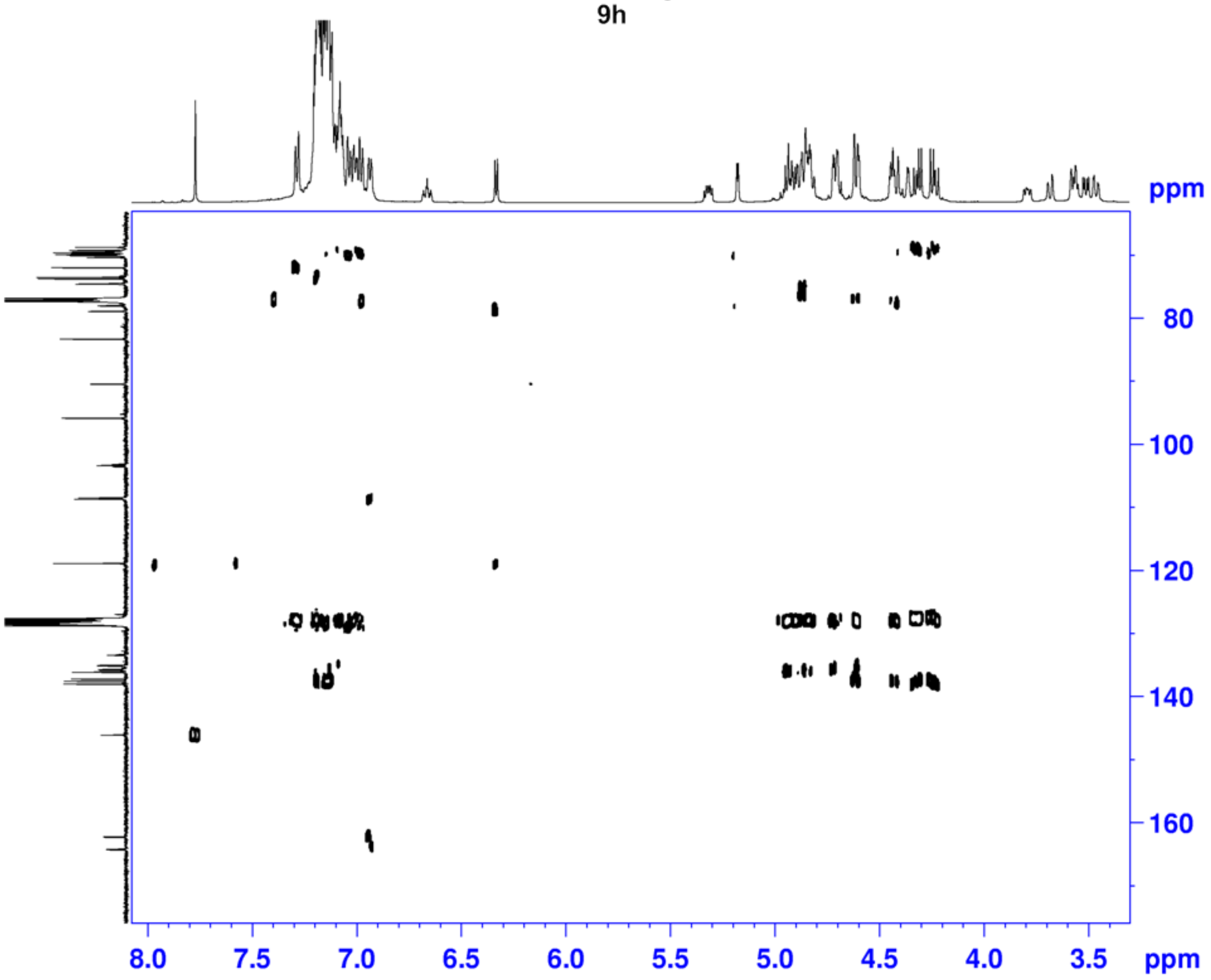

HMQC of 9h in $CDCl_3$

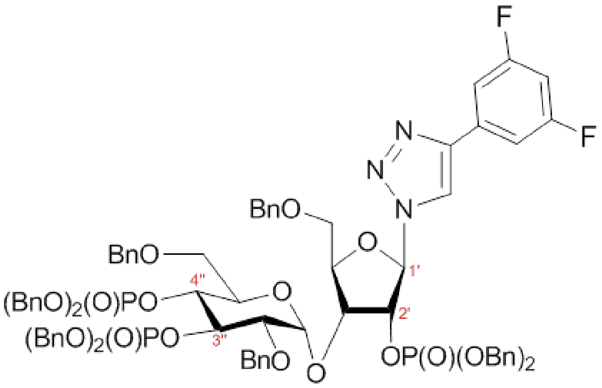

9h

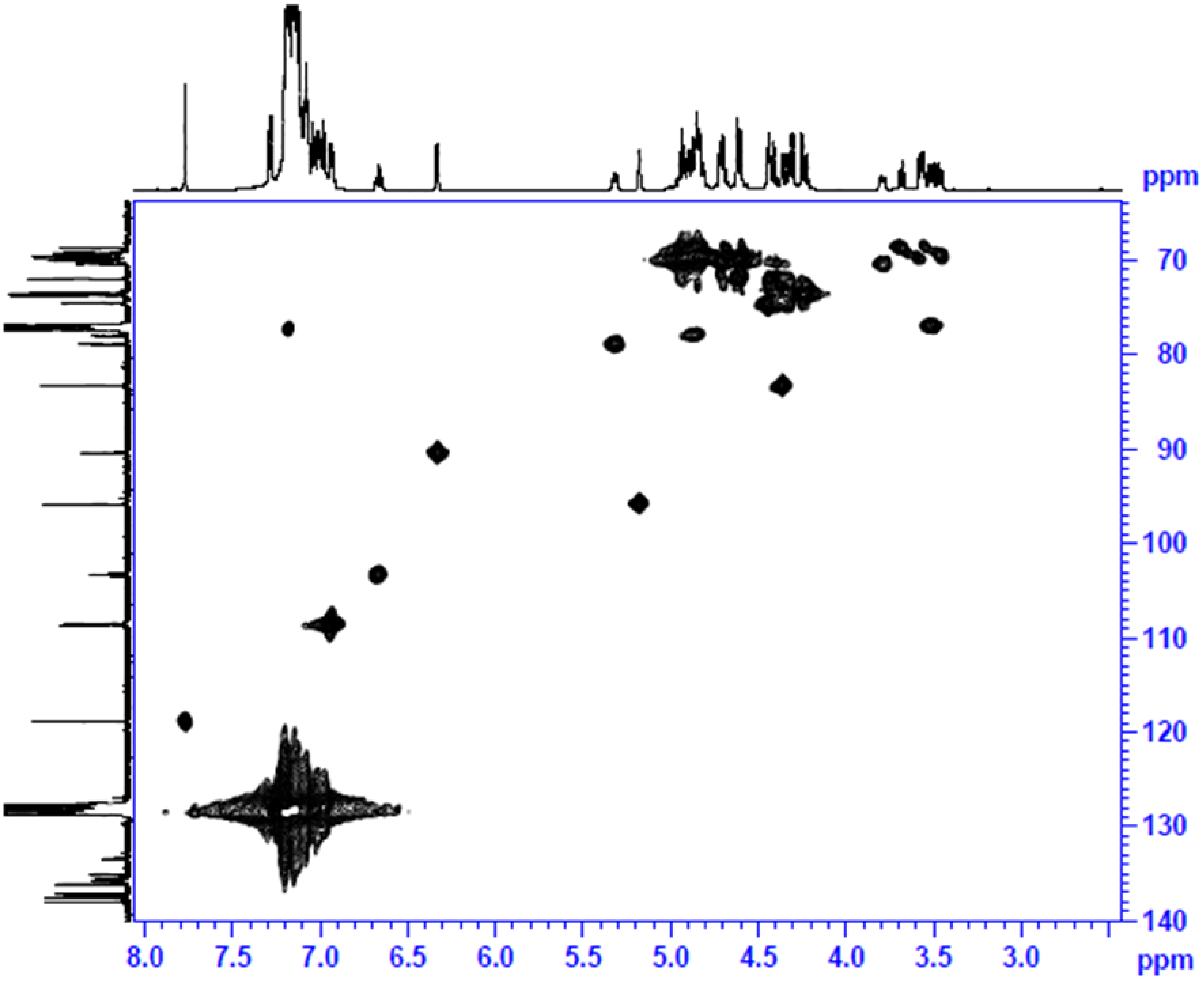

^{1}H NMR of 10h in MeOD

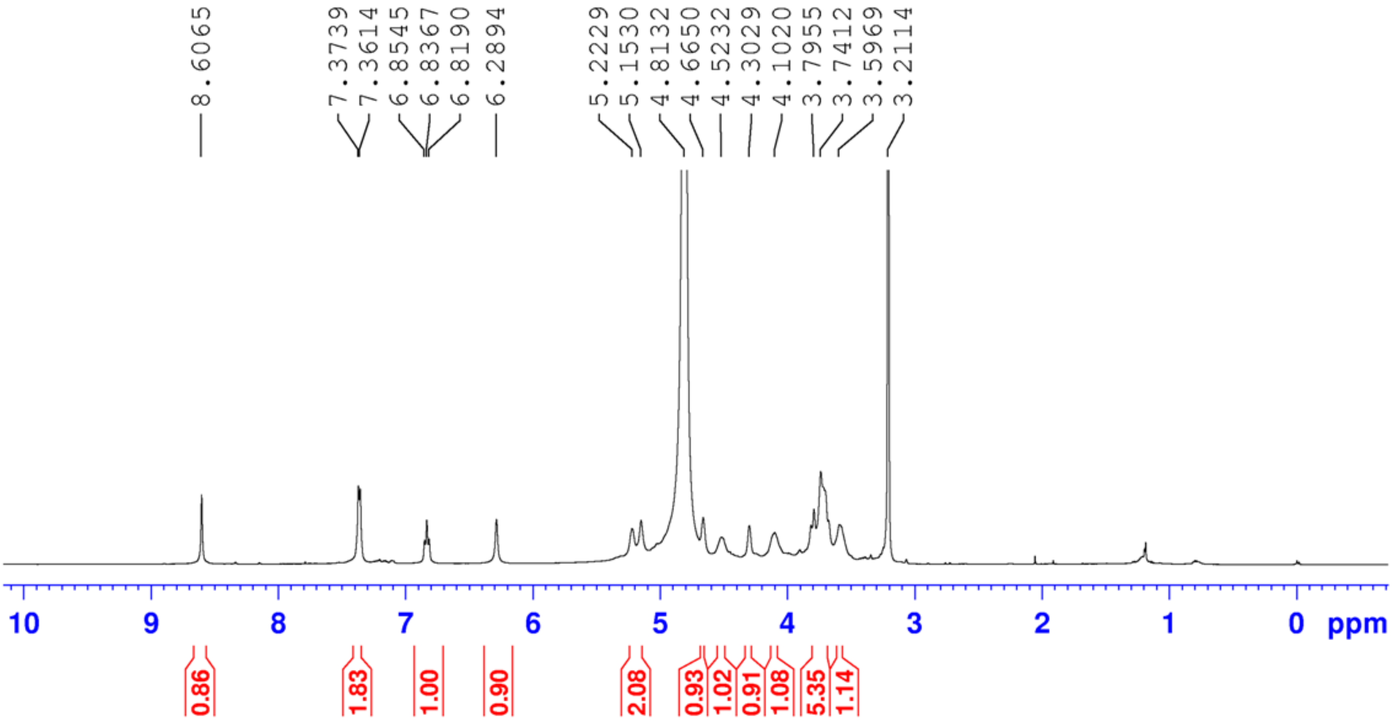

^{19}F NMR of 10h in MeOD

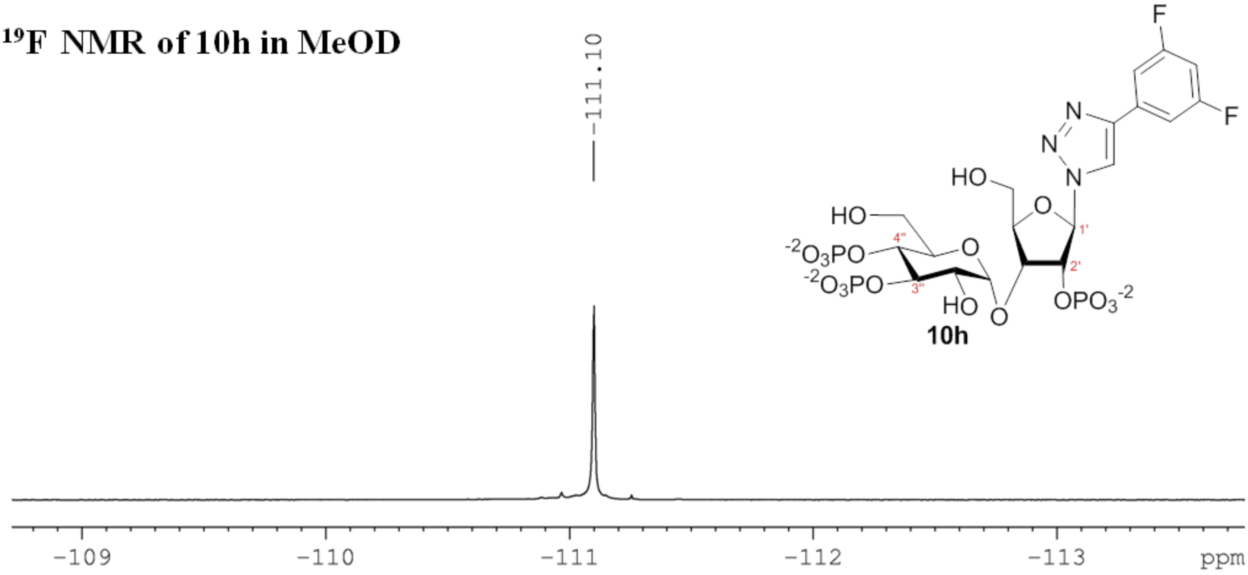

10h

COSY of 10h in MeOD

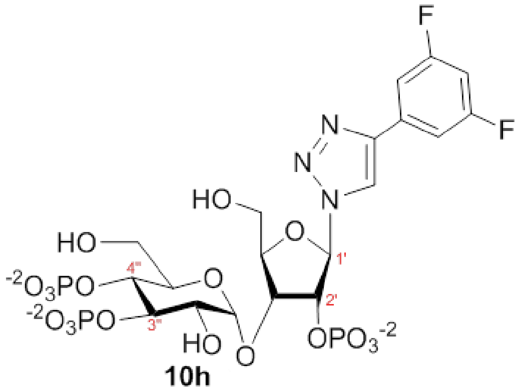

10h

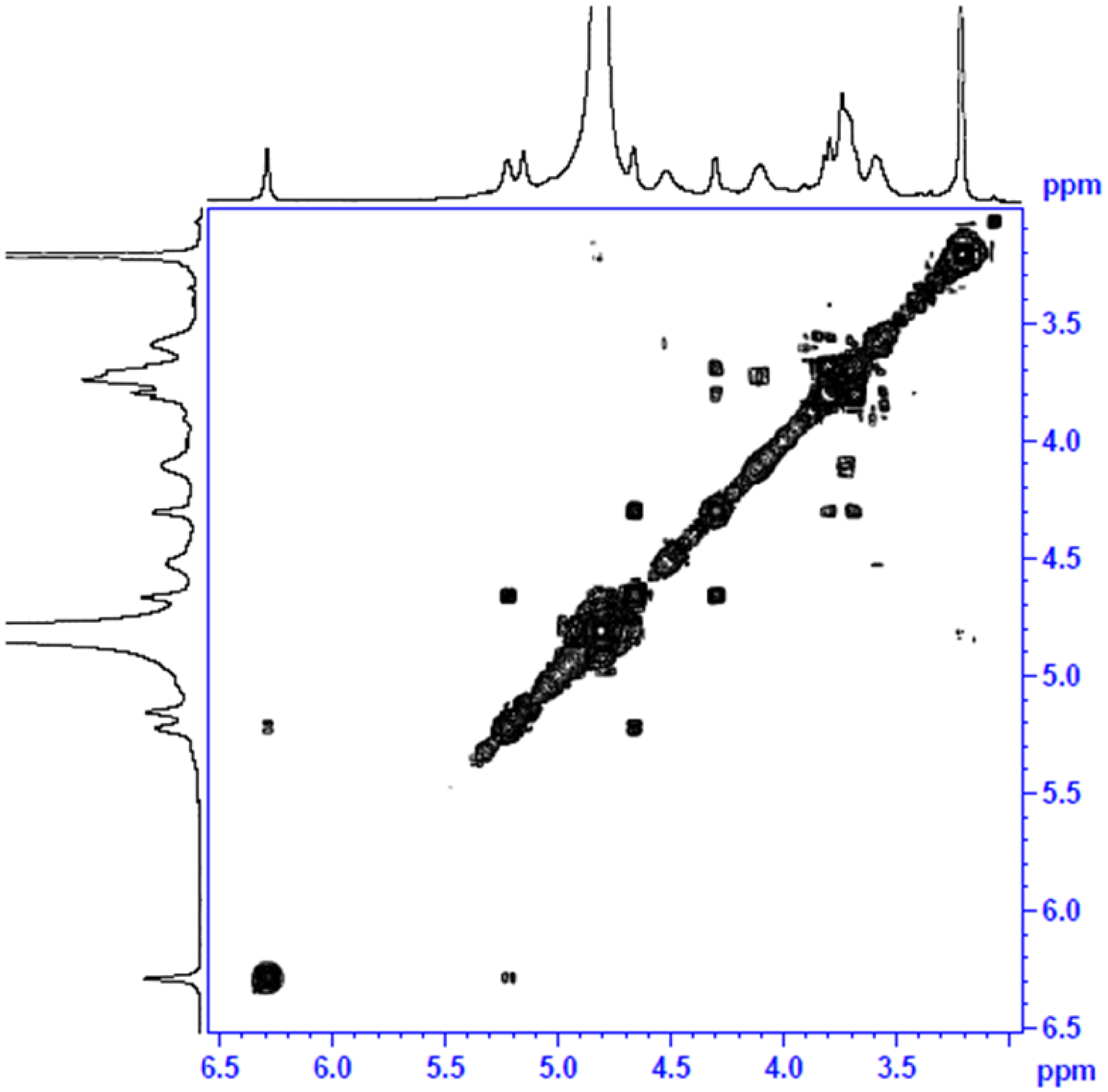

^{13}C NMR of 10h in MeOD

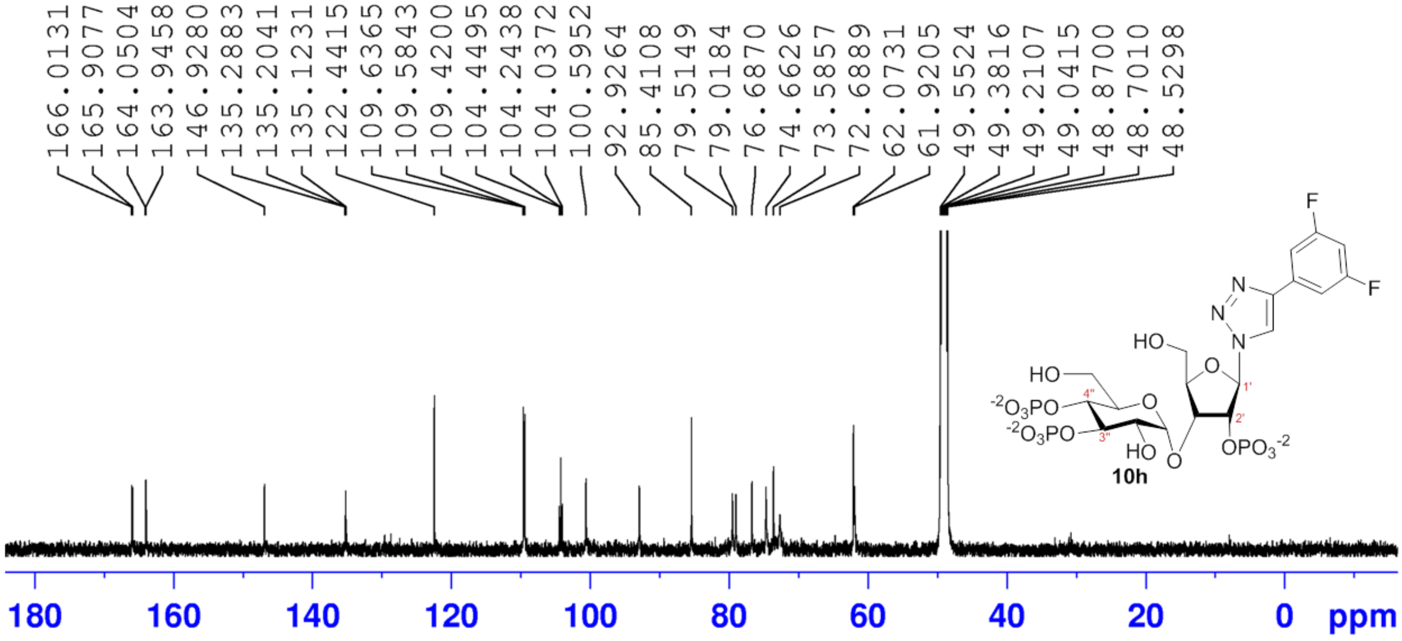

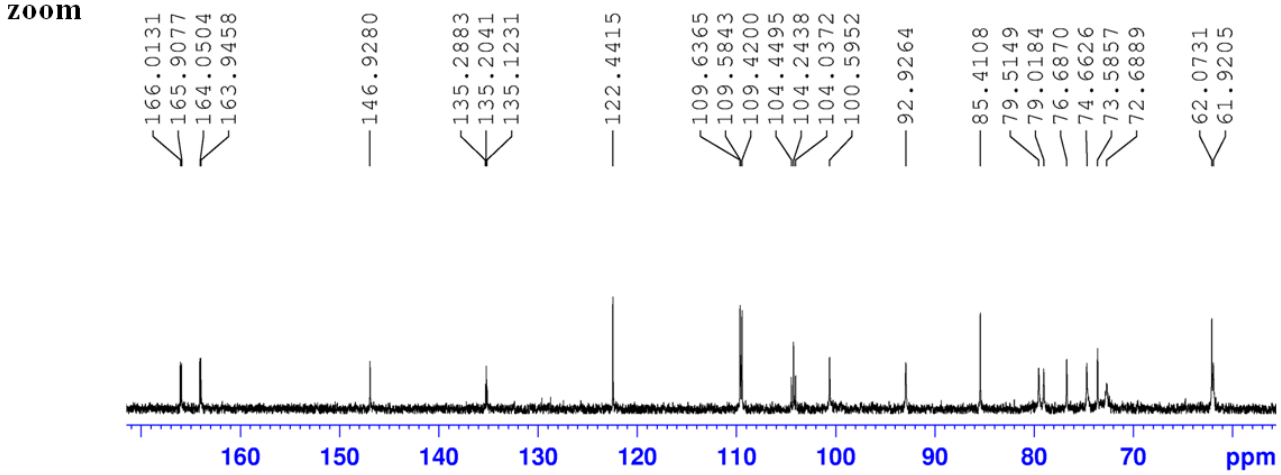

DEPT of 10h in MeOD

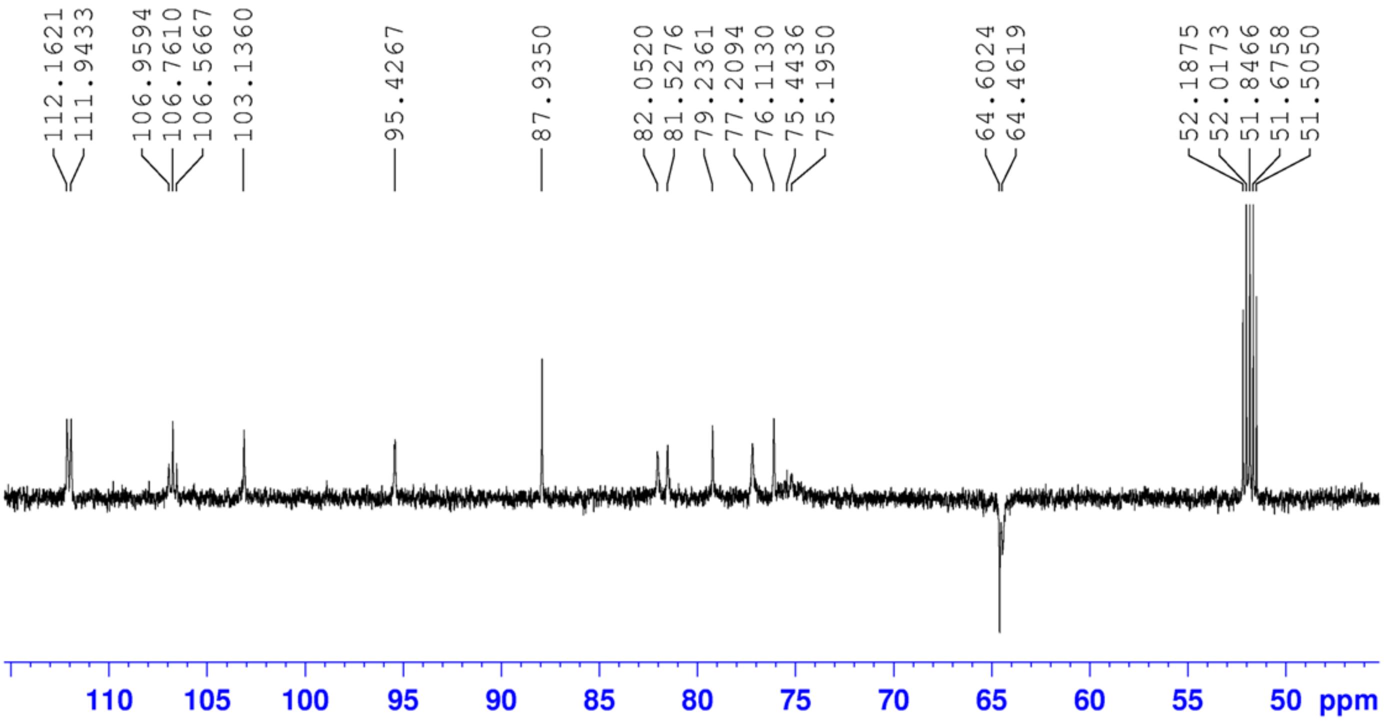

^{31}P NMR of 10h in MeOD

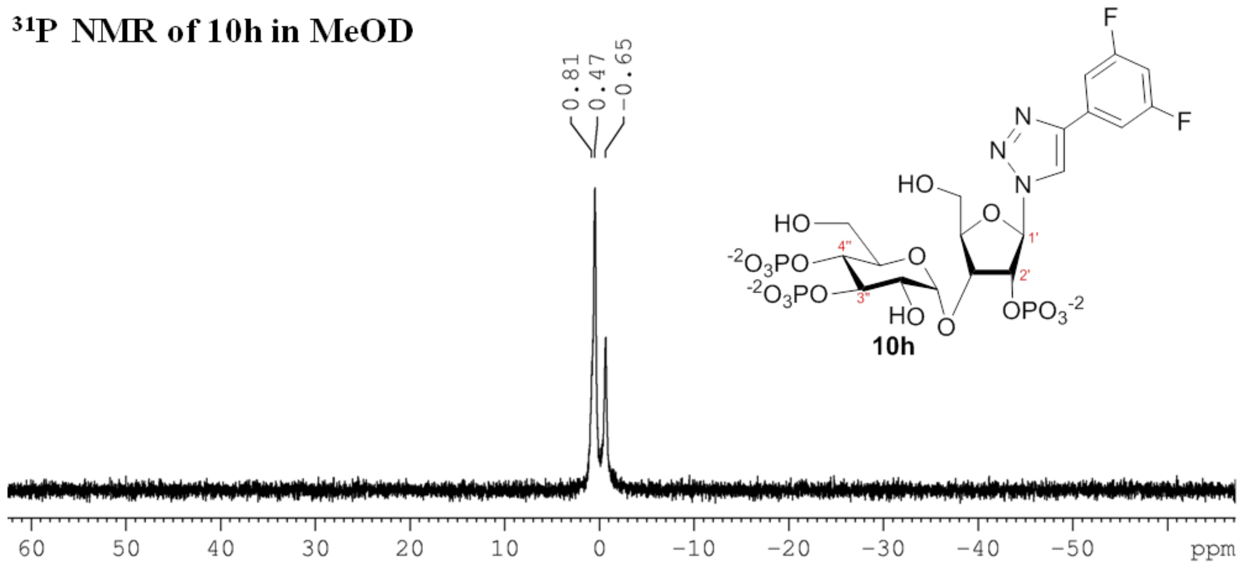

10h

HMBC of 10h in MeOD

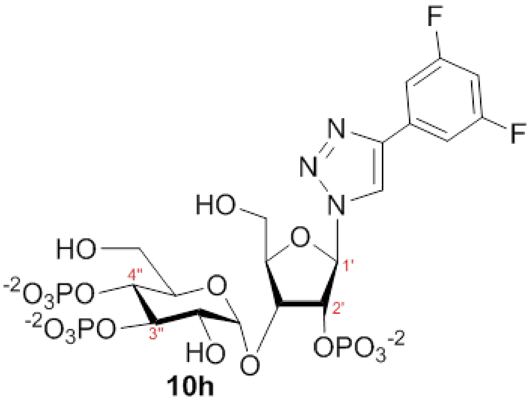

10h

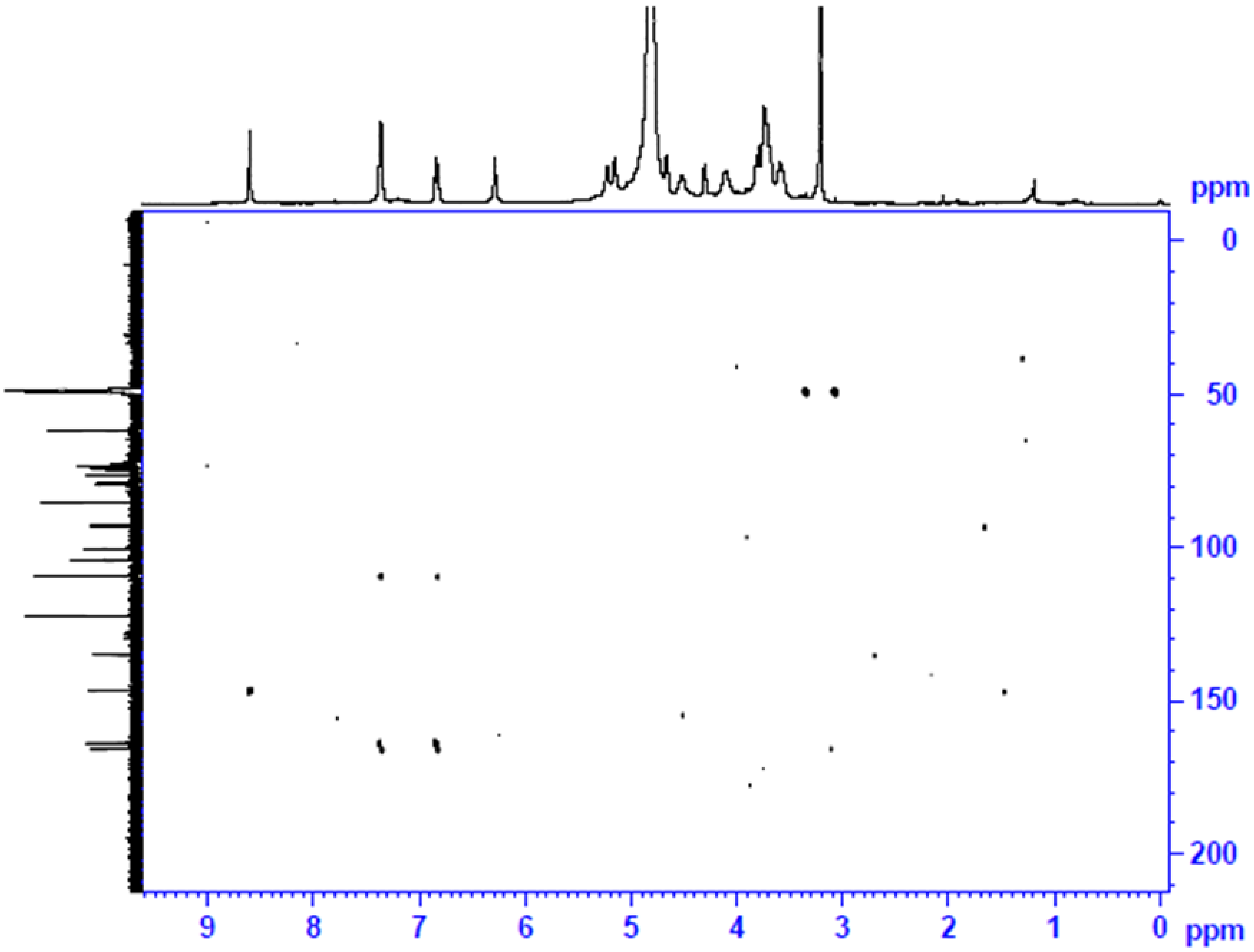

HMQC of 10h in MeOD

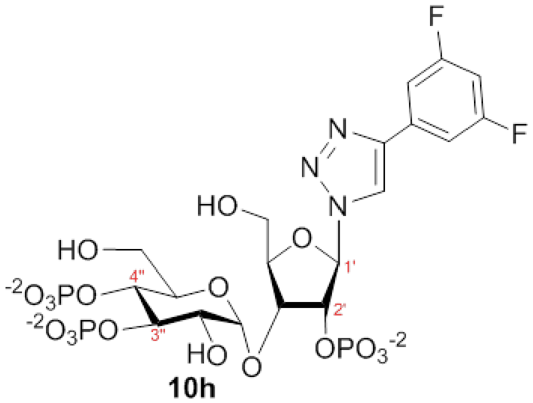

10h

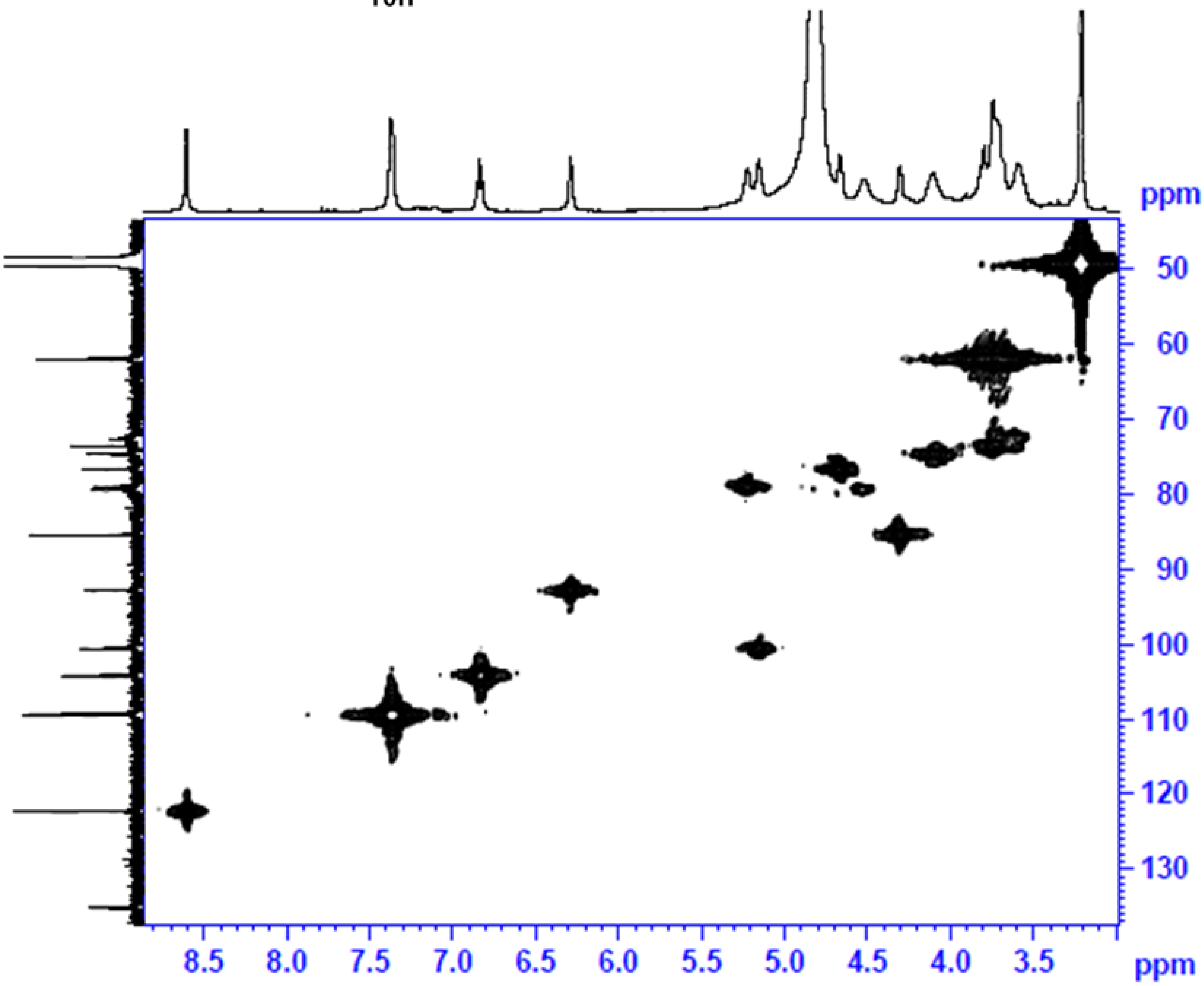

^{1}H NMR of 9i in $CDCl_3$

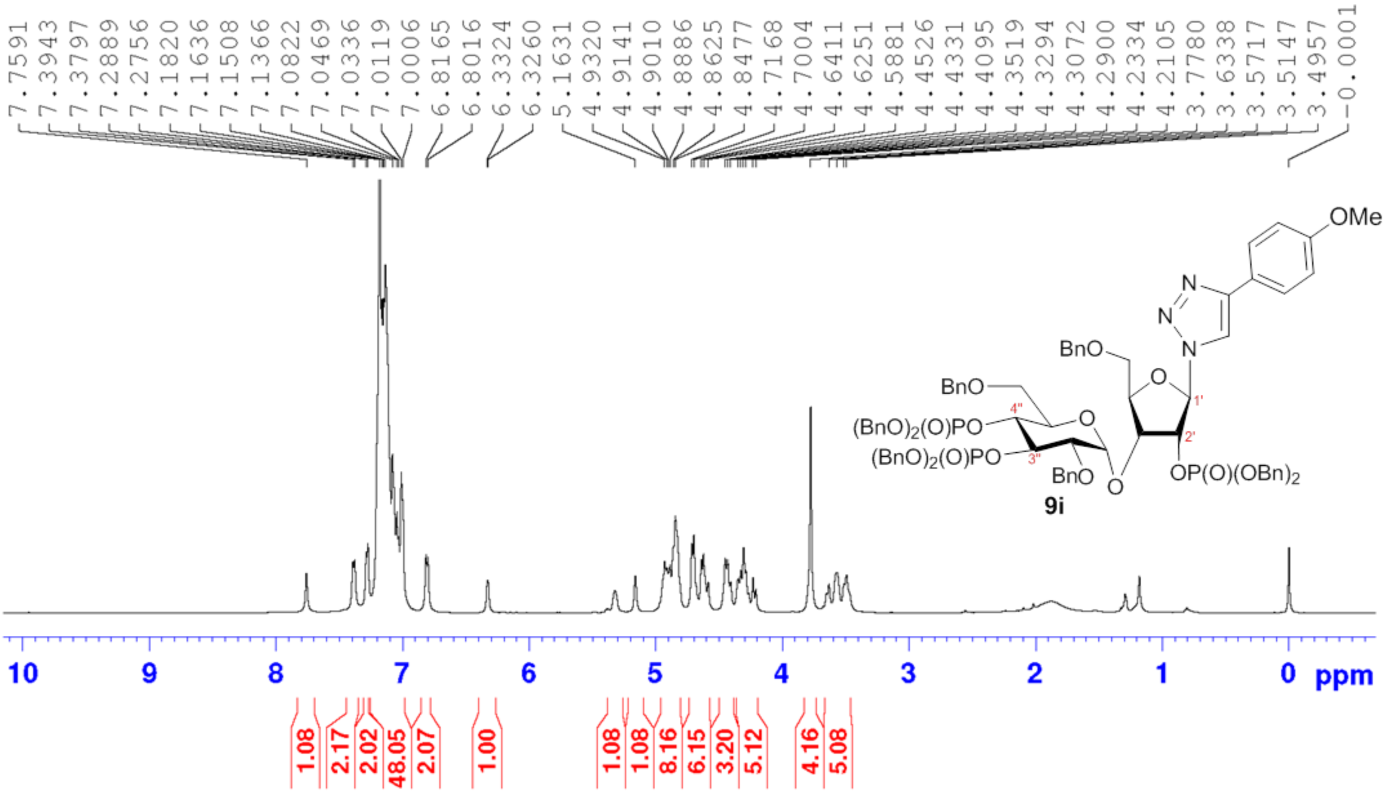

zoom

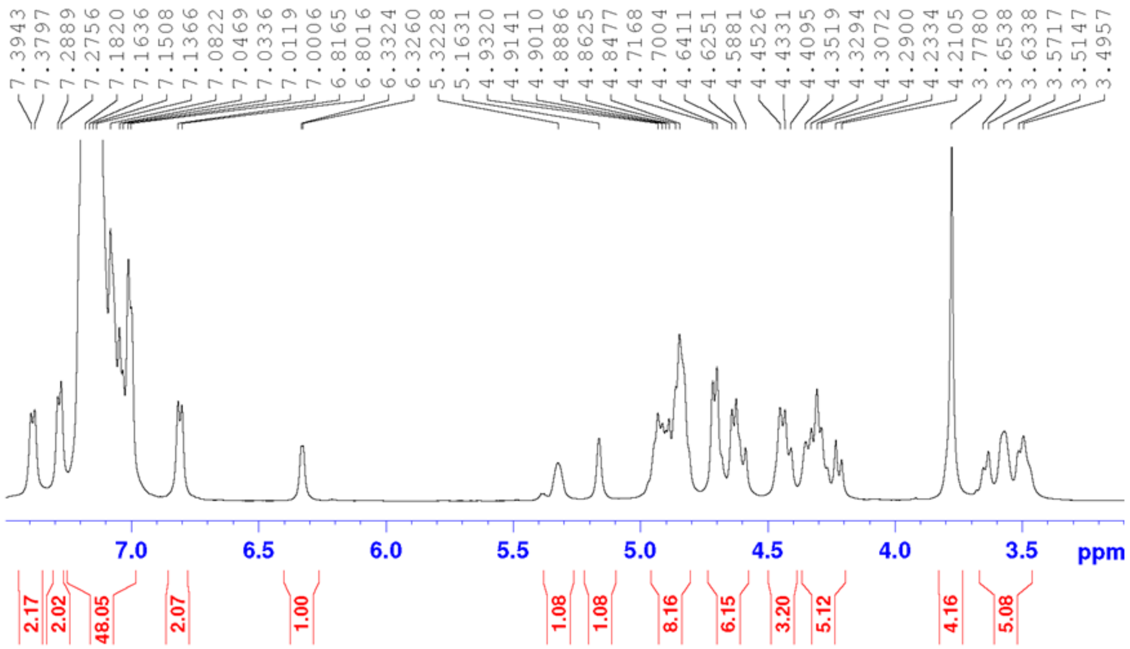

COSY of 9i in $CDCl_3$

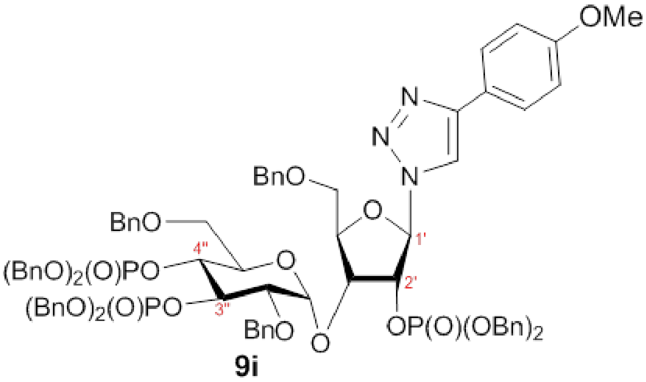

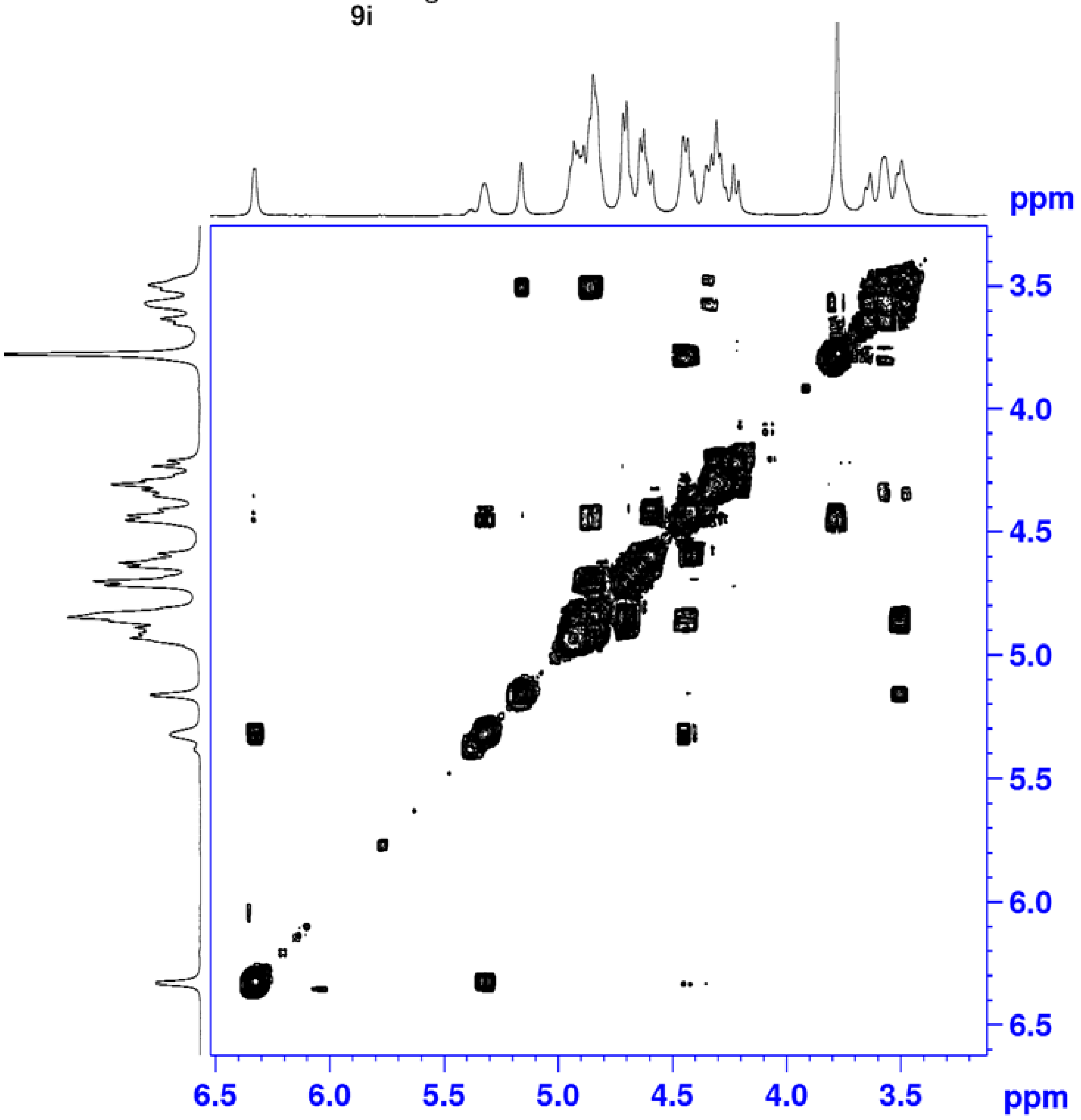

^{13}C NMR of 9i in $CDCl_3$

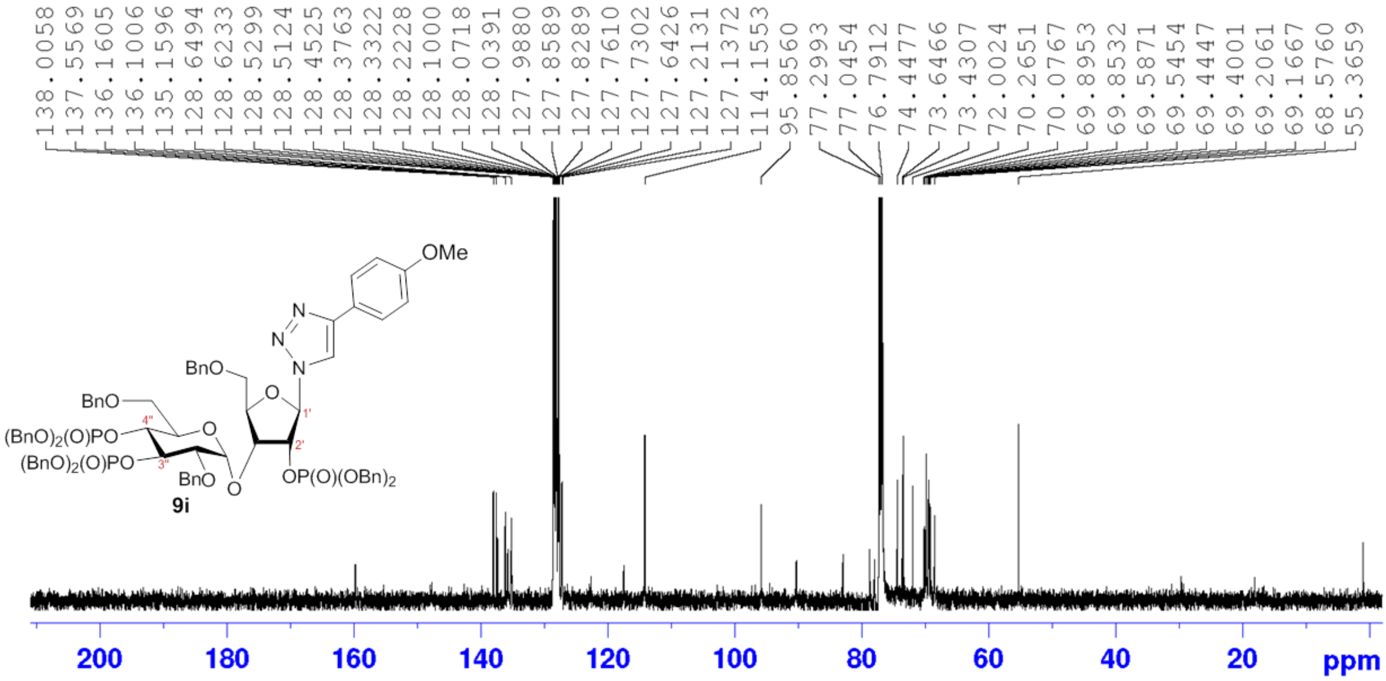

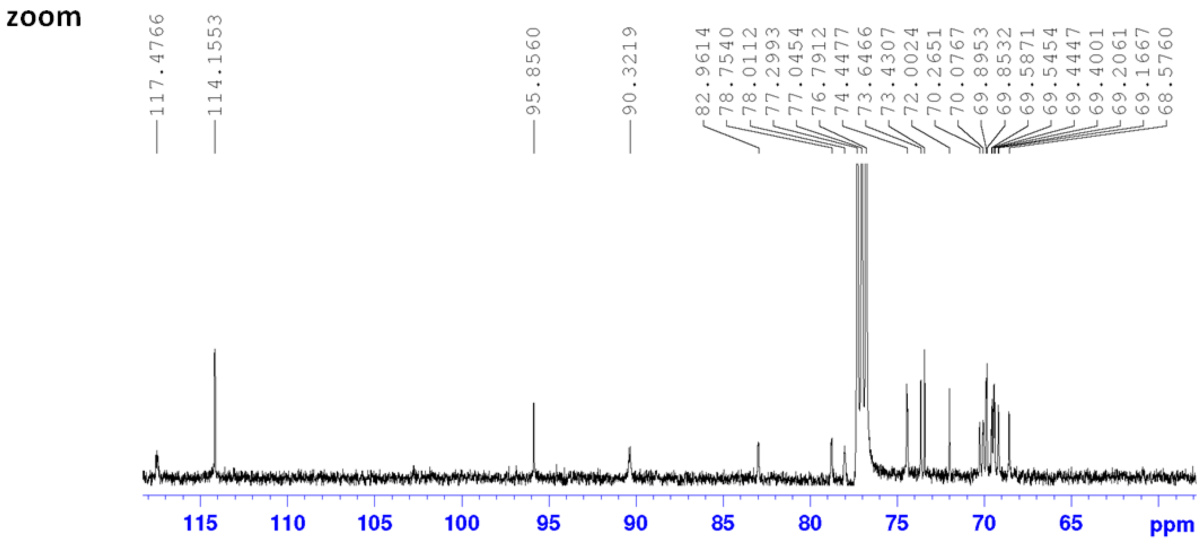

DEPT of 9i in $CDCl_3$

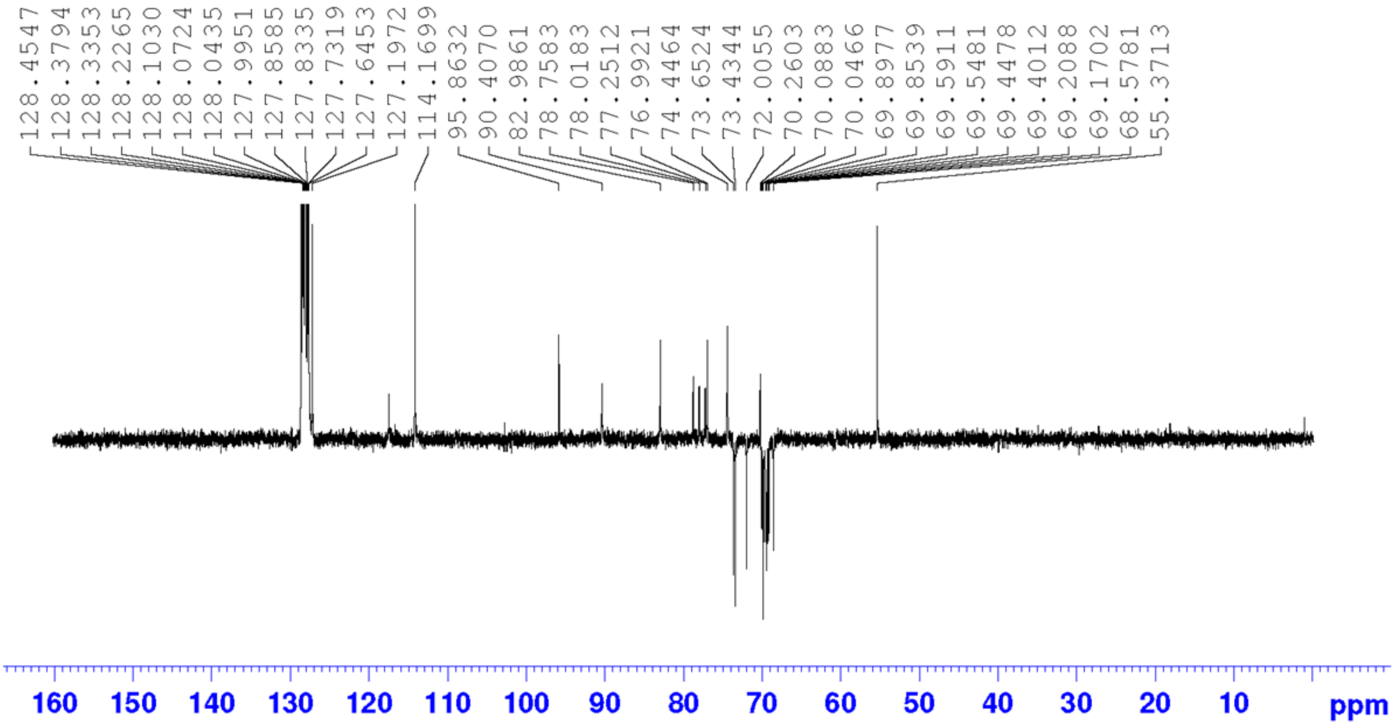

^{31}P NMR of 9i in $CDCl_3$

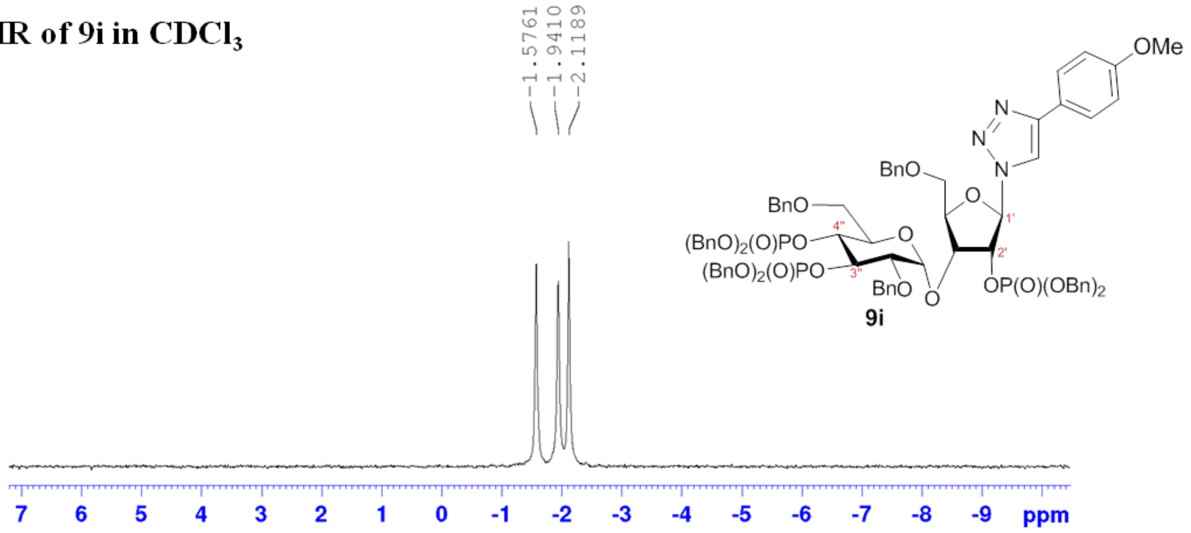

HMBC of 9i in $CDCl_3$

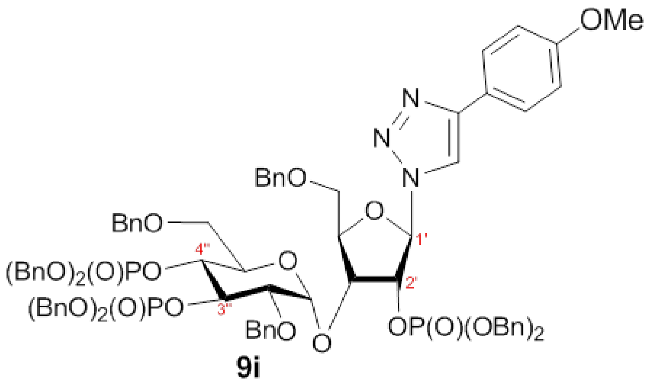

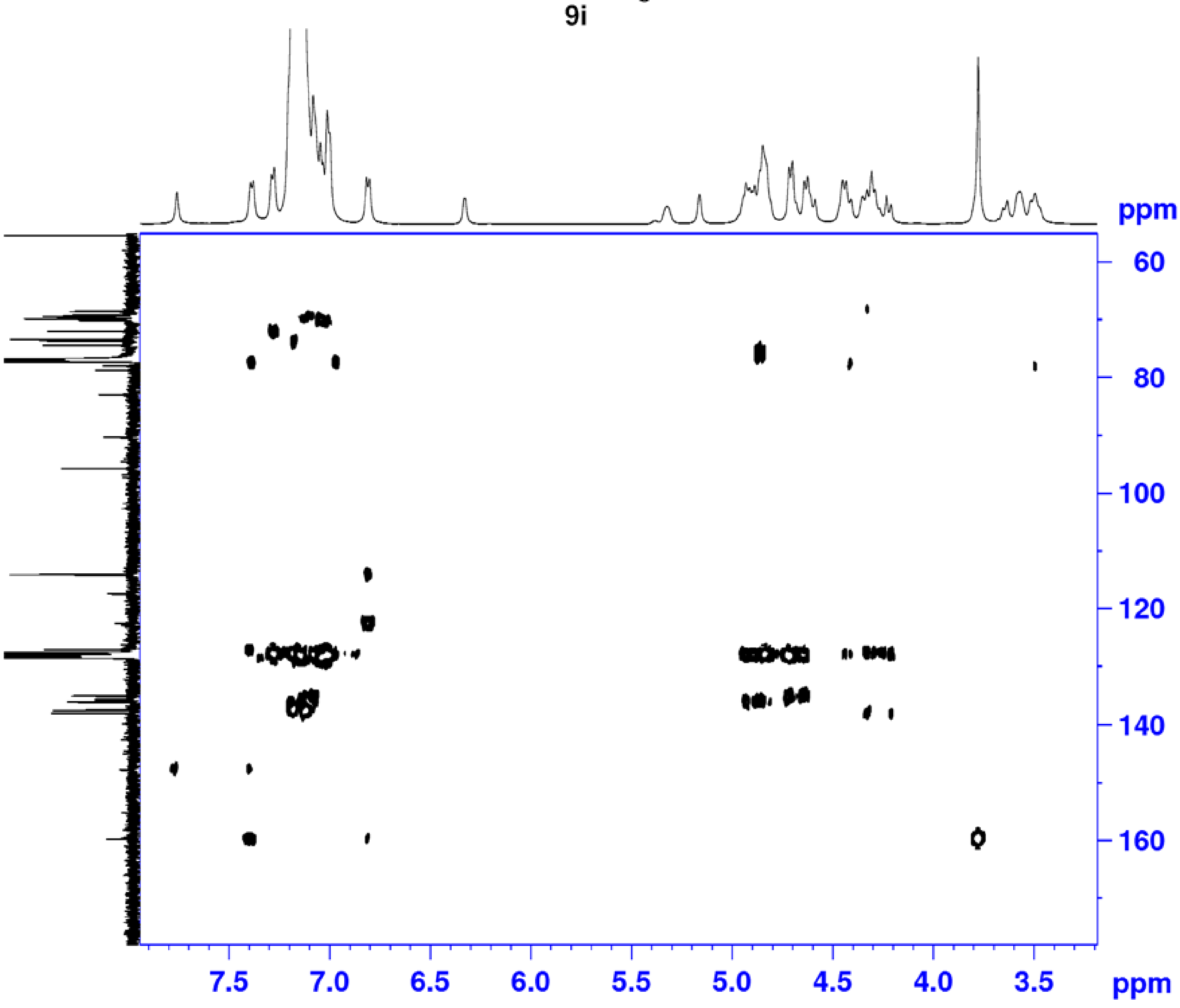

HMQC of 9i in $CDCl_3$

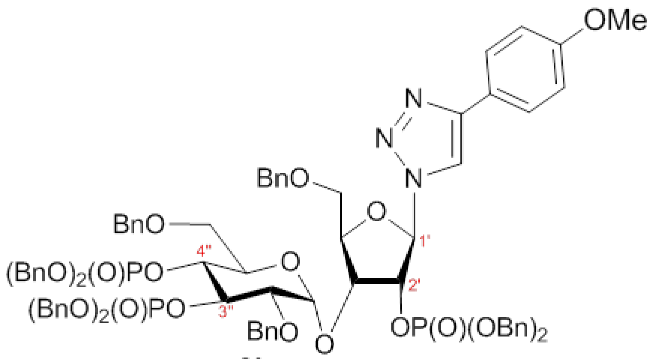

9i

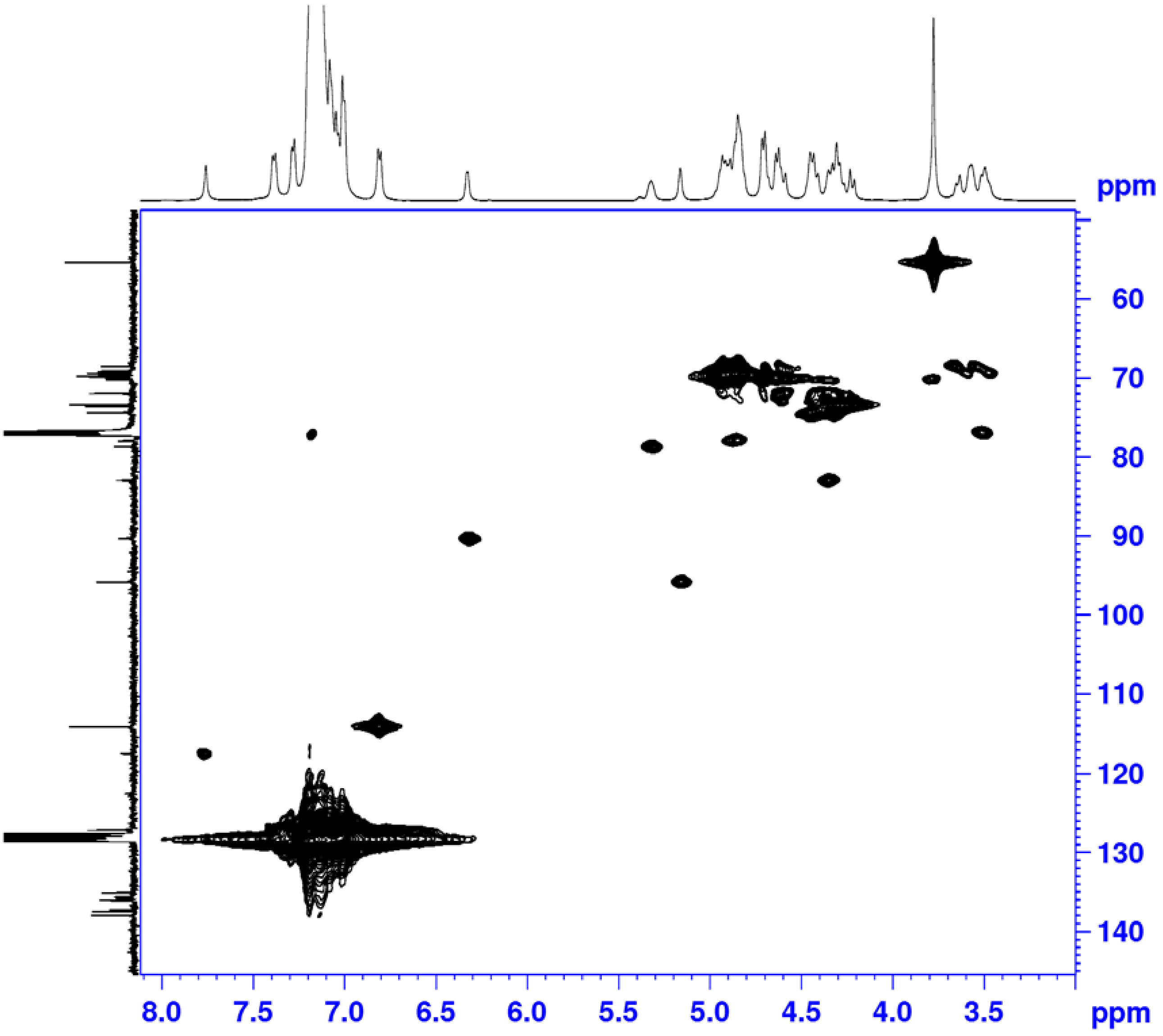

^{1}H NMR of 10i in D_2O

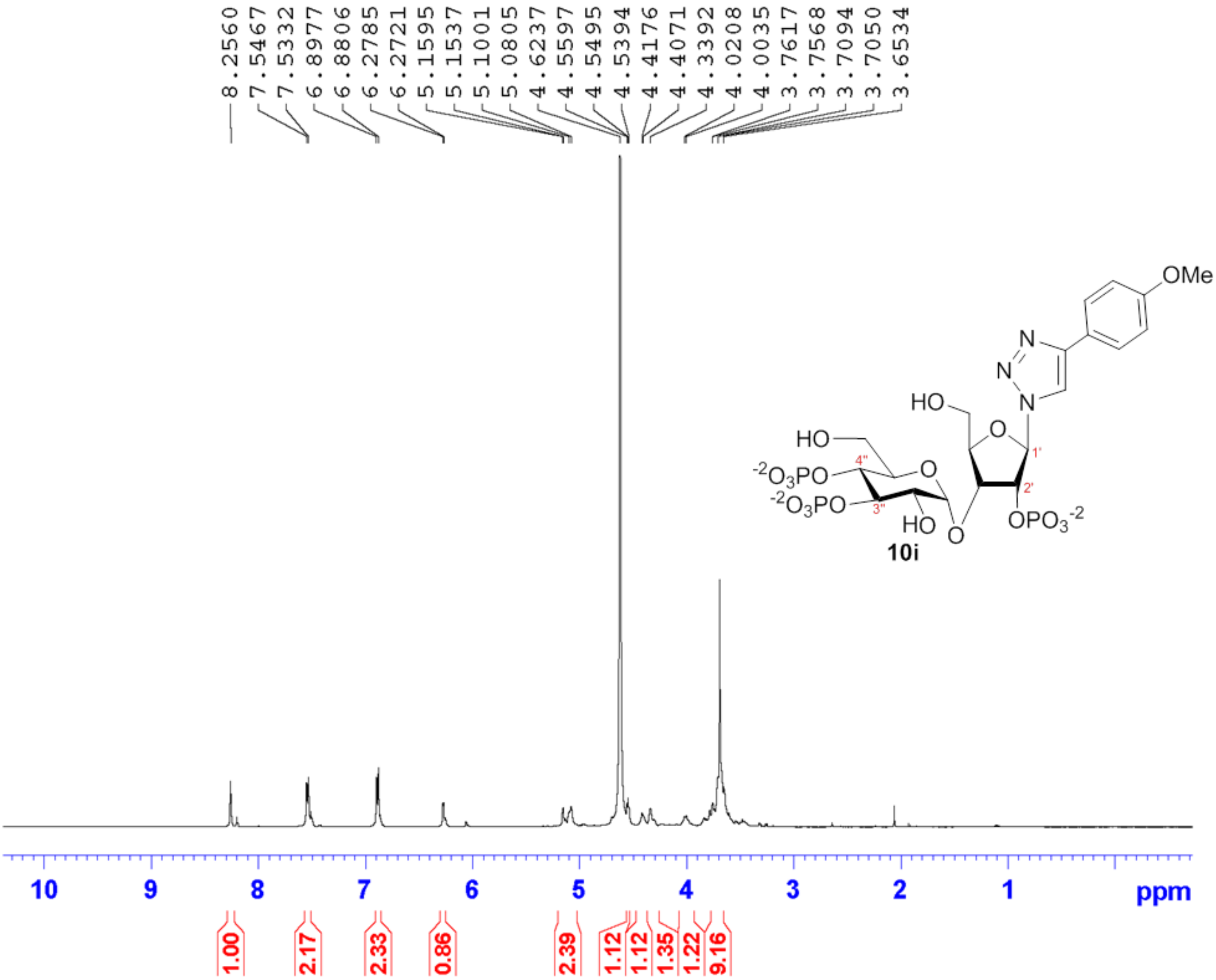

COSY of 10i in D_2O

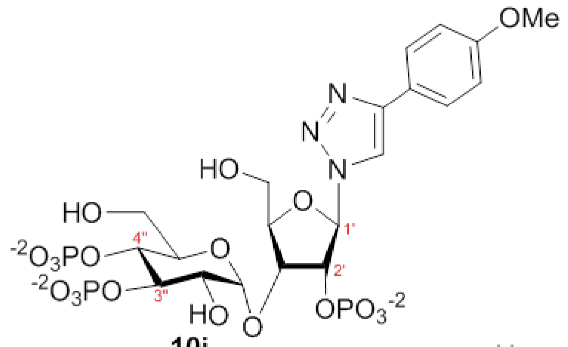

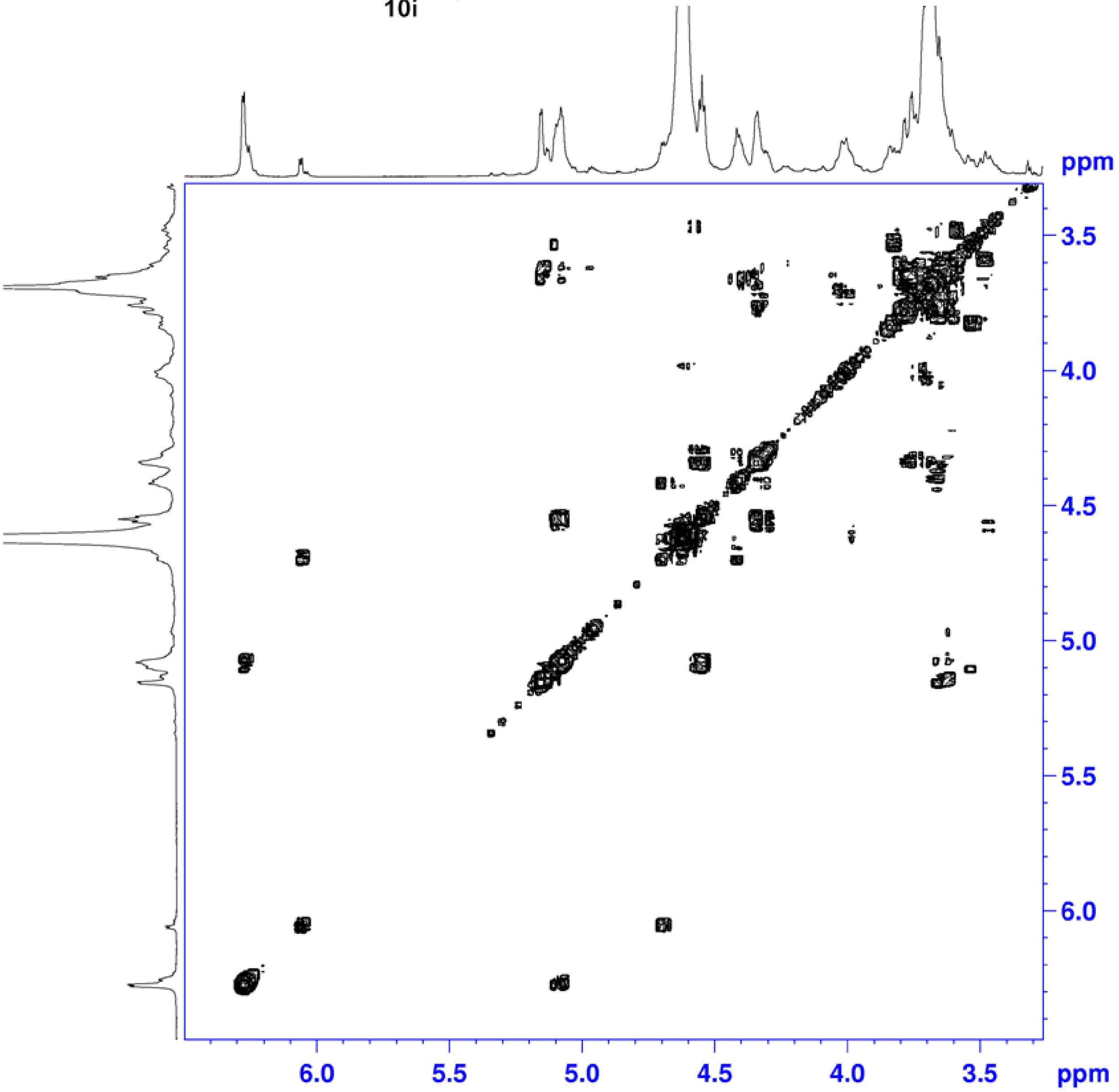

^{13}C NMR of 10i in D_2O

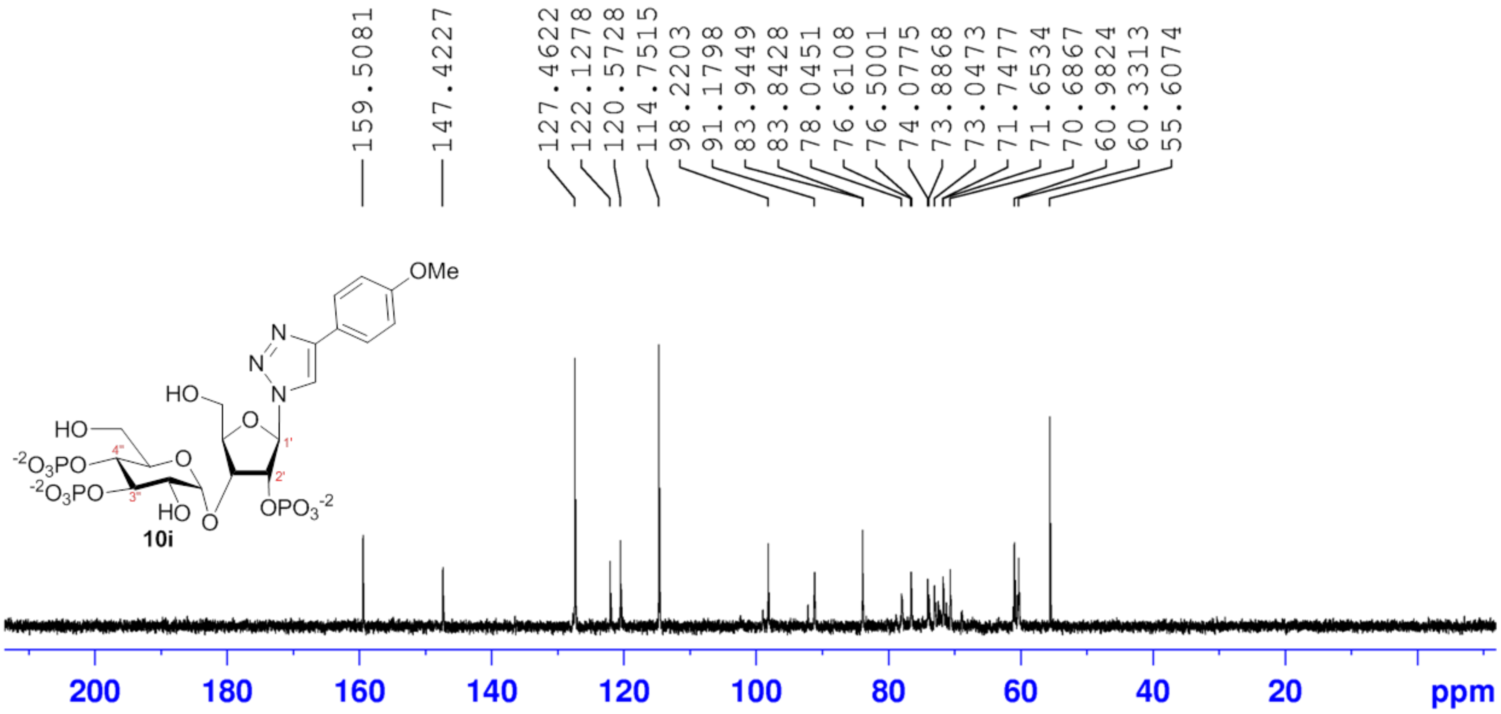

DEPT of 10i in D_2O

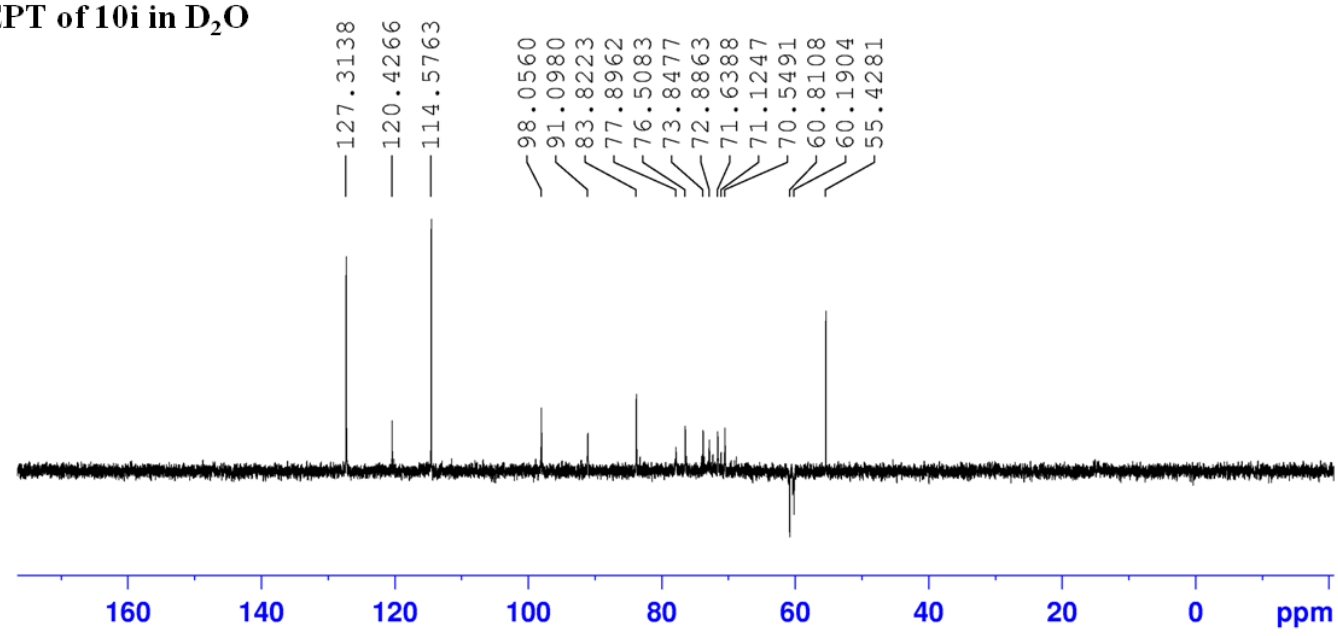

^{31}P NMR of 10i in MeOD

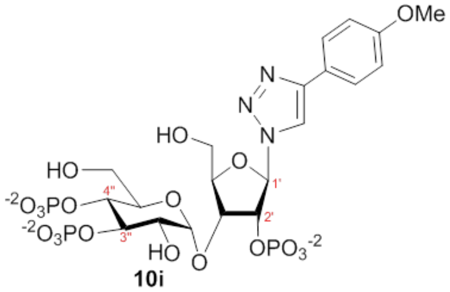

10i

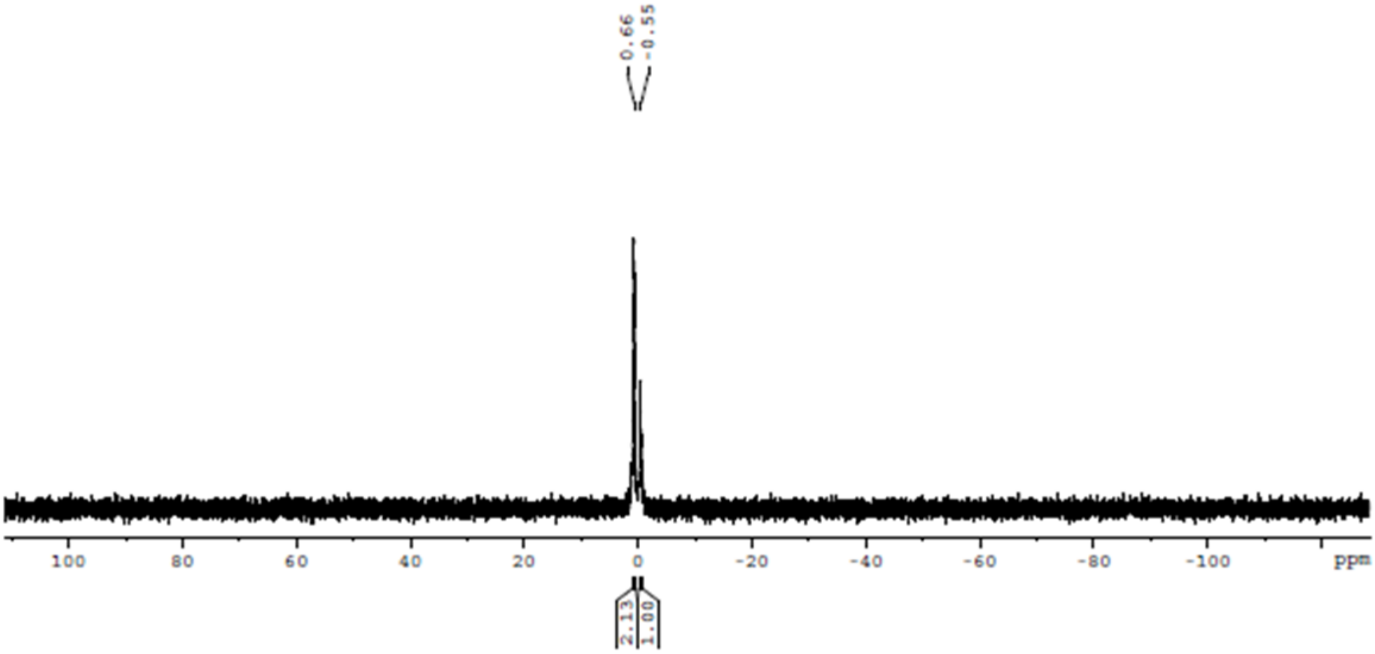

HMBC of 10i in D_2O

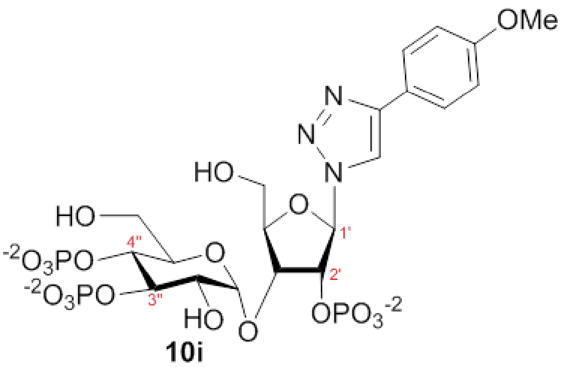

10i

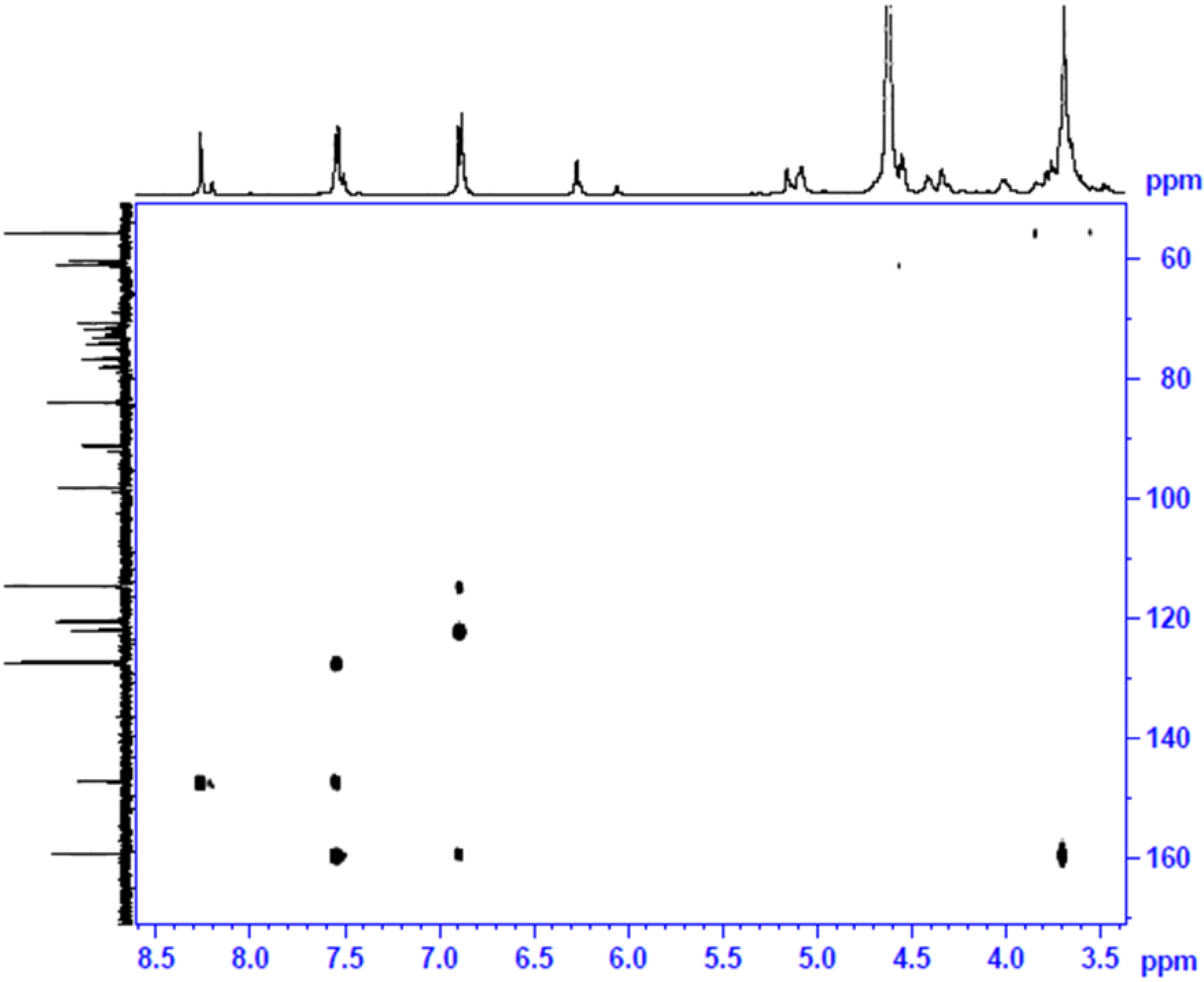

HMQC of 10i in D_2O

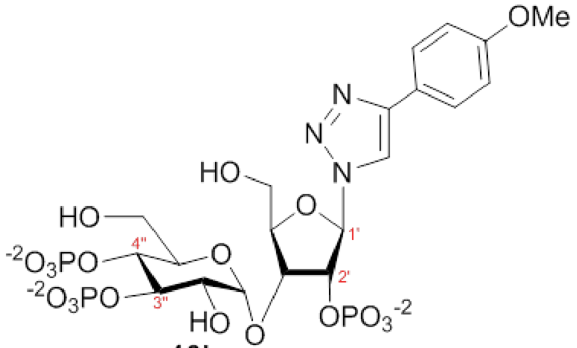

10i

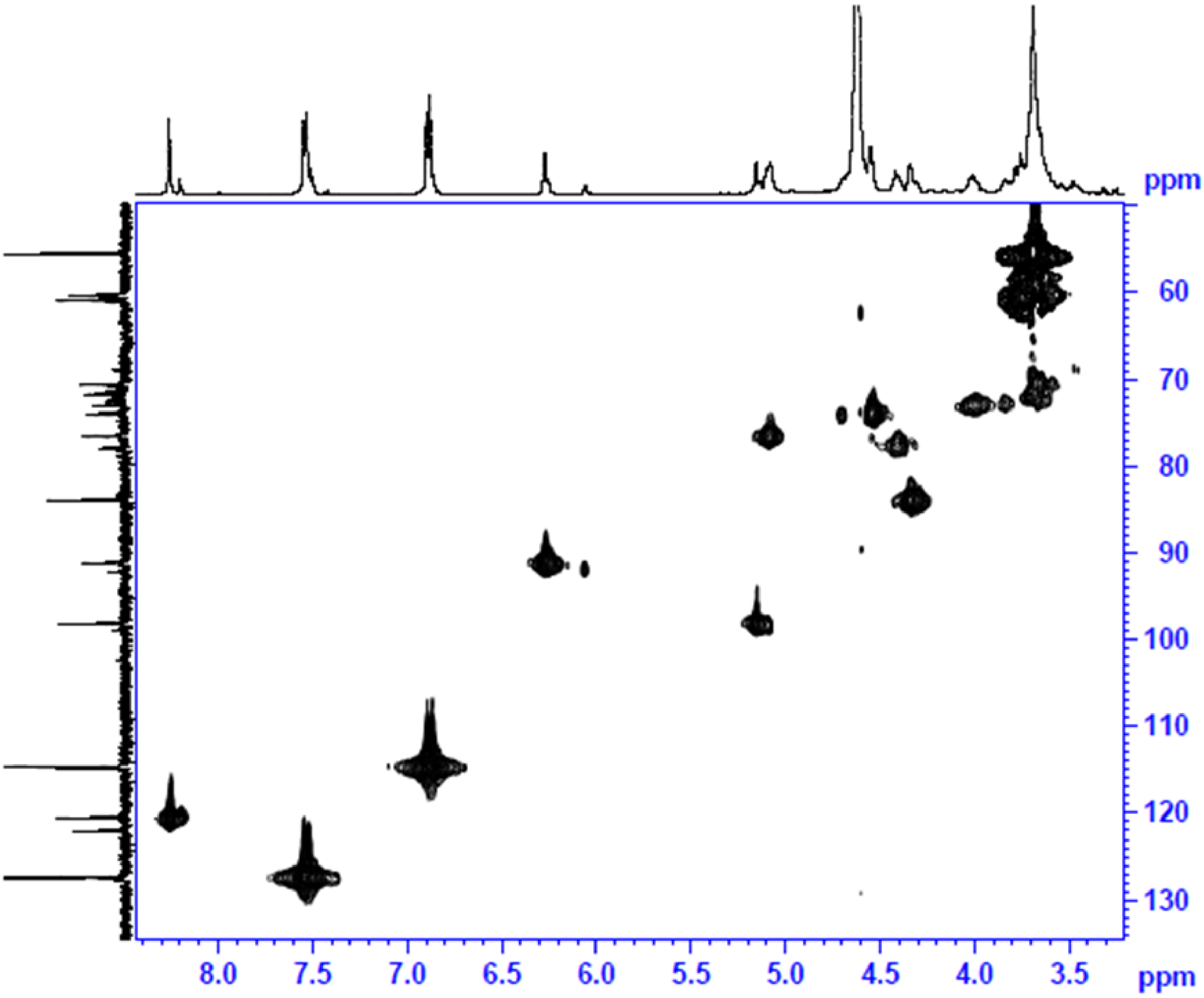

^{1}H NMR of 9j in $CDCl_3$

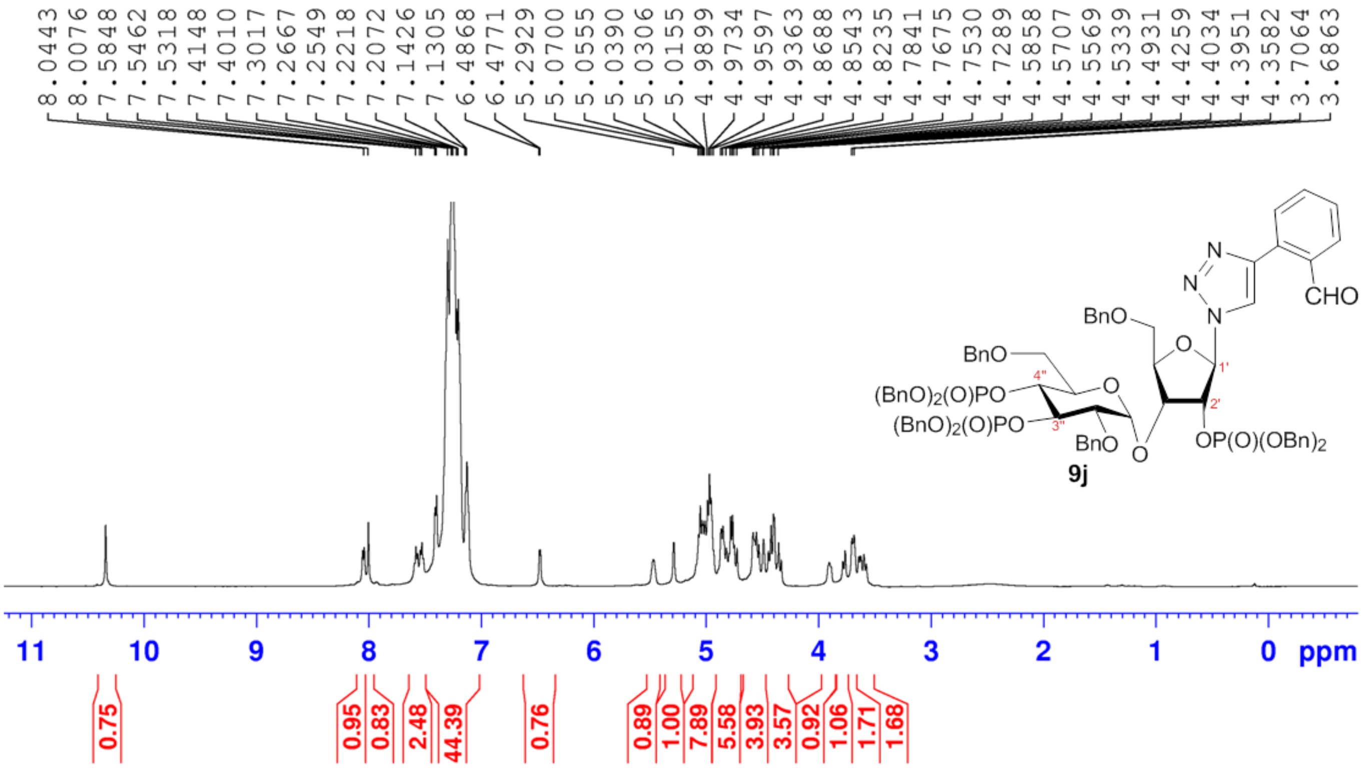

zoom

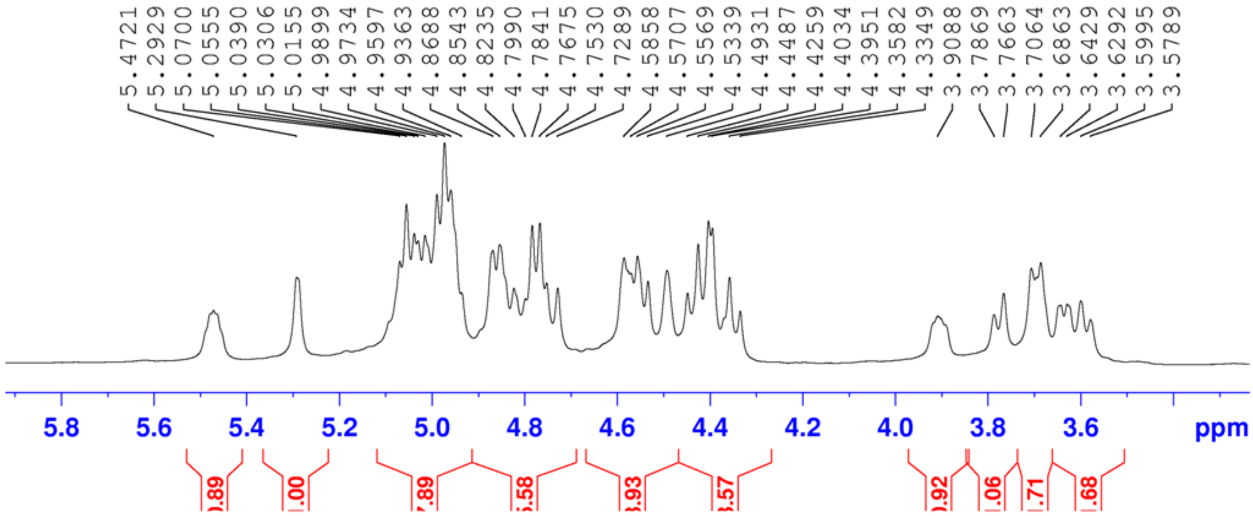

COSY of 9j in $CDCl_3$

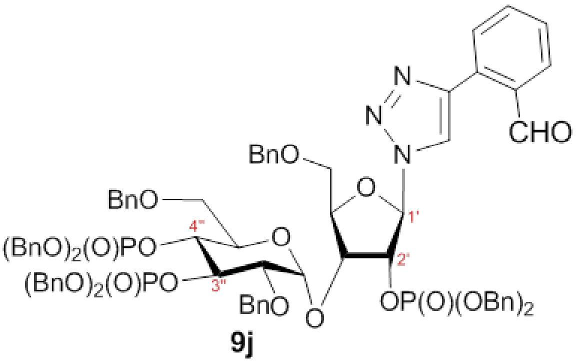

9j

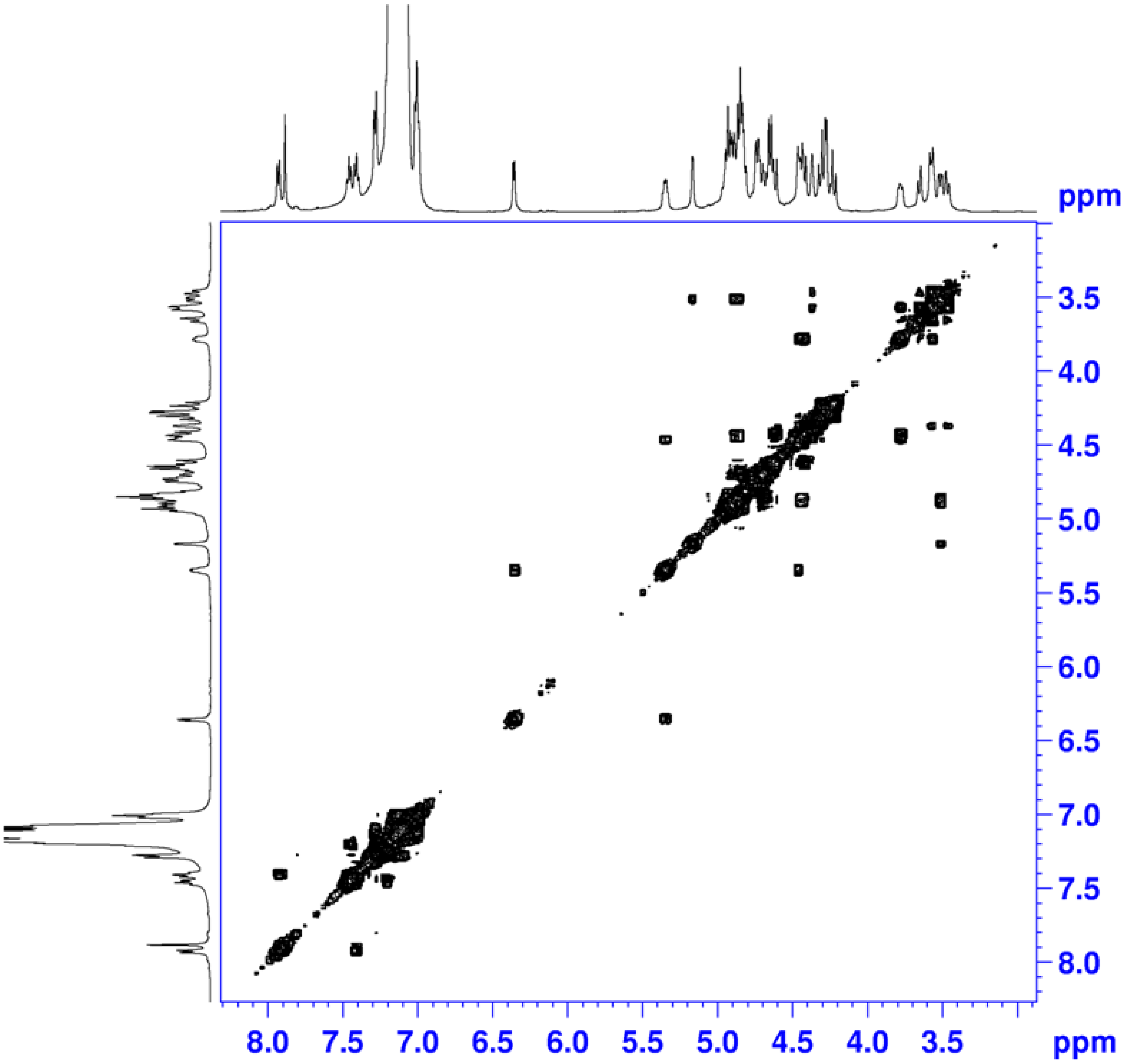

^{13}C NMR of 9j in $CDCl_3$

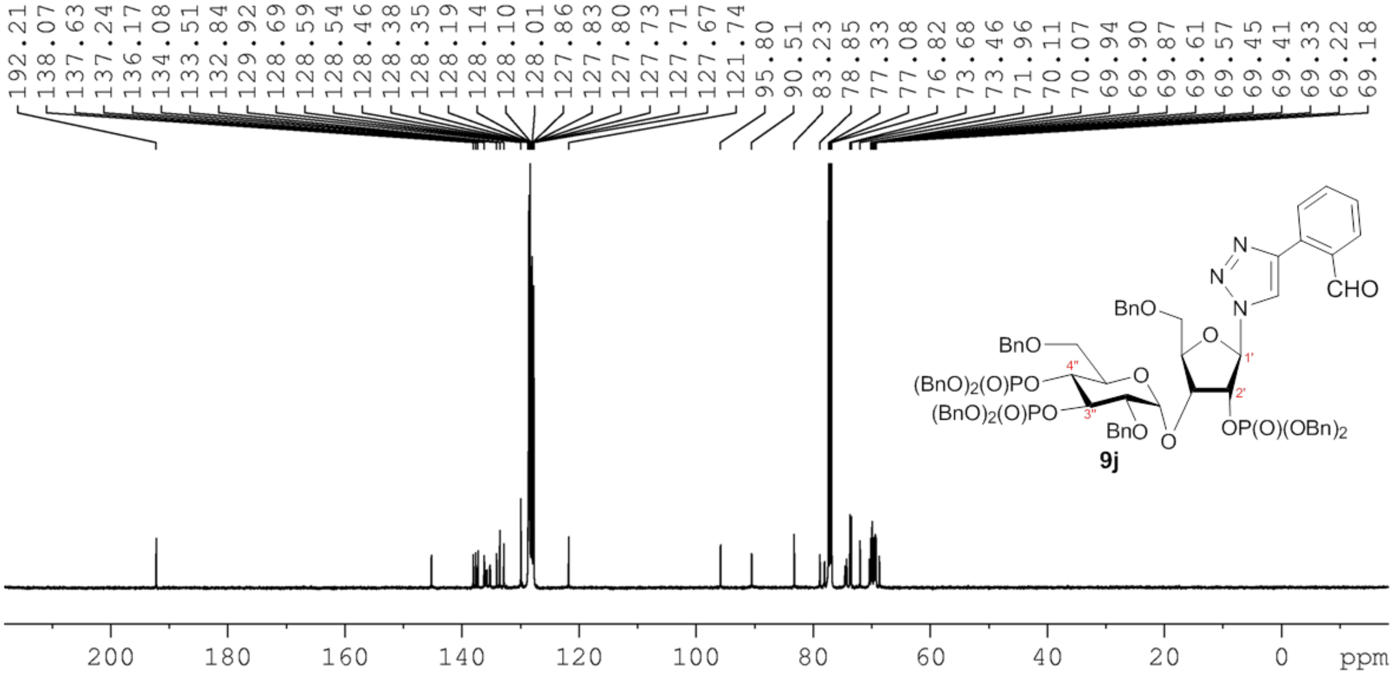

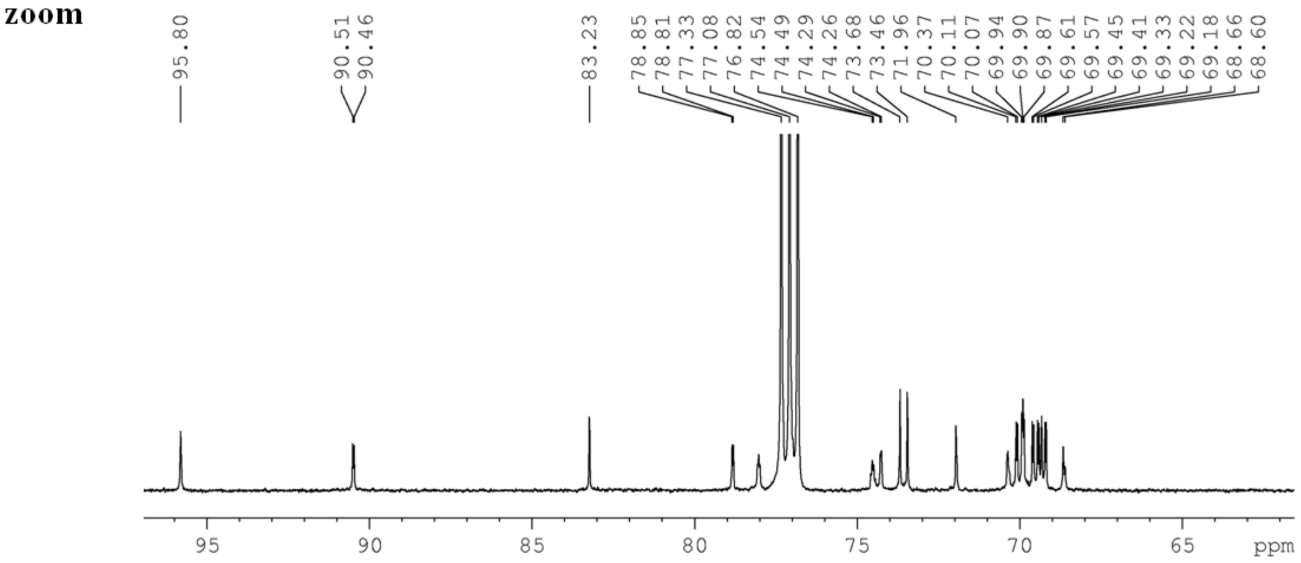

DEPT of 9j in $CDCl_3$

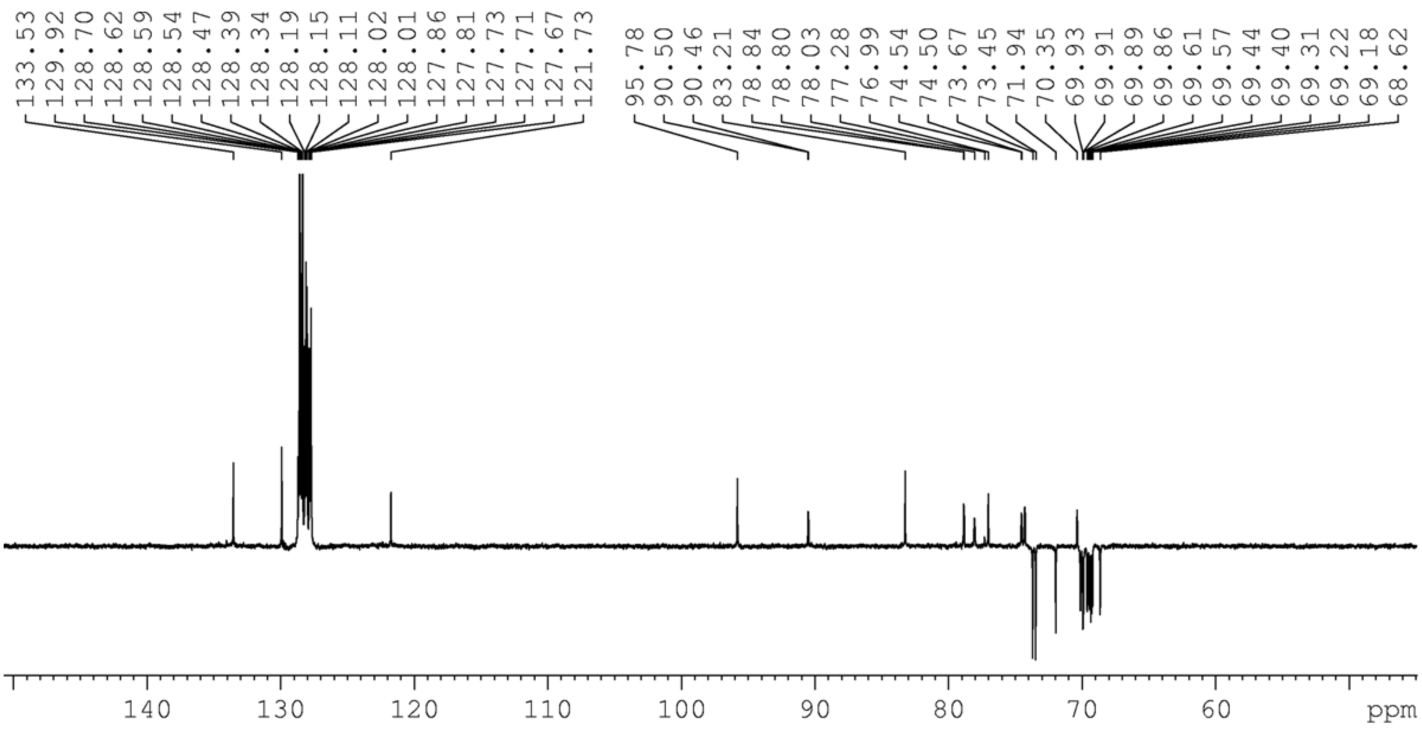

^{13}P NMR of 9j in $CDCl_3$

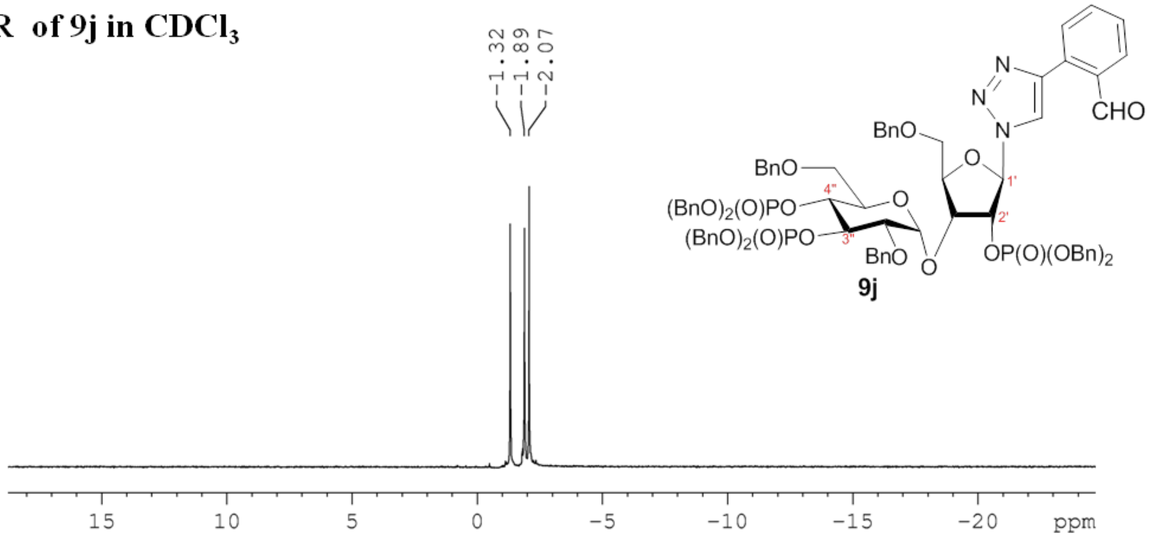

HMBC of 9j in $CDCl_3$

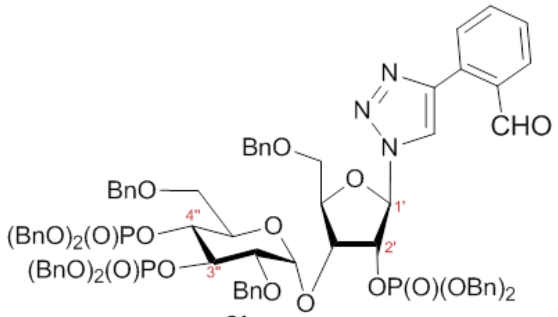

9j

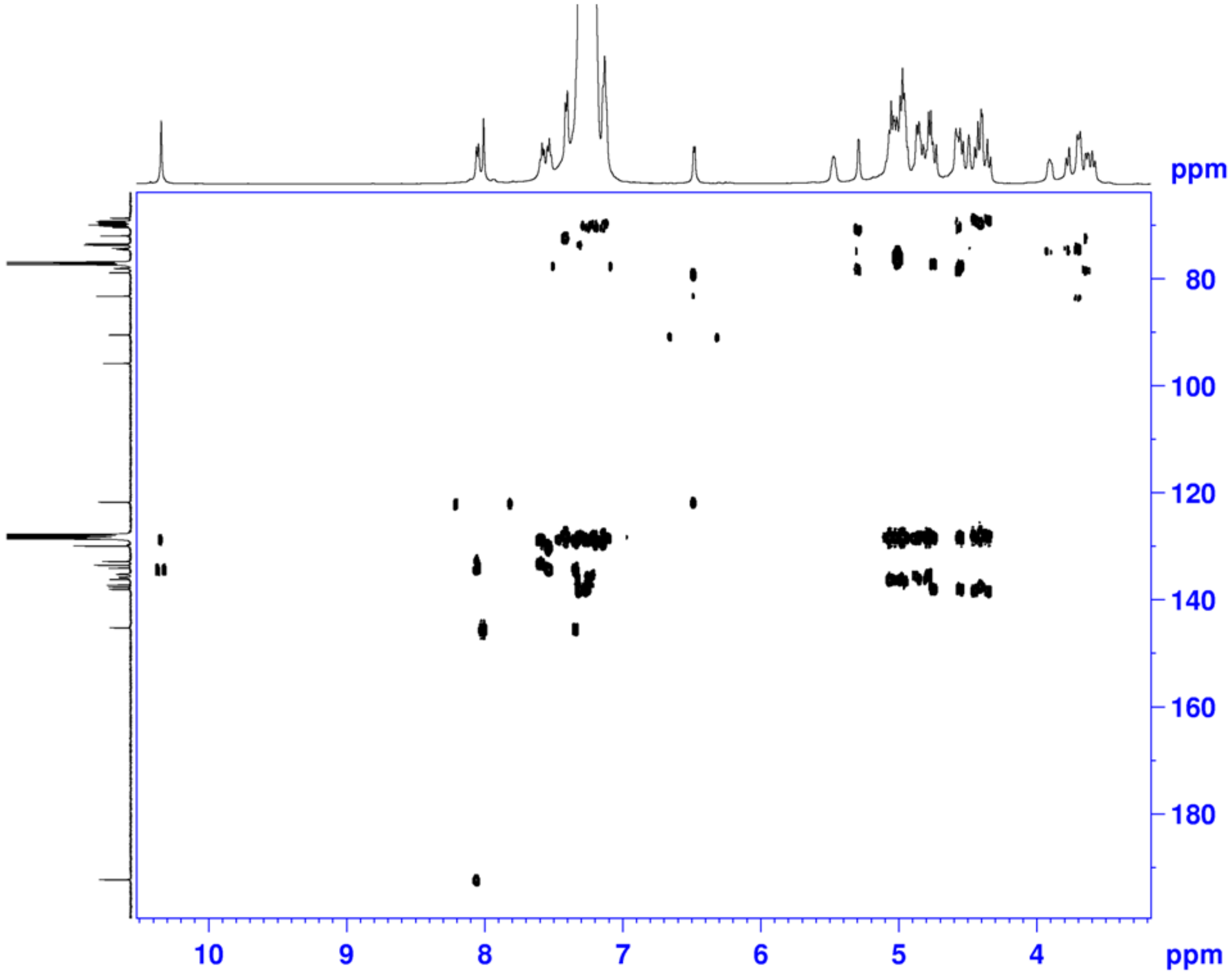

HMQC of 9j in $CDCl_3$

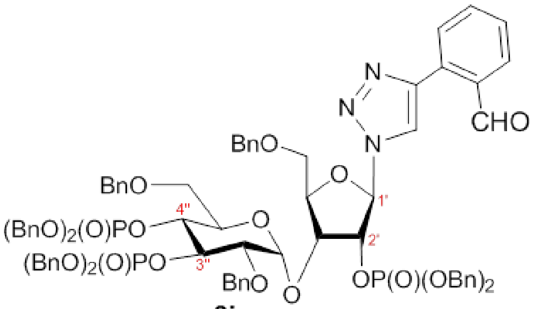

9j

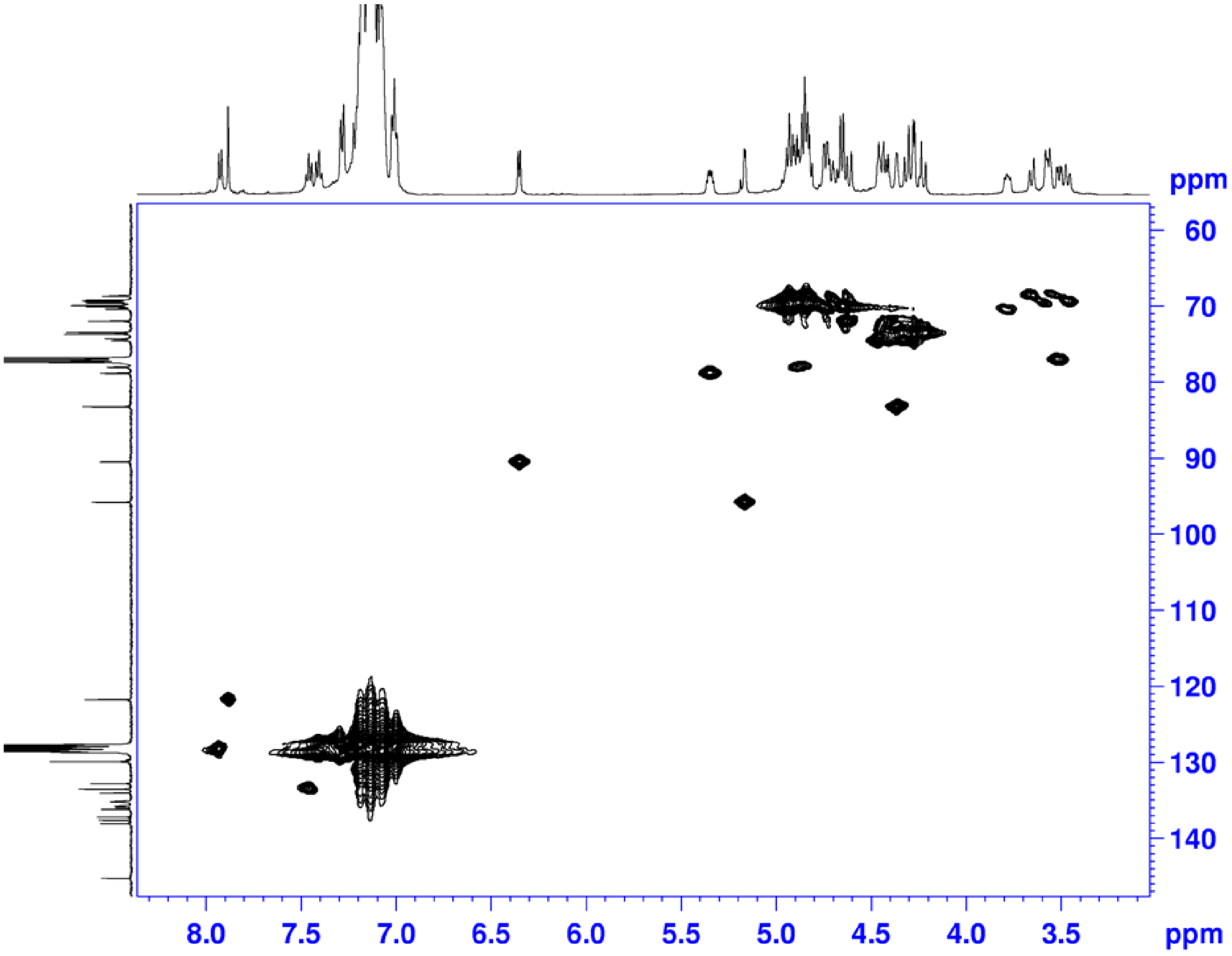

^1H NMR of 10j in D_2O

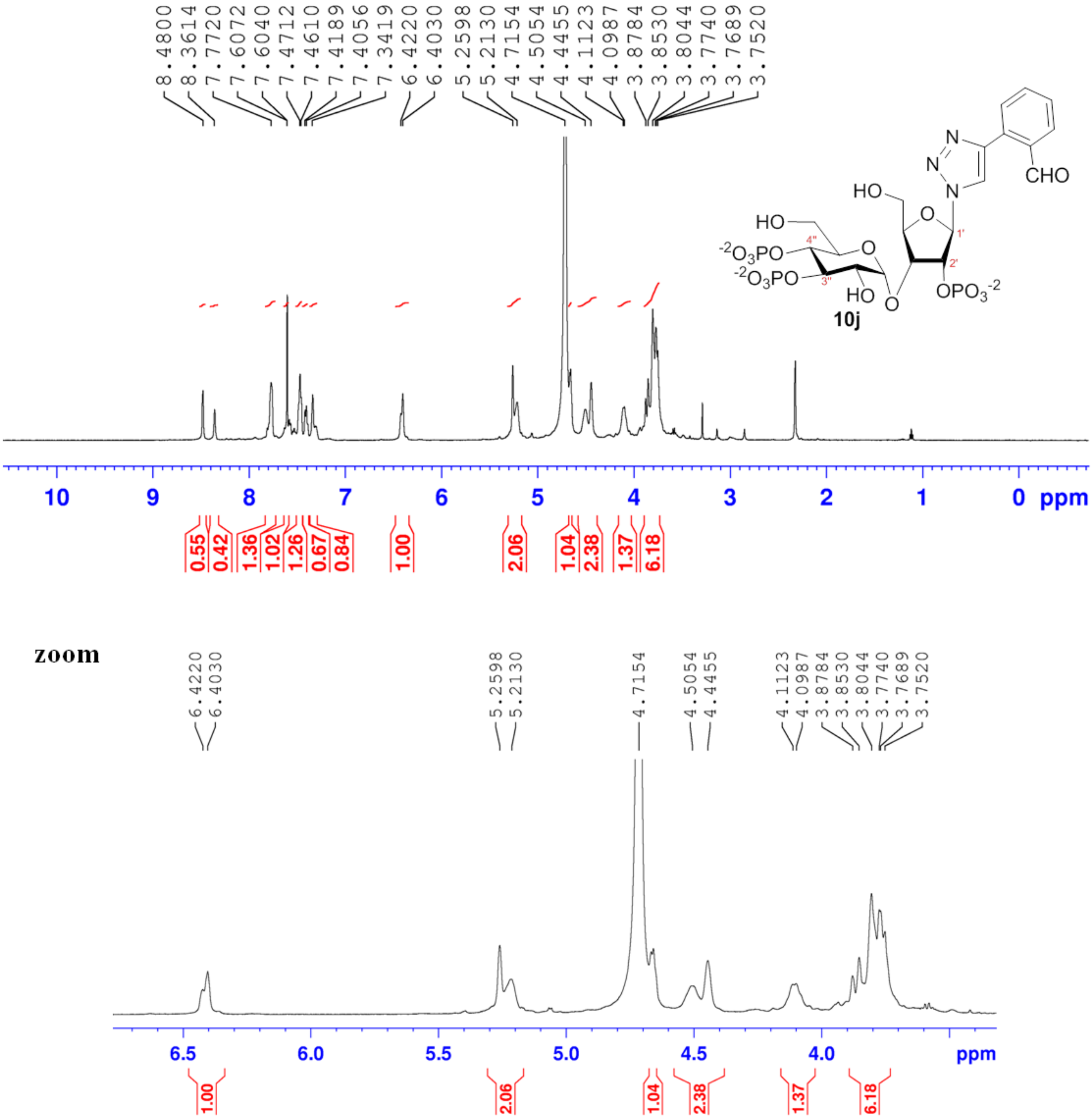

10j

COSY of 10j in D_2O

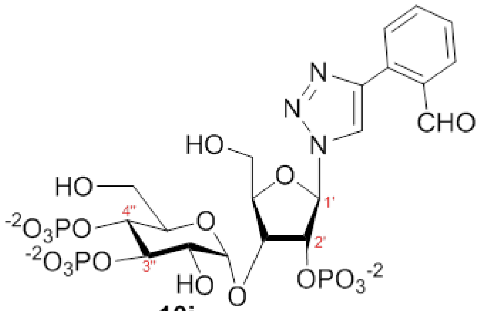

10j

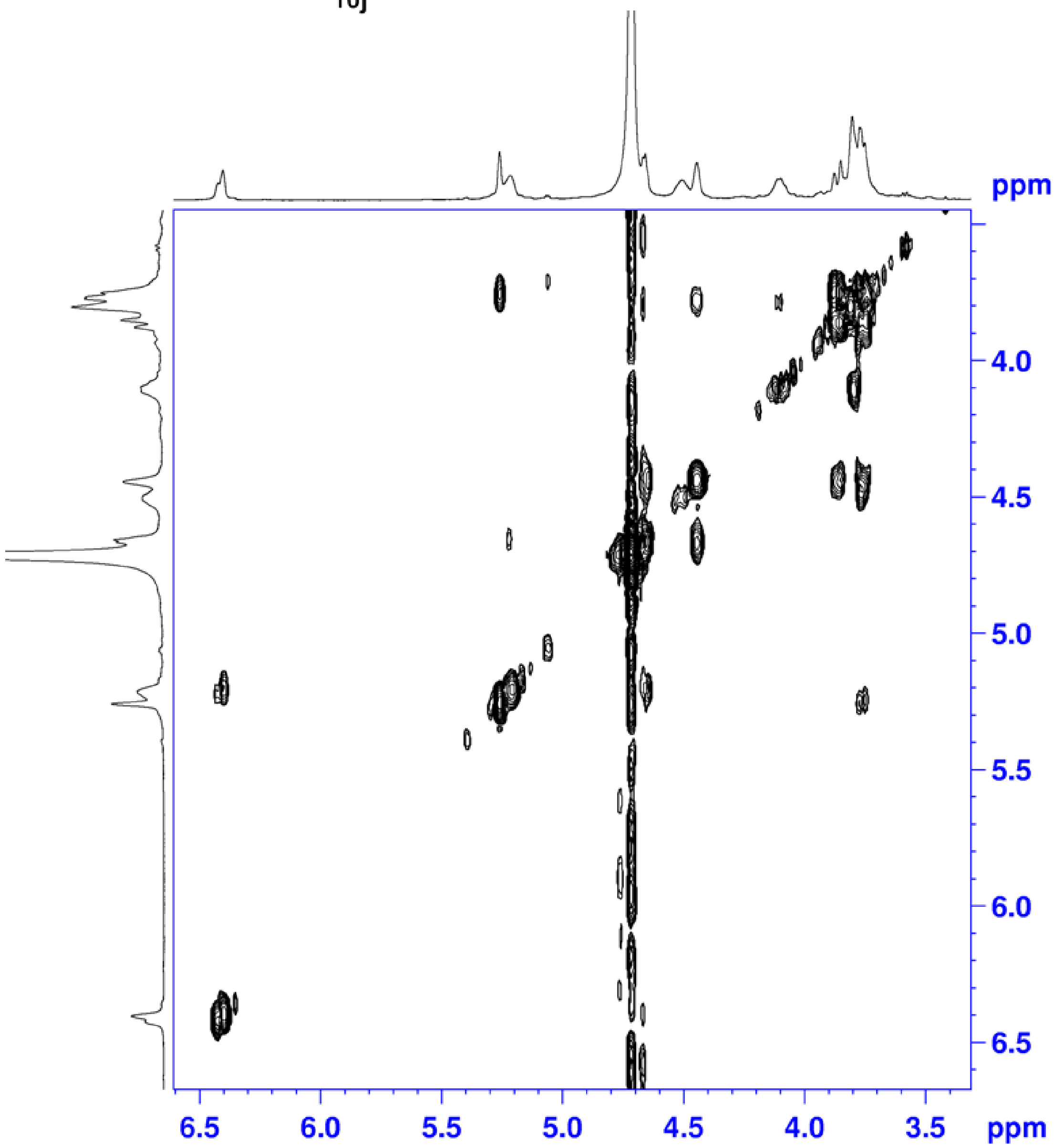

^{13}C NMR of 10j in D_2O

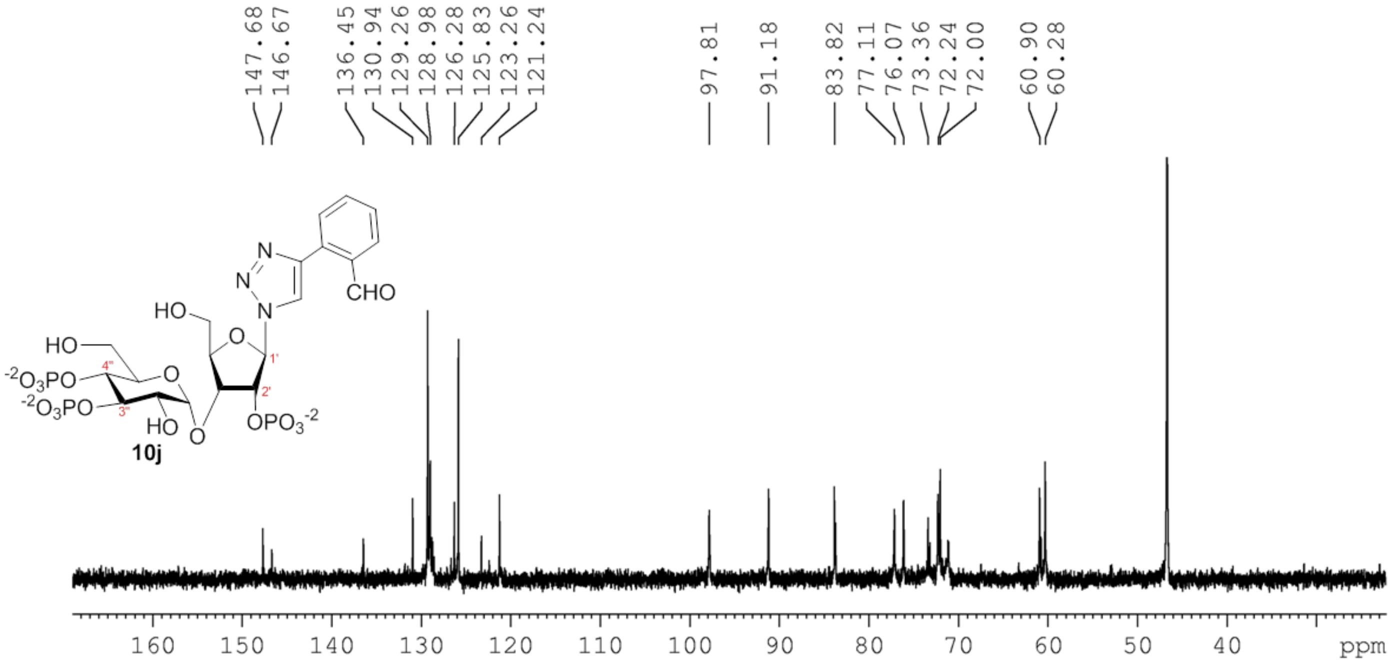

DEPT of 10j in D_2O

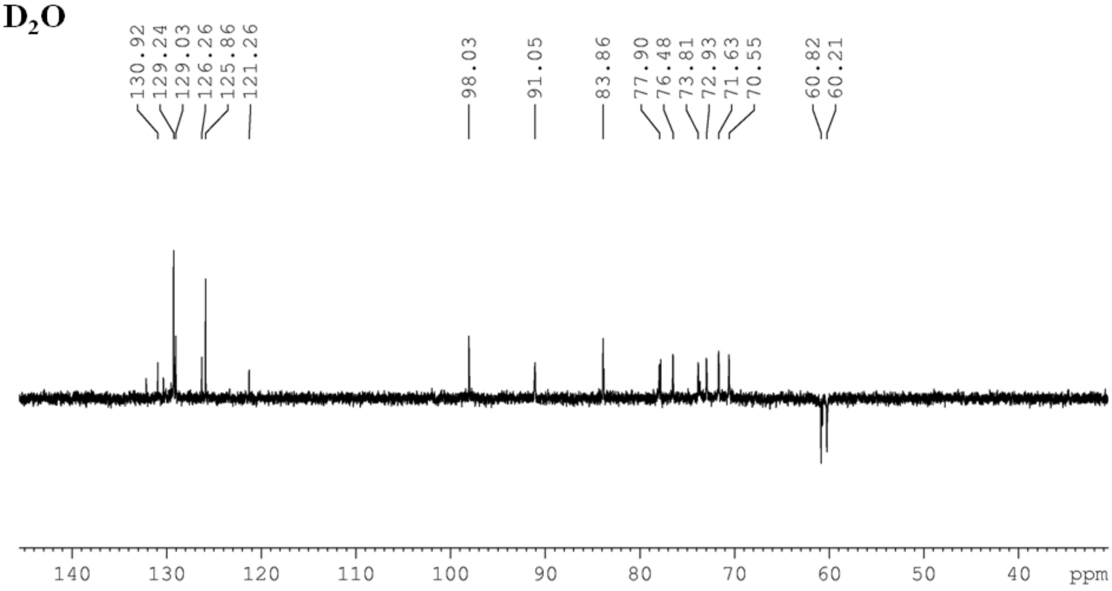

^{31}P NMR of 10j in D_2O

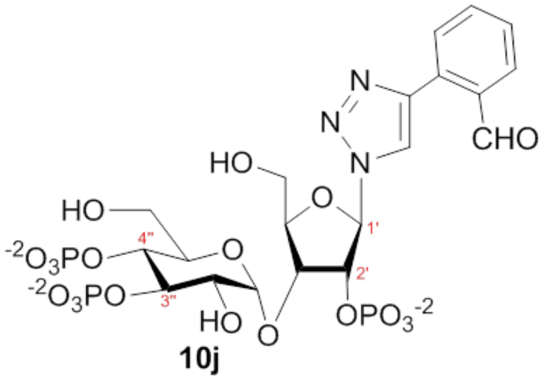

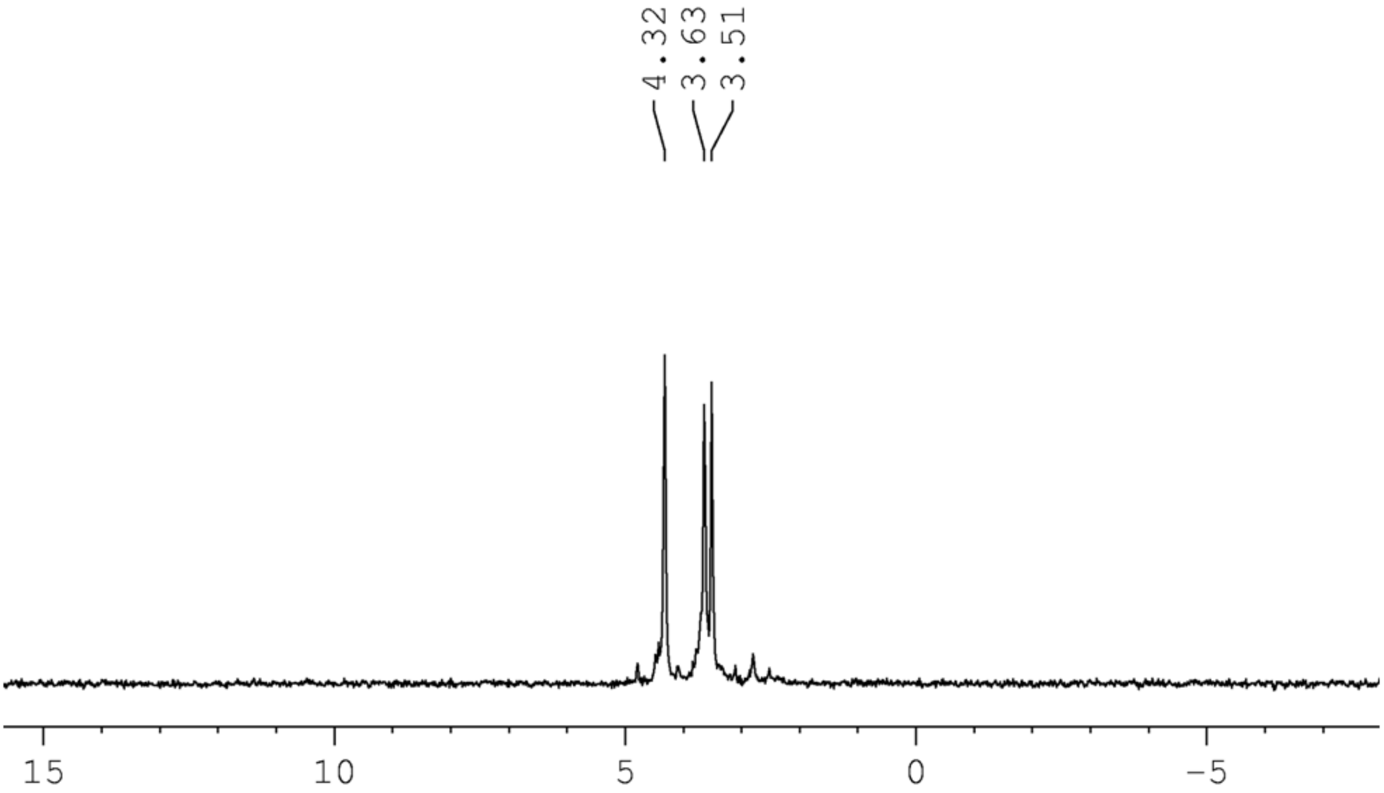

HMQC of 10j in D_2O

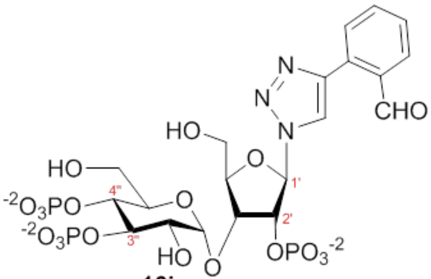

10j

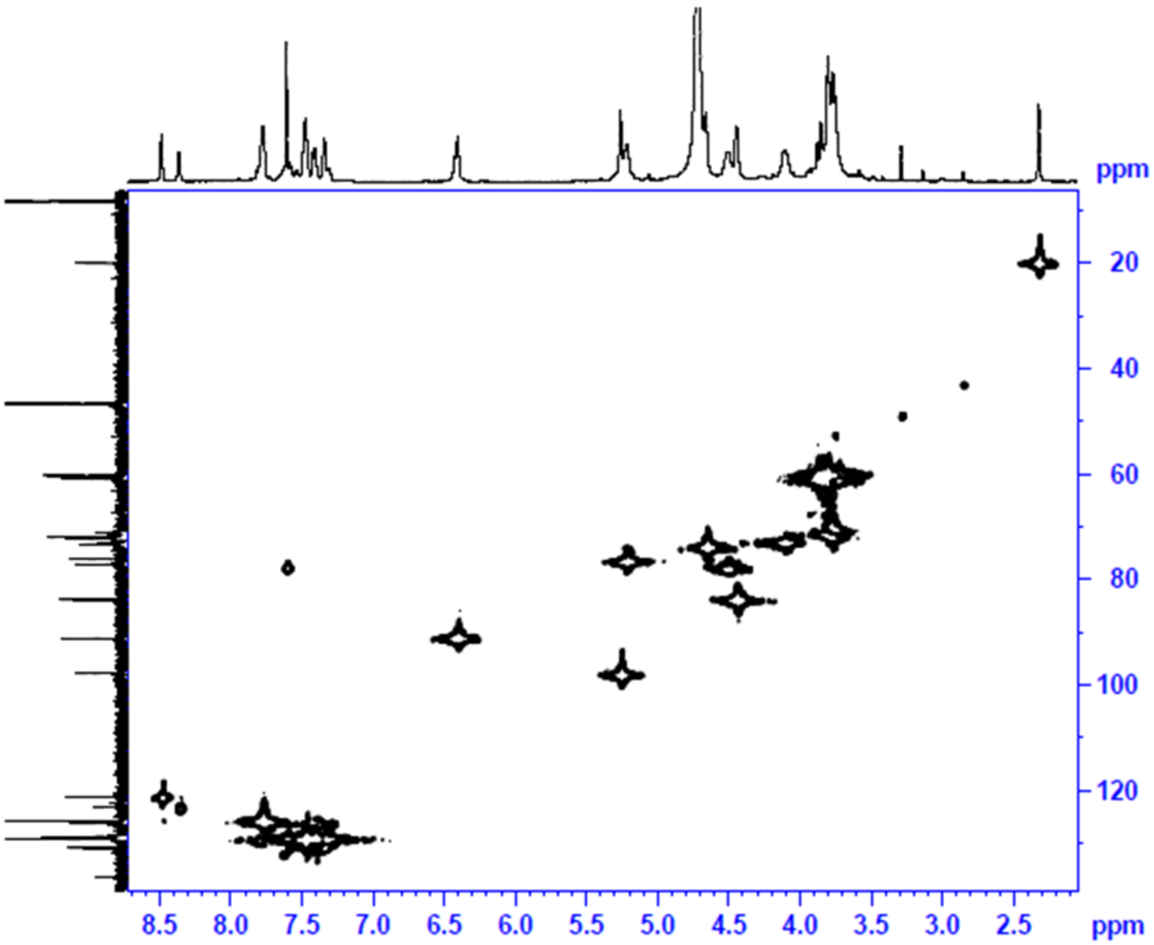

^{1}H NMR of 9k in $CDCl_3$

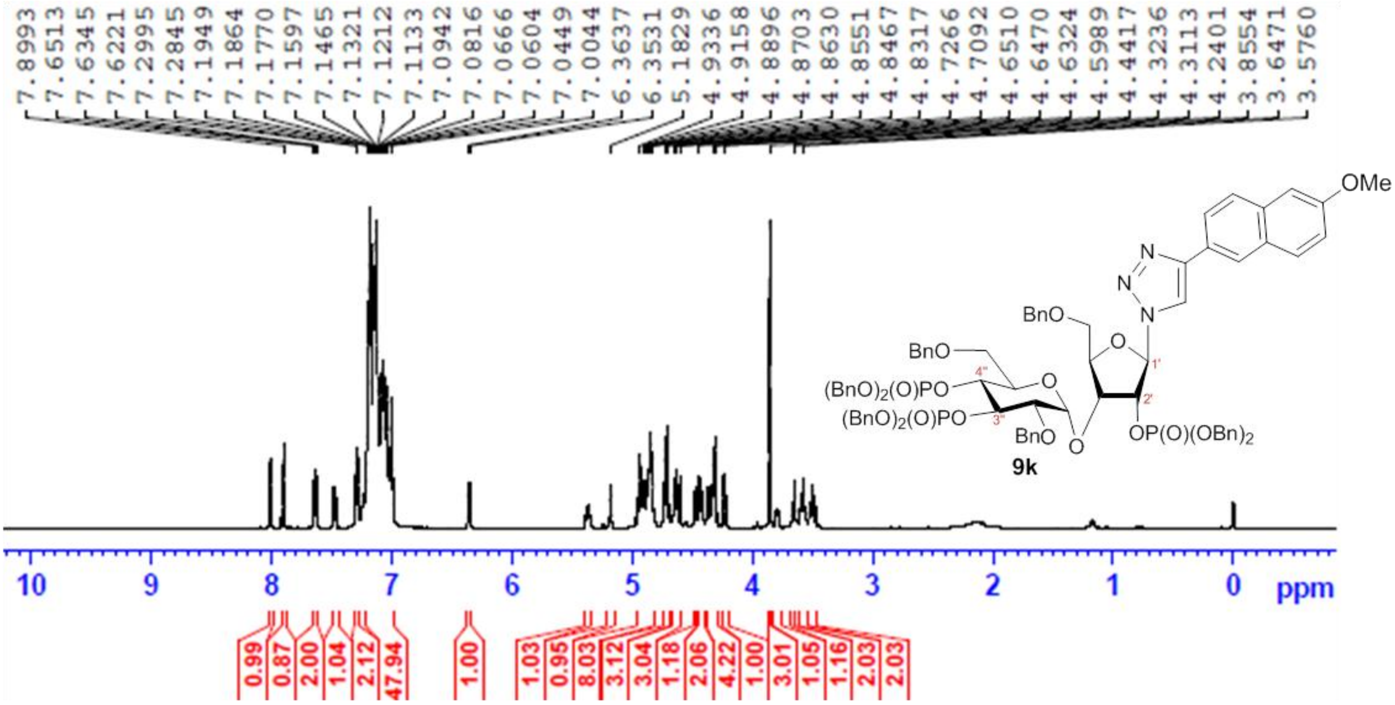

zoom

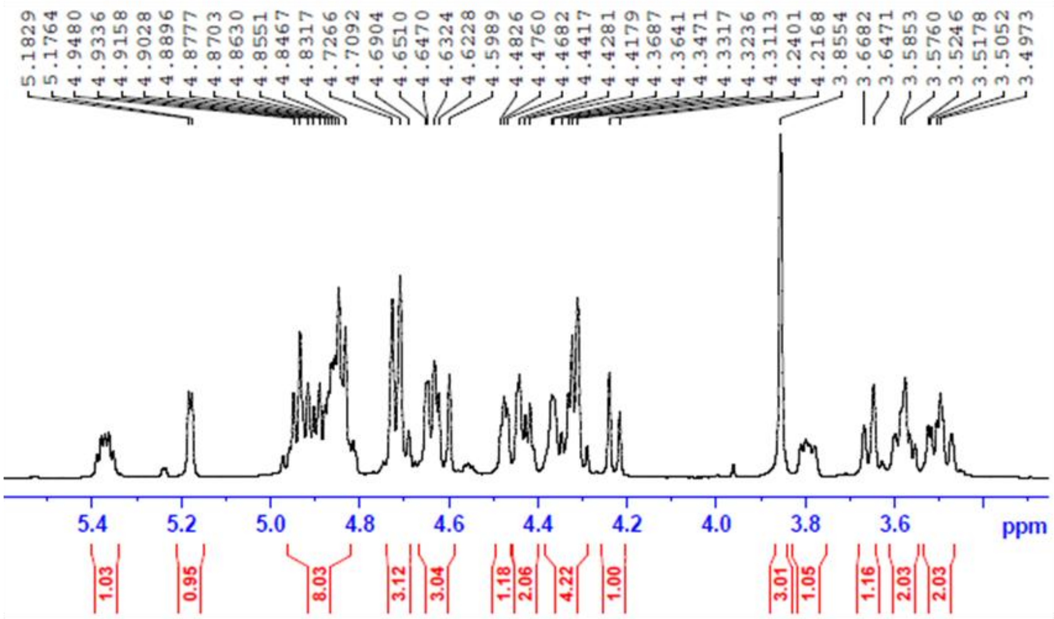

COSY of 9k in $CDCl_3$

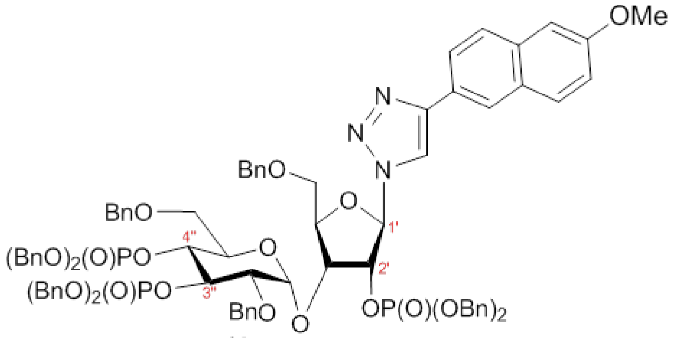

9k

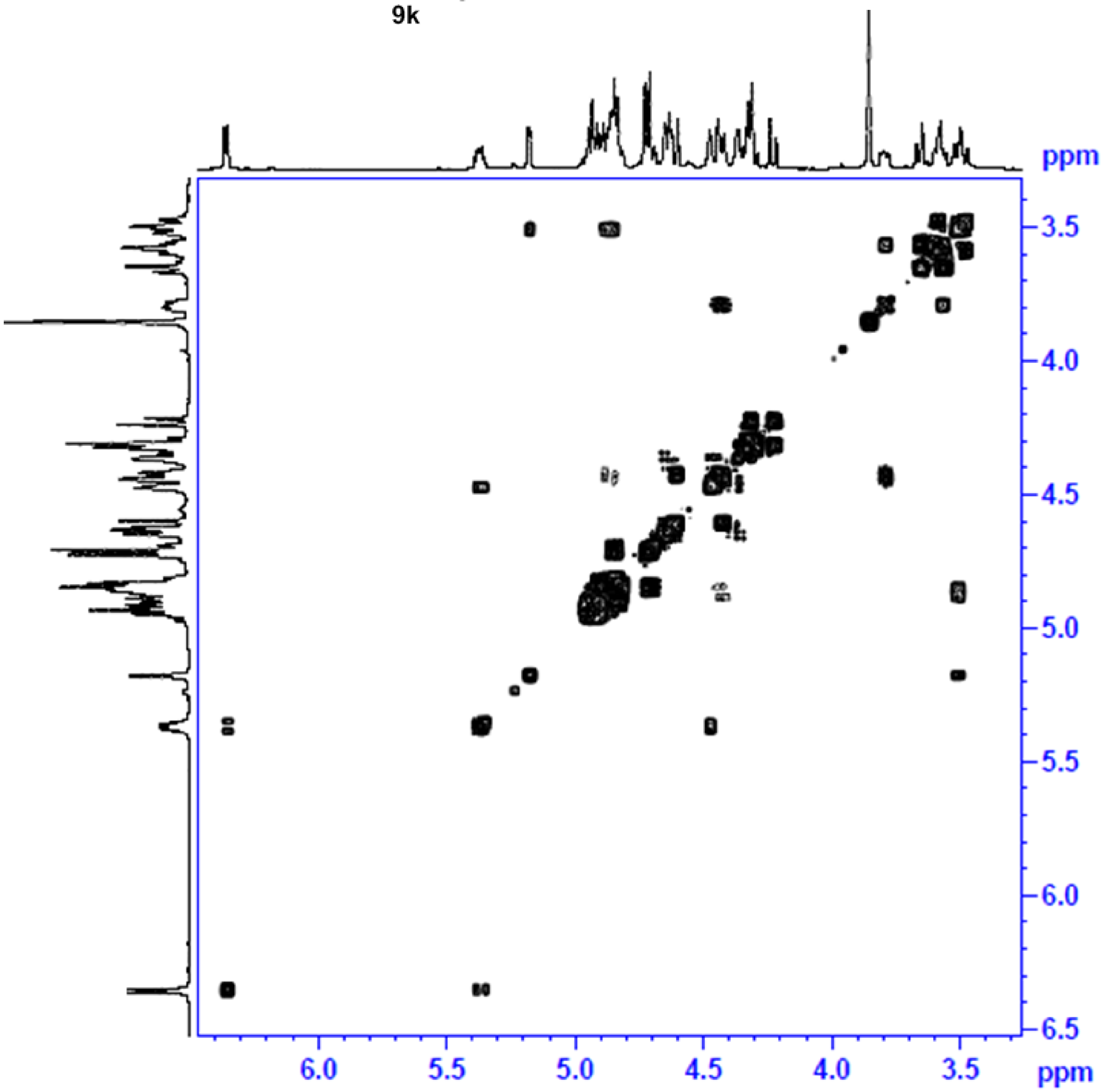

^{13}C NMR of 9k in CDCl$_3$

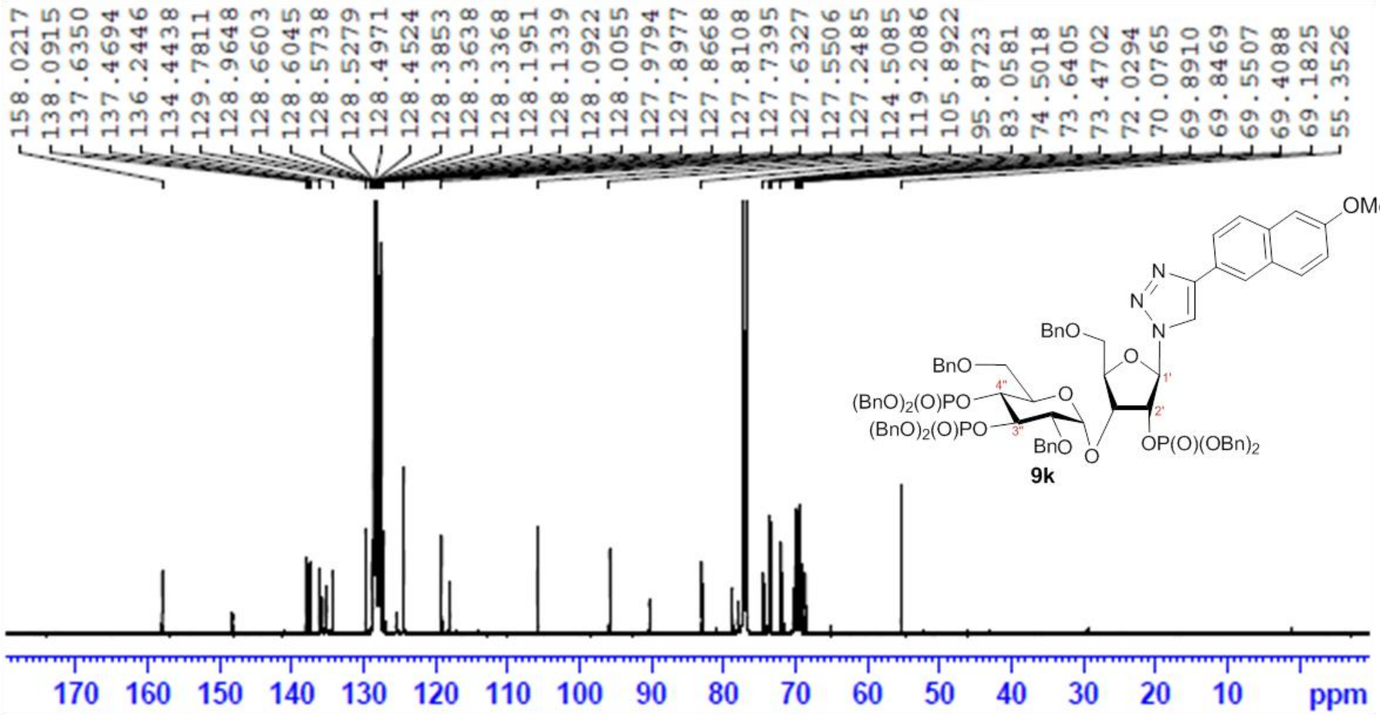

zoom

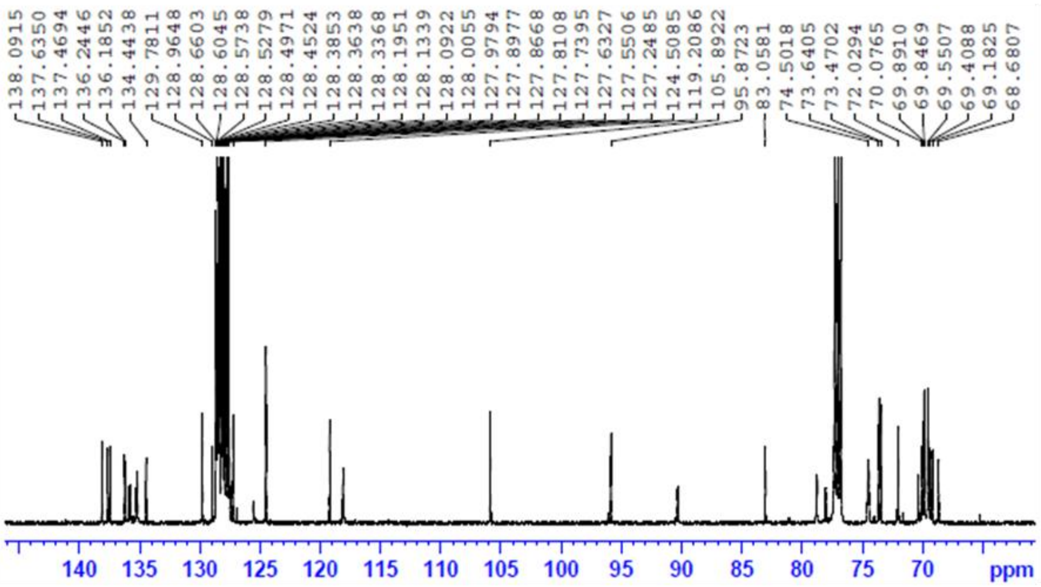

DEPT of 9k in $CDCl_3$

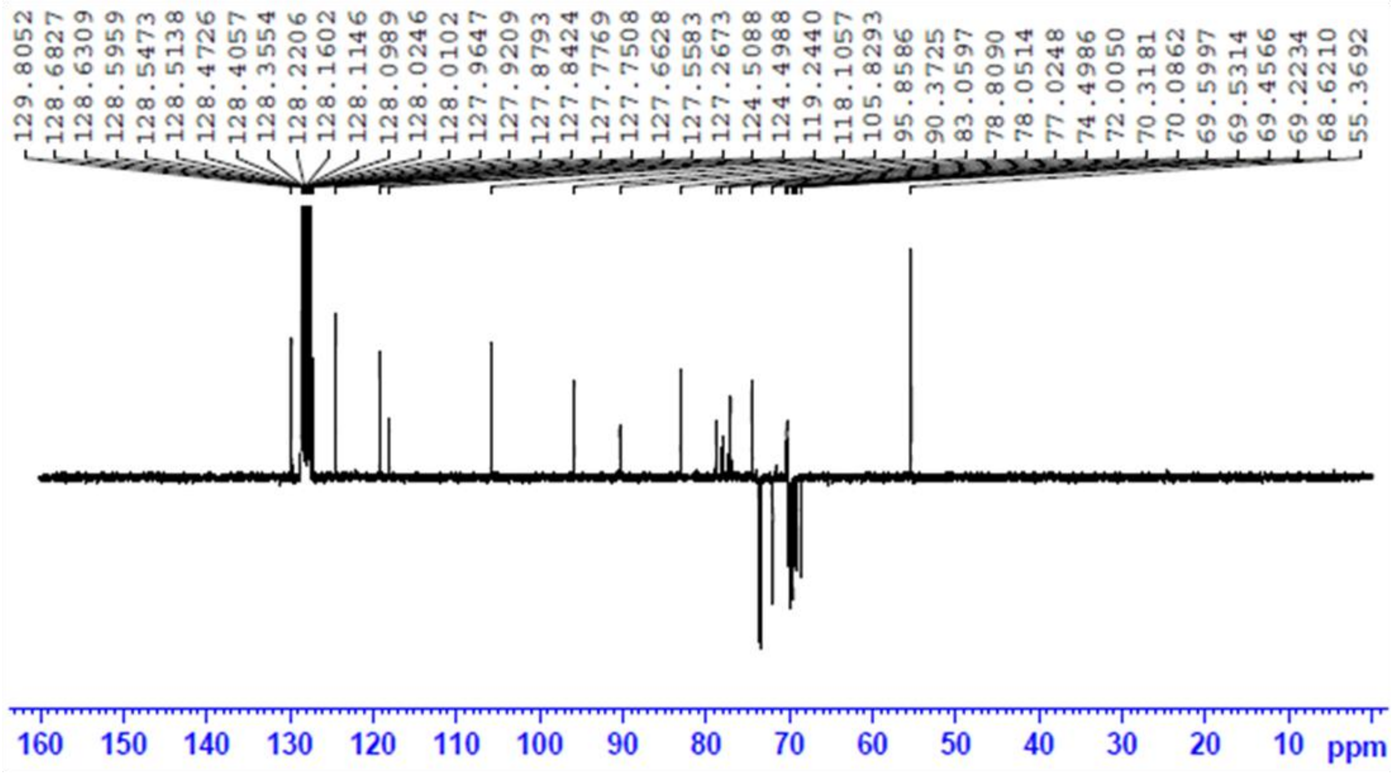

^{31}P NMR of 9k in $CDCl_3$

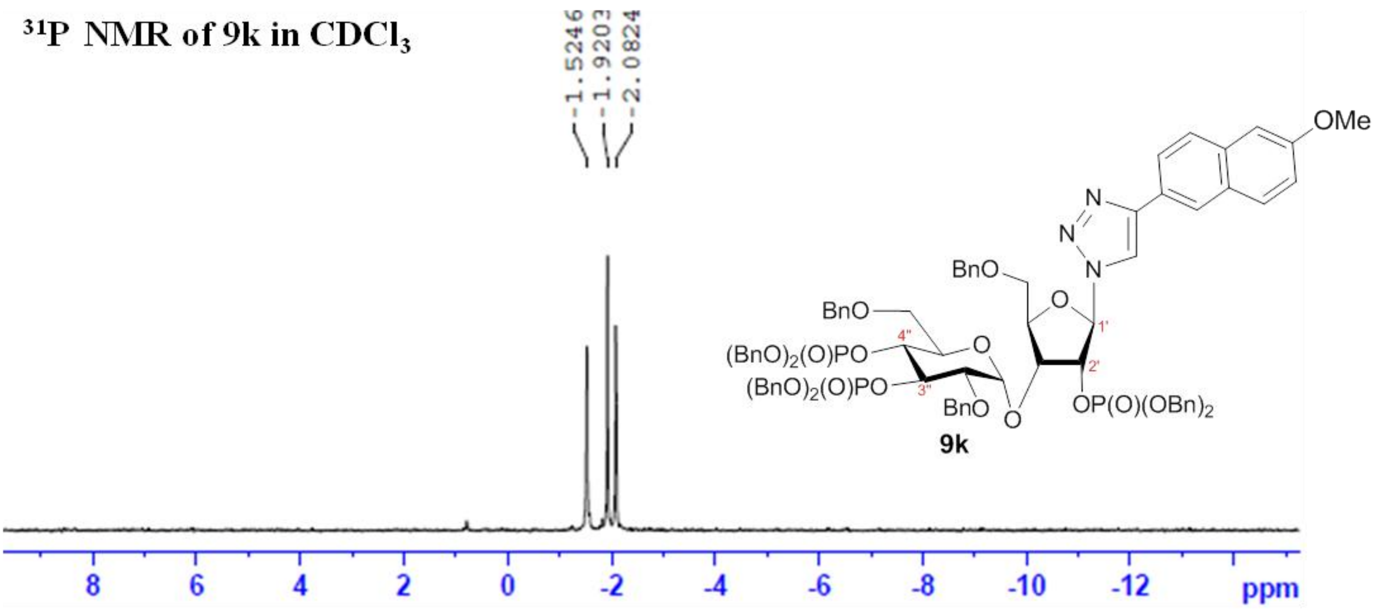

HMBC of 9k in $CDCl_3$

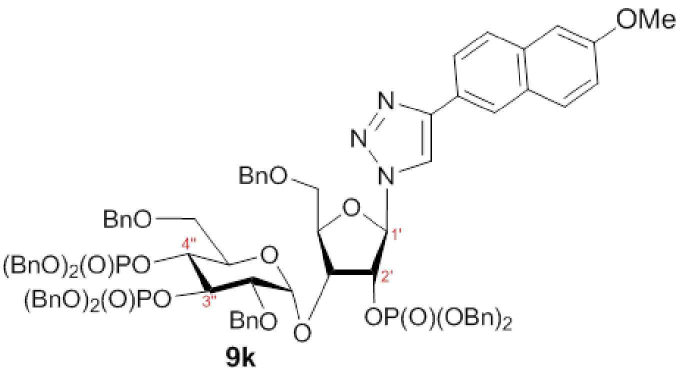

9k

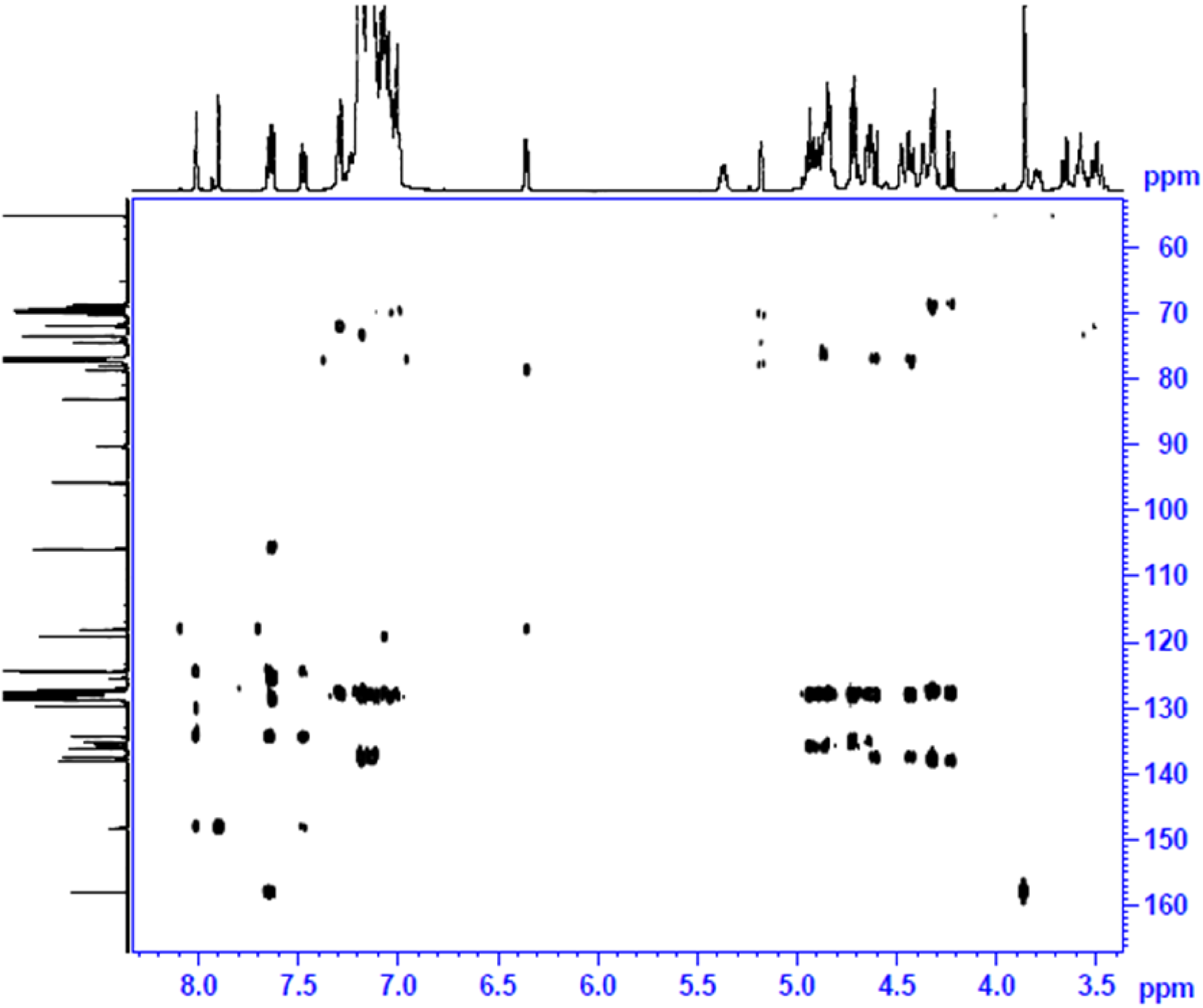

HMQC of 9k in $CDCl_3$

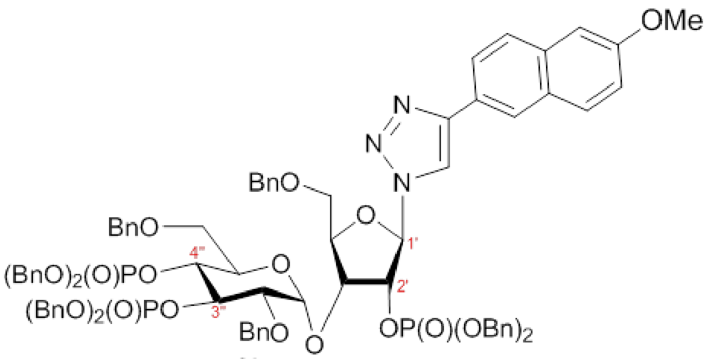

9k

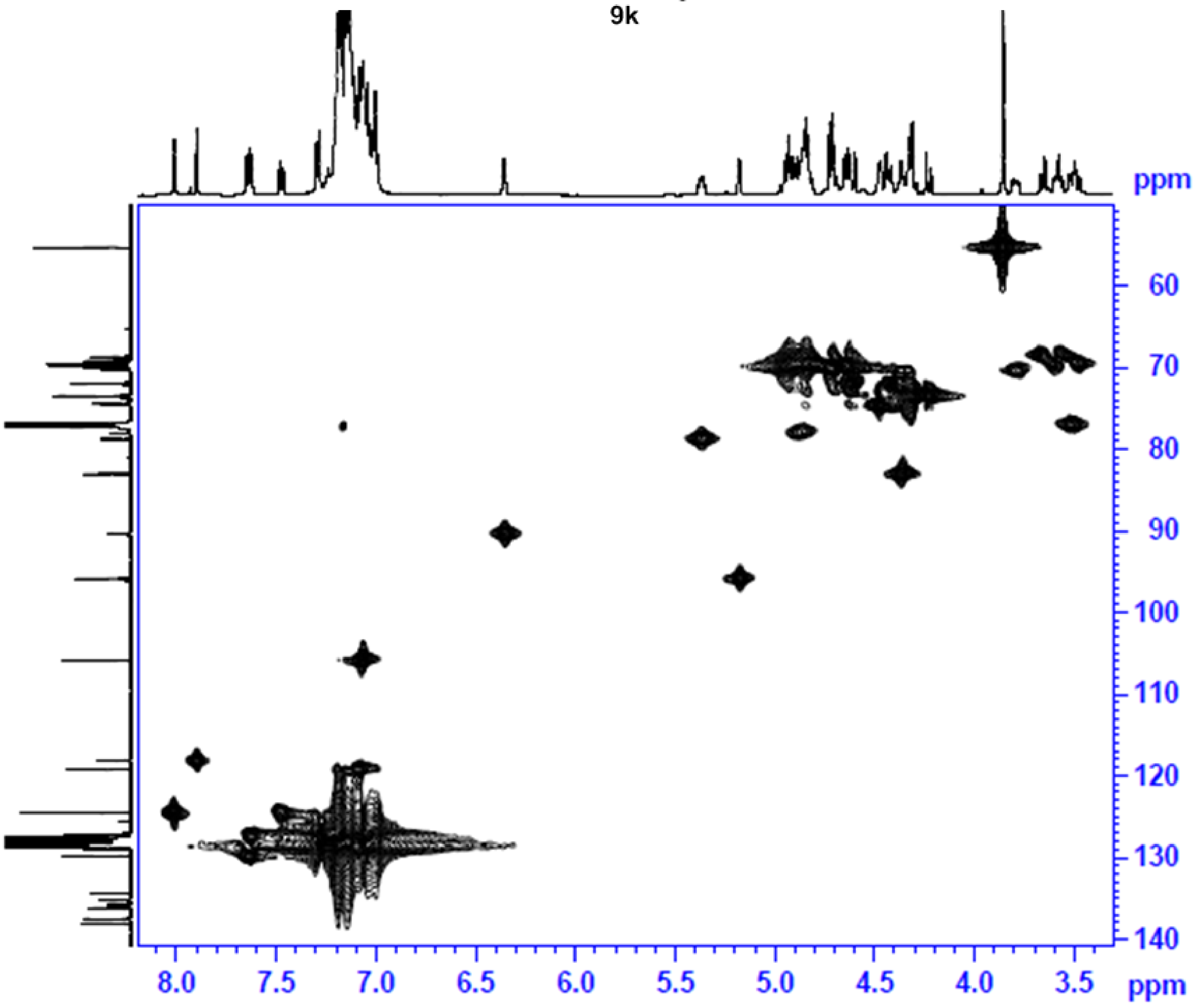

^{1}H NMR of 10k in MeOD

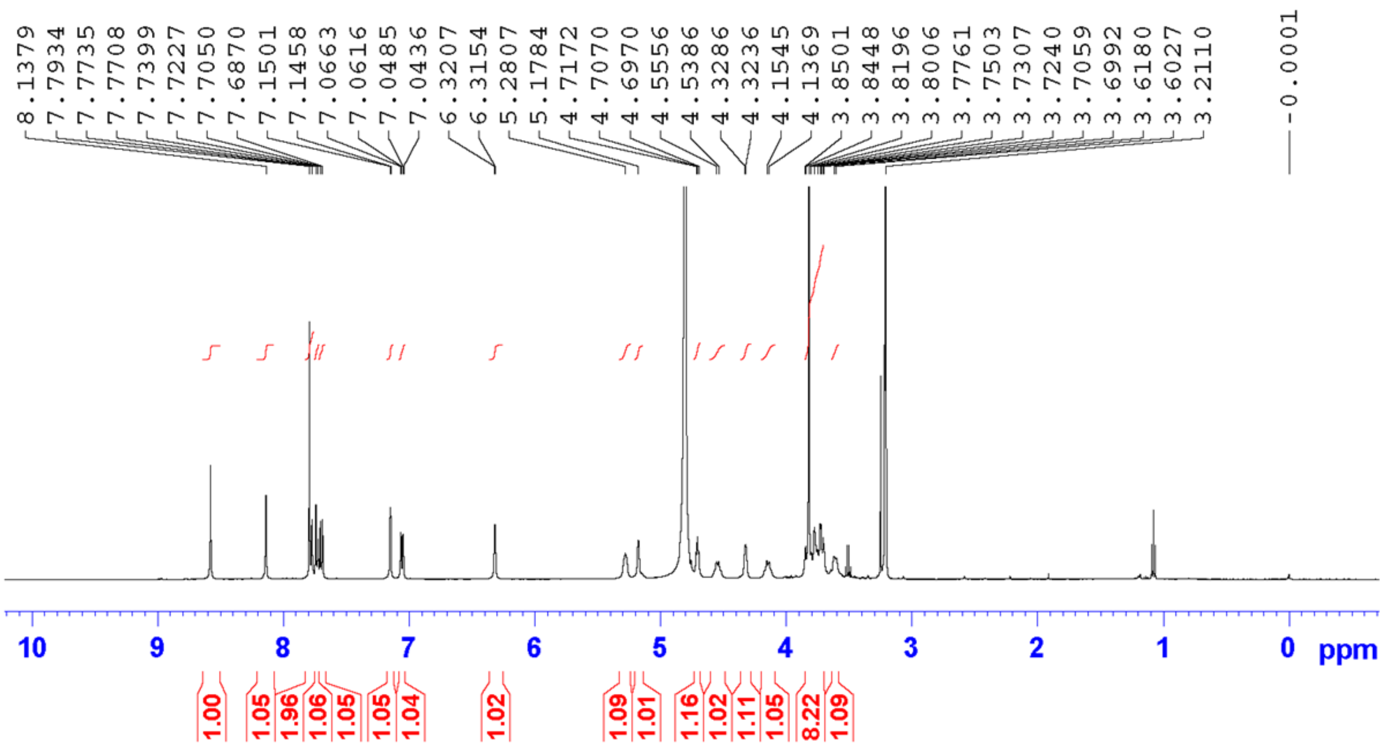

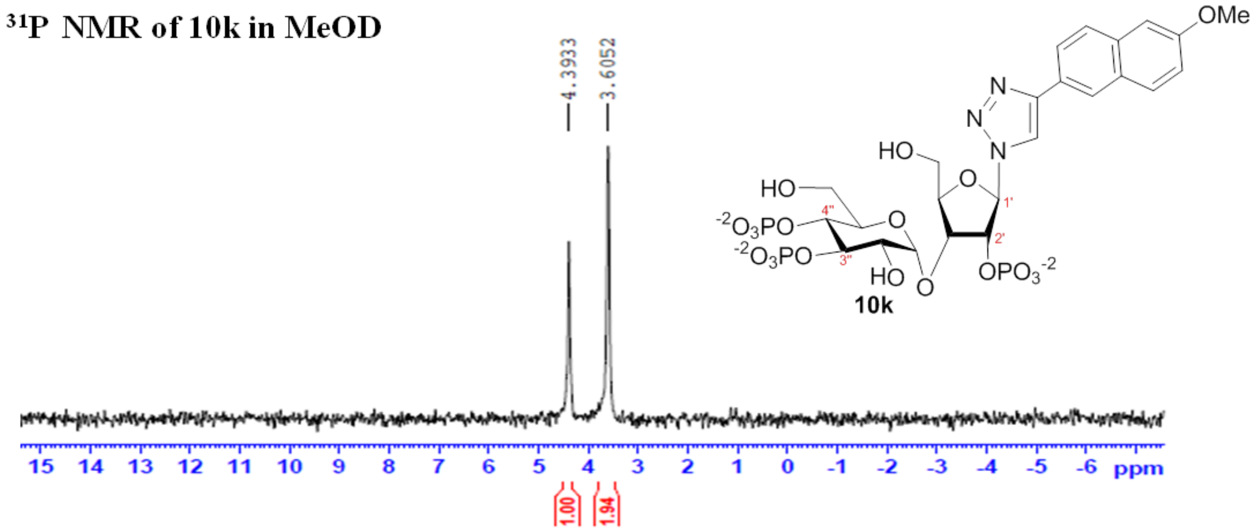

COSY of 10k in MeOD

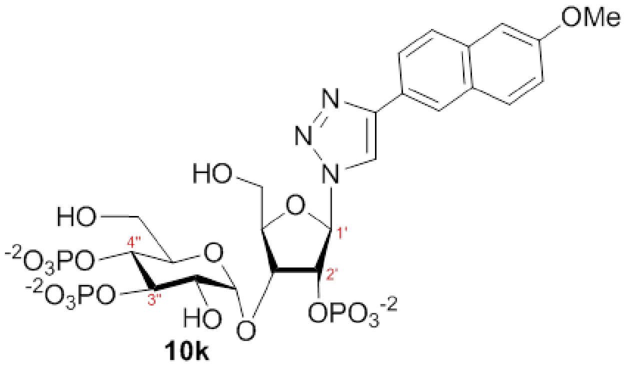

10k

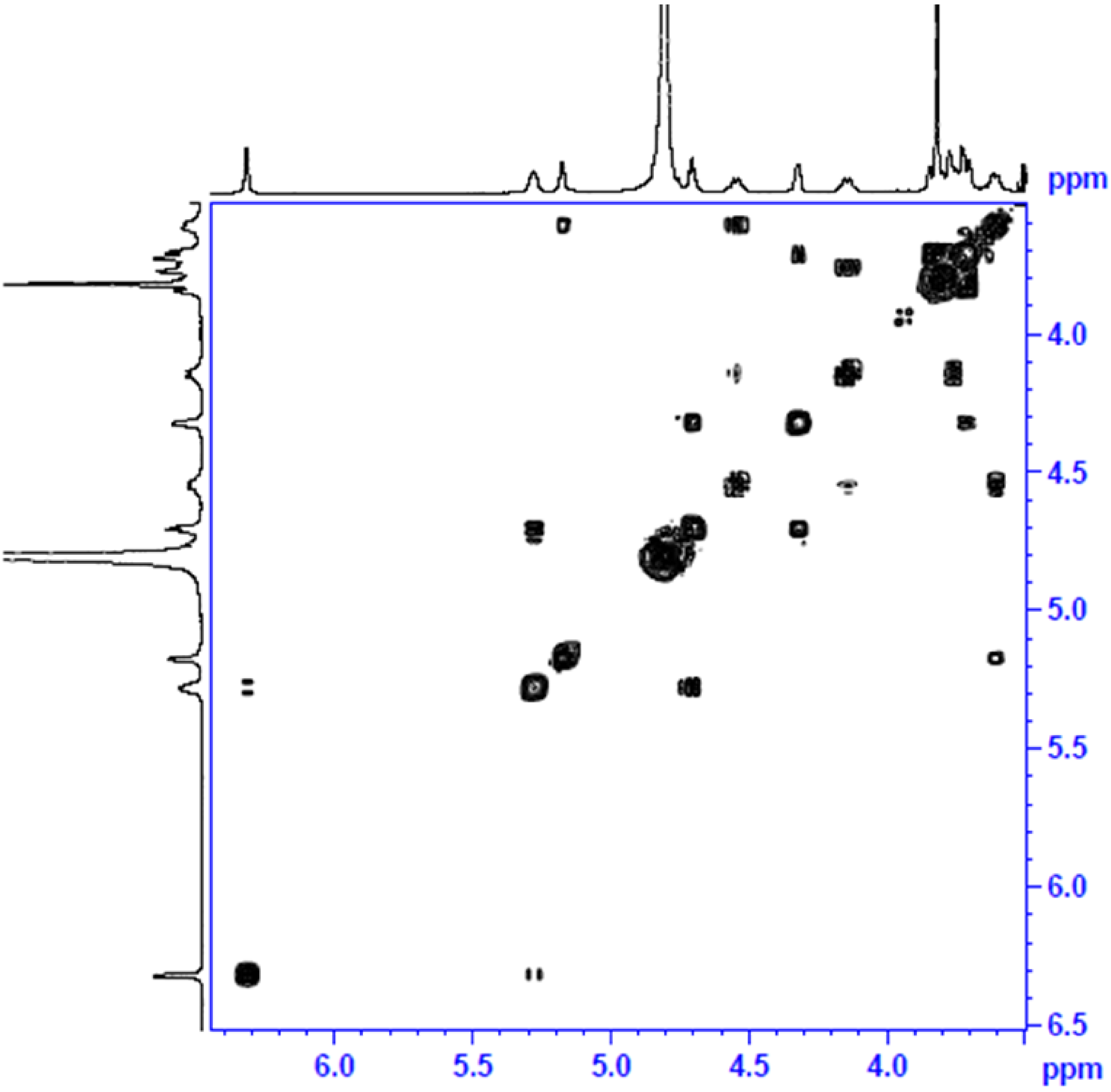

^{13}C NMR of 10k in MeOD

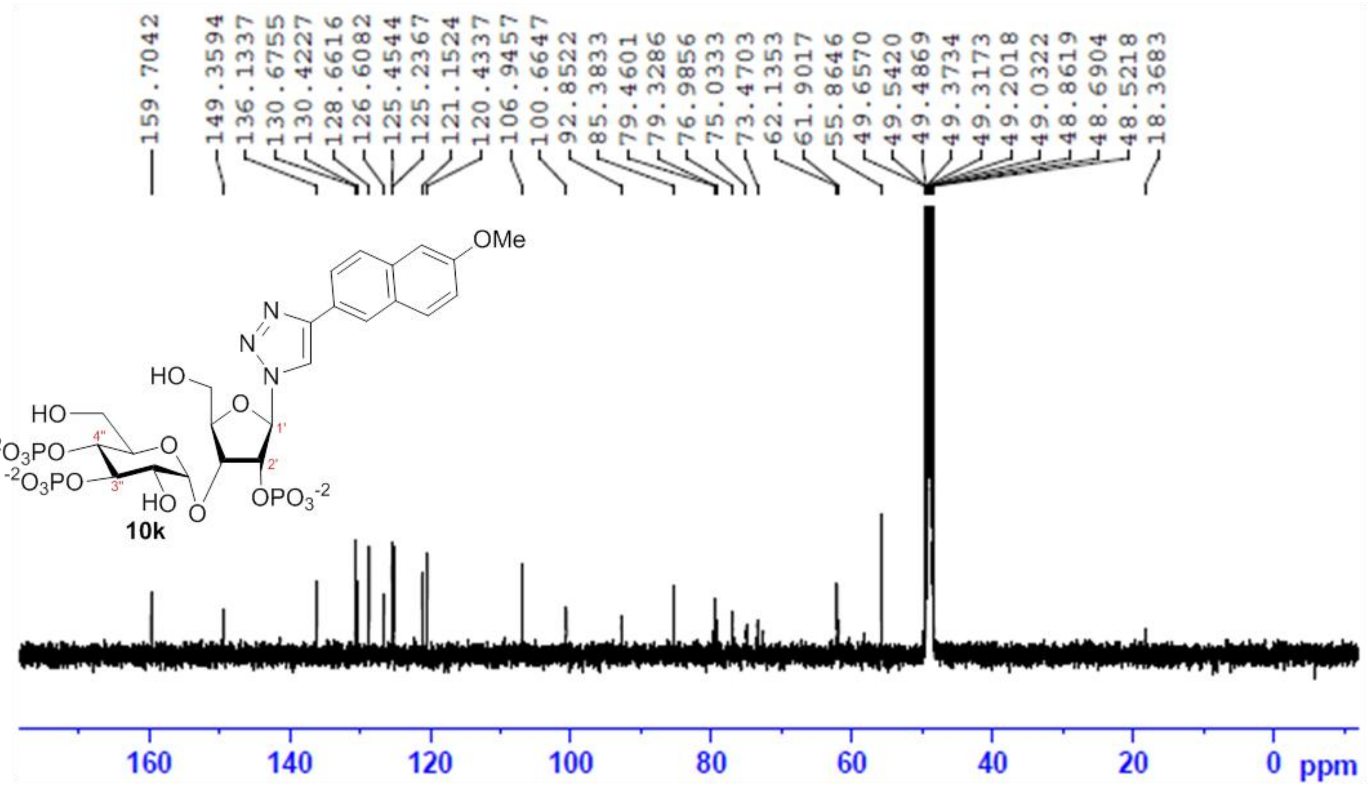

DEPT of 10k in MeOD

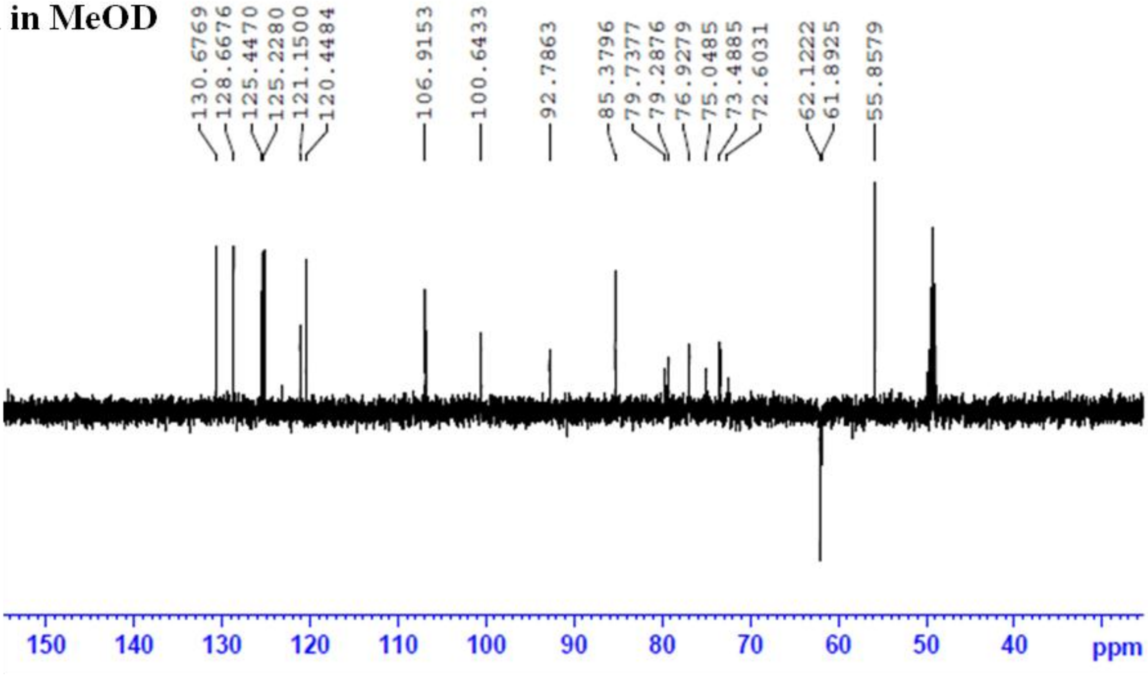

HMBC of 10k in MeOD

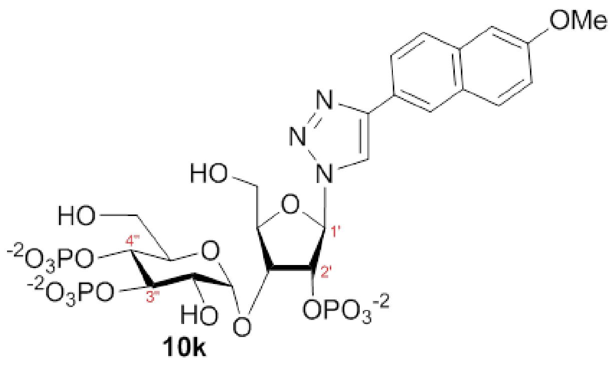

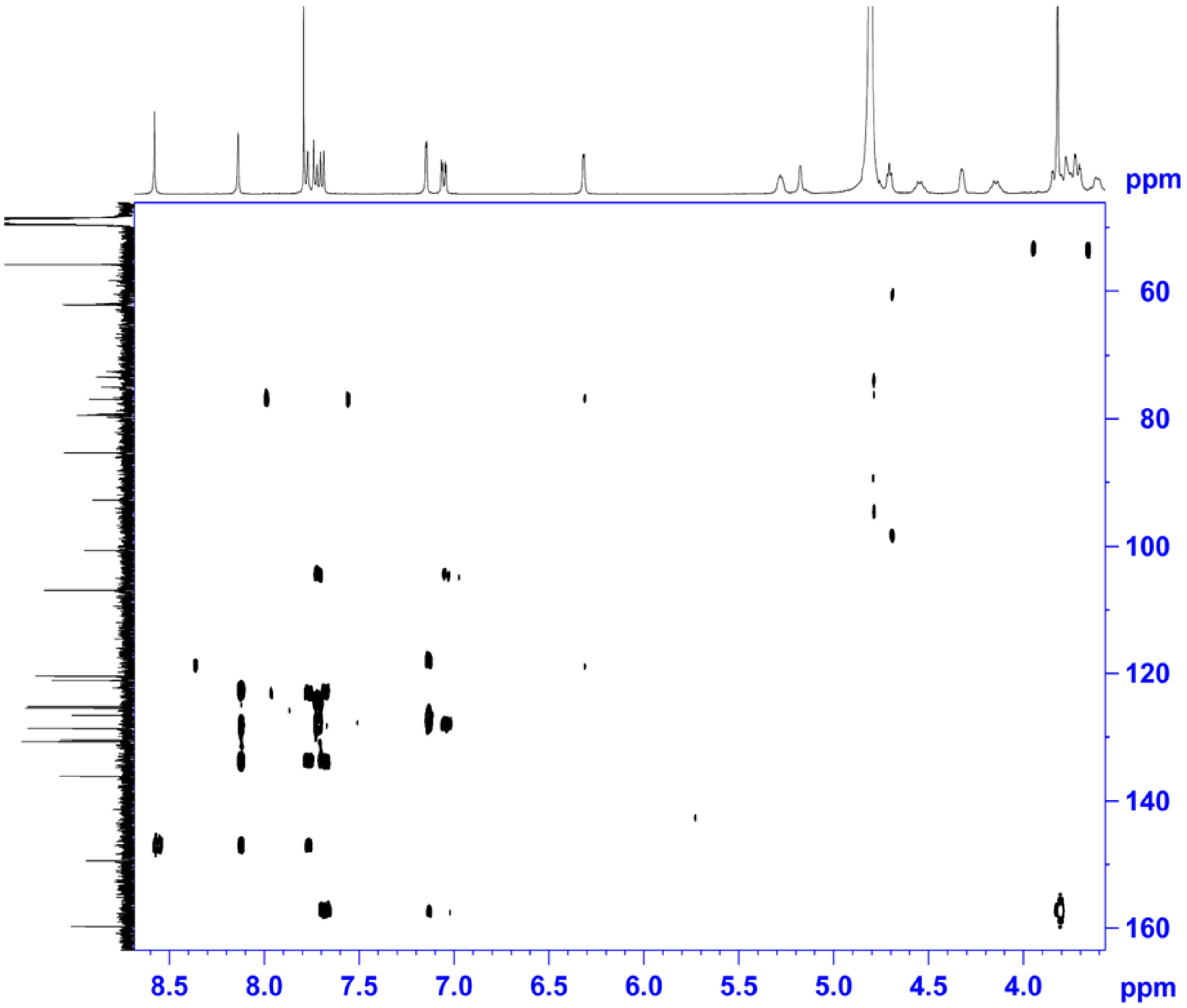

HMQC of 10k in MeOD

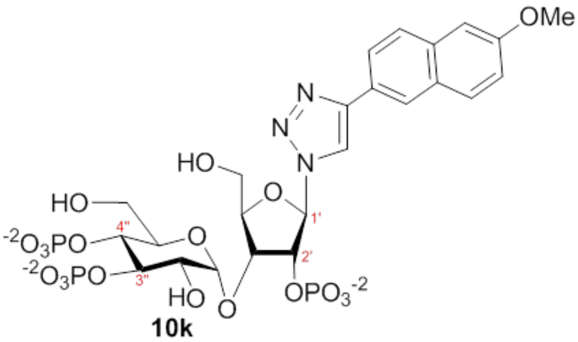

10k

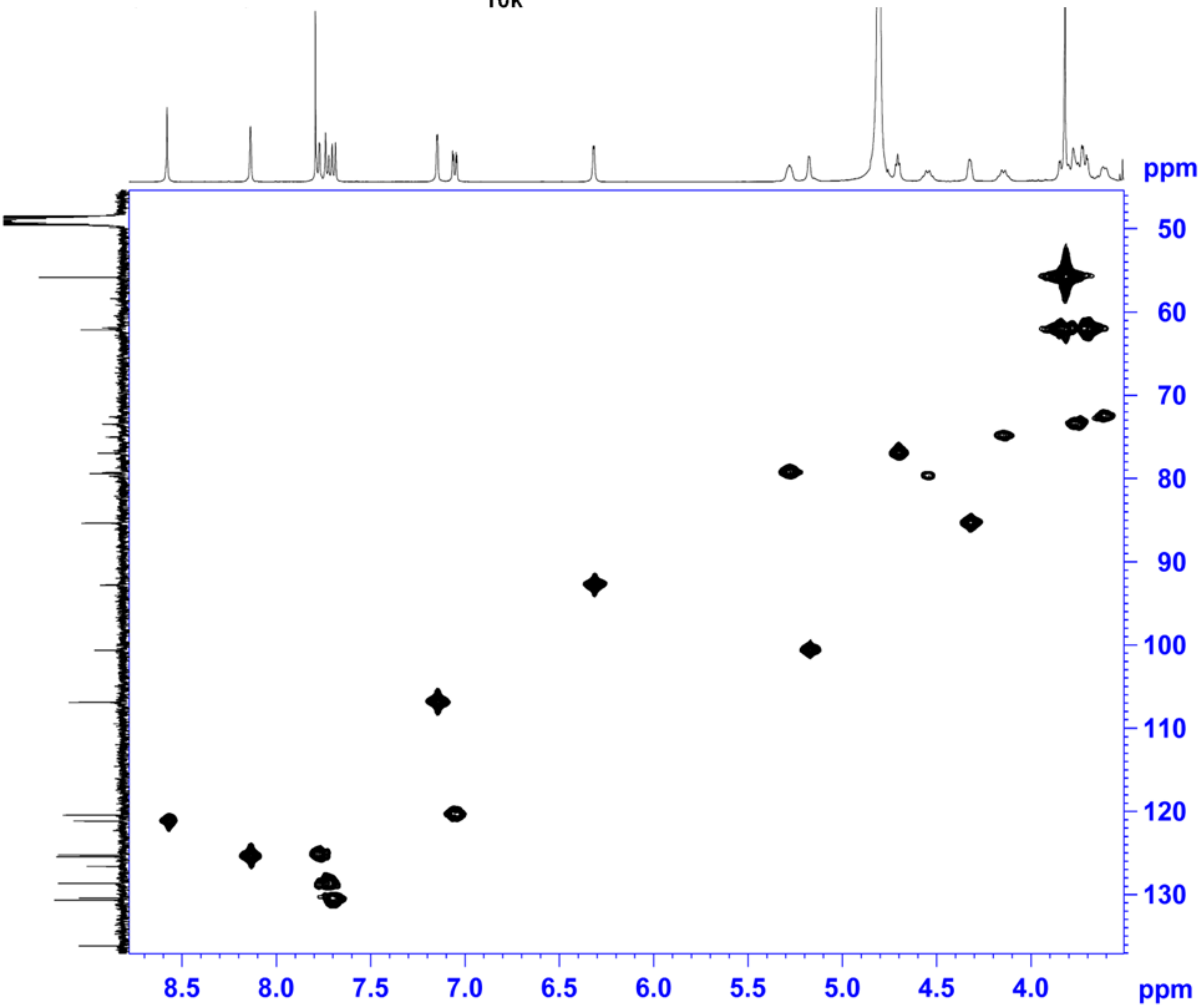

References

(1) O. Trott and A. J. Olson, *J. Comput. Chem*. 2010, **31**, 455.

(2) I. Bosanac, J. -R. Alattia, T. K. Mal, J. Chan, S. Talarico, F. K. Tong, K. I. Tong, F. Yoshikawa, T. Furuichi, M. Iwai, T. Michikawa, K. Mikoshiba and M. Ikura, *Nature,* 2002, **420**, 696.

(3) H. J. Rosenberg, A. M. Riley, A. J. Laude, C. W. Taylor and B. V. L. Potter, *J. Med. Chem.* 2003, **46**, 4860.

(4) P. Politzer, P. Lane, M. C. Concha, Y. Ma and J. S. Murray, *J. Mol. Model*, 2007, **13**, 305.
